# Asymmetric Biocatalytic Synthesis of 1‐Aryltetrahydro‐β‐carbolines Enabled by “Substrate Walking”

**DOI:** 10.1002/chem.202004449

**Published:** 2020-11-03

**Authors:** Elisabeth Eger, Joerg H. Schrittwieser, Dennis Wetzl, Hans Iding, Bernd Kuhn, Wolfgang Kroutil

**Affiliations:** ^1^ Institute of Chemistry, Biocatalytic Synthesis University of Graz, NAWI Graz, BioTechMed Graz Heinrichstrasse 28/II 8010 Graz Austria; ^2^ Process Chemistry & Catalysis F. Hoffmann-La Roche Ltd. Grenzacherstrasse 124 4070 Basel Switzerland; ^3^ Pharma Research & Early Development F. Hoffmann-La Roche Ltd. Grenzacherstrasse 124 4070 Basel Switzerland; ^4^ Field of Excellence BioHealth— University of Graz 8010 Graz Austria

**Keywords:** asymmetric catalysis, biocatalysis, carbolines, enzyme engineering, Pictet–Spengler reaction

## Abstract

Stereoselective catalysts for the Pictet–Spengler reaction of tryptamines and aldehydes may allow a simple and fast approach to chiral 1‐substituted tetrahydro‐β‐carbolines. Although biocatalysts have previously been employed for the Pictet–Spengler reaction, not a single one accepts benzaldehyde and its substituted derivatives. To address this challenge, a combination of substrate walking and transfer of beneficial mutations between different wild‐type backbones was used to develop a strictosidine synthase from *Rauvolfia serpentina* (*Rs*STR) into a suitable enzyme for the asymmetric Pictet–Spengler condensation of tryptamine and benzaldehyde derivatives. The double variant *Rs*STR V176L/V208A accepted various *ortho*‐, *meta*‐ and *para*‐substituted benzaldehydes and produced the corresponding chiral 1‐aryl‐tetrahydro‐β‐carbolines with up to 99 % enantiomeric excess.

## Graphical abstract

**Walking to new frontiers**: Taking the challenge that not a single biocatalyst was available for the Pictet–Spengler reaction with benzaldehyde derivatives, modern tools of enzyme engineering were applied in a joined forces approach. Thus, a catalyst displaying the desired activity was created, giving optically enriched products with up to 99 % *ee*.

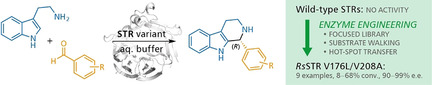

1‐Aryltetrahydro‐β‐carbolines have been shown to exert various biological activities: for instance, the phosphodiesterase‐5 inhibitor Tadalafil is used to treat erectile dysfunction and pulmonary arterial hypertension;^[1]^ 1‐phenyltetrahydro‐β‐carboline (**3 a**, Scheme 1) possesses biological activity binding to the human 5‐HT_7_ receptor^[2]^ and was proven effective as growth inhibitor of the African cotton leafworm (*Spodoptera littoralis*) in feeding bioassays.^[3]^ Interestingly, in the latter case and for many other non‐natural tetrahydro‐β‐carboline derivatives, mostly racemic compounds were tested, indicating a lack of suitable asymmetric methods.

**Scheme 1:**
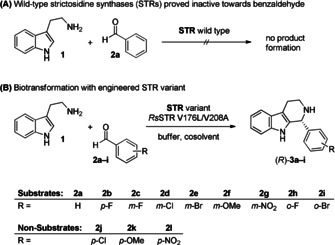
Biocatalytic Pictet–Spengler reaction using a strictosidine synthase variant to transform benzaldehyde. **(A)** The wild‐type STR does not transform benzaldehyde. **(B)** Substrate scope of *Rs*STR V176L/V208.

An efficient approach for the synthesis of 1‐substituted tetrahydro‐β‐carbolines is the Pictet–Spengler reaction, in which a 2‐arylethylamine reacts with a carbonyl compound, usually forming a six‐membered ring.[^4^, ^5^, ^6^] Various chemical protocols for the Pictet–Spengler reaction have been established, including many stereoselective approaches.[^5^, ^7^] In nature this condensation reaction is catalysed by enzymes called Pictet–Spenglerases,^[8]^ which are sub‐categorised according to their substrates. For instance, norcoclaurine synthases (NCSs)^[9]^ prefer dopamine as amine substrate, while strictosidine synthases (STRs)^[10]^ accept tryptamine and its derivatives, leading to β‐carbolines as products. While for norcoclaurine synthases a broad scope of carbonyl substrates has been reported,^[9]^ it has only recently been shown that STRs accept small aliphatic aldehydes besides the natural, highly functionalized aldehyde secologanin (**4 c**, Figure 1) and its analogues.^[10d]^ In contrast to the natural reaction of tryptamine (**1**) with secologanin (**4 c**), which gives the (*S*)‐configured product, (*S*)‐strictosidine, small aldehydes such as isovaleraldehyde led to the (*R*)‐configured product. Computational methods suggested the reason for this inverted absolute configuration to be an inverted binding mode: in the natural reaction the indole ring of tryptamine is located at the back of the active‐site pocket, while in the reaction with isovaleraldehyde it points to the outside of the active site.^[11]^

**Figure 1:**
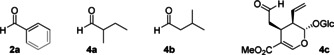
Aldehyde **4 a** used for substrate walking as structural transformant between the well accepted substrate **4 b** and the target substrate benzaldehyde (**2 a**), which is not accepted by the wild‐type enzymes. The structure of the natural substrate, secologanin (**4 c**, Glc=β‐d‐glucosyl), is shown for comparison.

Interestingly, acceptance of benzaldehyde as a substrate has neither been reported for NCSs nor for STRs. In fact, only very recently a variant of a norcoclaurine synthase has been shown to accept aldehydes branched in α‐position,^[9b]^ but benzaldehyde was not investigated in this work. An earlier study found no product formation from benzaldehyde and dopamine by NCS from *Thalictrum flavum*.^[9d]^

When testing heterologously expressed wild‐type strictosidine synthases originating from *Catharanthus roseus* (*Cr*STR),[^10d^, ^12^] *Ophiorrhiza pumila*, (*Op*STR),[^10d^, ^13^] and *Rauvolfia serpentina* (*Rs*STR, PDB: for example, 2V91)[^10d^, ^14^] with tryptamine (**1**) and benzaldehyde (**2 a**, Scheme 1 A), none of them led to any trace of product formation. Consequently, a new biocatalyst had to be developed for this type of substrate structure. Since STR has been shown to enable two binding modes of tryptamine and the aldehyde substrate (see above),^[11]^ it is not obvious which region of the active site needs to be modified to improve activity. Moreover, since benzaldehyde was not converted at all, a substrate walking^[15]^ approach was followed. The idea is to adapt the enzyme to a substrate possessing a structure, figuratively speaking, between the target substrate and a compound known to be well accepted, for example, isovaleraldehyde (**4 b**). Adapting the catalyst for the structural homologue might also induce low activity for the target aldehyde **2 a**, and this activity can then be further improved. As a smaller structural analogue of benzaldehyde, the α‐substituted aldehyde 2‐methylbutanal (**4 a**, Figure 1) was selected, which was accepted by the three STRs investigated, although the conversions observed after 24 h were low (0.8 % for *Op*STR, using 10 mm of **1** and 50 mm of **4 a**, Table S1). For comparison, the isomeric aldehyde isovaleraldehyde (**4 b**) is transformed under the same conditions with 50–63 % conversion.

Since *Op*STR proved to be a synthetically useful catalyst in a previous study,^[10b]^ the initial mutations were performed using this scaffold. In a focused library 13 residues inside and around the active‐site pocket were addressed as well as additional residues identified by MD simulations (Table S7; see also Supporting Information „Selection of Sites for Mutagenesis“).[^16^, ^17^] The 83 variants were successfully expressed; however, only 19 variants were active with aldehyde **4 a**. Out of these active variants four displayed minimally higher conversion than the wild type (Table 1, entries 2–5; Table S2). Next, double variants were prepared by pairwise combination of the beneficial substitutions in positions V147, I179 and L290. Additionally, the V147I and I179V substitutions were combined with amino acid exchanges that had resulted in similar activity as the wild type (Y76W, F197Y). This led to the identification of two *Op*STR double variants (V147I/I179V, V147I/L290I) displaying a four‐fold increase of conversion for **4 a** compared to the wild type (Table 1, entries 6, 7; data for all double variants: Table S3). When the small library of double variants was tested with benzaldehyde (**2 a**), formation of the desired product **3 a** was detected for the first time. Thereby *Op*STR V147I/I179V proved to be best (3 % conv.; Table 1, entry 6).

**Table 1:** Developing a Pictet–Spenglerase for the asymmetric condensation of benzaldehyde (**2 a**) and tryptamine (**1**) via identification of hot spots in the *Op*STR backbone and transferring these to *Rs*STR.^[a]^

Entry	Backbone	Mutation	Conv.^[b]^ **4 a** [%]	Conv.^[b]^ **2 a** [%]
1	*Op*STR	none	0.8	n.c.
2	*Op*STR	V147L	0.9	n.c.
3	*Op*STR	V147I	0.9	n.c.
4	*Op*STR	I179V	1.2	n.c.
5	*Op*STR	L290I	0.9	n.c.
6	*Op*STR	V147I/I179V	3.5	3
7	*Op*STR	V147I/L290I	3.5	n.c.
8	*Rs*STR	none	1.0	n.c.
9	*Rs*STR	V176L	5.5	3
10	*Rs*STR	V208A	3.0	13
11	*Rs*STR	V176L/V208A	10.1	24

[a] Reaction conditions: **1** (10 mm), aldehyde (**4 a** or **2 a**; 50 mm; **2 a** added as stock solution in DMSO; final DMSO conc.: 10 % *v*/*v*), STR preparation (50 mg mL^−1^ lyophilised cells), PIPES buffer (0.5 mL; 50 mm, pH 6.1), 35 °C, 650 rpm, 24 h. Conversion determined by GC analysis (**4 a**) or HPLC analysis (**2 a**). n.c.=no conversion. Blank reactions (no enzyme added) resulted in conversions <0.1 %.

Since the residues V147 and I179 seemed to represent hot spots in *Op*STR, we hypothesized that exchanges at the corresponding positions might also be beneficial for the strictosidine synthase of *Rauvolfia serpentina* (*Rs*STR). Such an approach^[18]^ to identify hot spots in one enzyme and then transfer them to related enzymes may enable screening a broader overall sequence space of possibly suitable candidates. The positions V147 and I179 of *Op*STR correspond to residues V176 and V208 in *Rs*STR.^[19]^ When first investigating single variants for these positions, V208A allowed already a reasonable conversion of benzaldehyde under standard assay conditions (13 %, Table 1, entry 10). In the double variant V176L/V208A, the exchange of V176 to leucine even increased the conversion of **2 a** to 24 % (Table 1, entry 11), while other amino acids in this position such as I, F or M were less beneficial (Table S4).

To better understand the observed reactivity with respect to the non‐natural aldehydes of the V176L/V208A mutation we generated a binding mode model (Figures 2 and 3) based on X‐ray crystal structure overlays and computational refinement (for computational details, see the Supporting Information). Our most likely model assumes the inverted binding mode that was found previously for **4 b** in *Op*STR (PDB: 6s5q).^[11]^ We think that the exchange V208A creates space in the back of the active‐site pocket, making it better able to accommodate the phenyl moiety (Figure 2). The substitution of Val176 by the larger leucine residue leads, in our model, to modified dispersion interactions with the indole core, which might result in a more favourable positioning of the substrate for catalysis.

**Figure 2:**
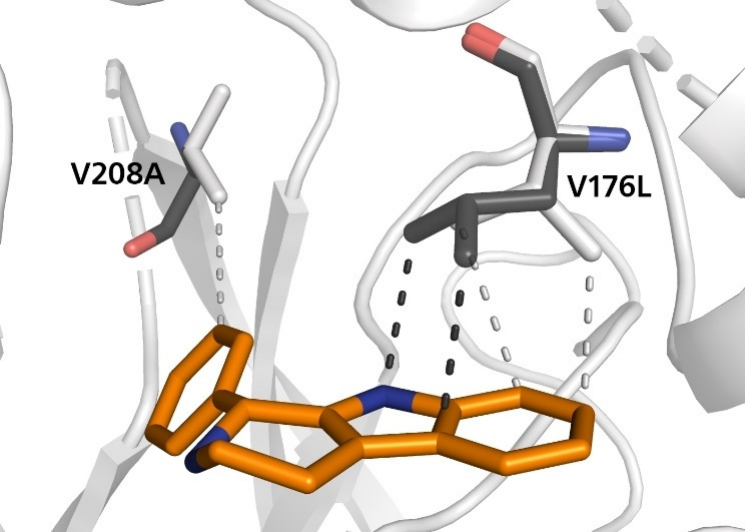
Model of (*R*)‐1‐phenyl‐β‐carboline (**3 a**) in the active site of wild‐type *Rs*STR (white) and *Rs*STR V176L/V208A (black). Short non‐bonded contacts between substrate and enzyme are shown as dashed lines.

**Figure 3:**
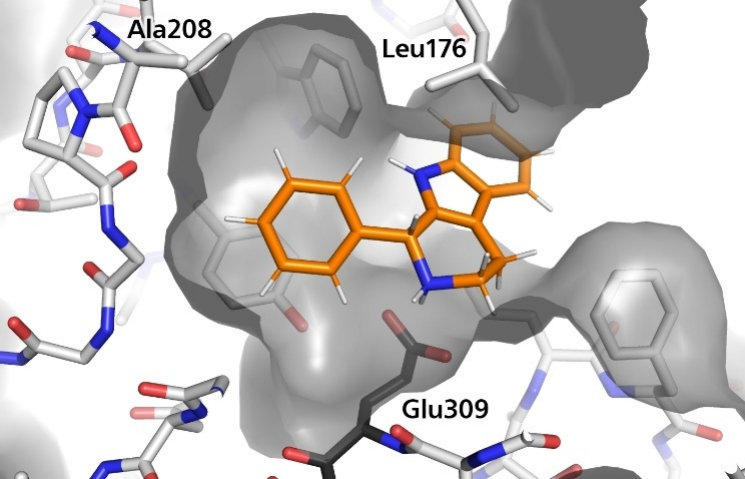
Model of (*R*)‐1‐phenyl‐β‐carboline (**3 a**) in the active site of *Rs*STR V176L/V208A with surface of the enzyme showing the space available in *meta*‐position in contrast to the limited space in *para*‐position. The catalytically essential active‐site residue Glu309 is shown in black.

It is also worth to note that *Rs*STR V176L/V208A is about twice as active towards the previously reported substrate isovaleraldehyde (**4 b**) as the wild‐type enzyme, while its specific activity with the natural substrate secologanin (**4 c**) is reduced to approx. 58 % relative to the wild type (Table S5).

Analysis of the optical purity of the obtained product **3 a** revealed an *ee* of 99 % with (*R*)‐configuration. This absolute configuration is in line with the previously observed stereochemical outcome of *Rs*STR‐catalysed Pictet–Spengler reactions of small aldehydes.^[10b]^ Furthermore, the observed absolute configuration and the site of mutation that created space at the back of the active‐site pocket (V208A) to accommodate **2 a** also support a substrate binding mode in which the indole ring of tryptamine points out of the active site, as previously suggested by computational studies.^[11]^

To elucidate the substrate scope of *Rs*STR V176L/V208A, substituted benzaldehyde derivatives were tested and revealed a tolerance of the variant for various *meta*‐substituents, including halogens (F, Cl, Br), methoxy and nitro groups (substrates **2 c**–**2 g**). The best conversion was achieved with the *m*‐bromo derivative (**2 e**, Table 2, entry 5). Presence of a fluorine atom was also accepted in *para*‐position (substrate **2 b**, Table 2, entry 2), while larger substituents like chloro, methoxy, or nitro (**2 j**–**2 l**) were not tolerated there.

**Table 2:** Pictet–Spengler reaction between substituted benzaldehydes **2 a**–**i** and tryptamine (**1**) employing *Rs*STR V176L/V208A.^[a]^

Entry	Aldehyde **2**	Substituent R	Conv.^[b]^ [%]	*ee* ^[c]^ [%]
1	**2 a**	H	38±1	99 (*R*)
2	**2 b**	*p‐*F	8±1	97 (*R*)
3	**2 c**	*m‐*F	16±2	96
4	**2 d**	*m‐*Cl	29±3	98
5	**2 e**	*m‐*Br	33±3	98
6	**2 f**	*m‐*MeO	24±1	98
7	**2 g** ^[d]^	*m‐*NO_2_	21±1	90
8	**2 h**	*o‐*F	68±2	99 (*R*)
9	**2 i**	*o‐*Br	59±12	99 (*R*)

[a] Reaction conditions: **1** (10 mm), aldehyde (50 mm), STR preparation (50 mg mL^−1^ lyophilised cells), MOPS buffer (50 mm, pH 6.1), total volume 500 μL, 35 °C, 650 rpm, 24 h. [b] Conversion determined by HPLC analysis on an achiral stationary phase. Reactions were run in triplicates and conversion is reported as mean ± standard deviation. [c] Enantiomeric excess was determined by HPLC analysis on a chiral stationary phase. The absolute configuration is given in parentheses. [d] Substrate added as stock solution (500 mm) in DMSO. Final DMSO conc. 10 % v/v.

The reason that *meta*‐substituents are well accepted while there is a clear limitation in *para*‐position is most likely steric hindrance, as a model of (*R*)‐1‐phenyl‐β‐carboline (**3 a**) in the active site of *Rs*STR V176L/V208A revealed free space in *meta*‐position and restriction in *para*‐position (Figure 3).

Since the *ortho*‐position is closest to the carbaldehyde moiety, which is the site of reaction, it was expected that *ortho*‐substitution would not be tolerated. However, it turned out that *o*‐fluoro‐ (**2 h**) and even *o*‐bromobenzaldehyde (**2 i**) are well‐accepted substrates. Unexpectedly, the conversion even improved significantly for the *o*‐Br derivative (**2 i**) compared to the unsubstituted **2 a**, reaching up to 59 % (Table 2, entry 9).

The products **3 b**–**i** were obtained in optical purities of 96–99 % *ee*, the only exception being **3 g**, which was formed in 90 % *ee*. This reduced optical purity can be ascribed to the substantial non‐enzymatic background reactivity of **1** with **2 g** (2.1 % conversion in 24 h). The background reactivity of the other aldehydes is significantly lower (≤0.6 %).

Optimisation of the reaction conditions (see Supporting Information) allowed to improve the ratio of tryptamine (**1**) to aldehyde **2** to 1:1.25 at a tryptamine concentration of 40 mm. Four selected biotransformations (**2 a**,**b**,**h**,**i**) were carried out on preparative scale (5 mmol **1**) to confirm/establish the absolute configuration (Table S6). The products were isolated in 4–31 % yield and the optical purities obtained were between 96 and 98 % *ee*. Analysis of the optically enriched tetrahydro‐β‐carbolines by optical rotation, circular dichroism (CD) spectroscopy and HPLC showed that all of them possess (*R*)‐configuration (Table S15).

In summary, a Pictet–Spenglerase reaction was developed for the stereoselective reaction of tryptamine with benzaldehyde derivatives. Because benzaldehyde was not accepted at all by the investigated wild‐type enzymes, a substrate‐walking strategy was applied, in which suitable hot spots identified in one STR backbone (*Op*STR) were transferred to another (*Rs*STR). The *Rs*STR variant V176L/V208A turned out to accept a broad scope of benzaldehyde derivatives, particularly those substituted in *meta*‐ and *ortho*‐position, allowing to obtain (*R*)‐configured products with up to 99 % *ee*. The suitable catalyst was created by testing a rather small library of variants (ca. 100) by combining rational design, single‐site saturation and hot‐spot transfer to other backbones. The concept and the catalyst developed open new approaches for the synthesis of important bioactive 1‐aryltetrahydro‐β‐carbolines in optically enriched form.

## Conflict of interest

The authors declare no conflict of interest.

## Supporting information

As a service to our authors and readers, this journal provides supporting information supplied by the authors. Such materials are peer reviewed and may be re‐organized for online delivery, but are not copy‐edited or typeset. Technical support issues arising from supporting information (other than missing files) should be addressed to the authors.

Supplementary

Supplementary

Supplementary
