# Supplementary material for: Asymmetric Biocatalytic Synthesis of 1‐Aryltetrahydro‐β‐carbolines Enabled by “Substrate Walking”
Source: Chemistry. 2020 Nov 3;26(69):16281–5. doi: 10.1002/chem.202004449 (PMC7756766; doi:10.1002/chem.202004449)
Supplement: Supplementary file 2 — Supplementary [file CHEM-26-16281-s002.pdf]

Chemistry–A European Journal

Supporting Information

Asymmetric Biocatalytic Synthesis of 1-Aryltetrahydro-β-carbolines Enabled by "Substrate Walking"

Elisabeth Eger,[a] Joerg H. Schrittwieser,[a] Dennis Wetzl,[b] Hans Iding,[b] Bernd Kuhn,[c] and Wolfgang Kroutil*[a, d]

Chemistry–A European Journal

Supporting Information

Asymmetric Biocatalytic Synthesis of 1-Aryltetrahydro-β-carbolines Enabled by “Substrate Walking”

Elisabeth Eger,[a] Joerg H. Schrittwieser,[a] Dennis Wetzl,[b] Hans Iding,[b] Bernd Kuhn,[c] and Wolfgang Kroutil*[a, d]

Contents:

Compound Table S2

Supplementary Data S3

- Screening Results S3
- Optimisation of Reaction Conditions S4
- Specific Activities S8
- Preparative Biotransformations S10

Supplementary Methods S11

- General Methods and Materials S11
- Synthesis of Reference Compounds S11
- Selection of Sites for Mutagenesis S16
- Site-directed Mutagenesis S18
- Primers S18
- Plasmids S22
- Protein Expression and Purification S23
- Biotransformations S24
- Structural Bioinformatics and Computational Model Building S29
- Analytical Methods S30

NMR and MS Spectra S50

References S110

Compound Table

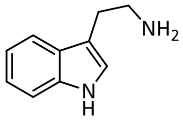

tryptamine (**1**)

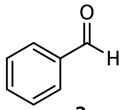

2a

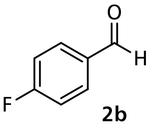

2b

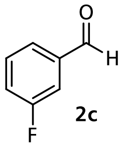

2c

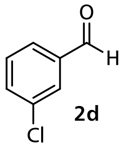

2d

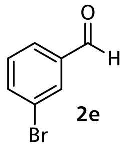

2e

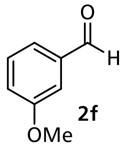

2f

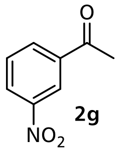

2g

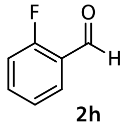

2h

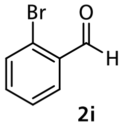

2i

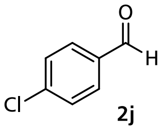

2j

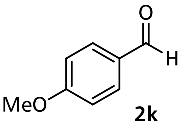

2k

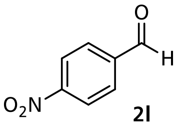

2l

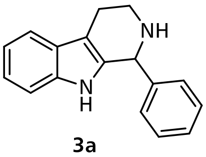

3a

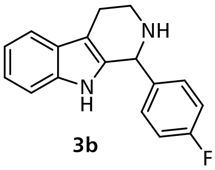

3b

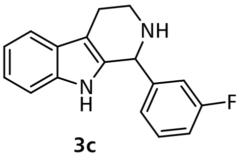

3c

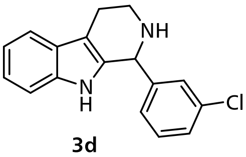

3d

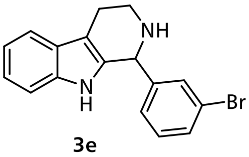

3e

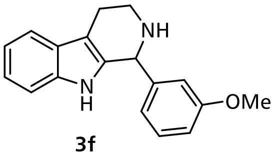

3f

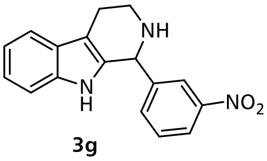

3g

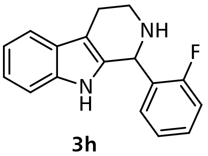

3h

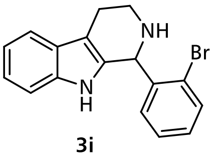

3i

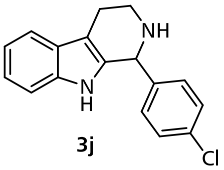

3j

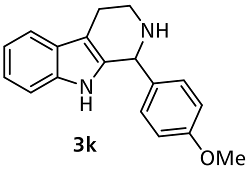

3k

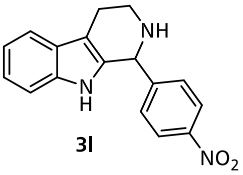

3l

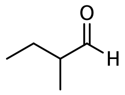

4a

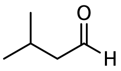

4b

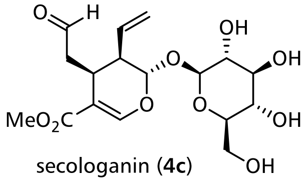

secologanin (**4c**)

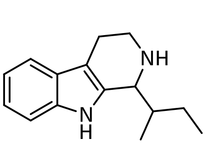

5a

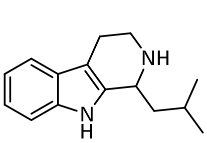

5b

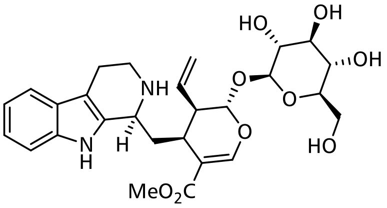

strictosidine (**5c**)

Supplementary Data

Screening Results

Three wild-type STRs as well as a total of 99 *Op*STR and *Rs*STR variants were screened for activity with tryptamine (**1**) and 2-methylbutyraldehyde (**4a**) or benzaldehyde (**2a**) as substrates. The results of these screenings are summarised in Tables S1–S4.

Table S1. Conversions observed for wild-type STRs in biotransformations between tryptamine (**1**) and 2-methylbutyraldehyde (**4a**) or benzaldehyde (**2a**).

	Conversion [%]	
**Enzyme**	**4a**[a]	**2a**[b]
*Cr*STR	0.2	–
*Op*STR	0.8	–
*Rs*STR	1.0	–

[a] Conversion into tetrahydro-β-carboline **5a** (sum of diastereomers) determined by GC–FID analysis on an achiral stationary phase, using dodecane (1 mM) as internal standard. [b] Conversion into tetrahydro-β-carboline **3a** determined by HPLC analysis on an achiral stationary phase. Reactions were run in duplicates, and the conversion is given as the mean value of both reactions. *Reaction conditions:* **1** (10 mM), aldehyde (**4a** or **2a**; 50 mM; **2a** added as stock solution in DMSO; final DMSO conc.: 10% *v/v*), STR preparation (50 mg/mL lyophilised cells), PIPES buffer (0.5 mL; 50 mM, pH 6.1), 35 °C, 650 rpm, 24 h.

Table S2. Conversions observed for *Op*STR variants in biotransformations between tryptamine (**1**) and 2-methylbutyraldehyde (**4a**). Variants leading to higher conversions than the wild type (*conv.* = 0.8%) are highlighted.

Entry	Variant[a]	Conversion[b] [%]
1	Y76W	0.7
2	Y76F	0.8
3	Y122F	0.3
4	V147L	0.9
5	V147I	0.9
6	I151A	0.3
7	I179A	0.6
8	I179L	0.4
9	I179V	1.2
10	F197Y	0.8
11	H274A	0.4
12	H274S	0.6
13	H274T	0.3
14	E276D	0.2
15	L290A	0.4
16	L290I	0.9
17	L290V	0.3
18	F291A	0.4
19	F291N	0.3

[a] Numbering of residues based on amino acid sequence lacking the N-terminal signal peptide. [b] Conversion into tetrahydro-β-carboline **5a** (sum of diastereomers) determined by GC–FID analysis on an achiral stationary phase, using dodecane (1 mM) as internal standard. Reactions were run in duplicates, and the conversion is given as the mean value of both reactions. *Reaction conditions:* **1** (10 mM), **4a** (50 mM), STR preparation (50 mg/mL lyophilised cells), PIPES buffer (0.5 mL; 50 mM, pH 6.1), 35 °C, 650 rpm, 24 h.

Table S3. Conversions observed for *Op*STR double variants in biotransformations between tryptamine (**1**) and 2-methylbutyraldehyde (**4a**) or benzaldehyde (**2a**).

		Conversion [%]	
**Entry**	**Variant**[a]	**4a**[b]	**2a**[c]
1	Y76W/I179V	1.3	–
2	V147I/I179V	3.5	3
3	I179V/F197Y	1.5	2
4	I179V/L290I	2.0	–
5	Y76W/V147I	1.8	–
6	V147I/F197Y	1.8	–
7	V147I/L290I	3.5	–

[a] Numbering of residues based on amino acid sequence lacking the N-terminal signal peptide. [b] Conversion into tetrahydro-β-carboline **5a** (sum of diastereomers) determined by GC–FID analysis on an achiral stationary phase, using dodecane (1 mM) as internal standard. [c] Conversion into tetrahydro-β-carboline **3a** determined by HPLC analysis on an achiral stationary phase. Reactions were run in duplicates, and the conversion is given as the mean value of both reactions. *Reaction conditions:* **1** (10 mM), aldehyde (**4a** or **2a**; 50 mM; **2a** added as stock solution in DMSO; final DMSO conc.: 10% *v/v*), STR preparation (50 mg/mL lyophilised cells), PIPES buffer (0.5 mL; 50 mM, pH 6.1), 35 °C, 650 rpm, 24 h.

Table S4. Conversions observed for *Rs*STR single variants ("hot spot" positions identified in *Op*STR) and double variants in biotransformations between tryptamine (**1**) and 2-methylbutyraldehyde (**4a**) or benzaldehyde (**2a**).

		Conversion [%]	
**Entry**	**Variant**[a]	**4a**[b]	**2a**[c]
1	V176A	<1	–
2	V176L	5.5	3
3	V176I	2.5	2
4	V208A	3.0	13
5	V176A/V208A	2.1	7
6	V176L/V208A	10.1	24
7	V176I/V208A	3.8	15
8	V176M/V208A	4.0	6
9	V176F/V208A	3.0	3

[a] Numbering of residues based on amino acid sequence including the N-terminal signal peptide. [b] Conversion into tetrahydro-β-carboline **5a** (sum of diastereomers) determined by GC–FID analysis on an achiral stationary phase, using dodecane (1 mM) as internal standard. [c] Conversion into tetrahydro-β-carboline **3a** determined by HPLC analysis on an achiral stationary phase. Reactions were run in duplicates, and the conversion is given as the mean value of both reactions. *Reaction conditions:* **1** (10 mM), aldehyde (**4a** or **2a**; 50 mM; **2a** added as stock solution in DMSO; final DMSO conc.: 10% *v/v*), STR preparation (50 mg/mL lyophilised cells), PIPES buffer (0.5 mL; 50 mM, pH 6.1), 35 °C, 650 rpm, 24 h.

Optimisation of Reaction Conditions

The biotransformation between tryptamine (**1**) and benzaldehyde (**2a**) catalysed by *Rs*STR V176L/V208A was optimised regarding the following parameters: addition of co-solvents, pH, temperature, tryptamine concentration, and biocatalyst loading.

The effect of different water-miscible co-solvents (10% v/v) on the conversion is shown in Figure S1. No significant positive influence of the addition of co-solvent on conversion was observed under these conditions compared to the reaction performed in buffer alone.

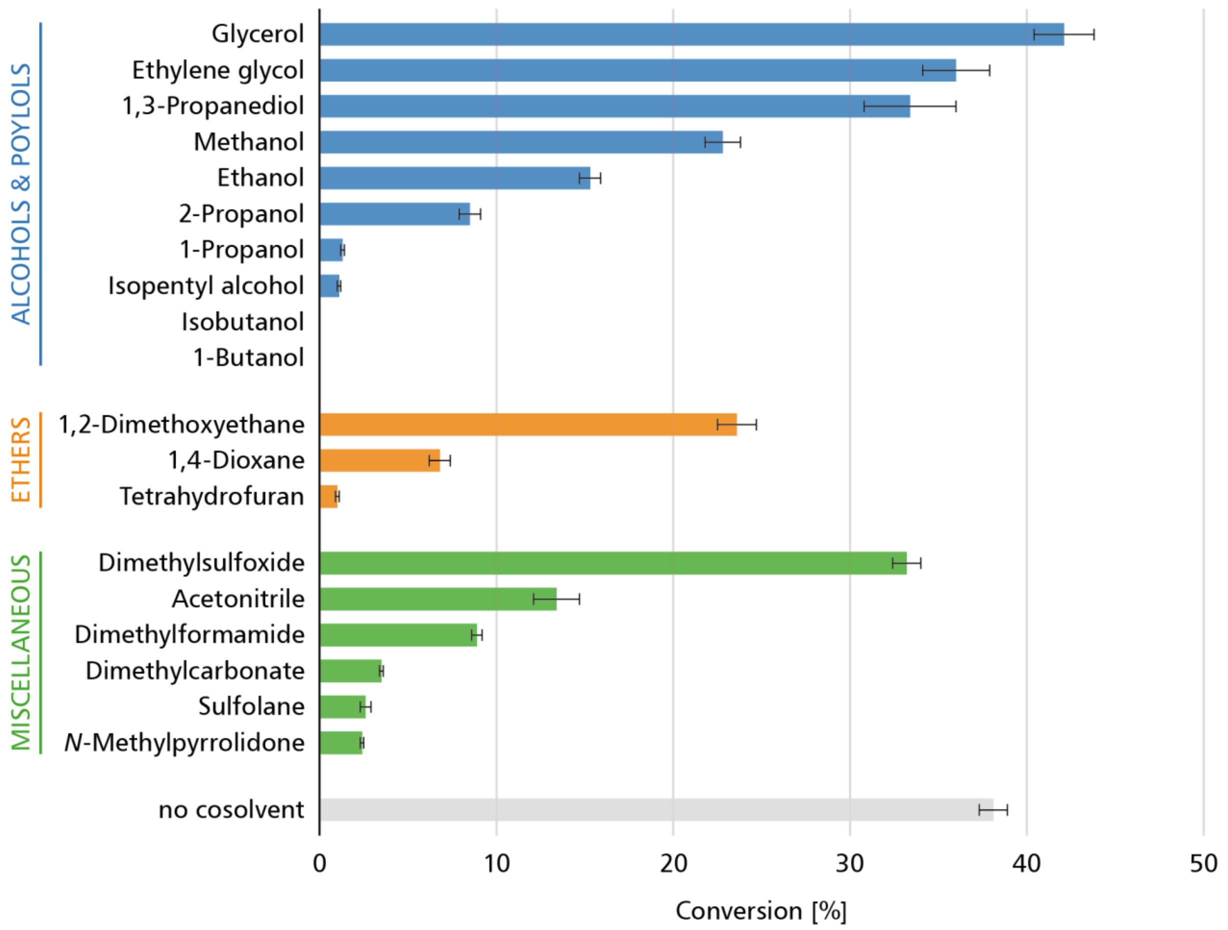

Figure S1. Conversions observed in the biotransformation between tryptamine (**1**) and benzaldehyde (**2a**) catalysed by *RsSTR* V176L/V208A in the presence of various water-miscible co-solvents (10% v/v). The conversion observed in the absence of cosolvent is shown for comparison. *Reaction conditions:* **1** (10 mM), **2a** (50 mM), STR preparation (50 mg/mL lyophilised cells), MOPS buffer (450 μL; 50 mM, pH 6.2), cosolvent (50 μL), 35 °C, 650 rpm, 24 h. Error bars represent standard deviations of triplicate experiments.

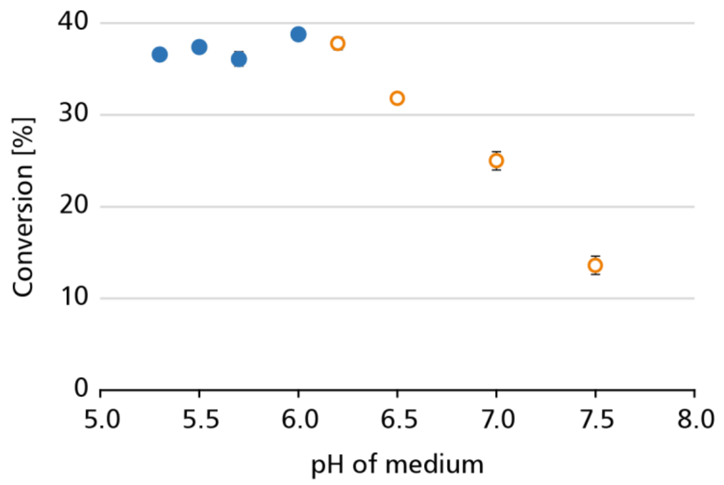

Figure S2. Conversions observed in the biotransformation between tryptamine (**1**) and benzaldehyde (**2a**) catalysed by *RsSTR* V176L/V208A at varied pH values, using MES (●) or MOPS (○) as buffer. *Reaction conditions:* **1** (10 mM), **2a** (50 mM), STR preparation (50 mg/mL lyophilised cells), buffer (0.5 mL; 50 mM, pH 5.3–7.5), 35 °C, 650 rpm, 24 h. Error bars represent standard deviations of triplicate experiments.

All further optimisation reactions were performed without the addition of any co-solvent. Varying the pH of the reaction medium revealed a broad pH optimum around the initially chosen value of pH 6 (Figure S2, see previous page). Similarly, the temperature optimum of the biotransformation was found to be broad, with reactions performed at 30 °C, 35 °C, and 37 °C giving almost identical conversions (Figure S3). The initially chosen reaction temperature of 35 °C was therefore retained for all further experiments.

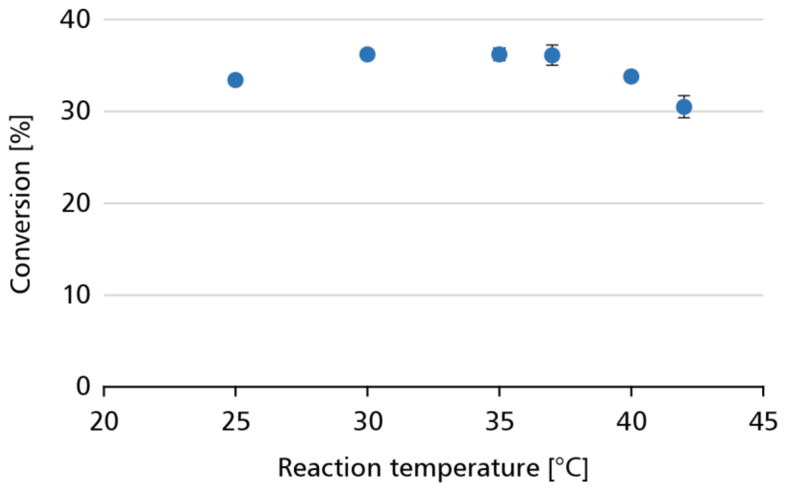

Figure S3. Conversions observed in the biotransformation between tryptamine (**1**) and benzaldehyde (**2a**) catalysed by *RsSTR* V176L/V208A at varied temperature. *Reaction conditions:* **1** (10 mM), **2a** (50 mM), STR preparation (50 mg/mL lyophilised cells), MOPS buffer (0.5 mL; 50 mM, pH 6.2), 25–42 °C, 650 rpm, 24 h. Error bars represent standard deviations of triplicate experiments.

To optimise the productivity of the reaction, the initial concentration of benzaldehyde (**2a**) was kept constant at 50 mM while the concentration of tryptamine (**1**) was increased from 10 mM to 80 mM. As shown in Figure S4, productivity (quantified as the product concentration after 24 h) increased between 10 mM and 40 mM of **1**, but levelled out at higher concentrations.

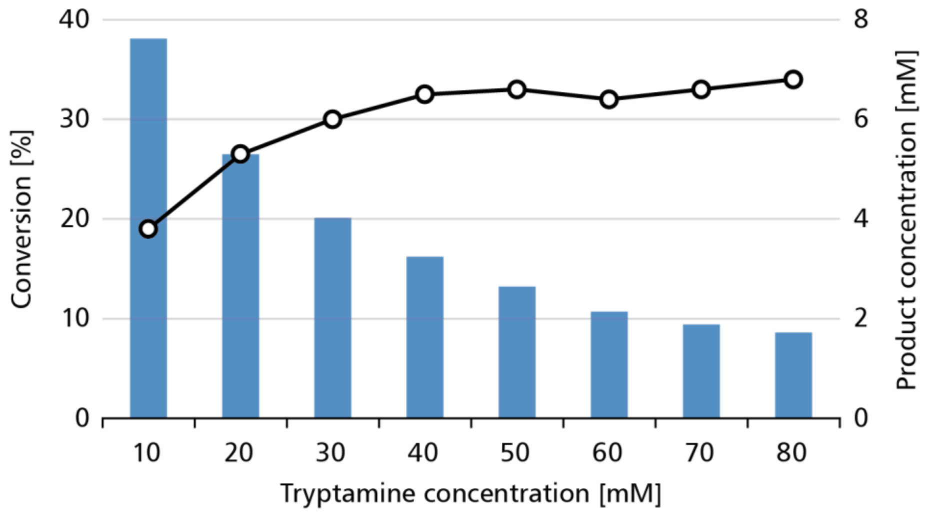

Figure S4. Conversions (bars) and product concentrations (circles) observed in the biotransformation between tryptamine (**1**) and benzaldehyde (**2a**) catalysed by *RsSTR* V176L/V208A at varied tryptamine concentration. *Reaction conditions:* **1** (10–80 mM), **2a** (50 mM), STR preparation (50 mg/mL lyophilised cells), MOPS buffer (0.5 mL; 50 mM, pH 6.2), 35 °C, 650 rpm, 24 h.

Finally, the biotransformation was performed with varied biocatalyst loading to investigate how conversion and productivity depend on the amount of cell mass employed. As expected, the conversion increased when the biocatalyst loading was raised from 50 mM to 100 mM, but productivity (expressed as the molar amount of product formed per gram cell mass per hour) slightly decreased (Figure S5). Consequently, all further biotransformations were performed using 50 mg/mL of lyophilised cells.

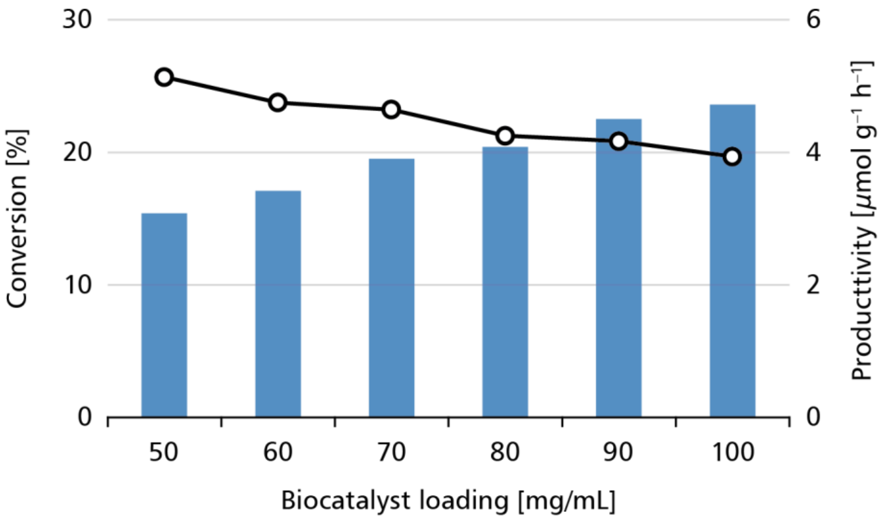

Figure S5. Conversions (bars) and productivities (circles) observed in the biotransformation between tryptamine (**1**) and benzaldehyde (**2a**) catalysed by *RsSTR* V176L/V208A at biocatalyst loadings. *Reaction conditions:* **1** (40 mM), **2a** (50 mM), STR preparation (50–100 mg/mL lyophilised cells), MOPS buffer (0.5 mL; 50 mM, pH 6.2), 35 °C, 650 rpm, 24 h.

Specific Activities

To gain insights into the effects of the mutations introduced in *Rs*STR on reaction kinetics, the specific activities of wild-type *Rs*STR and the best double variant, *Rs*STR V176L/V208A, were determined for reactions of tryptamine (**1**) with benzaldehyde (**2a**), 2-methylbutyraldehyde (**4a**), isovaleraldehyde (**4b**), and the natural substrate, secologanin (**4c**). To this end, biotransformations using purified STR were set up and followed over time by HPLC analysis of aliquots taken at regular intervals.

The pH value of the buffer used for biotransformations with isolated enzymes had to be raised to pH 7 to enhance protein stability, as the calculated isoelectric point of *Rs*STR (pI = 5.2)[1] is too close to the original buffer pH of 6.1, resulting in precipitation of the protein within the time frame of the experiment. Initial biotransformations further revealed that the purified STRs tend to precipitate upon incubation with benzaldehyde, and that this problem becomes more severe with increasing benzaldehyde concentration. Therefore, the concentration of both substrates in biotransformations with benzaldehyde (**2a**) was reduced to half of the standard conditions, *i.e.*, to 25 mM for **2a** and 5 mM for tryptamine (**1**). The natural reaction was set up with substrate concentrations of 2 mM for secologanin (**4c**) and 1 mM for tryptamine (**1**), as reported in a previous study.[2]

Despite these adjustments, only the reactions performed with **4a** as aldehyde substrate gave a progress curve (concentration of product over time) that was linear over the entire time frame of the experiment (30 min). The progress curves of the other biotransformations deviate significantly from an ideal, linear increase in product concentration, and this non-linearity is particularly pronounced in the natural reaction (Figure S6). We attribute this to differences in enzyme stability at different enzyme concentrations: The activity assay of **4a**, a poorly accepted substrate, required high enzyme concentration (0.5 mg/mL for *Rs*STR V176L/V208A, 5 mg/mL for *Rs*STR wild type) to achieve reliably quantifiable levels of product. In contrast, the natural reaction is so fast that it was complete within 5 min at 20 μg/mL *Rs*STR (wild-type) and had to be performed at 0.5 μg/mL (wild type and variant) to observe conversions <15% at 30 min. Apparently, this high dilution is detrimental to the stability of *Rs*STR.

To still obtain a useful estimate of the initial reaction rates, the collected data were fitted to an exponential equation by non-linear regression and the first derivative at $x = 0$ min was used to calculate the initial slope of the reaction progress curve. The only exception is the biotransformation using **4a**, for which the slope was determined by a linear fit. A detailed description of the calculations is provided in the *Supplementary Methods* section, the time-point data and curve fits are shown in Figure S6, and the specific activities calculated from these data are summarised in Table S5.

Table S5. Specific activities of wild-type *Rs*STR and *Rs*STR V176L/V208A in biotransformations between tryptamine (**1**) and four different aldehydes.

			Specific Activity [mU mg^{-1}]	
**Substrate**	**c(1)^{a} [mM]**	**c(ald)^{b} [mM]**	**wild type**	**V176L/V208A**
Benzaldehyde (**2a**)	5	25	*no activity*	3.72
2-Methylbutyraldehyde (**4a**)	10	50	0.077	0.85
Isovaleraldehyde (**4b**)	10	50	55.3	111
			**Specific Activity [U mg^{-1}]**	
Secologanin (**4c**)	1	2	34.4	19.9

^{a} Concentration of tryptamine (**1**). ^{b} Concentration of the aldehyde substrate. *General reaction conditions:* **1** (1–10 mM), aldehyde (2–50 mM), purified STR (0.5–5000 μg/mL), MOPS buffer (0.5 mL; 50 mM, pH 7.0), 35 °C, 650 rpm, 5–70 min.

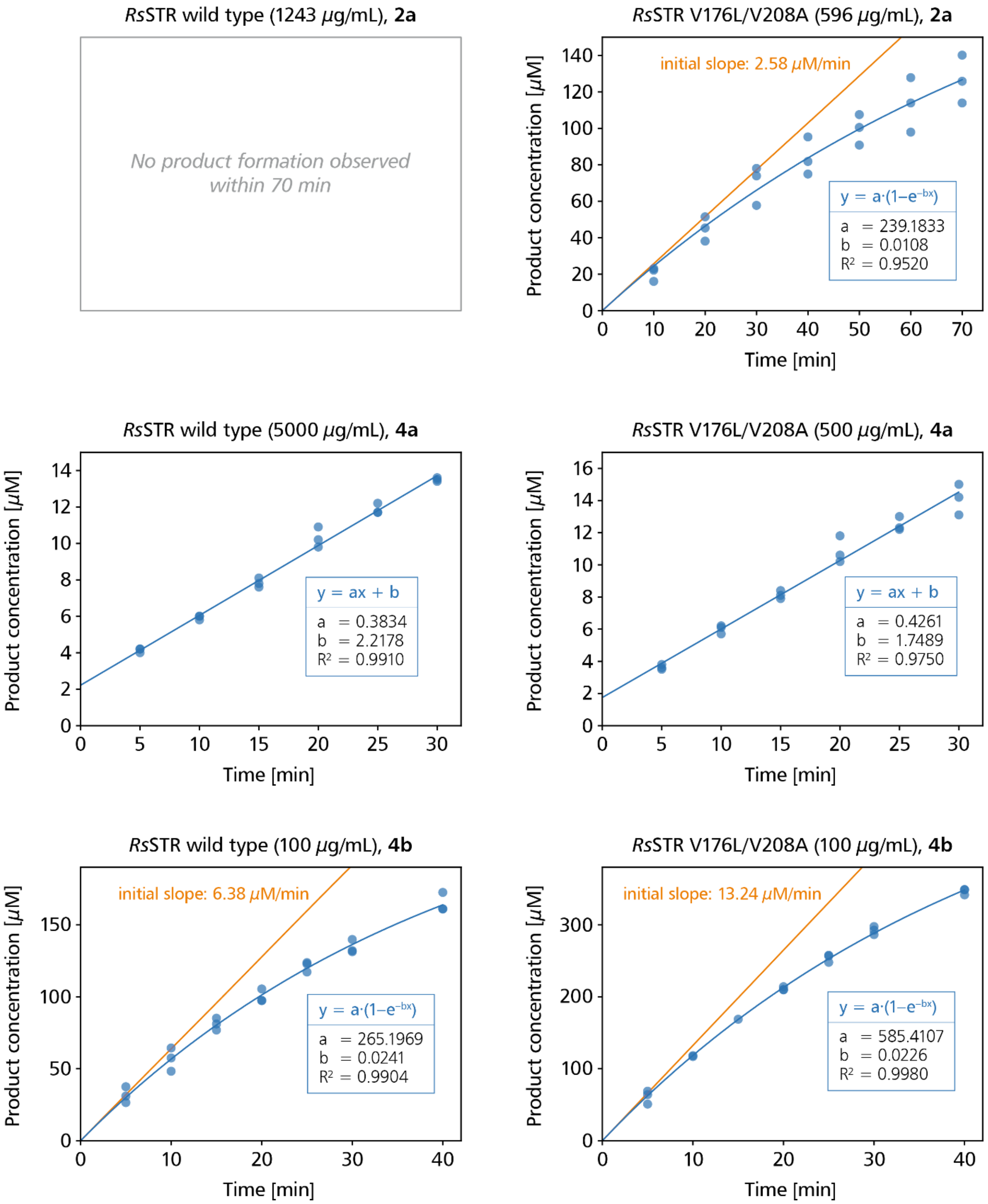

Figure S6. Time-point data and curve fits obtained for wild-type *Rs*STR and *Rs*STR V176L/V208A in biotransformations between tryptamine (**1**) and four different aldehydes. (*continued on next page*)

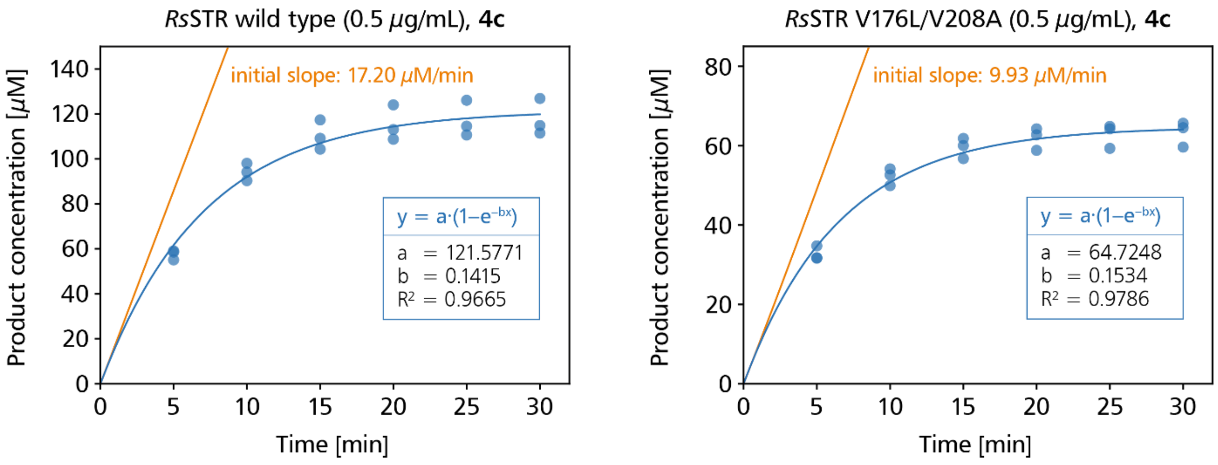

Figure S6. Time-point data and curve fits obtained for wild-type *Rs*STR and *Rs*STR V176L/V208A in biotransformations between tryptamine (**1**) and four different aldehydes. (*continued from previous page*)

Preparative Biotransformations

Four selected biotransformations were carried out on preparative scale (5 mmol **1**·HCl) and the products were isolated and purified by column chromatography. The conversions, yields, and optical purities obtained are summarised in Table S6.

Table S6. Preparative-scale biotransformations employing *Rs*STR V176L/V208A.

Entry	Product	Conv.[a] [%]	Yield[b] [%]	e.e.[c] [%]
1	**3a**	15	15	97 (*R*)
2	**3b**	6	4	96 (*R*)
3	**3h**	45	24	98 (*R*)
4	**3i**	32	31	98 (*R*)

[a] Conversion determined by HPLC analysis on an achiral stationary phase. [b] Isolated yield of tetrahydro-β-carboline **3** (free base) after column chromatography. [c] Enantiomeric excess determined by HPLC analysis on a chiral stationary phase. The absolute configuration is given in parentheses. *General reaction conditions:* **1** (40 mM), aldehyde (50 mM), STR preparation (50 mg/mL lyophilised cells), MOPS buffer (50 mM, pH 6.1), total volume 125 mL, 35 °C, 650 rpm, 48 h.

Supplementary Methods

General Methods and Materials

NMR spectra were recorded on a *Bruker* Avance III instrument operating at 300 MHz (^{1}H) and 75 MHz (^{13}C) and processed using the MestreReNova 9.0 software from *Mestrelab Research S.L.* (Santiago de Compostela, Spain). Chemical shifts are given in parts per million (ppm) relative to tetramethylsilane (δ = 0 ppm) and coupling constants (*J*) are reported in Hertz (Hz). The resolution of 2D spectra was enhanced using MestReNova's built-in "Resolution Booster" feature. Melting points were determined in open capillary tubes using the internal electronic thermometer of the melting point apparatus. Thin layer chromatography was carried out on silica gel 60 F_{254} plates and compounds were visualised by UV light. Unit resolution GC–MS analyses were performed using electron impact (EI) ionisation at 70 eV and quadrupole mass selection. High-resolution MS analyses were performed using electrospray ionisation (ESI) and time-of-flight mass selection. Optical rotation values $[\alpha]_D^{20}$ were measured at 589 nm (Na D-line) and 20 °C using a cuvette of 1 dm path length.

Unless otherwise noted, reagents and organic solvents were obtained from commercial suppliers in reagent grade quality and used without further purification. Liquid aldehydes used for biotransformations and for the synthesis of reference compounds were purified by short-path distillation (Kugelrohr) before use and stored under argon atmosphere.

Restriction enzymes and the corresponding buffers were purchased from *Thermo Fisher Scientific* (Vienna, Austria), and competent cells of *E. coli* NEB5α and *E. coli* BL21(DE3) were obtained from *New England Biolabs* (Frankfurt am Main, Germany).

Synthesis of Reference Compounds

Synthesis of racemic tetrahydro-β-carbolines (General procedure):[2-3] In a screw-top glass vial (4 mL), tryptamine hydrochloride (**1**·HCl; 197 mg, 1 mmol) was dissolved in maleic acid buffer (2 mL; 10 mM, pH 2.0), and aldehyde **2a–k** (1.2 mmol) was added. The vial was tightly sealed with a screw cap and the reaction mixture was magnetically stirred for 18 h at 60 °C in an aluminium heating block. After cooling to room temperature, the solid product was isolated by filtration using a Büchner funnel, washed with cold water, and dried over $CaCl_2$ in an evacuated desiccator overnight. The crude product was suspended in hot EtOAc (5 mL) and stirred for 10 min. The solid was recovered by filtration using a Büchner funnel, washed with warm EtOAc (10 mL), and dried in an evacuated desiccator overnight.

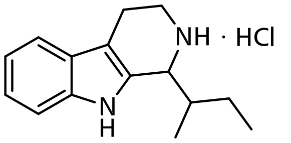

***all-rac*-1-(*sec*-Butyl)-2,3,4,9-tetrahydro-1*H*-pyrido[3,4-*b*]-indole hydrochloride (5a·HCl)**. Off-white solid, 146 mg (53%). m.p. 246–247 °C (decomp.). The product is a mixture of diastereomers (d.r. 56:44), to which the NMR signals were assigned based on signal intensities as well as COSY and HSQC correlations. *Major diastereomer:* ^{1}H-NMR (300 MHz, CD_3OD): δ [ppm] = 7.47 (dt, *J* = 7.8, 1.0 Hz, 1H), 7.38 (ddt, *J* = 8.1, 2.0, 0.9 Hz, 1H), 7.15 (ddd, *J* = 8.2, 7.0, 1.1 Hz, 1H), 7.06 (ddd, J = 8.0, 7.1, 1.0 Hz, 1H), 4.77 (dt, J = 3.4, 1.7 Hz, 1H), 3.80–3.67 (m, 1H), 3.41 (m, 1H), 3.21–2.91 (m, 2H), 2.47–2.26 (m, 1H), 1.69 (dtd, *J* = 14.8, 7.4, 5.8 Hz, 1H), 1.51 (ddq, *J* = 14.1, 8.8, 7.3 Hz, 1H), 1.13 (t, *J* = 7.4 Hz, 3H), 0.91 (d, *J* = 7.1 Hz, 3H). ^{13}C-NMR (75 MHz, CD_3OD): δ [ppm] = 138.4, 129.3, 127.5, 123.5, 120.6, 119.0, 112.4, 108.4, 59.2, 44.3, 37.8, 27.2, 19.4, 13.4, 12.5. *Minor diastereomer:* ^{1}H-NMR (300 MHz, CD_3OD): δ [ppm] = 7.47 (dt, *J* = 7.8, 1.0 Hz, 1H), 7.38 (ddt, *J* = 8.1, 2.0, 0.9 Hz, 1H),

7.15 (ddd, J = 8.2, 7.0, 1.1 Hz, 1H), 7.06 (ddd, J = 8.0, 7.1, 1.0 Hz, 1H), 4.70 (dt, J = 3.5, 1.7 Hz, 1H), 3.80–3.67 (m, 1H), 3.41 (m, 1H), 3.21–2.91 (m, 2H), 2.47–2.26 (m, 1H), 1.43–1.25 (m, 2H), 1.22 (d, J = 7.1 Hz, 3H), 0.99 (t, J = 7.3 Hz, 3H). ^{13}C-NMR (75 MHz, CD_3OD): δ [ppm] = 138.4, 129.1, 127.4, 123.5, 120.6, 119.0, 112.4, 108.4, 60.4, 44.1, 38.0, 24.7, 19.4, 15.9, 12.4. GC–MS (EI, 70 eV): m/z (%): 228 (3) $[M]^+$, 171 (100), 154 (6), 144 (6). HRMS (ESI): m/z calcd for $C_{15}H_{20}N_2$+H^+: 229.1699 $[M+H]^+$; found: 229.1701. No NMR reference data for this compound were available in the literature.

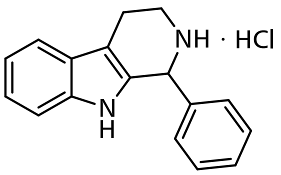

***rac*-1-Phenyl-2,3,4,9-tetrahydro-1*H*-pyrido[3,4-*b*]indole hydrochloride (3a·HCl)** [CAS 3574-01-4]. Colourless solid, 186 mg (65%). m.p. 271–273 °C (decomp.). ^{1}H-NMR (300 MHz, CD_3OD): δ [ppm] = 7.57 (dt, J = 7.7, 1.1 Hz, 1H), 7.56–7.50 (m, 3H), 7.49–7.44 (m, 2H), 7.32 (dt, J = 8.1, 1.0 Hz, 1H), 7.18 (ddd, J = 8.2, 7.1, 1.4 Hz, 1H), 7.11 (ddd, J = 8.1, 7.1, 1.2 Hz, 1H), 5.93 (s, 1H), 3.65 (dt, J = 12.6, 5.5 Hz, 1H), 3.56 (ddd, J = 12.8, 7.7, 5.6 Hz, 1H), 3.31–3.22 (m, 1H), 3.17 (dtd, J = 16.5, 5.5, 1.5 Hz, 1H). ^{13}C-NMR (75 MHz, CD_3OD): δ [ppm] = 137.1, 134.1, 130.1, 129.5, 129.1, 127.1, 125.8, 122.3, 119.2, 117.8, 111.1, 107.6, 56.9, 40.8, 18.1. GC–MS (EI, 70 eV): m/z (%): 248 (91) $[M]^+$, 218 (100), 171 (39), 115 (8), 109 (11). HRMS (ESI): m/z calcd for $C_{17}H_{16}N_2$+H^+: 249.1386 $[M+H]^+$; found: 249.1386. The NMR data are in agreement with literature values.[4]

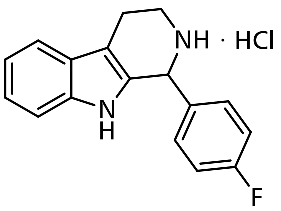

***rac*-1-(4-Fluorophenyl)-2,3,4,9-tetrahydro-1*H*-pyrido-[3,4-*b*]indole hydrochloride (3b·HCl)** [CAS 3380-82-3]. *Experimental note:* Washing with hot EtOAc as described in the general procedure was not sufficient to remove the remaining aldehyde in this case, but washing with boiling 2-PrOH was successful. Colourless solid, 71 mg (23%). m.p. 269–270 °C (decomp.). ^{1}H-NMR (300 MHz, CD_3OD): δ [ppm] = 7.57 (d, J = 7.7 Hz, 1H), 7.53–7.43 (m, 2H), 7.36–7.23 (m, 3H), 7.18 (ddd, J = 8.1, 7.0, 1.3 Hz, 1H), 7.11 (td, J = 7.5, 1.2 Hz, 1H), 5.97 (s, 1H), 3.66 (dt, J = 12.4, 5.5 Hz, 1H), 3.58 (ddd, J = 12.8, 7.5, 5.6 Hz, 1H), 3.31–3.22 (m, 1H), 3.18 (dtd, J = 16.5, 5.6, 1.5 Hz, 1H). ^{13}C-NMR (75 MHz, CD_3OD): δ [ppm] = 163.9 (d, J_{CF} = 248.9 Hz), 137.1, 131.8 (d, J_{CF} = 8.8 Hz), 130.2 (d, J_{CF} = 3.2 Hz), 126.9, 125.8, 122.4, 119.3, 117.9, 115.9 (d, J_{CF} = 22.2 Hz), 111.1, 107.7, 56.1, 40.7, 18.1. GC–MS (EI, 70 eV): m/z (%): 266 (87) $[M]^+$, 236 (100), 171 (28), 118 (14). HRMS (ESI): m/z calcd for $C_{17}H_{15}FN_2$+H^+: 267.1292 $[M+H]^+$; found: 267.1293. The ^{1}H-NMR data are in agreement with literature values.[5]

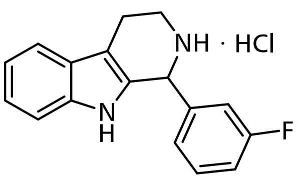

***rac*-1-(3-Fluorophenyl)-2,3,4,9-tetrahydro-1*H*-pyrido-[3,4-*b*]indole hydrochloride (3c·HCl)** [CAS 3380-81-2]. Off-white solid, 194 mg (64%). m.p. 266–267 °C (decomp.). ^{1}H-NMR (300 MHz, CD_3OD): δ [ppm] = 7.58–7.50 (m, 2H), 7.34–7.25 (m, 3H), 7.23–7.15 (m, 2H), 7.10 (td, J = 7.6 Hz, 1.1 Hz, 1H), 5.97 (s, 1H), 3.64 (dt, J = 12.6 Hz, 5.6, 1H), 3.55 (ddd, J = 12.8, 7.7, 5.7 Hz, 1H), 3.30–3.10 (m, 2H). ^{13}C-NMR (75 MHz, CD_3OD): δ [ppm] = 164.4 (d, J_{CF} = 246.9 Hz), 138.5, 138.0 (d, J_{CF} = 7.1 Hz), 132.4 (d, J_{CF} = 8.3 Hz), 127.9, 127.2, 126.9 (d, J_{CF} = 3.1 Hz), 123.9, 120.7, 118.4 (d, J_{CF} = 21.3 Hz), 117.8 (d, J_{CF} = 22.9 Hz), 112.5, 109.2, 57.6 (d, J_{CF} = 1.8 Hz), 42.3, 19.5. GC–MS (EI, 70 eV): m/z (%): 266 (88) $[M]^+$, 236 (100), 171 (26), 118 (10). HRMS (ESI): m/z calcd for $C_{17}H_{15}FN_2$+H^+: 267.1292 $[M+H]^+$; found: 267.1294. No NMR reference data for this compound were available in the literature.

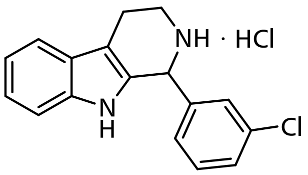

***rac*-1-(3-Chlorophenyl)-2,3,4,9-tetrahydro-1*H*-pyrido-[3,4-*b*]indole hydrochloride (3d·HCl)** [CAS 3380-79-8]. Colourless solid, 83 mg (26%). m.p. 257–258 °C (decomp.). ^{1}H-NMR (300 MHz, CD_3OD): δ [ppm] = 8.42 (ddd, J = 8.0, 2.3, 1.2 Hz, 1H), 8.38 (t, J = 2 Hz, 1H), 7.88–7.84 (m, 1H), 7.79 (t, J = 7.9 Hz, 1H), 7.61 (dt, J = 7.7, 1.1 Hz, 1H), 7.33 (d, J = 7.9 Hz, 1H), 7.21 (ddd, J = 8.2, 7.1, 1.4 Hz, 1H), 7.13 (ddd, J = 8.1, 7.1 Hz, 1.2 Hz, 1H), 6.16 (s, 1H), 3.70 (dt, J = 12.7 Hz, 5.6, 1H), 3.62 (ddd, J = 12.8, 7.7, 5.6 Hz, 1H), 3.35–3.26 (m, 1H), 3.20 (dtd, J = 16.6, 5.5, 1.4 Hz, 1H). ^{13}C-NMR (75 MHz, CD_3OD): δ [ppm] = 148.7, 137.2, 136.1, 135.7, 130.5, 126.0, 125.8, 124.9, 124.5, 122.7, 119.5, 118.0, 111.2, 108.2, 56.0, 41.1, 18.1. GC–MS (EI, 70 eV): m/z (%): 284 (33) $[M]^+$ (^{37}Cl), 282 (100) $[M]^+$ (^{35}Cl), 253 (44), 218 (86), 171 (66), 109 (22). HRMS (ESI): m/z calcd for $C_{17}H_{15}ClN_2+H^+$: 283.0997 $[M+H]^+$ (^{35}Cl); found: 283.1002. The ^{1}H-NMR data are in agreement with literature values.[6]

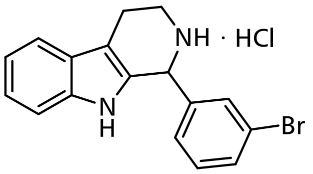

***rac*-1-(3-Bromophenyl)-2,3,4,9-tetrahydro-1*H*-pyrido-[3,4-*b*]indole hydrochloride (3e·HCl)**. Colourless solid, 20 mg (5%). m.p. 236–238 °C (decomp.). ^{1}H-NMR (300 MHz, CD_3OD): δ [ppm] = 7.72 (dt, J = 7.2, 2.0 Hz, 1H), 7.65 (t, J = 1.8 Hz, 1H), 7.58 (dt, J = 7.7, 1.1 Hz, 1H), 7.50–7.39 (m, 2H), 7.33 (dt, J = 8.2, 1.0 Hz, 1H), 7.19 (ddd, J = 8.2, 7.0, 1.3 Hz, 1H), 7.12 (ddd, J = 8.0, 7.1, 1.2 Hz, 1H), 5.96 (s, 1H), 3.66 (dt, J = 12.7, 5.6 Hz, 1H), 3.58 (ddd, J = 12.8, 7.7, 5.7 Hz, 1H), 3.31–3.21 (m, 1H), 3.17 (dtd, J = 16.4, 5.5, 1.4 Hz, 1H). ^{13}C-NMR (75 MHz, CD_3OD): δ [ppm] = 137.2, 136.5, 133.2, 132.5, 130.9, 128.4, 126.4, 125.8, 122.8, 122.5, 119.4, 117.9, 111.1, 107.9, 56.2, 40.9, 18.1. GC–MS (EI, 70 eV): m/z (%): 328 (64) $[M]^+$ (^{81}Br), 326 (72) $[M]^+$ (^{79}Br), 297 (26), 218 (100), 171 (86), 144 (13), 123 (12), 109 (24). HRMS (ESI): m/z calcd for $C_{17}H_{15}BrN_2+H^+$: 327.0491 $[M+H]^+$ (^{79}Br); found: 327.0496. No NMR reference data for this compound were available in the literature.

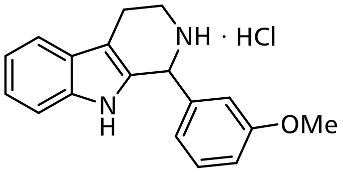

***rac*-1-(3-Methoxyphenyl)-2,3,4,9-tetrahydro-1*H*-pyrido-[3,4-*b*]indole hydrochloride (3f·HCl)** [CAS 98682-58-7]. Colourless solid, 158 mg (50%). m.p. 262–263 °C (decomp.). ^{1}H-NMR (300 MHz, CD_3OD): δ [ppm] = 7.57 (dt, J = 7.7, 1.1 Hz, 1H), 7.44 (t, J = 8.0 Hz, 1H), 7.32 (dt, J = 8.2, 1.0 Hz, 1H), 7.18 (td, J = 8.1, 7.6, 1.3 Hz, 1H), 7.14–7.05 (m, 2H), 7.04–6.95 (m, 2H), 5.90 (s, 1H), 3.82 (s, 3H), 3.67 (dt, J = 12.6, 5.5 Hz, 1H), 3.57 (ddd, J = 12.8, 7.8, 5.7 Hz, 1H), 3.31–3.22 (m, 1H), 3.17 (dtd, J = 16.5, 5.5, 1.4 Hz, 1H). ^{13}C-NMR (75 MHz, CD_3OD): δ [ppm] = 160.5, 137.1, 135.4, 130.2, 127.0, 125.8, 122.3, 121.4, 119.3, 117.8, 115.5, 114.9, 111.1, 107.5, 57.0, 54.5, 41.0, 18.1. GC–MS (EI, 70 eV): m/z (%): 278 (100) $[M]^+$, 249 (41), 218 (35), 204 (20), 171 (49). HRMS (ESI): m/z calcd for $C_{18}H_{18}N_2O+H^+$: 279.1492 $[M+H]^+$; found: 279.1495. No NMR reference data for this compound were available in the literature.

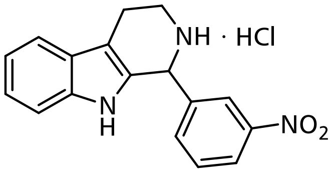

***rac*-1-(3-Nitrophenyl)-2,3,4,9-tetrahydro-1*H*-pyrido-[3,4-*b*]indole hydrochloride (3g·HCl)** [CAS 102207-59-0]. Pale-yellowish solid, 166 mg (50%). m.p. 231–233 °C (decomp.). ^{1}H-NMR (300 MHz, CD_3OD): δ [ppm] = 8.42 (ddd, J = 8.0, 2.3, 1.3 Hz, 1H), 8.38 (t, J = 2.0 Hz, 1H), 7.86 (dt, J = 7.7, 1.5 Hz, 1H), 7.79 (t, J = 7.9 Hz, 1H), 7.61 (d, J = 7.7 Hz, 1 H), 7.33 (d, J = 8.0 Hz, 1H), 7.21 (ddd, J = 8.2, 7.0, 1.4 Hz, 1H), 7.13 (ddd, J = 8.1, 7.1, 1.2 Hz, 1H), 6.16 (s, 1H), 3.74–3.58 (m, 2H), 3.37–3.26 (m, 1H), 3.20 (dtd, J = 16.5, 5.5, 1.5 Hz, 1H). ^{13}C-NMR (75 MHz, CD_3OD): δ [ppm] = 148.7, 137.2, 136.1, 135.7, 130.5, 126.0, 125.8, 124.9, 124.5, 122.7, 119.5, 118.0, 111.2, 108.2, 56.0, 41.1, 18.1. GC–MS (EI, 70 eV): m/z (%): 293 (100) $[M]^+$, 264 (42), 247

(33), 217 (85), 171 (71), 109 (17). HRMS (ESI): *m/z* calcd for $C_{17}H_{15}N_3O_2+H^+$: 294.1237 $[M+H]^+$; found: 294.1241. The ^{1}H-NMR data are in agreement with literature values.[7]

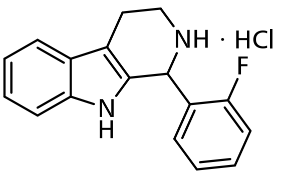

***rac*-1-(2-Fluorophenyl)-2,3,4,9-tetrahydro-1*H*-pyrido-[3,4-*b*]indole hydrochloride (3h·HCl)** [CAS 1177348-44-5]. Colourless solid, 194 mg (64%). m.p. 265–266 °C (decomp.). ^{1}H-NMR (300 MHz, CD_3OD): δ [ppm] = 7.63–7.56 (m, 2H), 7.40–7.36 (m, 1H), 7.33 (d, J = 7.9 Hz, 1H), 7.27 (dd, J = 7.4, 1.0 Hz, 1H), 7.22–7.15 (m, 2H), 7.11 (td, J = 7.5 Hz, 1.2 Hz, 1H), 6.25 (s, 1H), 3.68–3.55 (m, 2H), 3.31–3.22 (m, 1H), 3.19 (dtd, J = 16.5, 6.0, 1.5 Hz, 1H). ^{13}C-NMR (75 MHz, CD_3OD): δ [ppm] = 161.2 (d, J_{CF} = 249.3 Hz), 137.1, 132.5 (d, J_{CF} = 8.7 Hz), 131.0, 131.0, 125.8 (d, J_{CF} = 4.5 Hz), 124.9 (d, J_{CF} = 3.7 Hz), 122.5, 121.2 (d, J_{CF} = 13.1 Hz), 119.3, 117.9, 115.9 (d, J_{CF} = 21.3 Hz), 111.1, 108.0, 49.7 (d, J_{CF} = 4.9 Hz), 40.5, 18.1. GC–MS (EI, 70 eV): *m/z* (%): 266 (80) $[M]^+$, 236 (100), 217 (17), 171 (29), 118 (10). HRMS (ESI): *m/z* calcd for $C_{17}H_{15}FN_2+H^+$: 267.1292 $[M+H]^+$; found: 267.1292. No NMR reference data for this compound were available in the literature.

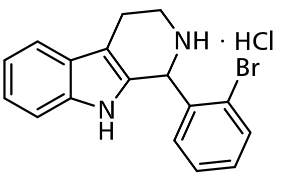

***rac*-1-(2-Bromophenyl)-2,3,4,9-tetrahydro-1*H*-pyrido-[3,4-*b*]indole hydrochloride (3i·HCl)**. Colourless solid, 142 mg (39%). m.p. 266–267 °C (decomp.). ^{1}H-NMR (300 MHz, CD_3OD): δ [ppm] = 7.92–7.80 (m, 1H), 7.59 (d, J = 7.8 Hz, 1H), 7.51–7.39 (m, 2H), 7.32 (d, J = 8.0 Hz, 1H), 7.24–7.05 (m, 3H), 6.37 (s, 1H), 3.65 (dt, J = 12.8, 5.8 Hz, 1H), 3.56 (ddd, J = 12.8, 7.4, 5.3 Hz, 1H), 3.31–3.24 (m, 1H), 3.19 (dddd, J = 16.6, 7.3, 5.9, 1.6 Hz, 1H). ^{13}C-NMR (75 MHz, CD_3OD): δ [ppm] = 137.2, 133.6, 133.2, 132.0, 131.5, 128.3, 126.1, 125.7, 125.0, 122.6, 119.4, 117.9, 111.1, 108.2, 55.5, 40.0, 18.1. GC–MS (EI, 70 eV): *m/z* (%): 328 (68) $[M]^+$ (^{81}Br), 326 (75) $[M]^+$ (^{79}Br), 297 (22), 243 (15), 218 (100), 171 (65), 123 (17), 109 (20). HRMS (ESI): *m/z* calcd for $C_{17}H_{15}BrN_2+H^+$: 327.0491 $[M+H]^+$ (^{79}Br); found: 327.0490. No NMR reference data for this compound were available in the literature.

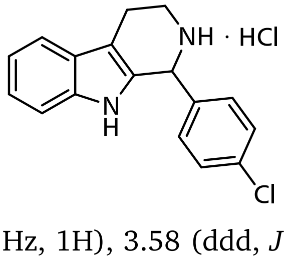

***rac*-1-(4-Chlorophenyl)-2,3,4,9-tetrahydro-1*H*-pyrido-[3,4-*b*]indole hydrochloride (3j·HCl)** [CAS 3380-80-1]. Off-white solid, 113 mg (35%). m.p. 277–278 °C (decomp.). ^{1}H-NMR (300 MHz, CD_3OD): δ [ppm] = 7.63–7.50 (m, 3H), 7.48–7.39 (m, 2H), 7.32 (dt, J = 8.2, 1.0 Hz, 1H), 7.18 (ddd, J = 8.2, 7.0, 1.3 Hz, 1H), 7.11 (ddd, J = 8.0, 7.1, 1.2 Hz, 1H), 5.97 (s, 1H), 3.66 (dt, J = 12.6, 5.7 Hz, 1H), 3.58 (ddd, J = 12.8, 7.4, 5.7 Hz, 1H), 3.31–3.22 (m, 1H), 3.18 (dtd, J = 16.6, 5.5, 1.4 Hz, 1H). ^{13}C-NMR (75 MHz, CD_3OD): δ [ppm] = 137.1, 136.2, 132.8, 131.2, 129.2, 126.6, 125.8, 122.5, 119.3, 117.9, 111.1, 107.8, 56.1, 40.7, 18.1. GC–MS (EI, 70 eV): *m/z* (%): 284 (33) $[M]^+$ (^{37}Cl), 282 (100) $[M]^+$ (^{35}Cl), 253 (43), 218 (96), 171 (48), 109 (25). HRMS (ESI): *m/z* calcd for $C_{17}H_{15}ClN_2+H^+$: 283.0997 $[M+H]^+$ (^{35}Cl); found: 283.0996. The NMR data are in agreement with literature values.[8]

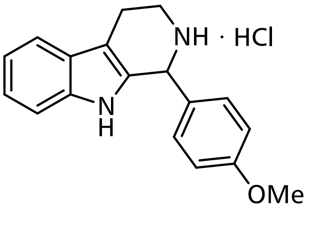

***rac*-1-(4-Methoxyphenyl)-2,3,4,9-tetrahydro-1*H*-pyrido-[3,4-*b*]indole hydrochloride (3k·HCl)** [CAS 3489-15-4]. Off-white solid, 103 mg (33%). m.p. 273–274 °C (decomp.). ^{1}H-NMR (300 MHz, CD_3OD): δ [ppm] = 7.56 (dt, J = 7.5, 1.1 Hz, 1H), 7.39–7.33 (m, 2H), 7.31 (dt, J = 8.2, 1.0 Hz, 1H), 7.17 (ddd, J = 8.2, 7.0, 1.4 Hz, 1H), 7.13–7.09 (m, 1H), 7.09–7.02 (m, 2H), 5.88 (t, J = 1.5 Hz, 1H), 3.85 (s, 3H), 3.65 (dt, J = 12.7, 5.6 Hz, 1H), 3.55 (ddd, J = 12.8, 7.6, 5.7 Hz, 1H), 3.31–3.22 (m, 1H), 3.16 (dtd, J = 16.5, 5.8, 1.5 Hz, 1H). ^{13}C-NMR (75 MHz, CD_3OD): δ [ppm]

= 161.4, 137.1, 130.8, 127.4, 125.9, 125.8, 122.3, 119.2, 117.8, 114.3, 111.0, 107.5, 56.5, 54.5, 40.6, 18.1. GC–MS (EI, 70 eV): *m/z* = 278 (M^+, 100), 249 (40), 218 (42), 204 (20), 171 (27). HRMS (ESI): *m/z* calcd for $C_{18}H_{18}N_2O+H^+$: 279.1492 $[M+H]^+$; found: 279.1490. The ^{1}H-NMR data are in agreement with literature values.[5]

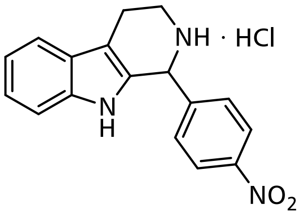

***rac*-1-(4-Nitrophenyl)-2,3,4,9-tetrahydro-1*H*-pyrido-[3,4-*b*]indole hydrochloride (3l·HCl)** [CAS 3380-77-6]. Yellowish solid, 161 mg (49%). m.p. 260–261 °C (decomp.). ^{1}H-NMR (300 MHz, CD_3OD): δ [ppm] = 8.43–8.32 (m, 2H), 7.80–7.65 (m, 2H), 7.60 (dt, J = 7.6, 1.0 Hz, 1H), 7.32 (dt, J = 8.2, 1.0 Hz, 1H), 7.20 (ddd, J = 8.2, 7.0, 1.3 Hz, 1H), 7.12 (ddd, J = 8.0, 7.1, 1.2 Hz, 1H), 6.14 (t, J = 1.5 Hz, 1H), 3.73–3.57 (m, 2H), 3.36–3.27 (m, 1H), 3.21 (dtd, J = 16.6, 5.7, 1.4 Hz, 1H). ^{13}C-NMR (75 MHz, CD_3OD): δ [ppm] = 149.2, 140.7, 137.2, 131.0, 126.0, 125.7, 123.9, 122.6, 119.5, 118.0, 111.1, 108.0, 55.8, 40.9, 18.1. GC–MS (EI, 70 eV): *m/z* = 293 (M^+, 78), 264 (31), 244 (28), 217 (100), 171 (53), 143 (17), 109 (16). HRMS (ESI): *m/z* calcd for $C_{17}H_{15}N_3O_2+H^+$: 294.1237 $[M+H]^+$; found: 294.1239. The NMR data are in agreement with literature values.[8]

Selection of Sites for Mutagenesis

The X-ray crystal structure of *Rs*STR in complex with strictosidine (PDB: 2v91) shows the indole moiety buried in the active-site pocket, with the sterically demanding and highly polar C1-substituent (derived from the natural aldehyde substrate, secologanin) pointing outwards into solution.[9] In contrast, recent experimental and computational studies suggest that the transformation of small, aliphatic aldehydes by wild-type STRs proceeds *via* a binding mode in which the indole moiety of tryptamine points out of the active site (PDB: 6s5q).[10] This inverted binding mode also results in inverted stereoselectivity, as tetrahydro-β-carbolines derived from small, aliphatic aldehydes are formed with (*R*)-stereopreference,[2] while the natural reaction leads to the exclusive formation of (*S*)-strictosidine.

In the context of the present study, it was initially unclear which binding mode would be preferred in the reaction of benzaldehyde or substituted benzaldehyde derivatives, as the phenyl ring is similar in size and electronic properties to the indole moiety. We therefore chose to vary a relatively large number of residues inside and around the active-site pocket. Since a high-throughput activity assay was not available, positions were varied individually by site-directed mutagenesis, rather than relying on single- or multi-site saturation strategies (*e.g.*, combinatorial active-site saturation test, CAST; iterative saturation mutagenesis, ISM)[11] that offer higher sequence diversity at the cost of increased screening effort.

The targeted positions can be categorised into four groups as shown in Table S7.

Table S7. *Op*STR variants designed for the focused library.

**Position**[a]	**Variants**
GROUP A: Aromatic residues in the active-site pocket	
Tyr76	Ala, Trp, Phe
Trp120	Ala, Phe, Tyr
Tyr122	Phe
Phe197	Tyr, Trp
GROUP B: Aliphatic residues in the active-site pocket	
Val138	Ala, Leu, Ile, Gly, Phe, Met, Pro, Ser, Thr, Tyr, His, Gln, Asn, Lys, Asp, Glu, Cys, Trp, Arg
Val147	Ala, Leu, Ile, Arg, Asn, Cys, Gln, Phe, His, Lys, Met, Pro, Ser, Thr
Ile151	Ala
Ile179	Ala, Leu, Gly, Val
Gly181	Ala
Leu290	Ala, Ile, Asn, Cys, Gln, Gly, His, Lys, Met, Pro, Ser, Arg, Val
GROUP C: "Gatekeeper" residues	
Asn243	Ala, Cys, Gly, Ile, Leu, Ser, Val
His274	Ala, Leu, Ser, Thr
Phe291	Ala, Asp, Asn
GROUP D: Mutations elsewhere (based on predictions from MD simulations)	
Tyr82	Glu
Tyr93	Phe
Asp102	Gly
Lys119	Ala
Gln148	Ala, Arg
Glu280	Ala

[a] Numbering of residues based on amino acid sequence lacking the N-terminal signal peptide.

Group A comprises four aromatic residues in the active-site pocket that might contribute to binding of aromatic substructures of the substrates by π–π-interactions. These positions were varied to other aromatic residues or alanine for enlarging the active-site pocket.

Group B comprises six aliphatic residues in the active-site pocket believed to directly interact with the indole moiety in the natural binding mode.

Group C consists of three positions at the entrance of the active site, termed "gatekeeper residues".

Group D comprises six residues distributed widely over the protein structure, whose conformations differed markedly in molecular dynamics (MD) simulations[12] run on the *Op*STR protein alone compared to simulations run on a complex of the protein and a reaction intermediate.

The location of the mutagenesis sites in the structure of *Op*STR is shown in Figure S7.

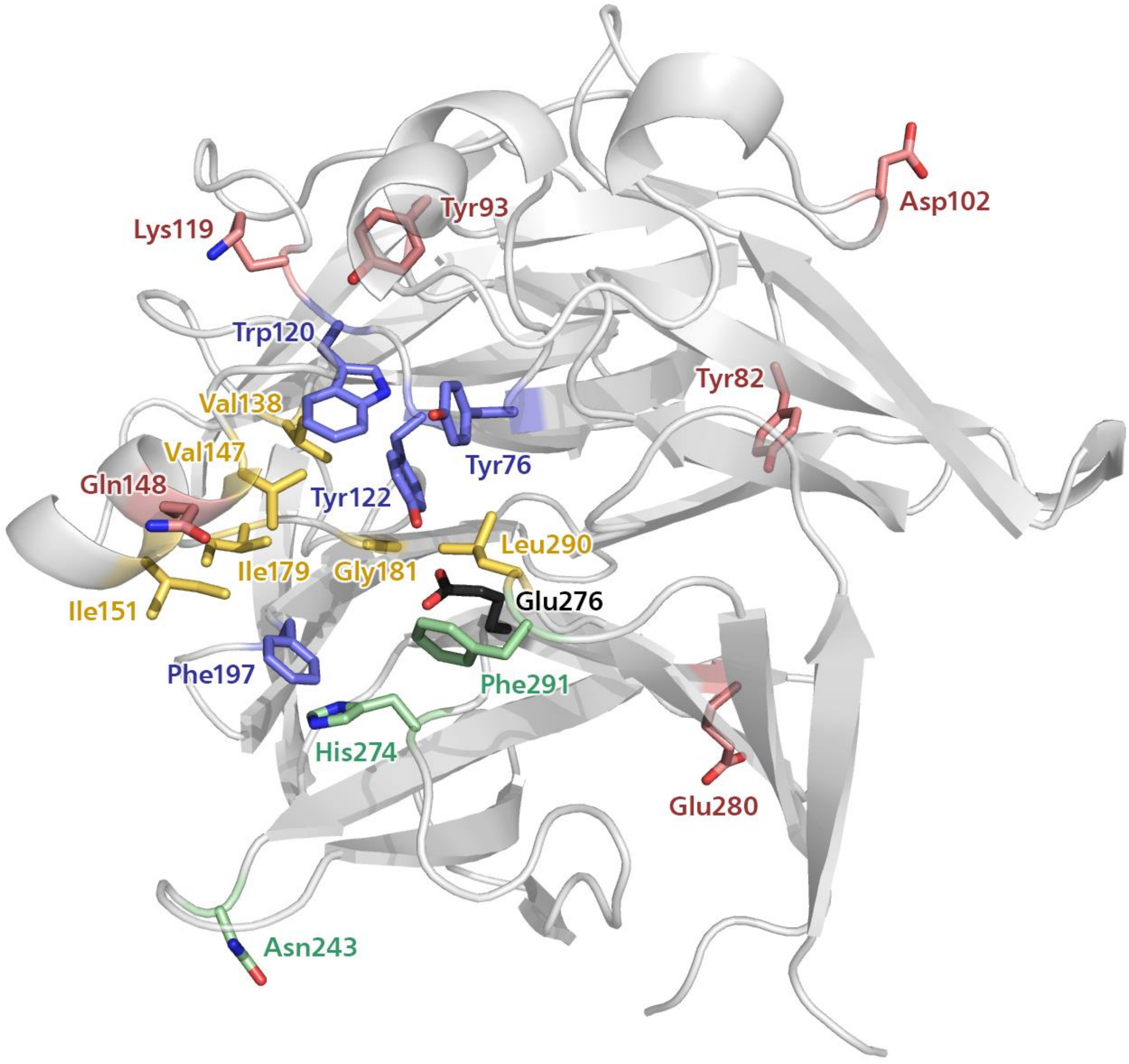

Figure S7. Location of mutagenesis sites in the structure of *Op*STR (PDB: 6s5q). The catalytically essential active-site residue Glu276 is shown in black. Mutagenesis positions of groups A–D are shown in blue (A), yellow (B), green (C), and red (D).

Site-directed Mutagenesis

Mutations were introduced using a two-step PCR protocol involving a linear amplification with one primer (forward or reverse) and subsequent full amplification of the plasmid with both primers. The PCR composition is specified in Table S8 and the temperature cycling parameters are given in Table S9. The primer sequences used are listed in the next section.

Table S8. Reaction composition for two-step mutagenesis PCR.

PCR Component	Volume [μL]	Amount	Final concentration
Phusion HF buffer	10		
Plasmid DNA	1.5	10 ng	0.2 ng/μL
Primer DNA (fw or rev)	1		0.5 μM
dNTP mix (10 mM)	1		200 μM
Phusion HF DNA polymerase	0.5	1 U	20 mU/μL
H_2O	36		
TOTAL:	50		

Table S9. Temperature cycling parameters of the two-step PCR protocol. Two individual reactions with forward and reverse primer were performed up to segment 4 and mixed before the start of segment 5.

Segment	Cycles	*T* [°C]	Time [min:s]	Comment
1	1	98	1:00	Initialisation
2	3	98	0:15	Denaturation
		65	0:30	Annealing
		72	15:00	Single-primer elongation
3	1	72	0:30	Final elongation
4		4	Unlimited	Intermediate Storage
5	1	98	1:00	Initialisation
6	15	98	0:30	Denaturation
		65	0:30	Annealing
		72	5:00	Double-primer elongation
7	1	72	5:00	Final elongation
8		4	Unlimited	Final Storage

After completion of the PCR, FastDigest™ buffer (6 μL), H_2O (3 μL), and FastDigest™ *Dpn*I (1 μL; 10 U/μL) were added to each amplification reaction (50 μL) and the samples were incubated for 6 h at 37 °C. The *Dpn*I-digested mixtures were used to transform *E. coli* Neb5α competent cells (*New England Biolabs*) according to the manufacturer's instructions. The correct introduction of mutations was confirmed by sequencing of plasmid DNA isolated from single bacterial colonies, using standard T7 promoter and terminator primers.

Primers

Primers were designed manually using common guidelines for PCR primer design, optimised to minimise secondary structure formation using the OligoAnalyzer online tool provided by *IDT*,[13] and ordered from *Eurofins Genomics* (Ebersberg, Germany). The primer sequences are listed in Table S10 (mutations in *Op*STR) and Table S11 (mutations in *Rs*STR).

Table S10. Primer sequences for mutations in the *OpSTR* gene. (*continued on next page*)

Variant[a]	ID[b]		Primer sequence
GROUP A: Aromatic residues in the active-site pocket			
Y76A	M1	fw:	CTGAAACCGCTGTGTGGTCGTGTTGCTGATTTTGGCTTTCATTA
		rev:	TAATGAAAGCCAAAATCAGCAACACGACCACACAGCGGTTTCAG
Y76W	M2	fw:	GTTTCATAATGAAAGCCAAAATCCCAAACACGACCACACAGCGGTTTC
		rev:	GAAACCGCTGTGTGGTCGTGTTTGGGATTTTGGCTTTCATTATGAAAC
Y76F	M3	fw:	GAAAGCCAAAATCAAAAACACGACCACACAGCGGTT
		rev:	AACCGCTGTGTGGTCGTGTTTTTGATTTTGGCTTTC
W120A	M4	fw:	CCAGTGCATACAGCGCTTTAAACTCAACACCATCACCGC
		rev:	GCGGTGATGGTGTTGAGTTTAAAGCGCTGTATGCACTGG
W120F	M5	fw:	AATTGCCAGTGCATACAGGAATTTAAACTCAACACCATCACCG
		rev:	CGGTGATGGTGTTGAGTTTAAATTCCTGTATGCACTGGCAATT
W120Y	M6	fw:	GCGGTGATGGTGTTGAGTTTAAATATCTGTATGCACTGGCAATTG
		rev:	CAATTGCCAGTGCATACAGATATTTAAACTCAACACCATCACCGC
W122F	M7	fw:	AATTGCCAGTGCAAACAGCCATTTAAACTCAACACCATC
		rev:	GATGGTGTTGAGTTTAAATGGCTGTTTGCACTGGCAATT
F197Y	M19	fw:	CGATGGCTTGCATATTCACCCACCAGAACAAAGCTA
		rev:	TAGCTTTGTTCTGGTGGGTGAATATGCAAGCCATCG
F197W	M20	fw:	TACGATGGCTTGCCCATTCACCCACCAGAACAAAGCTACC
		rev:	GGTAGCTTTGTTCTGGTGGGTGAATGGGCAAGCCATCGTA
GROUP B: Aliphatic residues in the active-site pocket			
V138A	M8	fw:	CGATCGTCATATTTGGTGCTAGCATCGGTAACATAAACAAAAC
		rev:	GTTTTGTTTATGTTACCGATGCTAGCACCAAATATGACGATCG
V138I	M9	fw:	CGATCGTCATATTTGGTGCTAATATCGGTAACATAAACAAAACCT
		rev:	AGGTTTTGTTTATGTTACCGATATTAGCACCAAATATGACGATCG
V138L	M10	fw:	GATCGTCATATTTGGTGCTAAGATCGGTAACATAAACAAAACC
		rev:	GGTTTTGTTTATGTTACCGATCTTAGCACCAAATATGACGATC
V138F	M68	fw:	GTTTTGTTTATGTTACCGATTTCAGCACCAAATATGACGATCG
		rev:	CGATCGTCATATTTGGTGCTGAAATCGGTAACATAAACAAAAC
V138M	M69	fw:	GTTTTGTTTATGTTACCGATATGAGCACCAAATATGACGATCG
		rev:	CGATCGTCATATTTGGTGCTCATATCGGTAACATAAACAAAAC
V138P	M70	fw:	GTTTTGTTTATGTTACCGATCCGAGCACCAAATATGACGATCG
		rev:	CGATCGTCATATTTGGTGCTCGGATCGGTAACATAAACAAAAC
V138S	M71	fw:	GTTTTGTTTATGTTACCGATAGCAGCACCAAATATGACGATCG
		rev:	CGATCGTCATATTTGGTGCTGCTATCGGTAACATAAACAAAAC
V138T	M72	fw:	GTTTTGTTTATGTTACCGATACCAGCACCAAATATGACGATCG
		rev:	CGATCGTCATATTTGGTGCTGGTATCGGTAACATAAACAAAAC
V138Y	M73	fw:	GTTTTGTTTATGTTACCGATTATAGCACCAAATATGACGATCG
		rev:	CGATCGTCATATTTGGTGCTATAATCGGTAACATAAACAAAAC
V138H	M74	fw:	GTTTTGTTTATGTTACCGATCATAGCACCAAATATGACGATCG
		rev:	CGATCGTCATATTTGGTGCTATGATCGGTAACATAAACAAAAC
V138Q	M75	fw:	GTTTTGTTTATGTTACCGATTAGAGCACCAAATATGACGATCG
		rev:	CGATCGTCATATTTGGTGCTCTAATCGGTAACATAAACAAAAC
V138N	M76	fw:	GTTTTGTTTATGTTACCGATAATAGCACCAAATATGACGATCG
		rev:	CGATCGTCATATTTGGTGCTATTATCGGTAACATAAACAAAAC
V138K	M77	fw:	GTTTTGTTTATGTTACCGATAAAAGCACCAAATATGACGATCG
		rev:	CGATCGTCATATTTGGTGCTTTTATCGGTAACATAAACAAAAC
V138D	M78	fw:	GTTTTGTTTATGTTACCGATGATAGCACCAAATATGACGATCG
		rev:	CGATCGTCATATTTGGTGCTATCATCGGTAACATAAACAAAAC
V138E	M79	fw:	GTTTTGTTTATGTTACCGATGAAAGCACCAAATATGACGATCG
		rev:	CGATCGTCATATTTGGTGCTTTCATCGGTAACATAAACAAAAC
V138C	M80	fw:	GTTTTGTTTATGTTACCGATTGCAGCACCAAATATGACGATCG
		rev:	CGATCGTCATATTTGGTGCTGCAATCGGTAACATAAACAAAAC

[a] Numbering of residues based on amino acid sequence lacking the N-terminal signal peptide.
[b] Internal identification code.

Table S10. Primer sequences for mutations in the *Op*STR gene. (*continued from previous page*)

Variant[a]	ID[b]		Primer sequence
V138W	M81	fw:	GTTTTGTTTATGTTACCGATTGGAGCACCAAATATGACGATCG
		rev:	CGATCGTCATATTTGGTGCTCCAATCGGTAACATAAACAAAAC
V138R	M82	fw:	GTTTTGTTTATGTTACCGATCGTAGCACCAAATATGACGATCG
		rev:	CGATCGTCATATTTGGTGCTACGATCGGTAACATAAACAAAAC
V147A	M12	fw:	TAATGCGAATGATATCCTGAGCACCACGATCGTCATATTTG
		rev:	CAAATATGACGATCGTGGTGCTCAGGATATCATTCGCATTA
V147L	M30	fw:	CAAATATGACGACCGTGGTCTGCAGGATATCATTCGC
		rev:	GCGAATGATATCCTGCAGACCACGGTCGTCATATTTG
V147I	M31	fw:	CAAATATGACGACCGTGGTATTCAGGATATCATTCGC
		rev:	GCGAATGATATCCTGAATACCACGGTCGTCATATTTG
V147R	M32	fw:	CAAATATGACGACCGTGGTCGCCAGGATATCATTCGC
		rev:	GCGAATGATATCCTGGCGACCACGGTCGTCATATTTG
V147N	M33	fw:	CAAATATGACGACCGTGGTAACCAGGATATCATTCGC
		rev:	GCGAATGATATCCTGGTTACCACGGTCGTCATATTTG
V147C	M34	fw:	CAAATATGACGACCGTGGTTGCCAGGATATCATTCGC
		rev:	GCGAATGATATCCTGGCAACCACGGTCGTCATATTTG
V147Q	M35	fw:	CAAATATGACGACCGTGGTCAGCAGGATATCATTCGC
		rev:	GCGAATGATATCCTGCTGACCACGGTCGTCATATTTG
V147G	M36	fw:	CAAATATGACGACCGTGGTGGTCAGGATATCATTCGC
		rev:	GCGAATGATATCCTGACCACCACGGTCGTCATATTTG
V147H	M37	fw:	CAAATATGACGACCGTGGTCATCAGGATATCATTCGC
		rev:	GCGAATGATATCCTGATGACCACGGTCGTCATATTTG
V147K	M38	fw:	CAAATATGACGACCGTGGTAAACAGGATATCATTCGC
		rev:	GCGAATGATATCCTGTTTACCACGGTCGTCATATTTG
V147M	M39	fw:	CAAATATGACGACCGTGGTATGCAGGATATCATTCGC
		rev:	GCGAATGATATCCTGCATACCACGGTCGTCATATTTG
V147P	M40	fw:	CAAATATGACGACCGTGGTCCGCAAGATATCATTCGC
		rev:	GCGAATGATATCTTGCGGACCACGGTCGTCATATTTG
V147S	M42	fw:	CAAATATGACGACCGTGGTAGCCAGGATATCATTCGC
		rev:	GCGAATGATATCCTGGCTACCACGGTCGTCATATTTG
V147T	M43	fw:	CAAATATGACGACCGTGGTACCCAGGATATCATTCGC
		rev:	GCGAATGATATCCTGGGTACCACGGTCGTCATATTTG
I151A	M13	fw:	GTGGTATCATTAATGCGAGCGATATCCTGAACACCACGATCG
		rev:	CGATCGTGGTGTTCAGGATATCGCTCGCATTAATGATACCAC
I179A	M14	fw:	GTGCCACCCGGAGCATTCAGACCTTTCATCAGAACGG
		rev:	CCGTTCTGATGAAAGGTCTGAATGCTCCGGGTGGCAC
I179L	M15	fw:	TGCCACCCGGAAGATTCAGACCTTTCATCAGAACG
		rev:	CGTTCTGATGAAAGGTCTGAATCTTCCGGGTGGCA
I179G	M16	fw:	GTGCCACCCGGACCATTCAGACCTTTCATCAGAACGG
		rev:	CCGTTCTGATGAAAGGTCTGAATGGTCCGGGTGGCAC
I179V	M17	fw:	TGCCACCCGGAACATTCAGACCTTTCATCAGAACG
		rev:	CGTTCTGATGAAAGGTCTGAATGTTCCGGGTGGCA
G181A	M18	fw:	GCTAACTTCGGTGCCAGCCGGAATATTCAGACC
		rev:	GGTCTGAATATTCCGGCTGGCACCGAAGTTAGC
L290I	M44	fw:	GCCCTGTTTGTTGGTAGCATTTTTCATGAATTTGTGGGC
		rev:	GCCCACAAATTCATGAAAAATGCTACCAACAAACAGGGC
L290R	M45	fw:	GCCCTGTTTGTTGGTAGCCGTTTTCATGAATTTGTGGGC
		rev:	GCCCACAAATTCATGAAAACGGCTACCAACAAACAGGGC
L290N	M46	fw:	GCCCTGTTTGTTGGTAGCAACTTTCATGAATTTGTGGGC
		rev:	GCCCACAAATTCATGAAAGTTGCTACCAACAAACAGGGC
L290C	M47	fw:	GCCCTGTTTGTTGGTAGCTGCTTTCATGAATTTGTGGGC
		rev:	GCCCACAAATTCATGAAAGCAGCTACCAACAAACAGGGC
L290Q	M48	fw:	GCCCTGTTTGTTGGTAGCCAGTTTCATGAATTTGTGGGC
		rev:	GCCCACAAATTCATGAAACTGGCTACCAACAAACAGGGC

[a] Numbering of residues based on amino acid sequence lacking the N-terminal signal peptide.
[b] Internal identification code.

Table S10. Primer sequences for mutations in the *Op*STR gene. (*continued from previous page*)

**Variant**[a]	**ID**[b]		**Primer sequence**
L290G	M49	fw:	GCCCTGTTTGTTGGTAGCGGCTTTCATGAATTTGTGGGC
		rev:	GCCCACAAATTCATGAAAGCCGCTACCAACAAACAGGGC
L290H	M50	fw:	GCCCTGTTTGTTGGTAGCCATTTTCATGAATTTGTGGGC
		rev:	GCCCACAAATTCATGAAAATGGCTACCAACAAACAGGGC
L290K	M51	fw:	GCCCTGTTTGTTGGTAGCAAATTTCATGAATTTGTGGGC
		rev:	GCCCACAAATTCATGAAAATGGCTACCAACAAACAGGGC
L290M	M52	fw:	GCCCTGTTTGTTGGTAGCATGTTTCATGAATTTGTGGGC
		rev:	GCCCACAAATTCATGAAACATGCTACCAACAAACAGGGC
L290P	M53	fw:	GCCCTGTTTGTTGGTAGCCCGTTTCATGAATTTGTGGGC
		rev:	GCCCACAAATTCATGAAACGGGCTACCAACAAACAGGGC
L290S	M55	fw:	GCCCTGTTTGTTGGTAGCAGCTTTCATGAATTTGTGGGC
		rev:	GCCCACAAATTCATGAAAGCTGCTACCAACAAACAGGGC
L290T	M56	fw:	GCCCTGTTTGTTGGTAGCACCTTTCATGAATTTGTGGGC
		rev:	GCCCACAAATTCATGAAAGGTGCTACCAACAAACAGGGC
L290V	M57	fw:	GCCCTGTTTGTTGGTAGCGTGTTTCATGAATTTGTGGGC
		rev:	GCCCACAAATTCATGAAACACGCTACCAACAAACAGGGC
GROUP C: “Gatekeeper” residues			
N243G	RO5	fw:	GCAAGCAGCGATAATGGTGGTATTACCGTTACACC
		rev:	GGTGTAACGGTAATACCACCATTATCGCTGCTTGC
N243A	RO6	fw:	GCAAGCAGCGATAATGCTGGTATTACCGTTACACC
		rev:	GGTGTAACGGTAATACCAGCATTATCGCTGCTTGC
N243V	RO7	fw:	GCAAGCAGCGATAATGTTGGTATTACCGTTACACC
		rev:	GGTGTAACGGTAATACCAACATTATCGCTGCTTGC
N243L	RO8	fw:	GCAAGCAGCGATAATCTGGGTATTACCGTTACACC
		rev:	GGTGTAACGGTAATACCCAGATTATCGCTGCTTGC
N243I	RO9	fw:	GCAAGCAGCGATAATATTGGTATTACCGTTACACC
		rev:	GGTGTAACGGTAATACCAATATTATCGCTGCTTGC
N243S	RO10	fw:	GCAAGCAGCGATAATTCTGGTATTACCGTTACACC
		rev:	GGTGTAACGGTAATACCAGAATTATCGCTGCTTGC
N243C	RO11	fw:	GCAAGCAGCGATAATTGTGGTATTACCGTTACACC
		rev:	GGTGTAACGGTAATACCACAATTATCGCTGCTTGC
H274A	M21	fw:	GTTCTTGCACCTGTTCAATAGCTTCACCTTTATACGGCAGCG
		rev:	CGCTGCCGTATAAAGGTGAAGCTATTGAACAGGTGCAAGAAC
H274L	M22	fw:	TTCTTGCACCTGTTCAATAAGTTCACCTTTATACGGCAG
		rev:	CTGCCGTATAAAGGTGAACTTATTGAACAGGTGCAAGAA
H274S	M23	fw:	GTTCTTGCACCTGTTCAATACTTTCACCTTTATACGGCAGCG
		rev:	CGCTGCCGTATAAAGGTGAAAGTATTGAACAGGTGCAAGAAC
H274T	M24	fw:	GTTCTTGCACCTGTTCAATAGTTTCACCTTTATACGGCAGCG
		rev:	CGCTGCCGTATAAAGGTGAAACTATTGAACAGGTGCAAGAAC
F291A	M27	fw:	GAATGCCCACAAATTCATGAGCCAGGCTACCAACAAACAGGG
		rev:	CCCTGTTTGTTGGTAGCCTGGCTCATGAATTTGTGGGCATTC
F291D	M28	fw:	GAATGCCCACAAATTCATGATCCAGGCTACCAACAAACAGGG
		rev:	CCCTGTTTGTTGGTAGCCTGGATCATGAATTTGTGGGCATTC
F291N	M29	fw:	CCCTGTTTGTTGGTAGCCTGAATCATGAATTTGTGGGCATTC
		rev:	GAATGCCCACAAATTCATGATTCAGGCTACCAACAAACAGGG
GROUP D: Mutations elsewhere (based on predictions from MD simulations)			
Y93F	M58	fw:	CCTGTATATTGCCGATTGTTTCTTTGGTCTGGGTTTTGTTGG
		rev:	CCAACAAAACCCAGACCAAAGAAACAATCGGCAATATACAGG
Q148A	M61	fw:	GACGATCGTGGTGTTGCGGATATCATTCGCATTAATG
		rev:	CATTAATGCGAATGATATCCGCAACACCACGATCGTC
Q148R	M64	fw:	GACGATCGTGGTGTTCGCGATATCATTCGCATTAATG
		rev:	CATTAATGCGAATGATATCGCGAACACCACGATCGTC

[a] Numbering of residues based on amino acid sequence lacking the N-terminal signal peptide. [b] Internal identification code.

Table S10. Primer sequences for mutations in the *Op*STR gene. (*continued from previous page*)

Variant[a]	ID[b]		Primer sequence
D102G	M62	fw:	GGTTTTGTTGGTCCGGGTGGTGGTCATGCAATTCAGC
		rev:	GCTGAATTGCATGACCACCACCCGGACCAACAAAACC
K119A	M63	fw:	GGTGATGGTGTTGAGTTTGCATGGCTGTATGCACTGGCAATTG
		rev:	CAATTGCCAGTGCATACAGCCATGCAAACTCAACACCATCACC
E280A	M66	fw:	CATATTGAACAGGTGCAAGCACATGATGGTGCCCTGTTTG
		rev:	CAAACAGGGCACCATCATGTGCTTGCACCTGTTCAATATG
Y82E	M67	fw:	GATTTTGGCTTTCATGAGGAAACCCAGCGCCTG
		rev:	CAGGCGCTGGGTTTCCTCATGAAAGCCAAAATC

[a] Numbering of residues based on amino acid sequence lacking the N-terminal signal peptide. [b] Internal identification code.

Table S11. Primer sequences for mutations in the *Rs*STR gene.

Variant[a]	ID[b]		Primer sequence
V176A	RR15	fw:	CCTGTATGATGATCGTGGTGCACAGCAGATTATGGATAC
		rev:	GTATCCATAATCTGCTGTGCACCACGATCATCATACAGG
V176L	RR16	fw:	CCTGTATGATGATCGTGGTCTGCAGCAGATTATGGATAC
		rev:	GTATCCATAATCTGCTGCAGACCACGATCATCATACAGG
V176I	RR17	fw:	CCTGTATGATGATCGTGGTATTCAGCAGATTATGGATAC
		rev:	GTATCCATAATCTGCTGAATACCACGATCATCATACAGG
V176M	RRD23	fw:	CCTGTATGATGATCGTGGTATGCAGCAGATTATGGATAC
		rev:	GTATCCATAATCTGCTGCATACCACGATCATCATACAGG
V176F	RRD24	fw:	CCTGTATGATGATCGTGGTTTTCAGCAGATTATGGATAC
		rev:	GTATCCATAATCTGCTGAAAACCACGATCATCATACAGG

[a] Numbering of residues based on amino acid sequence including the N-terminal signal peptide. [b] Internal identification code.

Plasmids

The plasmids encoding the wild-type STRs from *Rauvolfia serpentina* (*Rs*STR), *Ophiorrhiza pumila* (*Op*STR) and *Catharanthus roseus* (*Cr*STR) as well as the V208A variant of *Rs*STR have been described in a previous publication from our group.[2] The most important properties of these plasmids are summarised in Table S12. A ZIP-compressed folder containing plasmid maps (in Genbank format) of all used plasmids is provided as a separate Supporting Information file.

Table S12. Expression vectors used in the present study.

			Restriction Sites				
Internal ID	Vector	Insert	5′	3′	Antibiotic	Inducer[a]	Ref.
pEG290	pET28a(+)	*Rs*STR-V208A	*Nde*I	*Xho*I	Kanamycin	IPTG	[2]
pEG291	pET28a(+)	*Rs*STR	*Nde*I	*Xho*I	Kanamycin	IPTG	[2]
pEG292	pET28a(+)	*Op*STR	*Nde*I	*Xho*I	Kanamycin	IPTG	[2]
pEG289	pET28a(+)	*Cr*STR	*Nde*I	*Xho*I	Kanamycin	IPTG	[2]
pEG583	pET28a(+)	*Rs*STR-V176L-V208A	*Nde*I	*Xho*I	Kanamycin	IPTG	

[a] IPTG, isopropyl β-D-1-thiogalactopyranoside

Protein Expression and Purification

Expression of STRs in *E. coli* SHuffle T7 Express lysY:
Chemically competent cells of *E. coli* SHuffle T7 Express lysY (*New England Biolabs*) were transformed with the plasmids encoding the investigated STRs according to the cell supplier's instructions. A single colony from a transformant agar plate was used to inoculate LB medium (15 mL, cont. 50 μg/mL kanamycin) and the resulting culture was incubated at 30 °C and 120 rpm overnight in a closed plastic tube (50 mL). Glycerol stocks were prepared by diluting the cell suspension of the overnight culture (700 μL) with sterile 60% (v/v) aqueous glycerol (300 μL) and were stored at –20 °C (working stock) and –80 °C (backup stock). For expression, LB medium (15 mL, cont. 50 μg/mL kanamycin) was inoculated with 50 μL of cell suspension from a glycerol stock and the resulting culture was incubated at 30 °C and 120 rpm overnight in a closed plastic tube (50 mL). 3 mL of this overnight culture was used to inoculate TB medium (330 mL, cont. 50 μg/mL kanamycin) and the resulting culture was incubated at 37 °C and 120 rpm in a baffled Erlenmeyer flask (1 L). Samples (1 mL) of the culture were taken at regular intervals and analysed for their optical density at 600 nm (OD_{600}). When the OD_{600} reached a value of 0.7–0.8 (approx. after 3 h), protein expression was induced by the addition of IPTG (0.3 mM) and incubation was continued at 20 °C and 120 rpm overnight. The culture was transferred to a centrifuge beaker (500 mL) and centrifuged (4,000 rpm, 4 °C, 30 min) to pellet the cells. The supernatant was discarded and the cell pellet was resuspended in aq. NaCl solution (0.9% w/v; 15 mL). The cell suspension was transferred to a round-bottom flask (250 mL), flash-frozen in liquid nitrogen and lyophilised overnight to obtain the lyophilised whole-cell preparation (typically 0.8–1.0 g), which was stored at 4 °C until use.

Purification of wild-type *Rs*STR and *Rs*STR V176L/V208A by immobilised-metal affinity chromatography (IMAC): Wild-type *Rs*STR and *Rs*STR V176L/V208A were expressed as described above, using a total culture volume of 2 L. The cell pellets obtained by centrifugation (approx. 7 g) were resuspended in HisTrap buffer A (30 mL; Tris–HCl buffer, 50 mM Tris, 300 mM NaCl, 20 mM imidazole, pH 7.5), sonicated using a *Branson* Digital Sonifier 250 (30% amplitude, 1 s pulse, 4 s pause, total pulse time: 5 min) and centrifuged (14,000 rpm, 4 °C, 20 min). The supernatant was filtered (0.45 μm syringe filter) and loaded onto a 5 mL HisTrap FF column (*GE Healthcare*; Ni^{2+} on NTA-modified cross-linked agarose) that had been pre-equilibrated with HisTrap buffer A. The column was washed with the same buffer (20 column volumes) and the protein was eluted with buffer containing a higher imidazole concentration (HisTrap buffer B, 5 column volumes; 50 mM Tris, 300 mM NaCl, 50 mM imidazole, pH 7.5). The fractions were concentrated using VivaSpin 20 centrifugal filters (*Sartorius*, MWCO 30 kDa) and the buffer was exchanged to MOPS buffer (50 mM MOPS, pH 7.0) using a PD-10 desalting column (*GE Healthcare*). The protein concentration was determined photometrically (λ = 280 nm) using the extinction coefficient of wild-type *Rs*STR calculated by the ExPASy ProtParam online tool (ε_{280} = 47,330 M^{-1} cm^{-1}).[1] Aliquots were frozen in liquid nitrogen and stored at –20 °C.

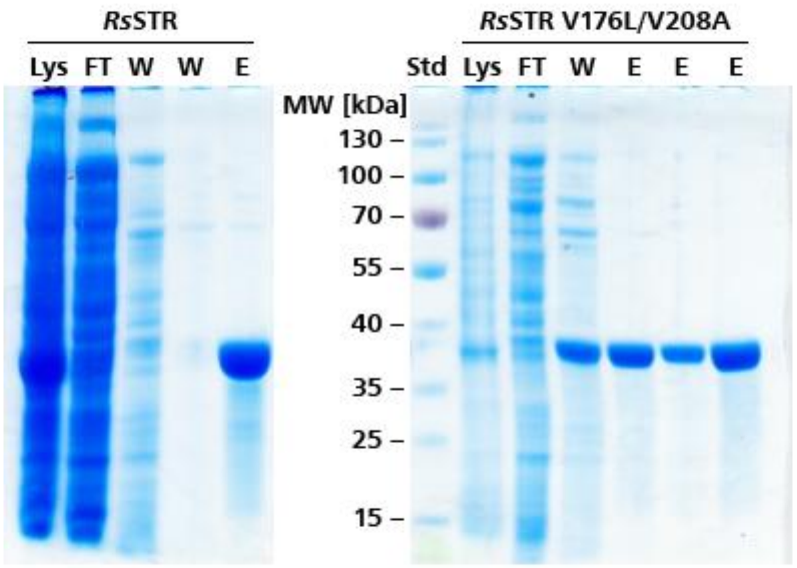

Figure S8. SDS-PAGE of the HisTrap purification of wild-type *Rs*STR and *Rs*STR V176L/V208A. Lys, lysate; FT, flow-through; W, wash; E, elution; Std, molecular weight standard (*Biorad* Precision Plus)

Biotransformations

STR reactions with 2-methylbutyraldehyde (4a) on analytical scale: Lyophilised cells of *E. coli* SHuffle T7 Express lysY expressing STR (25 mg) were resuspended in aqueous PIPES–tryptamine·HCl buffer (50 mM PIPES, 10 mM tryptamine·HCl, pH 6.1; 500 μL), and 2-methylbutyraldehyde (**4a**; 2.7 μL, 2.2 mg, 25 μmol; final conc. in reaction mixture: 50 mM) was added. The reaction mixture was incubated for 24 h on an orbital shaker at 35 °C and 650 rpm in vertical position. The reaction was then quenched by the addition of aq. NaOH solution (100 μL; 10 M) and extracted with ethyl acetate (2 × 500 μL). The combined organic phases were dried over Na_2SO_4, centrifuged (14,680 rpm, 1 min), and the supernatant was transferred to a glass vial for GC analysis.

STR reactions with benzaldehyde (2a) on analytical scale (screening conditions): Lyophilised cells of *E. coli* SHuffle T7 Express lysY expressing STR (25 mg) were resuspended in aqueous MOPS–tryptamine·HCl buffer (50 mM MOPS, 10 mM tryptamine·HCl, pH 6.1; 450 μL), and a solution of benzaldehyde (**2a**) in DMSO (500 mM **2a**; 50 μL; final conc. of **2a** in reaction mixture: 50 mM, final DMSO conc. 10% v/v) was added. The reaction mixture was incubated for 24 h on an orbital shaker at 35 °C and 650 rpm in vertical position. The reaction was then quenched by the addition of MeOH (500 μL), the mixture was thoroughly vortexed and incubated at room temperature for 30 min. Cells and precipitated protein were removed by centrifugation (14,680 rpm, 15 min), and the supernatant was filtered through a cotton plug and transferred to a glass vial for HPLC analysis.

Optimisation of reaction conditions: STR reactions with benzaldehyde (**2a**) were set up using cells expressing *Rs*STR V176L/V208A as described above, with the following adjustments: For the screening of cosolvents, stock solutions of **2a** (500 mM) in the investigated solvents were prepared, and 50 μL of the stock solution was added to the reaction mixture to achieve a final concentration of 50 mM **2a** and 10% v/v of solvent. Since a positive effect of cosolvent addition was not observed, all further optimisation reactions were performed in aqueous buffer (500 μL) without cosolvent. For the screening of pH values, the standard buffer (MOPS, 50 mM, pH 6.1) was replaced with MES or MOPS buffers (50 mM) of the specified pH. For the

temperature screening, the reactions were conducted in orbital shakers set to the temperatures specified. For the screening of tryptamine concentrations, a stock solution of tryptamine·HCl (**1**; 100 mM) in buffer (MOPS, 50 mM) was prepared and its pH adjusted to 6.1. This stock solution was mixed with buffer (MOPS, 50 mM, pH 6.1) to obtain the desired concentrations of **1**. For the screening of biocatalyst loadings, the reactions were conducted with a varied amount of lyophilised cells (25–50 mg).

STR reactions with aldehydes 2a–f, 2h, 2i, 2k on analytical scale (no organic co-solvent): Lyophilised cells of *E. coli* SHuffle T7 Express lysY expressing STR (25 mg) were resuspended in aqueous MOPS–tryptamine·HCl buffer (50 mM MOPS, 10 mM tryptamine·HCl, pH 6.1; 500 μL), and aldehyde (25 μmol; final conc. in reaction mixture: 50 mM) was added. The reaction mixture was incubated for 24 h on an orbital shaker at 35 °C and 650 rpm in vertical position. The reaction was then quenched by the addition of MeOH (500 μL), the mixture was thoroughly vortexed and incubated at room temperature for 30 min. Cells and precipitated protein were removed by centrifugation (14,680 rpm, 15 min), and the supernatant was filtered through a cotton plug and transferred to a glass vial for HPLC analysis.

STR reactions with aldehydes 2g, 2j on analytical scale (10% DMSO as organic co-solvent): Lyophilised cells of *E. coli* SHuffle T7 Express lysY expressing STR (25 mg) were resuspended in aqueous MOPS–tryptamine·HCl buffer (50 mM MOPS, 10 mM tryptamine·HCl, pH 6.1; 450 μL), and a solution of aldehyde in DMSO (500 mM aldehyde; 50 μL; final conc. of aldehyde in reaction mixture: 50 mM, final DMSO conc. 10% v/v) was added. The reaction mixture was incubated for 24 h on an orbital shaker at 35 °C and 650 rpm in vertical position. The reaction was then quenched by the addition of MeOH (500 μL), the mixture was thoroughly vortexed and incubated at room temperature for 30 min. Cells and precipitated protein were removed by centrifugation (14,680 rpm, 15 min), and the supernatant was filtered through a cotton plug and transferred to a glass vial for HPLC analysis.

STR reactions with aldehyde 2l on analytical scale (20% DMSO as organic co-solvent): Lyophilised cells of *E. coli* SHuffle T7 Express lysY expressing STR (25 mg) were resuspended in aqueous MOPS–tryptamine·HCl buffer (50 mM MOPS, 10 mM tryptamine·HCl, pH 6.1; 400 μL), and a solution of aldehyde **2l** in DMSO (250 mM **2l**; 100 μL; final conc. of **2l** in reaction mixture: 50 mM, final DMSO conc. 20% v/v) was added. The reaction mixture was incubated for 24 h on an orbital shaker at 35 °C and 650 rpm in vertical position. The reaction was then quenched by the addition of MeOH (500 μL), the mixture was thoroughly vortexed and incubated at room temperature for 30 min. Cells and precipitated protein were removed by centrifugation (14,680 rpm, 15 min), and the supernatant was filtered through a cotton plug and transferred to a glass vial for HPLC analysis.

Determination of specific activities with benzaldehyde (2a): A stock solution of tryptamine (**1**) in aqueous MOPS buffer (125 μL; 50 mM MOPS, 20 mM **1**·HCl, pH 7.0; final conc. of **1** in reaction mixture: 5 mM) was diluted in MOPS buffer (50 mM, pH 7.0; 344–364 μL), and benzaldehyde (**2a**; 1.3 μL, 1.3 mg, 12.5 μmol; final conc. in reaction mixture: 25 mM) was added. The reaction was started by adding a solution of purified STR (wild type: 29.6 μL, conc. of STR: 21 mg/mL, final conc. of STR in reaction mixture: 1243 μg/mL; V176L/V208A: 9.6 μL, conc. of STR: 31 mg/mL; final conc. of STR in reaction mixture: 596 μg/mL) to give a total volume of 500 μL, and the reaction mixture was incubated on an orbital shaker at 35 °C and 650 rpm in vertical position. Samples (50 μL) were taken 10, 20, 30, 40, 50, 60, and 70 min after starting the reaction and immediately quenched by the addition of MeOH (50 μL). The samples were centrifuged (14,680 rpm, 20 min), and the supernatants were transferred to plastic vials for HPLC analysis.

The obtained time-point data (x: time [min], y: product concentration [μM]) and the origin ($x = 0$, $y = 0$) were fitted to an exponential equation [eq. 1] using the non-linear curve-fit functionality of the SigmaPlot 14 software:

$$y = a \cdot (1 - e^{-bx}) \qquad \text{[eq. 1]}$$

The first derivative of [eq. 1] is:

$$y' = ab \cdot e^{-bx} \qquad \text{[eq. 2]}$$

Inserting $x = 0$ gives the initial slope of the progress curve as $y' = ab$, which was used for calculating the specific enzyme activity according to equation [eq. 3]:

$$A = y'/c_{\mathrm{E}} \qquad \text{[eq. 3]}$$

where A [U/mg] … specific enzyme activity; y' [μM/min] … initial slope of reaction progress curve; c_{E} [mg/L] … enzyme concentration

Determination of specific activities with 2-methylbutyraldehyde (4a): A stock solution of tryptamine (**1**) in aqueous MOPS buffer (250 μL; 50 mM MOPS, 20 mM **1**·HCl, pH 7.0; final conc. of **1** in reaction mixture: 10 mM) was mixed with 2-methylbutyraldehyde (**4a**; 2.7 μL, 2.2 mg, 25 μmol; final conc. in reaction mixture: 50 mM). The reaction was started by adding a solution of purified STR (250 μL; wild type: conc. of STR: 10 mg/mL, final conc. of STR in reaction mixture: 5000 μg/mL; V176L/V208A: conc. of STR: 1 mg/mL; final conc. of STR in reaction mixture: 500 μg/mL) to give a total volume of 500 μL, and the reaction mixture was incubated on an orbital shaker at 35 °C and 650 rpm in vertical position. Samples (50 μL) were taken 5, 10, 15, 20, 25, and 30 min after starting the reaction and immediately quenched by the addition of MeOH (150 μL). The samples were centrifuged (14,680 rpm, 10 min), and the supernatants were transferred to plastic vials for HPLC analysis.

The obtained time-point data (x: time [min], y: product concentration [μM]) were fitted to a linear equation [eq. 4] using the linear regression functionality of the SigmaPlot 14 software:

$$y = ax + b \qquad \text{[eq. 4]}$$

The slope a of the linear fit was used for calculating the specific enzyme activity according to equation [eq. 5]:

$$A = a/c_{\mathrm{E}} \qquad \text{[eq. 5]}$$

where A [U/mg] … specific enzyme activity; a [μM/min] … slope of reaction progress curve; c_{E} [mg/L] … enzyme concentration

Determination of specific activities with isovaleraldehyde (4b): A stock solution of tryptamine (**1**) in aqueous MOPS buffer (250 μL; 50 mM MOPS, 20 mM **1**·HCl, pH 7.0; final conc. of **1** in reaction mixture: 10 mM) was diluted in MOPS buffer (50 mM, pH 7.0; 245 μL), and isovaleraldehyde (**4b**; 2.7 μL, 2.2 mg, 25 μmol; final conc. in reaction mixture: 50 mM) was added. The reaction was started by adding a solution of purified STR (2.4 μL; wild type: conc. of STR: 21 mg/mL, final conc. of STR in reaction mixture: 100 μg/mL; V176L/V208A: conc. of STR: 31 mg/mL; final conc. of STR in reaction mixture: 149 μg/mL) to give a total volume of 500 μL, and the reaction mixture was incubated on an orbital shaker at 35 °C and 650 rpm in vertical position. Samples (50 μL) were taken 5, 10, 15, 20, 30, 40, 50, and 60 min after starting the reaction and immediately quenched by the addition of MeOH (50 μL). The samples were centrifuged (14,680 rpm, 20 min), and the supernatants were transferred to plastic vials for HPLC analysis.

The obtained time-point data were processed as described above (→ *Determination of specific activities with benzaldehyde*) to calculate the specific enzyme activity.

Determination of specific activities with secologanin (4c): A stock solution of tryptamine (**1**) in aqueous MOPS buffer (25 µL; 50 mM MOPS, 20 mM **1**·HCl, pH 7.0; final conc. of **1** in reaction mixture: 1 mM) was mixed with a stock solution of secologanin (**4c**) in MOPS buffer (100 µL; 50 mM MOPS, 10 mM **4c**, pH 7.0; final conc. of **4c** in reaction mixture: 2 mM). The reaction was started by adding a solution of purified STR (375 µL; conc. of STR: 0.67 µg/mL, final conc. of STR in reaction mixture: 0.5 µg/mL) to give a total volume of 500 µL, and the reaction mixture was incubated on an orbital shaker at 35 °C and 650 rpm in vertical position. Samples (50 µL) were taken 5, 10, 15, 20, 25, and 30 min after starting the reaction and immediately quenched by the addition of MeOH (150 µL). The samples were centrifuged (14,680 rpm, 10 min), and the supernatants were transferred to plastic vials for HPLC analysis.

The obtained time-point data were processed as described above (→ *Determination of specific activities with benzaldehyde*) to calculate the specific enzyme activity.

Preparative biotransformations (General procedure): In a screw-top Erlenmeyer flask (250 mL), lyophilised cells of *E. coli* SHuffle T7 Express lysY expressing *Rs*STR V176L/V208A (6.25 g) were resuspended in aqueous MOPS–tryptamine·HCl buffer (100 mM MOPS, 40 mM tryptamine·HCl, pH 6.2; 125 mL), and aldehyde (6.25 mmol; final conc. in reaction mixture: 50 mM) was added. The reaction mixture was incubated for 48 h in an orbital shaker at 35 °C and 650 rpm. At this time, a sample (500 µL) was taken, quenched by addition of MeOH (500 µL), and processed as described above (biotransformations on analytical scale) to determine conversion. The biotransformation was stopped by addition of saturated aqueous Na_2CO_3 solution (25 mL), and the reaction mixture was transferred to plastic tubes (4 × 50 mL) and centrifuged (4,000 rpm, 20 min) to remove the cell mass. The supernatant was mixed with brine (30 mL) and extracted with MTBE (4 × 50 mL), whereby phase separation was enhanced by filtering the aqueous–organic mixture through a Celite pad (2 cm) before each extraction step. The combined organic phases were washed with brine (30 mL), dried over Na_2SO_4, filtered, and concentrated under reduced pressure to obtain the crude product. The tetrahydro-β-carbolines were purified by column chromatography on silica gel 60 (340 mm × 30 mm). In the case of **3a** and **3b**, the column was packed in MTBE, eluted initially with MTBE (200–500 mL) to wash off non-basic impurities, and finally eluted with MTBE/MeOH/NH_3(aq.) = 96:3:1 (500 mL) to obtain the tetrahydro-β-carboline. In the case of **3h** and **3i**, TLC analysis revealed a substantial R_f value in MTBE, possibly due to an intramolecular hydrogen bond between the halogen substituent and the carboline NH group, which reduces the propensity for intermolecular hydrogen bonding interactions. A less polar eluent was therefore necessary, and the column was packed and eluted with cyclohexane/EtOAc = 2:1.

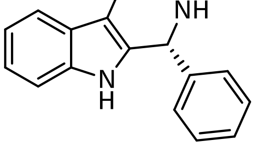

(*R*)-1-Phenyl-2,3,4,9-tetrahydro-1*H*-pyrido[3,4-*b*]indole (3a). Colourless, solid foam, 187 mg (15%). R_f = 0.82 (silica gel 60, MTBE/MeOH/NH_3(aq.) = 90:9:1, UV). m.p. 192–193 °C (lit.[14] 167 °C). $[\alpha]_D^{20}$ = +14.2 (*c* 0.62, EtOH); ref.[15] $[\alpha]_D^{21}$ = +10.6 (*c* 0.59, EtOH). ^{1}H NMR (300 MHz, $CDCl_3$): δ [ppm] = 7.63 (br s, 1H), 7.58–7.53 (m, 1H), 7.38–7.28 (m, 5H), 7.23–7.09 (m, 3H), 5.14 (t, *J* = 2.0 Hz, 1H), 3.37 (ddd, *J* = 12.5, 5.3, 3.8 Hz, 1H), 3.14 (ddd, *J* = 12.5, 8.8, 4.9 Hz, 1H), 2.93 (dddd, 1H, *J* = 14.0, 8.7, 5.3, 2.0 Hz, 1H), 2.82 (dddd, *J* = 15.4, 5.1, 3.7, 1.8 Hz, 1H). ^{13}C NMR (75 MHz, $CDCl_3$): δ [ppm] = 141.9, 136.0, 134.6, 129.0, 128.6, 128.3, 127.5, 121.8, 119.5, 118.4, 111.0, 110.4, 58.3, 43.0, 22.7. GC–MS (EI, 70 eV): *m/z* (%): 248 (90) $[M]^+$, 218 (100), 171 (40), 115 (9), 109 (11). HRMS (ESI): *m/z* calcd for $C_{17}H_{16}N_2+H^+$: 249.1386 $[M+H]^+$; found: 249.1390.

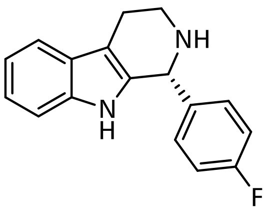

(*R*)-1-(4-Fluorophenyl)-2,3,4,9-tetrahydro-1*H*-pyrido-[3,4-*b*]indole (3b). Off-white solid, 51 mg (3.8%). R_f = 0.84 (silica gel 60, MTBE/MeOH/NH_3(aq.) = 90:9:1, UV). m.p. 179–181 °C. $[\alpha]_D^{20}$ = –9.6 (*c* 1.0, $CHCl_3$); ref.[16] $[\alpha]_D^{28}$ = +4.3 (*c* 2.0, $CHCl_3$) for (*S*)-enantiomer. ^{1}H NMR (300 MHz, $CDCl_3$): δ [ppm] = 7.58 (s, 1H), 7.49–7.41 (m, 1H), 7.21–6.99 (m, 5H), 6.94 (t, *J* = 8.6 Hz, 2H), 5.06 (t, *J* = 1.9 Hz, 1H), 3.25 (dt, *J* = 12.5, 4.6 Hz, 1H), 3.04 (ddd, *J* = 12.8, 8.4, 5.0 Hz, 1H), 2.90–2.67 (m, 2H). ^{13}C NMR (75 MHz, $CDCl_3$): δ [ppm] = 162.6 (d, J_{CF} = 246.9 Hz), 137.4 (d, J_{CF} = 3.2 Hz), 135.9, 133.9, 130.2 (d, J_{CF} = 8.1 Hz), 127.3, 121.9, 119.5, 118.3, 115.7 (d, J_{CF} = 21.4 Hz), 110.9, 110.4, 57.2, 42.6, 22.4. GC–MS (EI, 70 eV): *m/z* (%): 266 (90) $[M]^+$, 236 (100), 171 (28), 118 (13). HRMS (ESI): *m/z* calcd for $C_{17}H_{15}FN_2+H^+$: 267.1292 $[M+H]^+$; found: 267.1292.

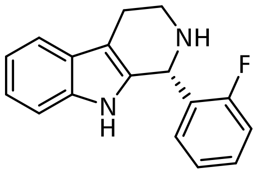

(*R*)-1-(2-Fluorophenyl)-2,3,4,9-tetrahydro-1*H*-pyrido-[3,4-*b*]indole (3h). Colourless, solid foam, 319 mg (24%). R_f = 0.20 (silica gel 60, cyclohexane/EtOAc = 2:1, UV). $[\alpha]_D^{20}$ = +1.6 (*c* 1.0, EtOH). m.p. 179–180 °C. ^{1}H-NMR (300 MHz, $CDCl_3$): δ [ppm] = 7.30 (dddd, *J* = 8.2, 7.2, 5.3, 2.0 Hz, 1H), 7.25–7.18 (m, 1H), 7.19–7.10 (m, 3H), 7.06 (td, *J* = 7.4, 1.2 Hz, 1H), 5.57 (t, *J* = 1.7 Hz, 1H), 3.31 (dt, *J* = 12.6, 5.2 Hz, 1H), 3.15 (ddd, *J* = 12.6, 7.2, 5.2 Hz, 1H), 2.97–2.86 (m, 1H), 2.83 (dtd, *J* = 15.4, 5.3, 1.7 Hz, 1H). ^{13}C-NMR (75 MHz, $CDCl_3$): δ [ppm] = 161.1 (d, J_{CF} = 246.7 Hz), 136.0, 133.2, 130.0 (d, J_{CF} = 4.0 Hz), 129.7 (d, J_{CF} = 8.4 Hz), 128.9 (d, J_{CF} = 13.4 Hz), 127.4, 124.5 (d, J_{CF} = 3.6 Hz), 121.9, 119.6, 118.3, 115.8 (d, J_{CF} = 21.9 Hz), 111.0, 110.9, 50.5 (d, J_{CF} = 3.6 Hz), 42.2, 22.6. GC–MS (EI, 70 eV): *m/z* (%): 266 (83) $[M]^+$, 236 (100), 217 (17), 171 (31), 118 (11). HRMS (ESI): *m/z* calcd for $C_{17}H_{15}FN_2+H^+$: 267.1292 $[M+H]^+$; found: 267.1296.

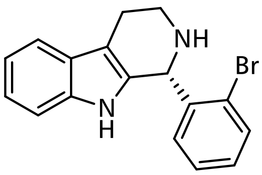

(*R*)-1-(2-Bromophenyl)-2,3,4,9-tetrahydro-1*H*-pyrido-[3,4-*b*]indole (3i). Colourless, solid foam, 508 mg (31%). R_f = 0.37 (silica gel 60, cyclohexane/EtOAc = 2:1, UV). $[\alpha]_D^{20}$ = –20.0 (*c* 1.0, MeOH); ref.[17] $[\alpha]_D^{25}$ = –0.17 (*c* 0.5, MeOH). m.p. 165–166 °C. ^{1}H-NMR (300 MHz, $CDCl_3$): δ [ppm] = 7.74 (s, 1H), 7.57–7.50 (m, 1H), 7.49–7.41 (m, 1H), 7.13–6.98 (m, 5H), 6.98–6.90 (m, 1H), 5.52 (t, *J* = 1.6 Hz, 1H), 3.11 (dt, *J* = 11.6, 5.5 Hz, 1H), 3.00 (dt, *J* = 12.2, 5.8 Hz, 1H), 2.86–2.66 (m, 2H). ^{13}C-NMR (75 MHz, $CDCl_3$): δ [ppm] = 140.8, 135.9, 133.2, 133.1, 130.5, 129.5, 127.7, 127.2, 124.3, 121.8, 119.4, 118.3, 110.9, 110.9, 56.2, 41.5, 22.4. GC–MS (EI, 70 eV): *m/z* (%): 328 (71) $[M]^+$ (^{81}Br), 326 (73) $[M]^+$ (^{79}Br), 297 (23), 243 (9), 218 (100), 171 (68), 123 (16), 109 (22). HRMS (ESI): *m/z* calcd for $C_{17}H_{15}BrN_2+H^+$: 327.0491 $[M+H]^+$ (^{79}Br); found: 327.0493.

Structural Bioinformatics and Computational Model Building

Different binding mode hypotheses of the product **3a** were evaluated computationally to aid in the selection of amino acids for mutagenesis and in the analysis and interpretation of the experimental results. To this end, all available X-ray complex structures of strictosidine synthase with ligands containing an indole substructure (PDB: 2fpb, 2v91, 2vaq, 3v1s, 4imb, 4iyg, 6s5q) were overlaid onto the reference structure from *Rauvolfia serpentina* (PDB: 2v91) using the program MOE.[18] A low-energy molecular model of **3a** in the desired (*R*)-configuration was built in MOE and its indole substructure superimposed onto the corresponding substructure of the STR crystal structure ligands. The ligand models were transferred to the protein structure 2v91 and the resulting complexes minimized with fixed protein coordinates, apart from selected residues (Y151, V176, M276, L323) that showed mobility in the structure overlay and were hence treated as flexible. Finally, the relaxed complexes were prioritised according to structural fit and ligand strain.

Two binding mode hypotheses stood out and were further explored. First, a model with an inverted binding mode (Figure S9, A) as recently identified in the investigation of non-natural STR substrates, exemplified by crystal structure 6s5q.[10] Second, a binding mode model in which the 1,2,3,4-tetrahydro-β-carboline core overlays well with the natural substrate in the crystal structure 2v91(Figure S9, B). Protein models for the two activating mutations V176L and V208A were prepared using the Protein Design feature of MOE. The first, inverted binding mode model is able to better rationalise the observed structure–activity relationship, for example the tolerance for *meta*-phenyl substitutions, and was hence used as the basis for Figures 2 and 3 in the main article. This model is also provided as a separate Supporting Information file (in PDB format).

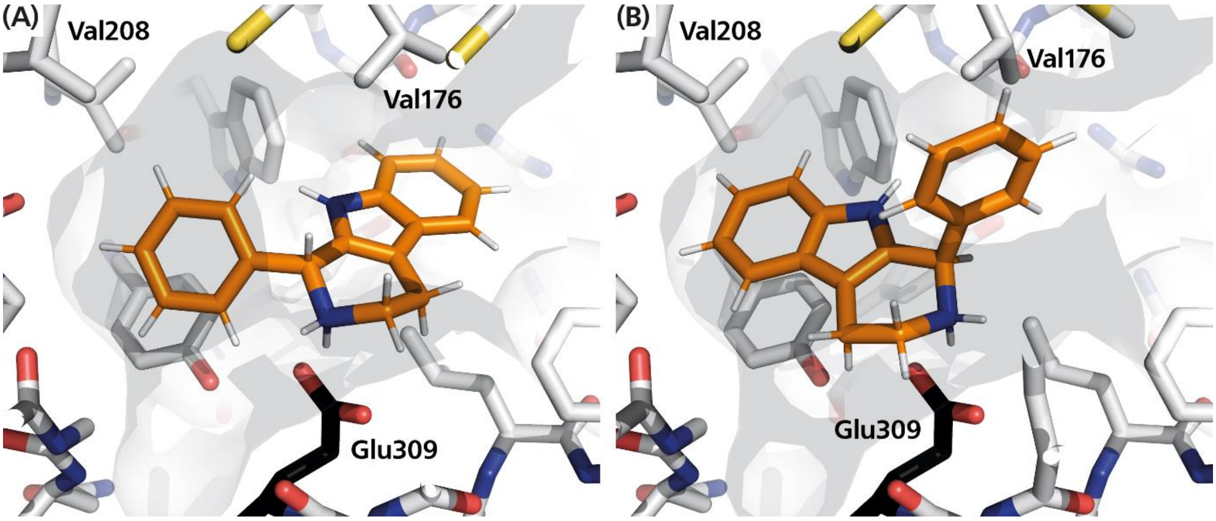

Figure S9. Structural hypotheses for the binding of tetrahydro-β-carboline (*R*)-**3a** in the active site of wild-type *Rs*STR. **(A)** 'Inverted' binding mode analogous to the complex of *OpSTR* with (*S*)-1-isobutyl-2,3,4,9-tetrahydro-1*H*-pyrido-[3,4-*b*]indole (PDB: 6s5q). **(B)** 'Natural' binding mode analogous to the complex of *RsSTR* with (*S*)-strictosidine (PDB: 2v91). The catalytically essential active-site residue Glu309 is shown in black.

Analytical Methods

Gas chromatography (achiral stationary phase):

Achiral-phase GC analyses were carried out on an *Agilent* 7890A GC system equipped with a flame-ionisation detector (FID) and an *Agilent J&W* HP-5 capillary column (dimensions: 30 m × 0.32 mm × 0.25 μm; stationary phase: bonded & cross-linked 5%-phenyl-methylpoly-siloxane), using helium (2 mL/min flow rate) as carrier gas. Compounds were quantified based on calibration curves that were set up using *n*-dodecane (1 mM in EtOAc used for extraction) as internal standard.

Method GC-A1 (compounds **1**, **4a**, **5a**)

Column:	HP-5 (30 m × 0.32 mm × 0.25 μm)
Carrier gas:	Helium
Flow rate:	2 mL/min
Injector temperature:	300 °C
Detector temperature:	300 °C
Split ratio:	15:1
Oven temperature program:	100 °C, 0.5 min; 20 °C/min to 210 °C; 210 °C, 3 min; 20 °C/min to 300 °C; 300 °C, 2 min
Total run time:	15.5 min

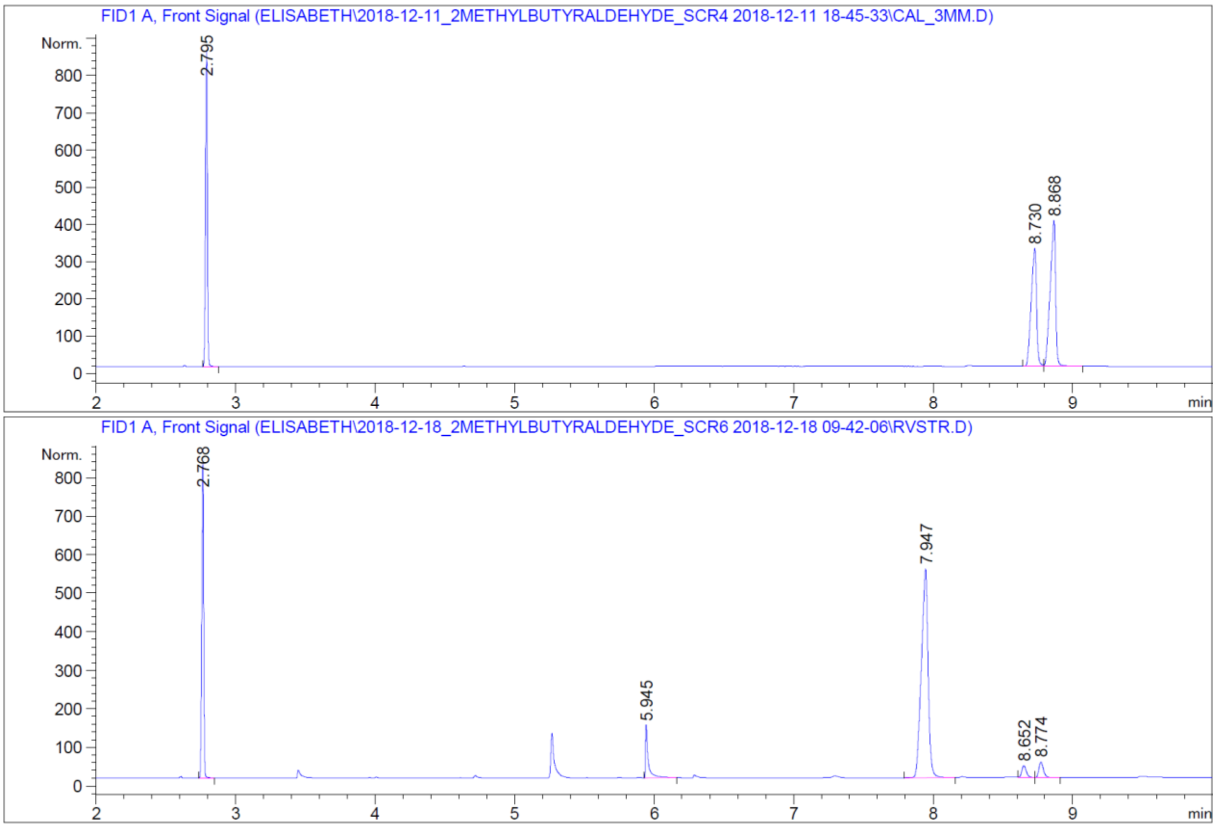

Figure S10. Chromatographic separation of *n*-dodecane (t_r 2.8 min), 2-methylbutyraldehyde (**4a**; t_r 5.9 min), tryptamine (**1**; t_r 7.9 min) and tetrahydro-β-carboline **5a** (diastereomers; t_r 8.7 min, 8.9 min) using GC method GC-A1. Product reference (**5a**; upper), biotransformation using *Rs*STR V208A (lower).

Gas chromatography with mass-selective detection (achiral stationary phase): GC–MS analyses were carried out on an *Agilent* 7890A GC system equipped with an *Agilent J&W* HP-5ms capillary column (dimensions: 30 m × 0.25 mm × 0.25 µm; stationary phase: bonded & cross-linked 5%-phenyl-methylpolysiloxane) and coupled to an Agilent 5975C mass-selective detector (electron impact ionisation, 70 eV; quadrupole mass selection), using helium (1 mL/min flow rate) as carrier gas.

Hydrochlorides of tetrahydro-β-carbolines were converted into the corresponding free bases for analysis: A sample of the compound (2–3 mg) was suspended in saturated aqueous Na_2CO_3 solution (500 µL) and extracted with EtOAc (2 × 500 µL). The combined organic phases were dried over Na_2SO_4, centrifuged (14,680 rpm, 1 min), and the supernatant was transferred to a glass vial for GC–MS analysis.

**Method GC-MS1** (used for all compounds)	
Injector temperature:	300 °C
Split ratio:	90:1
Oven temperature program:	200 °C, 0.5 min; 5 °C/min to 300 °C; 300 °C, 2 min
Total run time:	22.5 min

Table S13. Retention times and mass spectra of compounds analysed by GC–MS.

Compound	t_r[a] [min]	Mass spectrum (*m/z*)
tryptamine (**1**)	3.2	160 (16) [*M*]$^+$, 130 (100), 103 (9), 77 (11)
**3a**	10.5	248 (91) [*M*]$^+$, 218 (100), 171 (39), 115 (8), 109 (11)
**3b**	10.4	266 (87) [*M*]$^+$, 236 (100), 171 (28), 118 (14)
**3c**	10.4	266 (88) [*M*]$^+$, 236 (100), 171 (26), 118 (10)
**3d**	13.3	284 (33) [*M*]$^+$ (^{37}Cl), 282 (100) [*M*]$^+$ (^{35}Cl), 253 (44), 218 (86), 171 (66), 109 (22)
**3e**	14.8	328 (64) [*M*]$^+$ (^{81}Br), 326 (72) [*M*]$^+$ (^{79}Br), 297 (26), 218 (100), 171 (86), 144 (13), 123 (12), 109 (24)
**3f**	13.5	278 (100) [*M*]$^+$, 249 (41), 218 (35), 204 (20), 171 (49)
**3g**	17.3	293 (100) [*M*]$^+$, 264 (42), 247 (33), 217 (85), 171 (71), 109 (17)
**3h**	10.3	266 (80) [*M*]$^+$, 236 (100), 217 (17), 171 (29), 118 (10)
**3i**	14.2	328 (68) [*M*]$^+$ (^{81}Br), 326 (75) [*M*]$^+$ (^{79}Br), 297 (22), 243 (15), 218 (100), 171 (65), 123 (17), 109 (20)
**3j**	13.6	284 (33) [*M*]$^+$ (^{37}Cl), 282 (100) [*M*]$^+$ (^{35}Cl), 253 (43), 218 (96), 171 (48), 109 (25)
**3k**	14.2	278 (M^+, 100), 249 (40), 218 (42), 204 (20), 171 (27)
**3l**	17.9	293 (M^+, 78), 264 (31), 244 (28), 217 (100), 171 (53), 143 (17), 109 (16)
**5a**	6.2, 6.3	228 (3) [*M*]$^+$, 171 (100), 154 (6), 144 (6)
**5b**	6.5	228 (9) [*M*]$^+$, 171 (100), 156 (8), 144 (6)

[a] Retention time. [b] The molecular ion and the most prominent fragments in the spectrum are listed. The relative intensities (%) are given in parentheses.

High-performance liquid chromatography (achiral stationary phase):

Achiral-phase HPLC analyses were carried out on a *Shimadzu* HPLC system (Communication Bus Module CBM-20 A, Column Oven CTO-20 AC, Degasser DGU-20 A5, Liquid Chromatograph LC-20 AD, Autosampler SIL-20 AC, Diode Array Detector SPD-M20 A) equipped with a *Phenomenex* Luna C18(2) column (dimensions: 250 mm × 4.6 mm; stationary phase: C18 with TMS endcapping; particle size 5 μm; pore size: 100 Å), using water and acetonitrile, each containing 0.1% (v/v) trifluoroacetic acid (TFA), as eluents.

Integration was performed on the absorbance signal at a wavelength of 280 nm and conversions were calculated from the peak areas of tryptamine (**1**) and tetrahydro-β-carboline **3a–l** using a correction factor calculated from the molar extinction coefficients (Table S14).

Method HPLC-A1 (Compounds **1**, **2a–l**, **3a–l**, **5a–c**)

Column:	Luna C18(2)
Column temperature:	30 °C
Eluent gradient:	H_2O/MeCN = 90:10 (+ 0.1% TFA), 2 min;<br>linear gradient to H_2O/MeCN = 40:60 (+ 0.1% TFA), 11 min;<br>linear gradient to 100% MeCN (+ 0.1% TFA), 1 min;<br>100% MeCN (+ 0.1% TFA), 4 min;<br>H_2O/MeCN = 90:10 (+ 0.1% TFA), 3 min
Eluent flow rate:	1 mL/min
Run time:	21 min
Integration wavelength:	280 nm

Table S14. Molar extinction coefficients (λ = 280 nm) of compounds analysed by HPLC.

Compound	ϵ (280 nm)[a] [M^{-1} cm^{-1}]	Comment
Tryptamine (**1**)	5700	taken from ref. [19]
Strictosidine (**5c**)	5140	taken from ref. [20]
**3a**	7618 (± 87)	determined experimentally
**3b**	7000 (± 154)	determined experimentally
**3c**	6946 (± 159)	determined experimentally
**3d**	7078 (± 142)	determined experimentally
**3e**	7675 (± 56)	determined experimentally
**3f**	10252 (± 311)	determined experimentally
**3g**	12488 (± 378)	determined experimentally
**3h**	7875 (± 47)	determined experimentally
**3i**	8156 (± 105)	determined experimentally
**5a**	7078 (± 46)	determined experimentally
**5b**	6794 (± 96)	determined experimentally

[a] Experimentally determined values are the means of three replicates each at two different concentrations (0.1 mM, 0.05 mM). Standard deviations are given in parentheses.

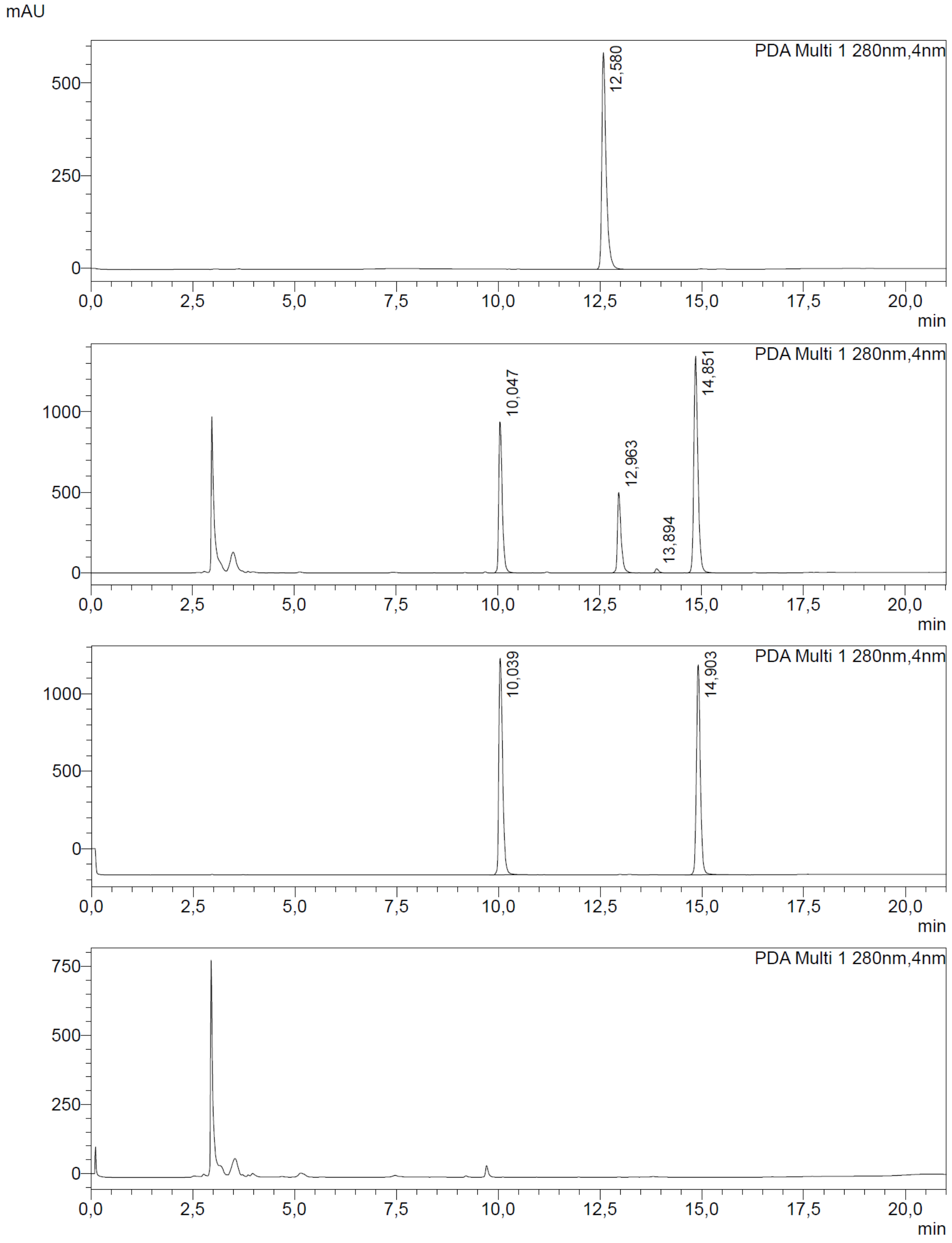

Figure S11. Chromatographic separation of tryptamine (**1**; t_r 10.0 min), tetrahydro-β-carboline **3a** (t_r 13.0 min) and benzaldehyde (**2a**; t_r 14.9 min) using HPLC method HPLC-A1. From top to bottom: Product reference (**3a**), biotransformation using *Rs*STR V176L/V208A, blank reaction without STR, blank reaction using *Rs*STR V176L/V208A without substrates. The peaks at t_r 2.5–4.0 min are present in all chromatograms of samples containing biocatalyst (independent of the presence or absence of substrates) and are hence ascribed to polar compounds liberated from the *E. coli* cells. The minor peak at t_r 13.9 min is ascribed to an oxidation product of tetrahydro-β-carboline **3a** that was also observed in GC–MS analysis (*m/z* 246).

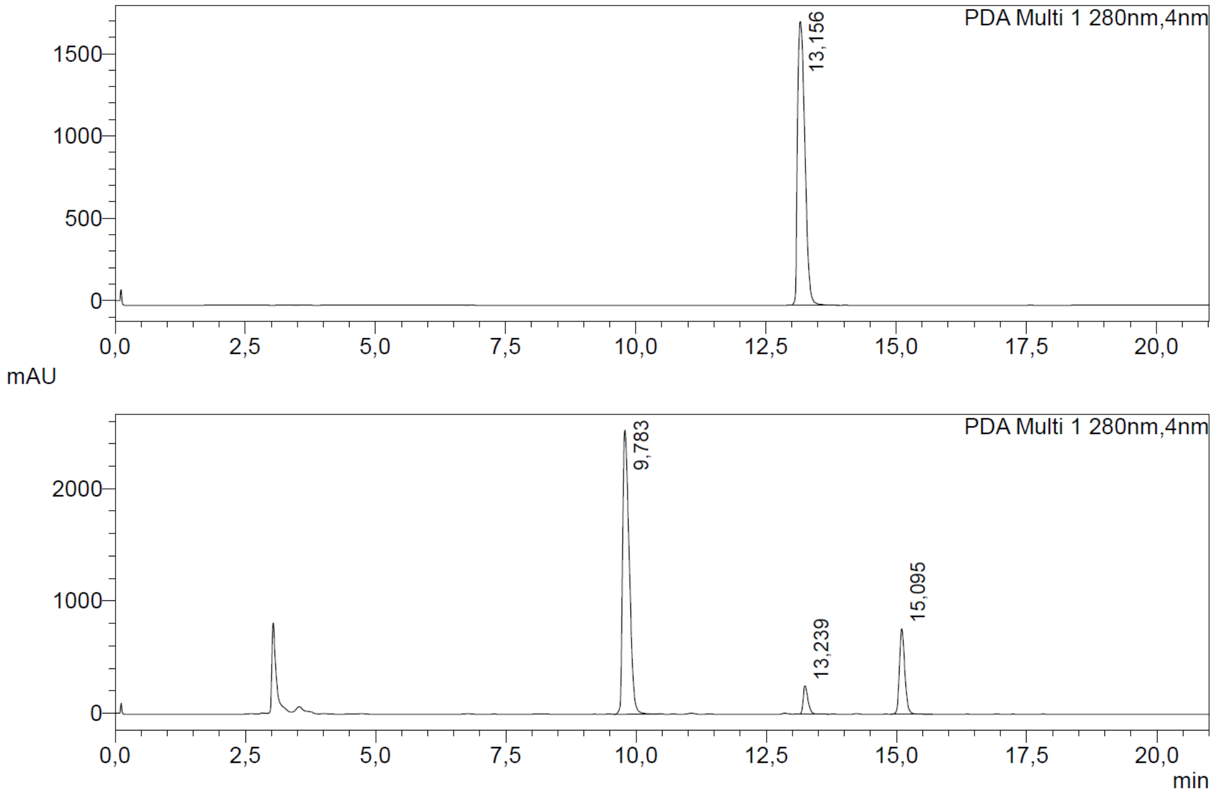

Figure S12. Chromatographic separation of tryptamine (**1**; t_r 9.8 min), tetrahydro-β-carboline **3b** (t_r 13.2 min) and *p*-fluorobenzaldehyde (**2b**; t_r 15.1 min) using HPLC method HPLC-A1. Product reference (**3b**; upper), biotransformation using *Rs*STR V176L/V208A (lower).

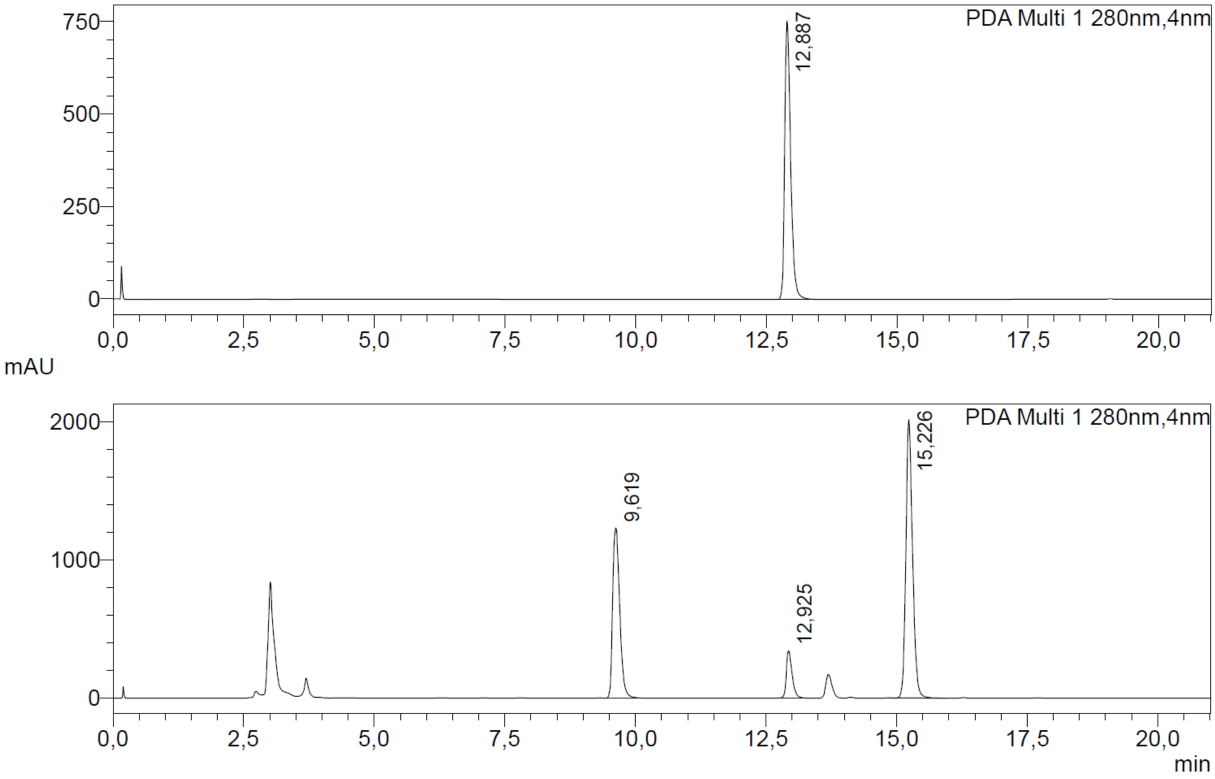

Figure S13. Chromatographic separation of tryptamine (**1**; t_r 9.6 min), tetrahydro-β-carboline **3c** (t_r 12.9 min) and *m*-fluorobenzaldehyde (**2c**; t_r 15.2 min) using HPLC method HPLC-A1. Product reference (**3c**; upper), biotransformation using *Rs*STR V176L/V208A (lower).

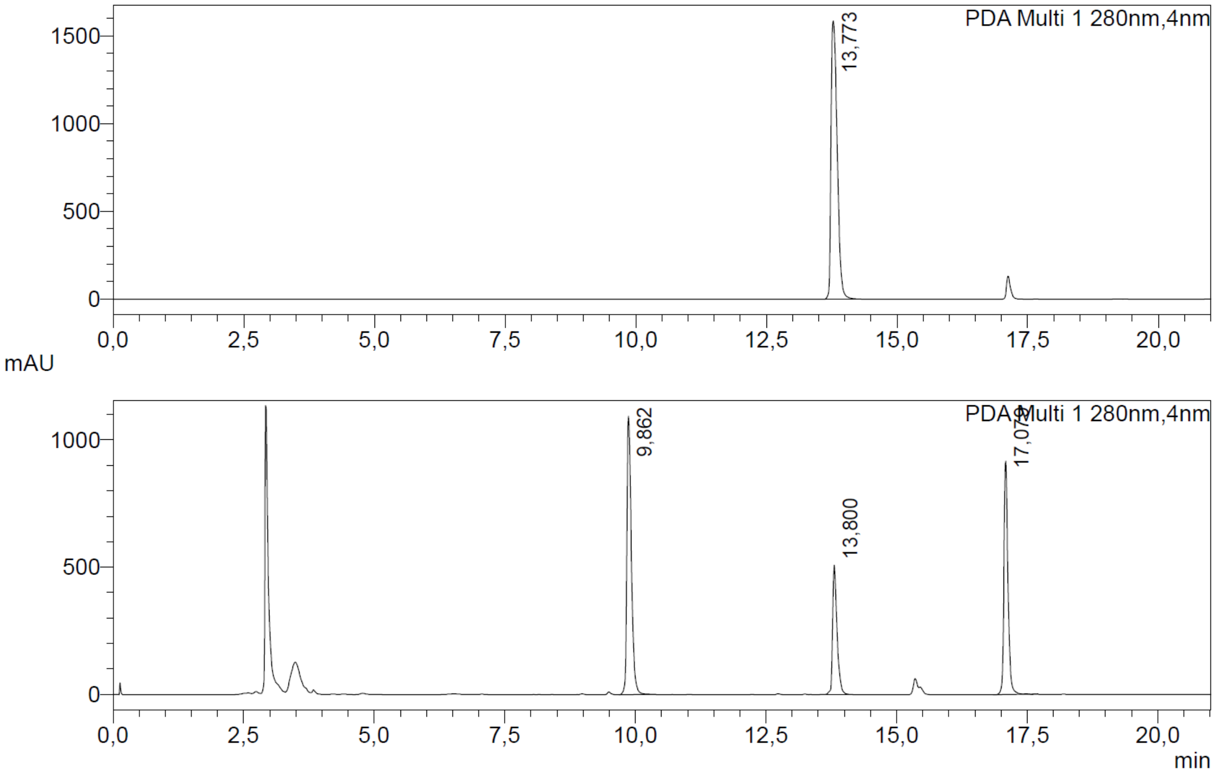

Figure S14. Chromatographic separation of tryptamine (**1**; t_r 9.9 min), tetrahydro-β-carboline **3d** (t_r 13.8 min) and *m*-chlorobenzaldehyde (**2d**; t_r 17.1 min) using HPLC method HPLC-A1. Product reference (**3d**; upper), biotransformation using *Rs*STR V176L/V208A (lower).

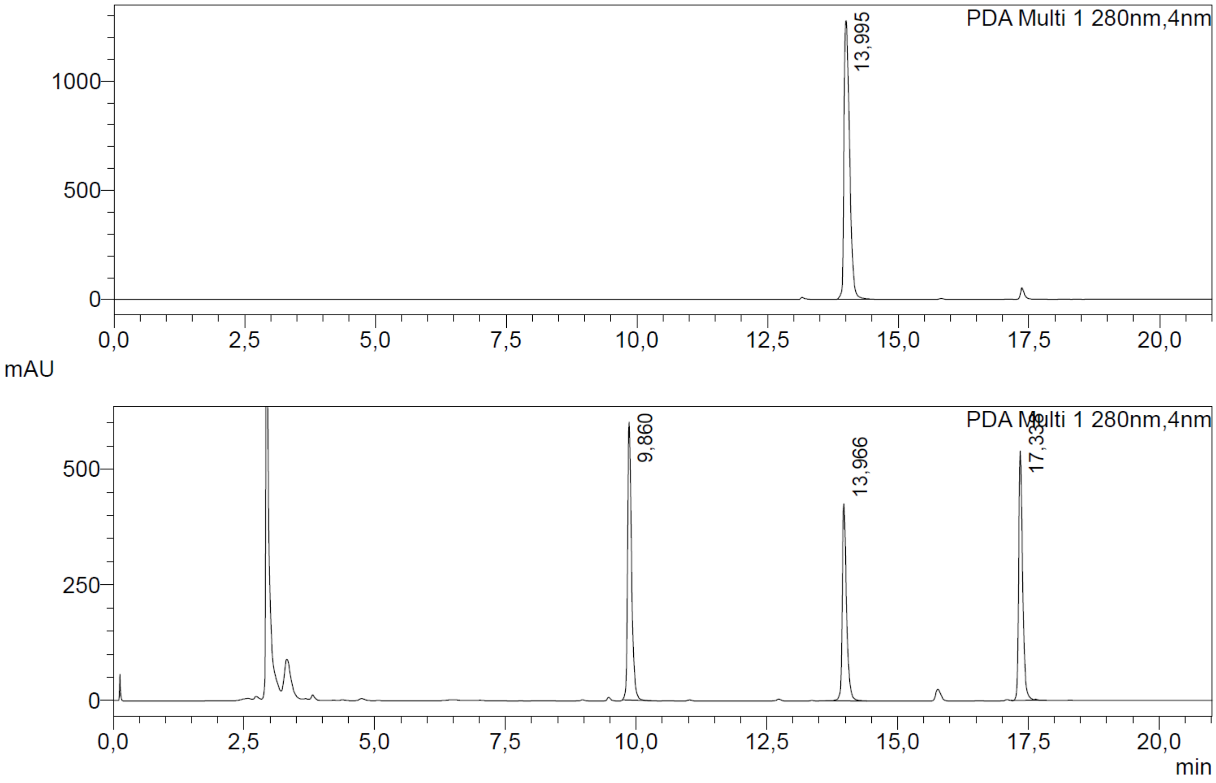

Figure S15. Chromatographic separation of tryptamine (**1**; t_r 9.9 min), tetrahydro-β-carboline **3e** (t_r 14.0 min) and *m*-bromobenzaldehyde (**2e**; t_r 17.3 min) using HPLC method HPLC-A1. Product reference (**3e**; upper), biotransformation using *Rs*STR V176L/V208A (lower).

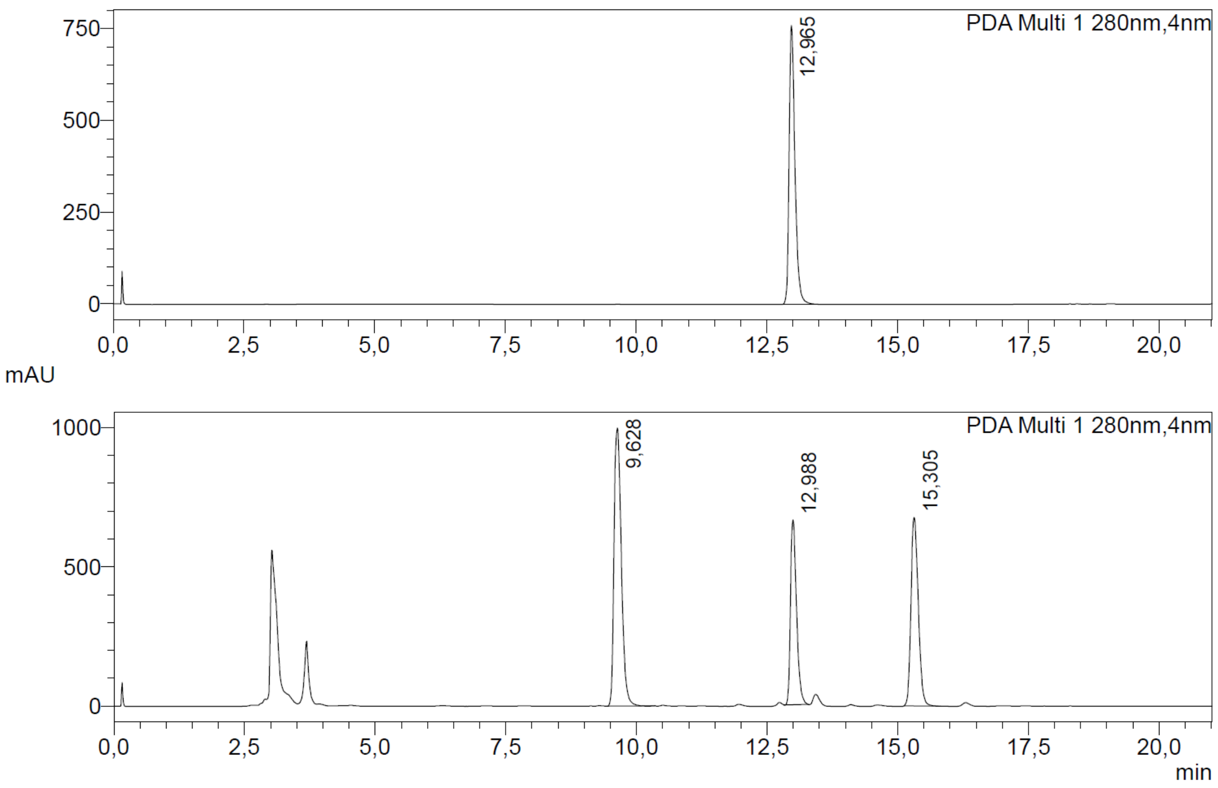

Figure S16. Chromatographic separation of tryptamine (**1**; t_r 9.6 min), tetrahydro-β-carboline **3f** (t_r 13.0 min) and *m*-anisaldehyde (**2f**; t_r 15.3 min) using HPLC method HPLC-A1. Product reference (**3f**; upper), biotransformation using *Rs*STR V176L/V208A (lower).

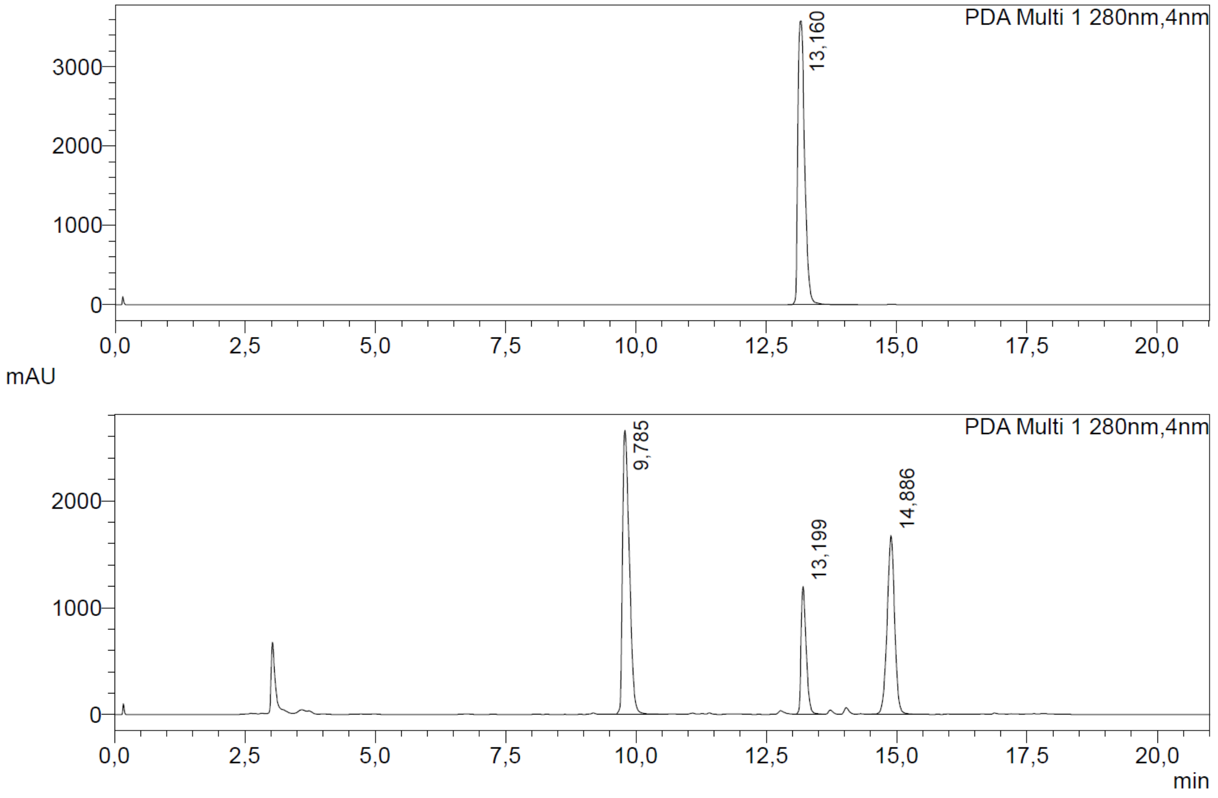

Figure S17. Chromatographic separation of tryptamine (**1**; t_r 9.8 min), tetrahydro-β-carboline **3g** (t_r 13.2 min) and *m*-nitrobenzaldehyde (**2g**; t_r 14.9 min) using HPLC method HPLC-A1. Product reference (**3g**; upper), biotransformation using *Rs*STR V176L/V208A (lower).

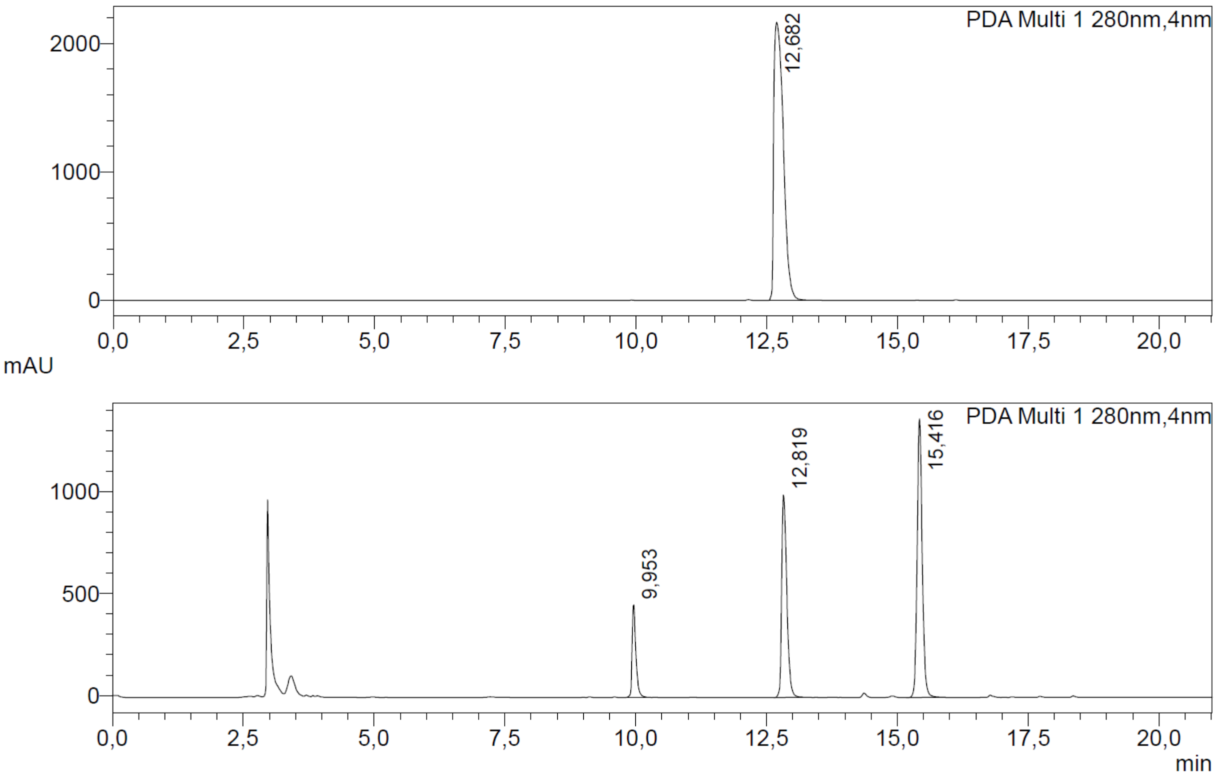

Figure S18. Chromatographic separation of tryptamine (**1**; t_r 10.0 min), tetrahydro-β-carboline **3h** (t_r 12.7 min) and *m*-anisaldehyde (**2h**; t_r 15.4 min) using HPLC method HPLC-A1. Product reference (**3h**; upper), biotransformation using *Rs*STR V176L/V208A (lower).

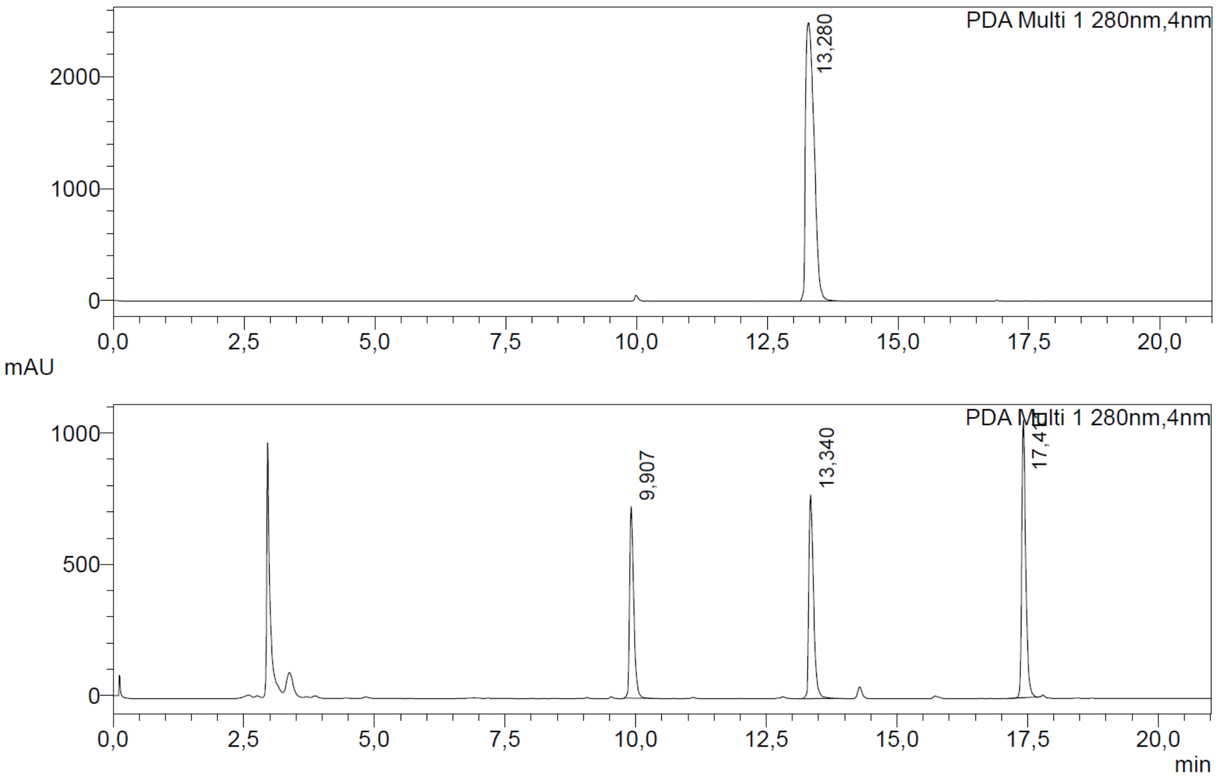

Figure S19. Chromatographic separation of tryptamine (**1**; t_r 9.9 min), tetrahydro-β-carboline **3i** (t_r 13.3 min) and *m*-nitrobenzaldehyde (**2i**; t_r 17.4 min) using HPLC method HPLC-A1. Product reference (**3i**; upper), biotransformation using *Rs*STR V176L/V208A (lower).

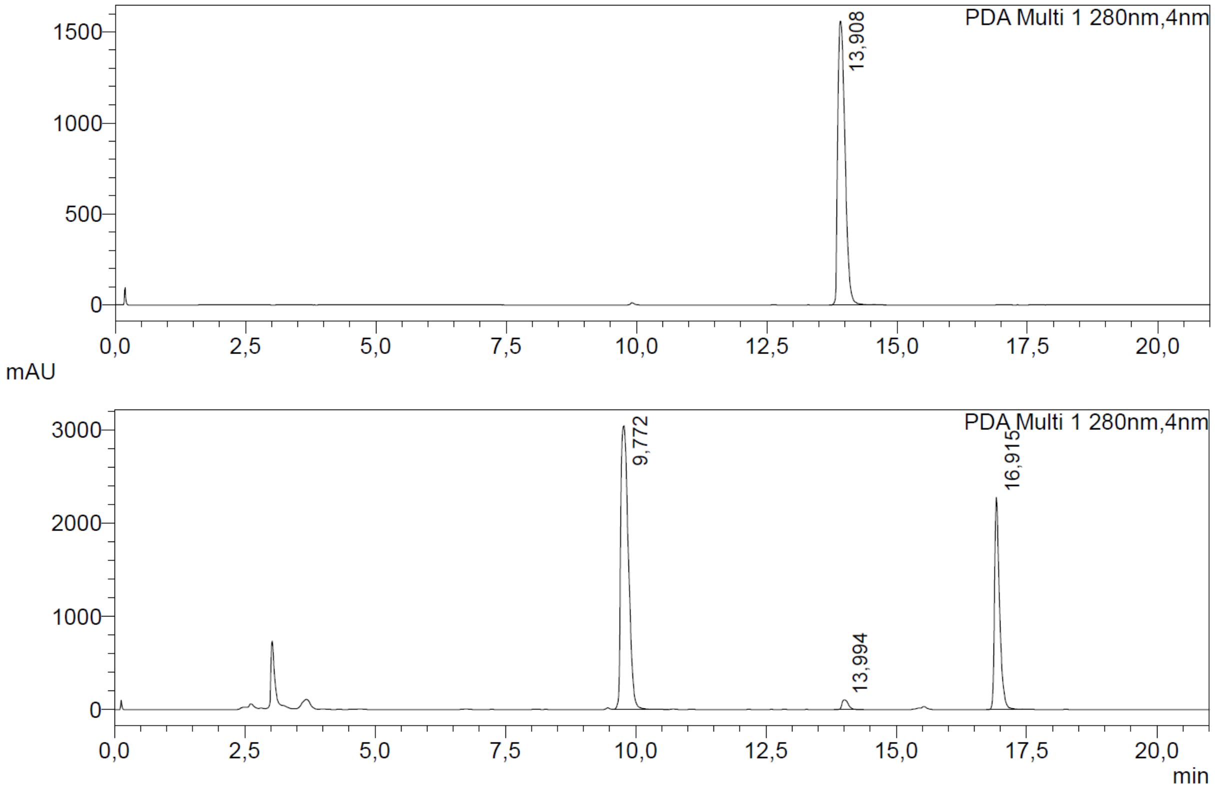

Figure S20. Chromatographic separation of tryptamine (**1**; t_r 9.8 min), tetrahydro-β-carboline **3j** (t_r 14.0 min) and *p*-chlorobenzaldehyde (**2j**; t_r 16.9 min) using HPLC method HPLC-A1. Product reference (**3j**; upper), biotransformation using *Rs*STR V176L/V208A (lower).

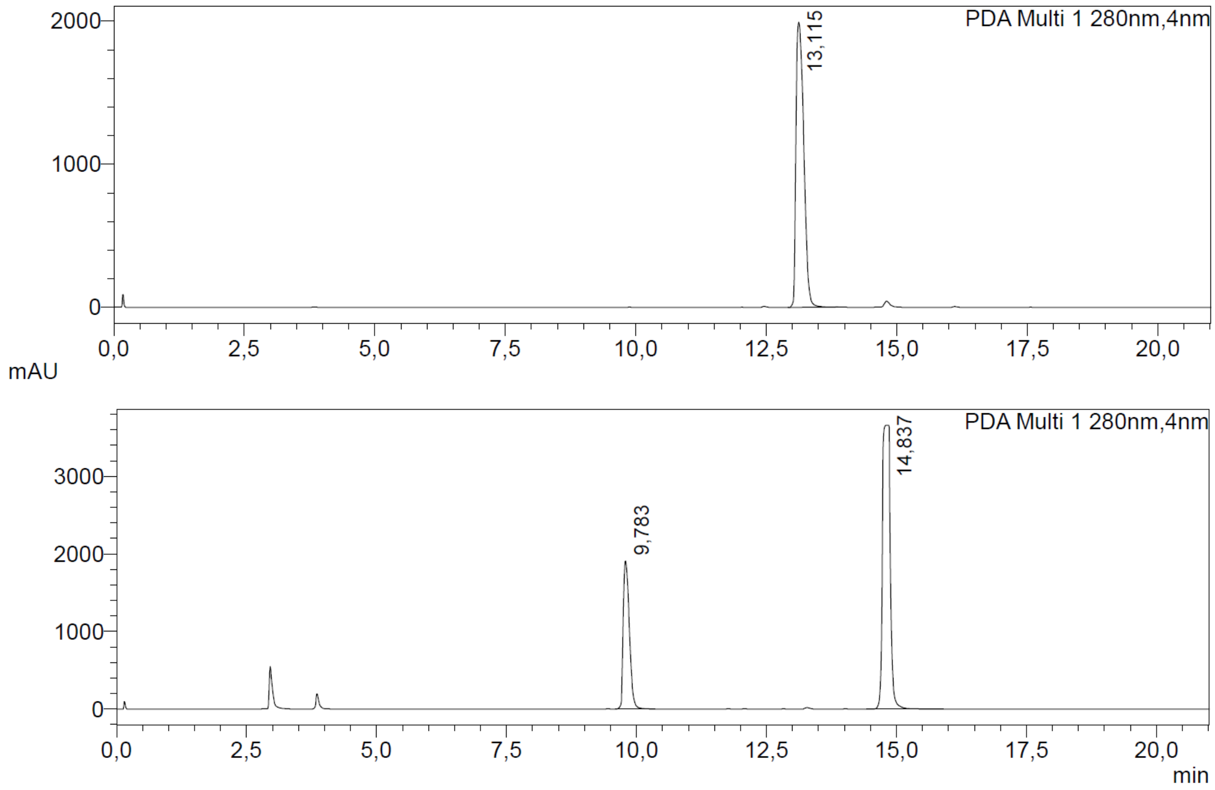

Figure S21. Chromatographic separation of tryptamine (**1**; t_r 9.8 min), tetrahydro-β-carboline **3k** (t_r 13.1 min) and *p*-anisaldehyde (**2k**; t_r 14.8 min) using HPLC method HPLC-A1. Product reference (**3k**; upper), biotransformation using *Rs*STR V176L/V208A (lower).

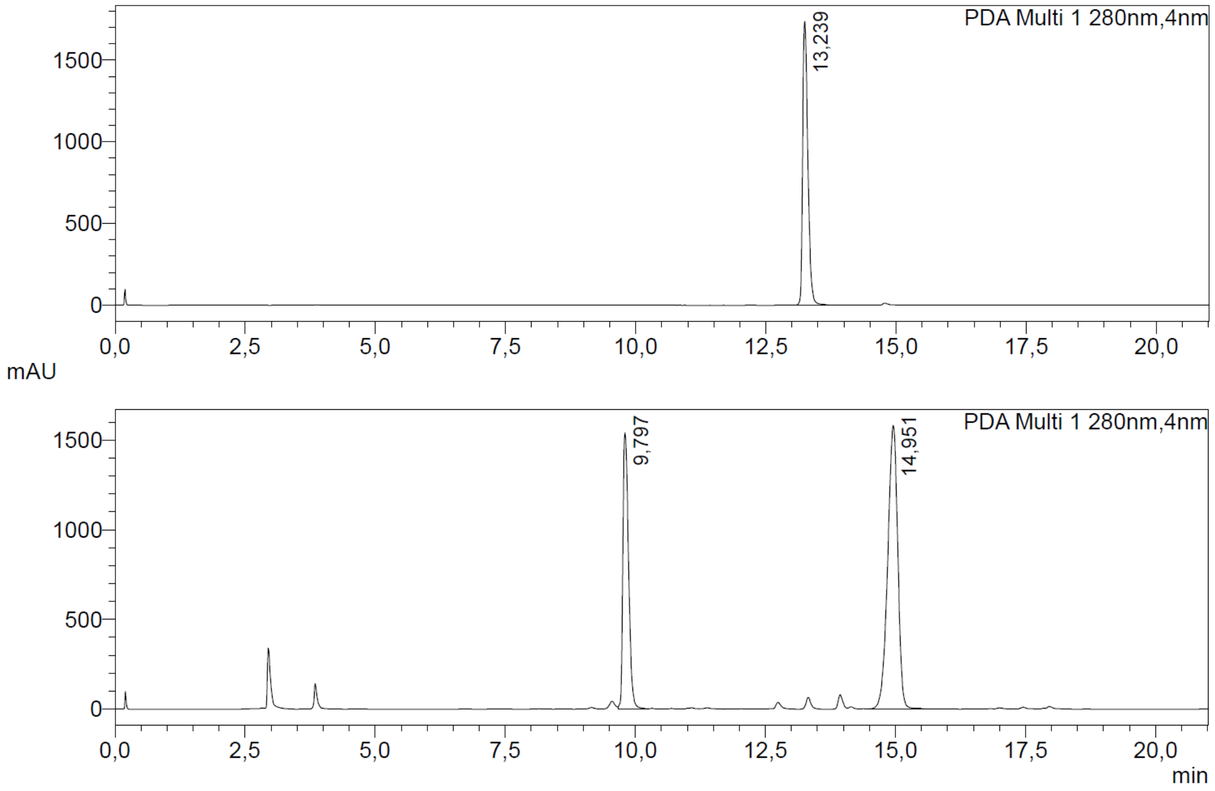

Figure S22. Chromatographic separation of tryptamine (**1**; t_r 9.8 min), tetrahydro-β-carboline **3l** (t_r 13.2 min) and *p*-nitrobenzaldehyde (**2l**; t_r 15.0 min) using HPLC method HPLC-A1. Product reference (**3l**; upper), biotransformation using *Rs*STR V176L/V208A (lower).

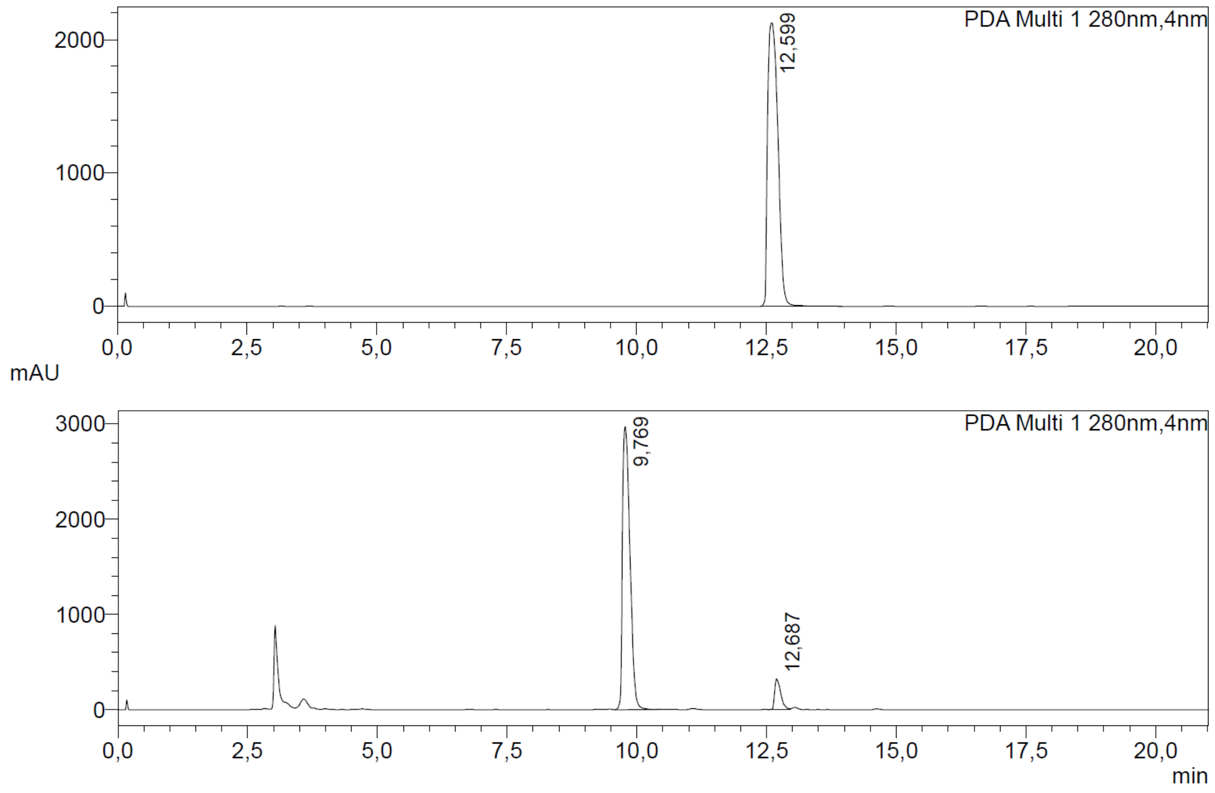

Figure S23. Chromatographic separation of tryptamine (**1**; t_r 9.8 min) and **5a** (t_r 12.7 min) using HPLC method HPLC-A1. Product reference (**5a**; upper), biotransformation using *Rs*STR V176L/V208A (lower).

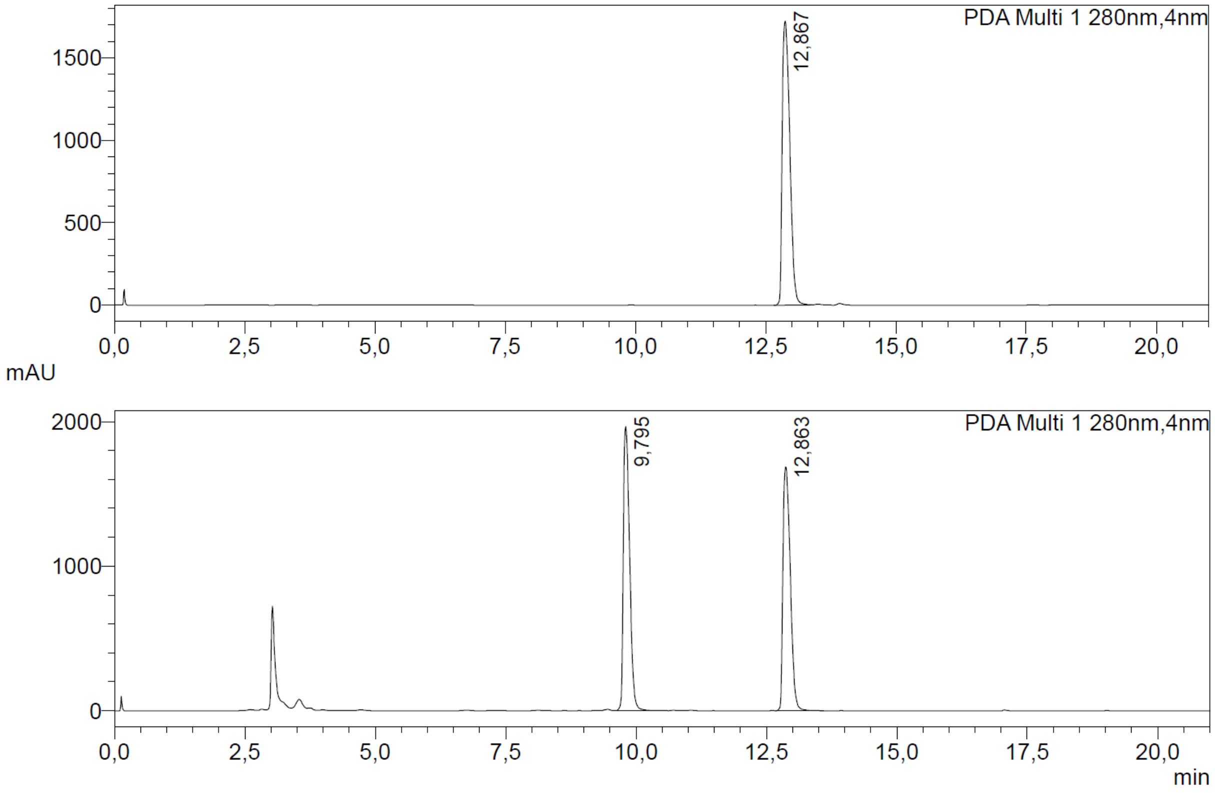

Figure S24. Chromatographic separation of tryptamine (**1**; t_r 9.8 min) and **5b** (t_r 12.9 min) using HPLC method HPLC-A1. Product reference (**5b**; upper), biotransformation using *Rs*STR V176L/V208A (lower).

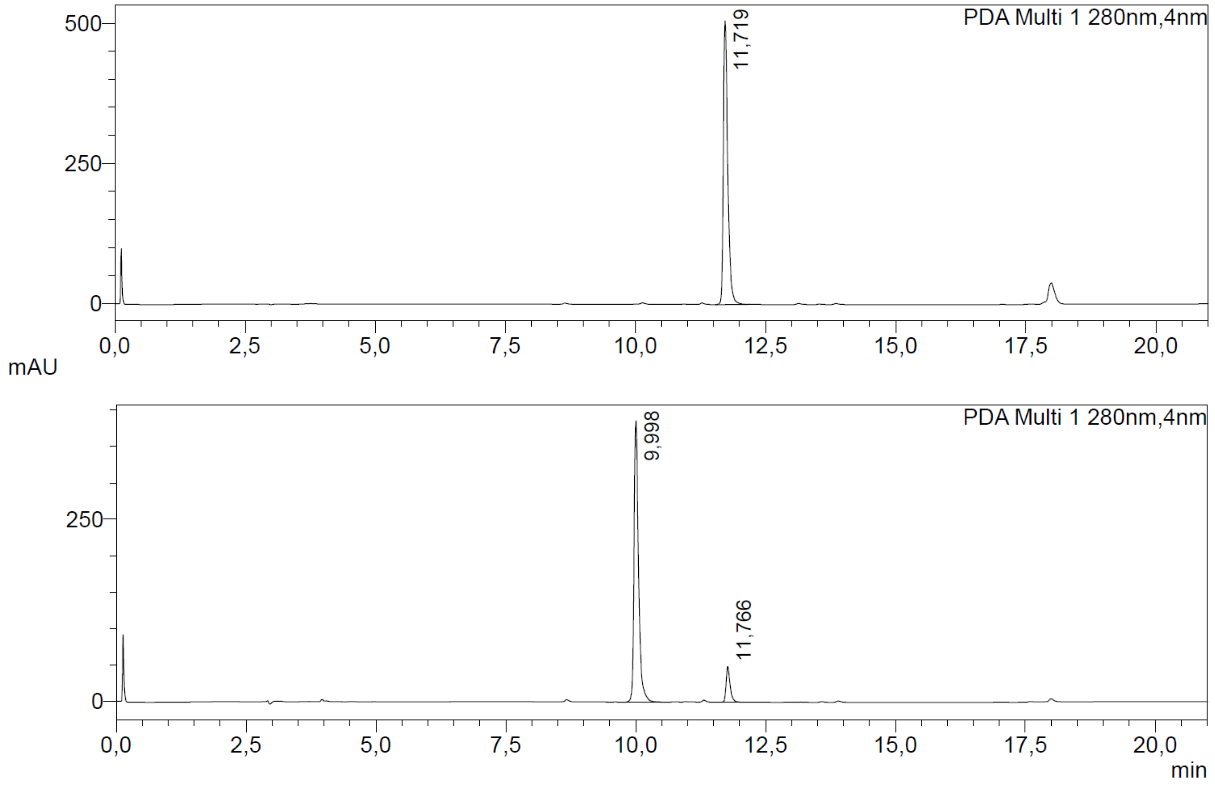

Figure S25. Chromatographic separation of tryptamine (**1**; t_r 10.0 min) and strictosidine (**5c**; t_r 11.8 min) using HPLC method HPLC-A1. Product reference (**5c**; upper), biotransformation using *Rs*STR wild type (lower).

High-performance liquid chromatography (chiral stationary phase):
Sample preparation: Biotransformations (500 µL) were quenched by addition of 10M NaOH solution (100 µL) and extracted with EtOAc (2 × 500 µL). The combined organic phases were dried over Na_2SO_4, centrifuged, and the supernatants were transferred to fresh vials and evaporated under a stream of air. Since co-elution problems were encountered when the samples were analysed directly, the THBC products were purified from the crude samples prior to analysis. To this end, the dried material was dissolved in EtOAc (200 µL) and subjected to small-scale column chromatography (approx. 6 × 50 mm silica gel 60 in a Pasteur pipette, column packed in MTBE, elution with MTBE (2 mL) and MTBE/MeOH/NH_3(aq.) = 95:4:1 (4 mL), collection of 1 mL fractions). The THBC-containing fractions were evaporated under a stream of air, re-dissolved in HPLC-grade MeOH (200 µL) and subjected to HPLC analysis.

Chiral-phase HPLC analyses were carried out on a *Shimadzu* HPLC system (Communication Bus Module CBM-20 A, Column Oven CTO-20 AC, Degasser DGU-20 A5, Liquid Chromatograph LC-20 AD, Autosampler SIL-20 AC, Diode Array Detector SPD-M20 A) equipped with a *Daicel* Chiralpak AZ-3 column (dimensions: 250 mm × 4.6 mm, stationary phase: amylose-tris-(3-chloro-4-methylphenylcarbamate) coated on silica gel, particle size: 3 µm) or a *Daicel* Chiralcel OD-H column (dimensions: 250 mm × 4.6 mm, stationary phase: cellulose-tris-(3,5-dimethyl-phenylcarbamate coated on silica gel, particle size: 5 µm), using *n*-heptane and 2-propanol, each containing 0.1% (v/v) diethylamine (DEA), as eluents.

**Method HPLC-C1** (compounds **3a–e, 3h, 3i**)	
Column:	Chiralpak AZ-3
Column temperature:	30 °C
Eluent:	*n*-heptane/2-propanol = 90:10 (+ 0.1% DEA)
Eluent flow rate:	1 mL/min
Run time:	30 min
Integration wavelength:	280 nm

**Method HPLC-C2** (compound **3g**)	
Column:	Chiralpak AZ-3
Column temperature:	30 °C
Eluent:	*n*-heptane/2-propanol = 85:15 (+ 0.1% DEA)
Eluent flow rate:	1 mL/min
Run time:	30 min
Integration wavelength:	280 nm

**Method HPLC-C3** (compound **3f**)	
Column:	Chiralpak AZ-3
Column temperature:	30 °C
Eluent:	*n*-heptane/2-propanol = 80:20 (+ 0.1% DEA)
Eluent flow rate:	1 mL/min
Run time:	30 min
Integration wavelength:	280 nm

**Method HPLC-C4** (compounds **3a, 3b**)	
Column:	Chiralcel OD-H
Column temperature:	30 °C
Eluent:	*n*-heptane/2-propanol = 80:20 (+ 0.1% DEA)
Eluent flow rate:	1 mL/min
Run time:	30 min
Integration wavelength:	280 nm

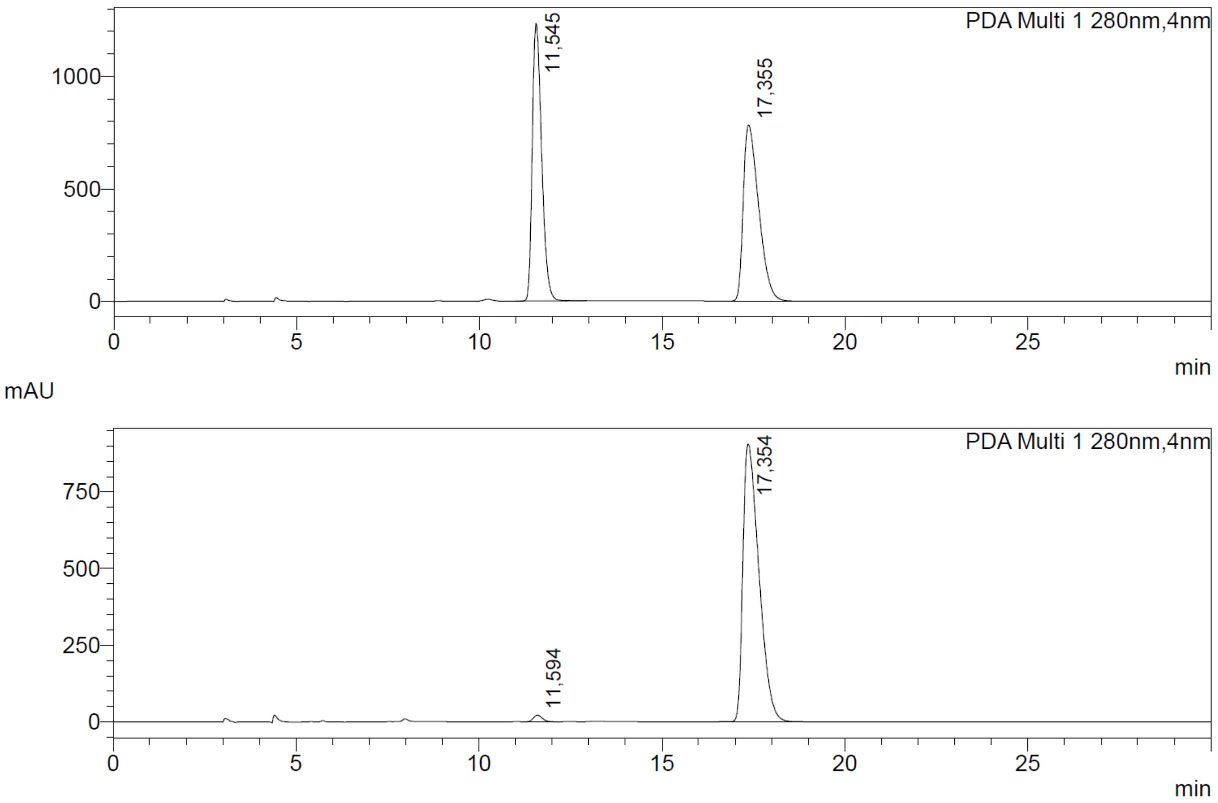

Figure S26. Chromatographic separation of the enantiomers of tetrahydro-β-carboline **3a** using HPLC method HPLC-C1. Racemic standard (upper), biotransformation using *Rs*STR V176L/V208A (lower).

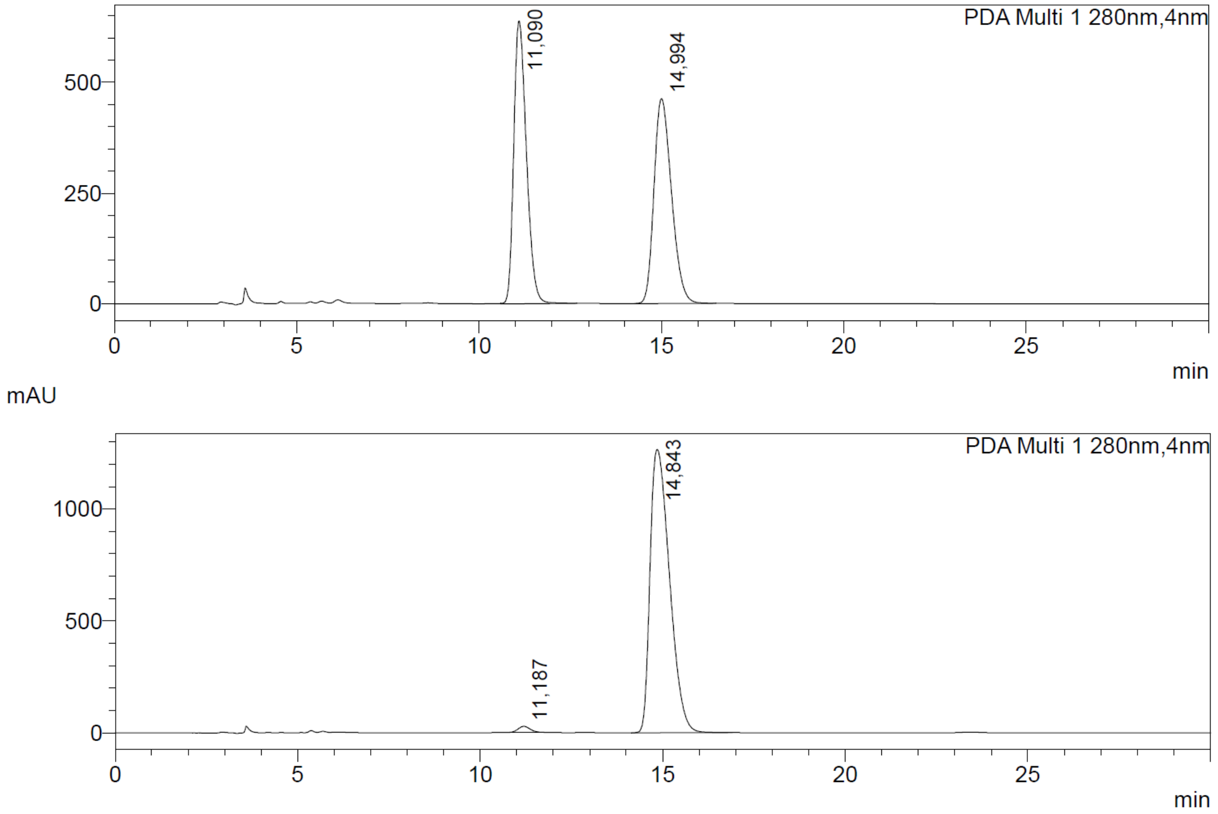

Figure S27. Chromatographic separation of the enantiomers of tetrahydro-β-carboline **3a** using HPLC method HPLC-C4. Racemic standard (upper), biotransformation using *Rs*STR V176L/V208A (lower).

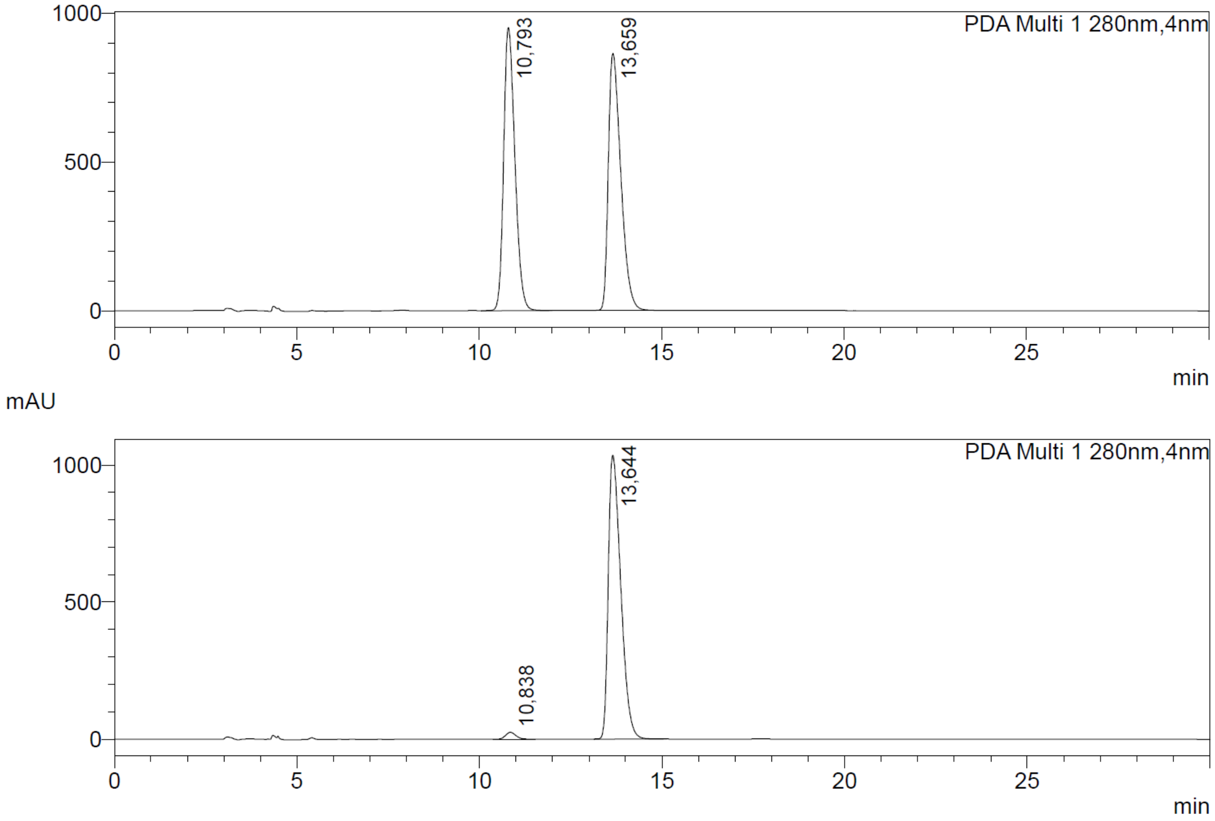

Figure S28. Chromatographic separation of the enantiomers of tetrahydro-β-carboline **3b** using HPLC method HPLC-C1. Racemic standard (upper), biotransformation using *Rs*STR V176L/V208A (lower).

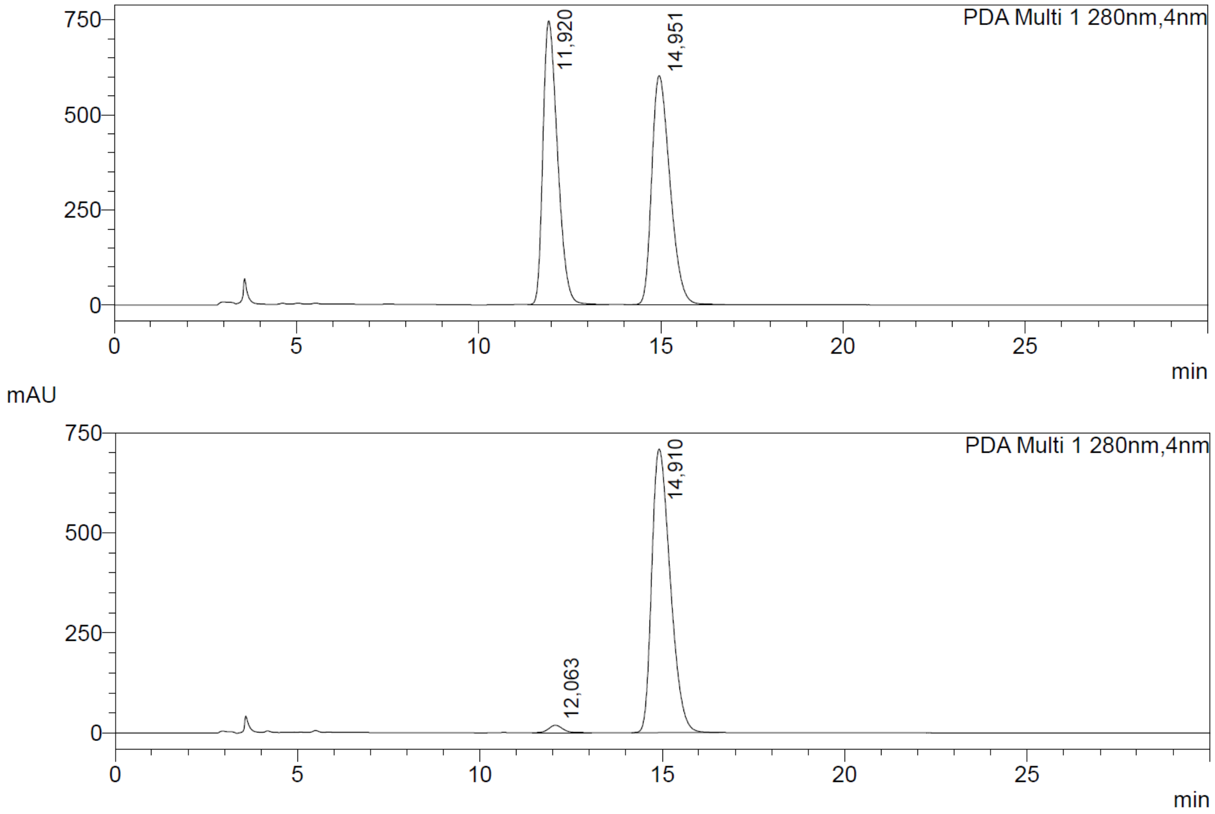

Figure S29. Chromatographic separation of the enantiomers of tetrahydro-β-carboline **3b** using HPLC method HPLC-C4. Racemic standard (upper), biotransformation using *Rs*STR V176L/V208A (lower).

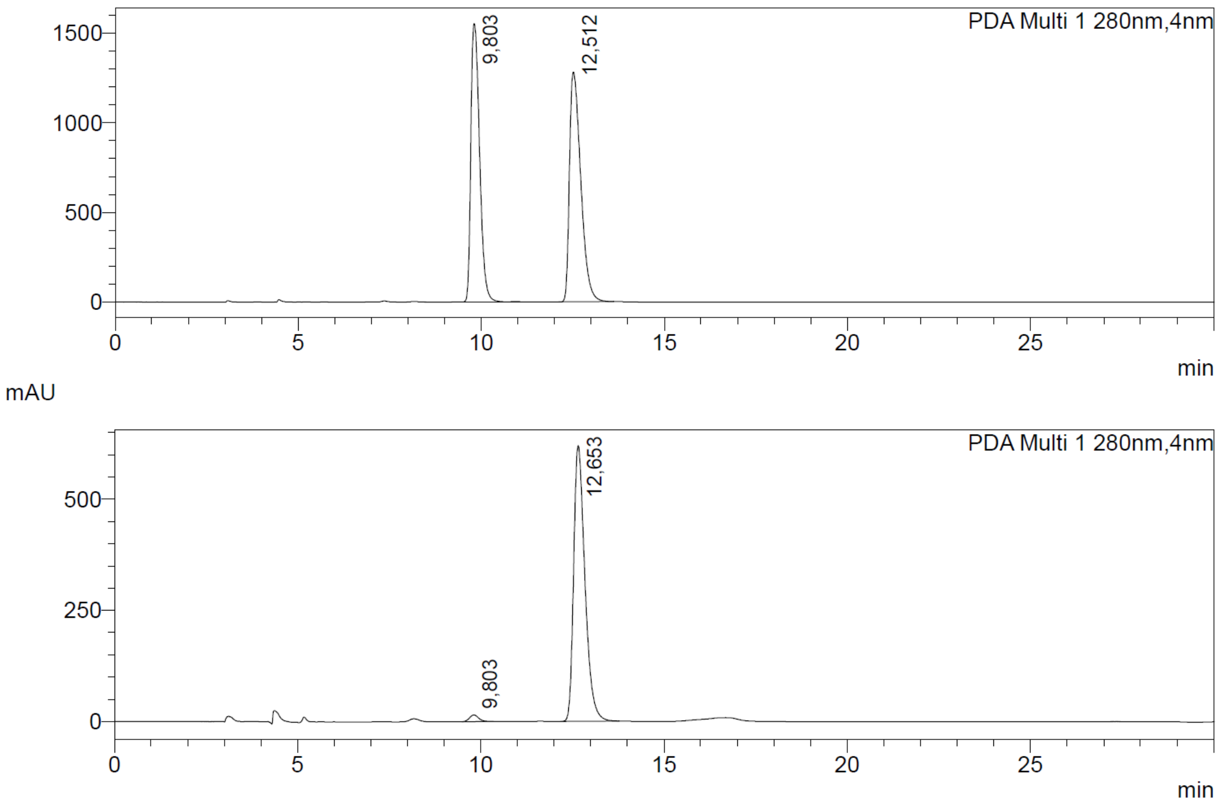

Figure S30. Chromatographic separation of the enantiomers of tetrahydro-β-carboline **3c** using HPLC method HPLC-C1. Racemic standard (upper), biotransformation using *Rs*STR V176L/V208A (lower).

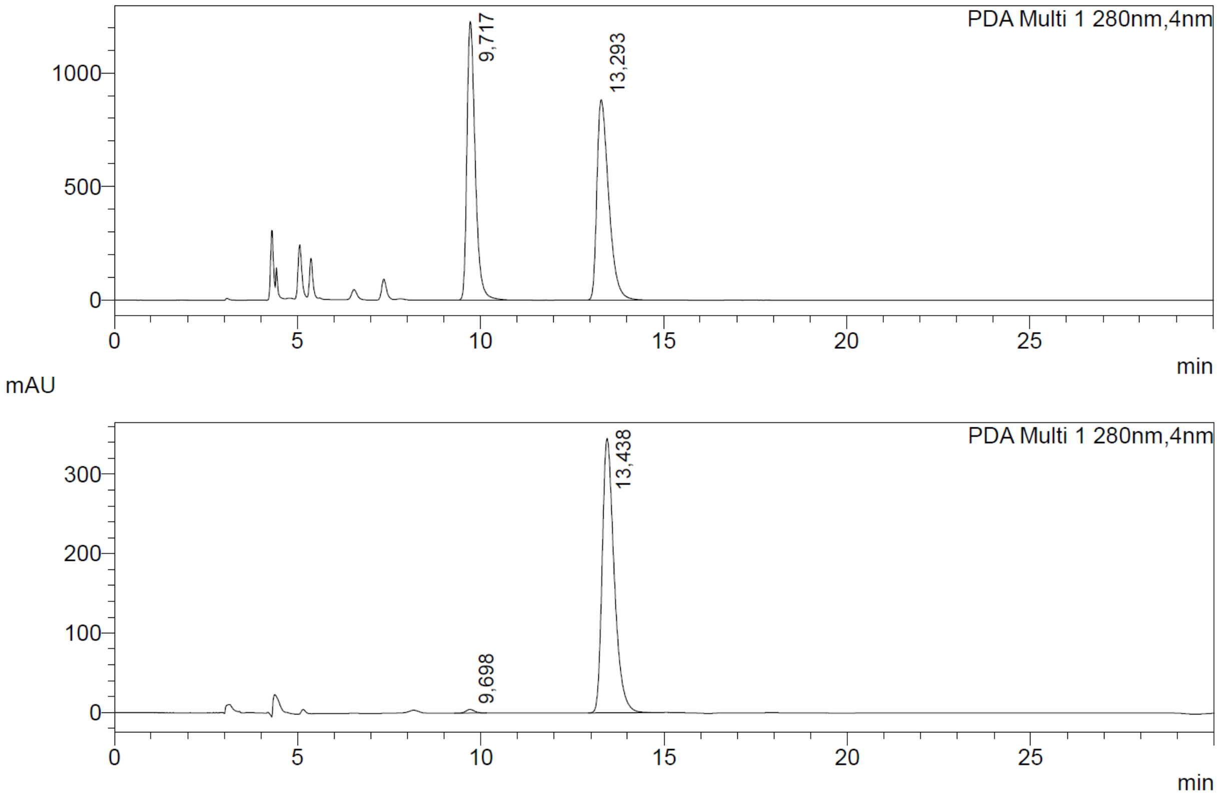

Figure S31. Chromatographic separation of the enantiomers of tetrahydro-β-carboline **3d** using HPLC method HPLC-C1. Racemic standard (upper), biotransformation using *Rs*STR V176L/V208A (lower).

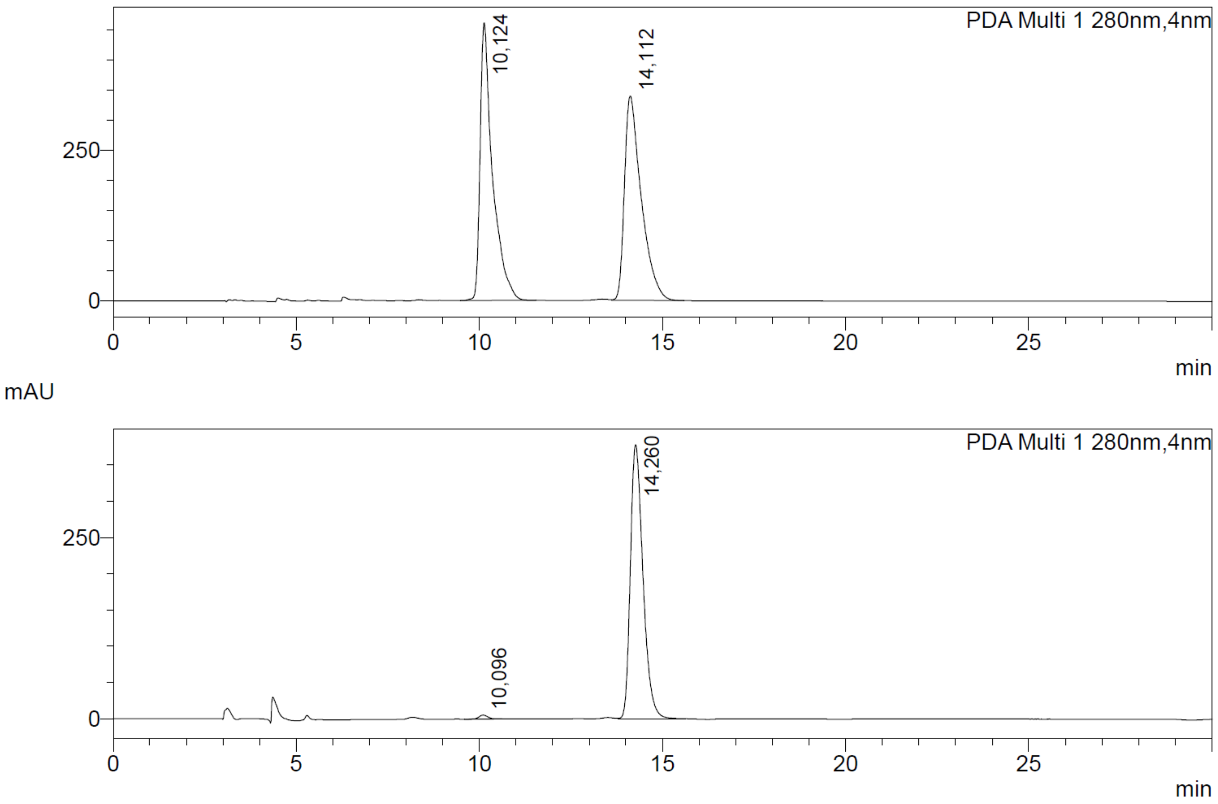

Figure S32. Chromatographic separation of the enantiomers of tetrahydro-β-carboline **3e** using HPLC method HPLC-C1. Racemic standard (upper), biotransformation using *Rs*STR V176L/V208A (lower).

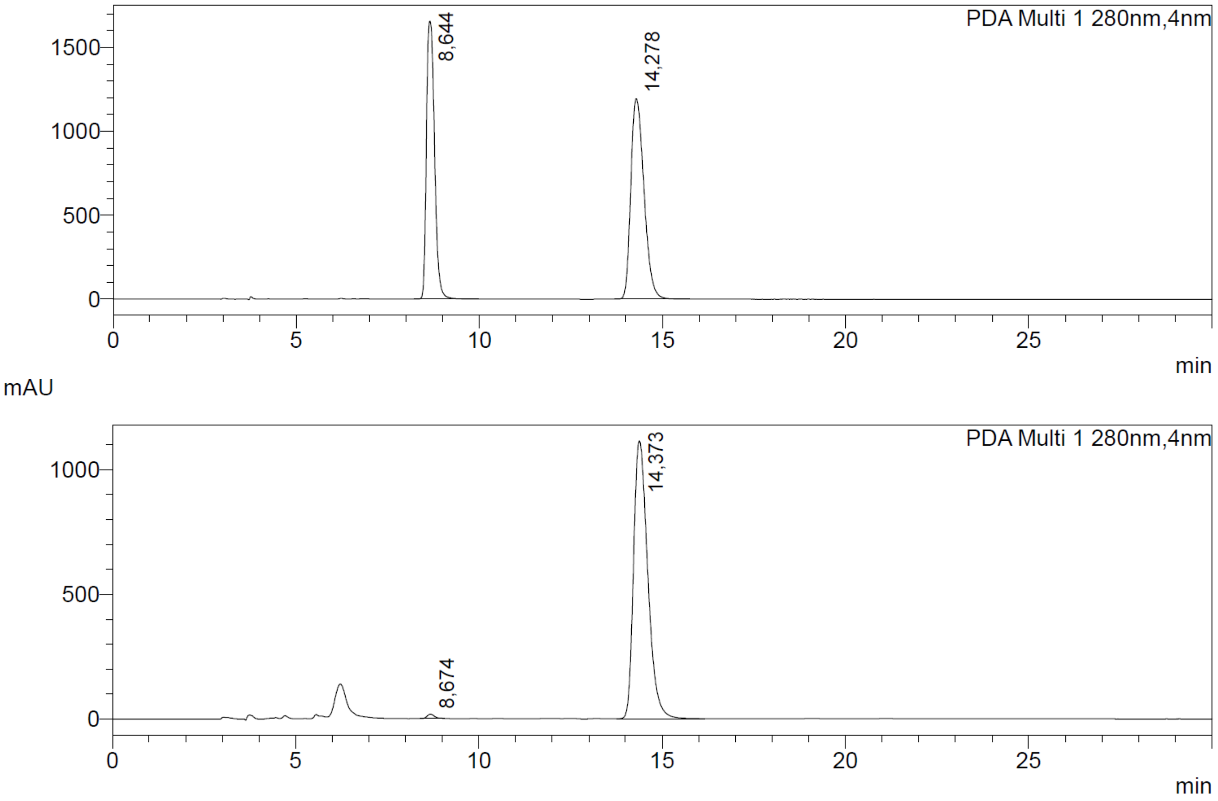

Figure S33. Chromatographic separation of the enantiomers of tetrahydro-β-carboline **3f** using HPLC method HPLC-C3. Racemic standard (upper), biotransformation using *Rs*STR V176L/V208A (lower).

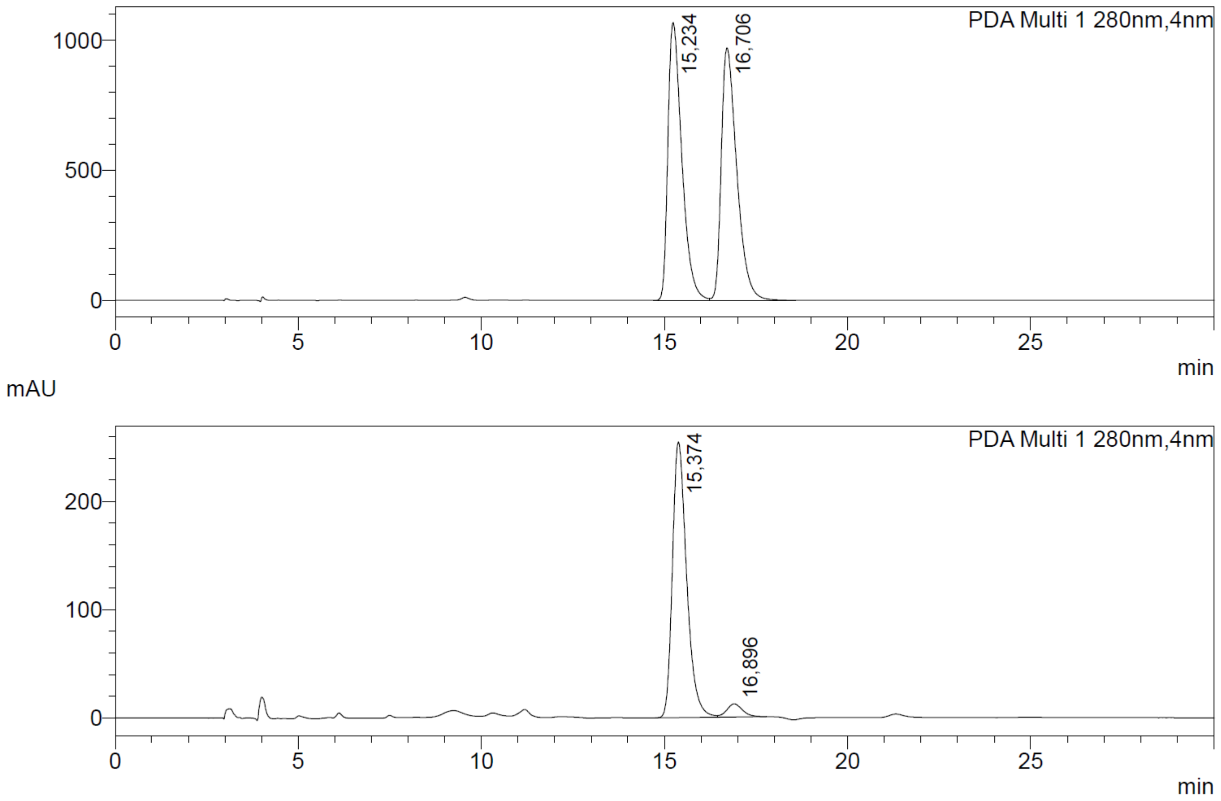

Figure S34. Chromatographic separation of the enantiomers of tetrahydro-β-carboline **3g** using HPLC method HPLC-C2. Racemic standard (upper), biotransformation using *Rs*STR V176L/V208A (lower).

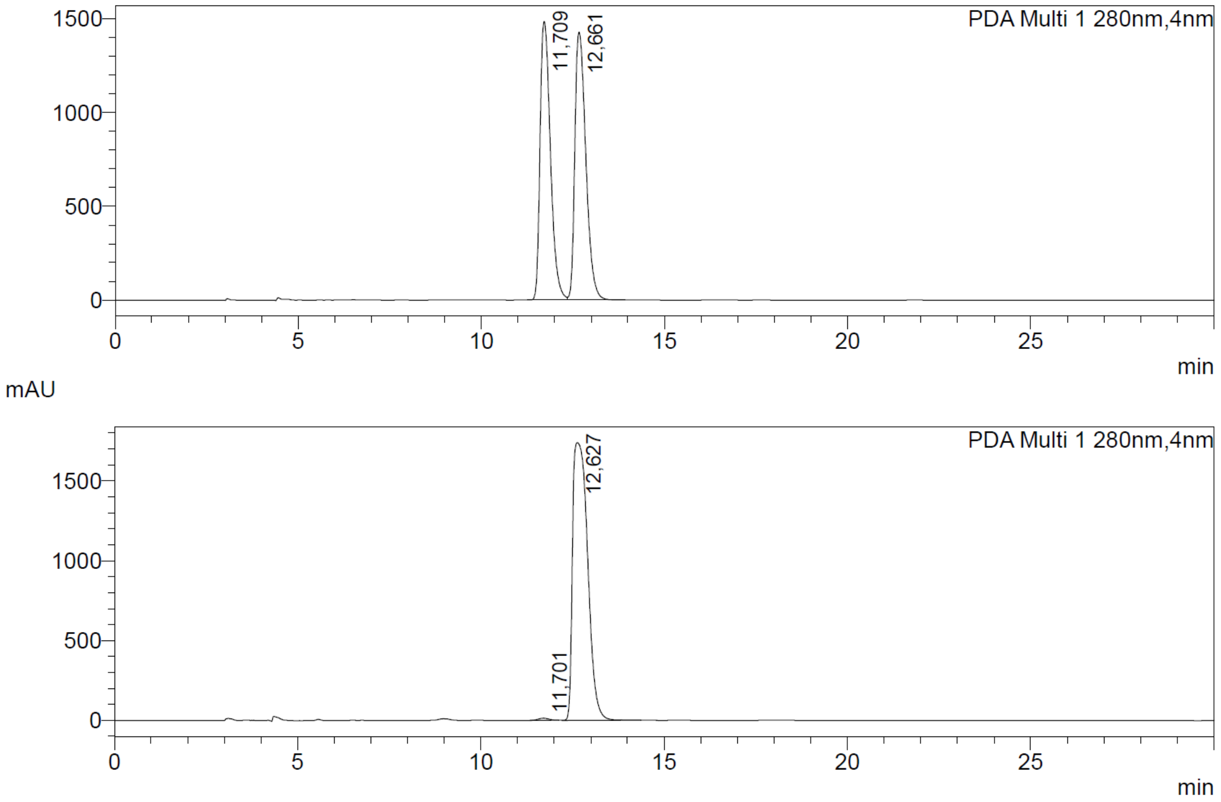

Figure S35. Chromatographic separation of the enantiomers of tetrahydro-β-carboline **3h** using HPLC method HPLC-C1. Racemic standard (upper), biotransformation using *Rs*STR V176L/V208A (lower).

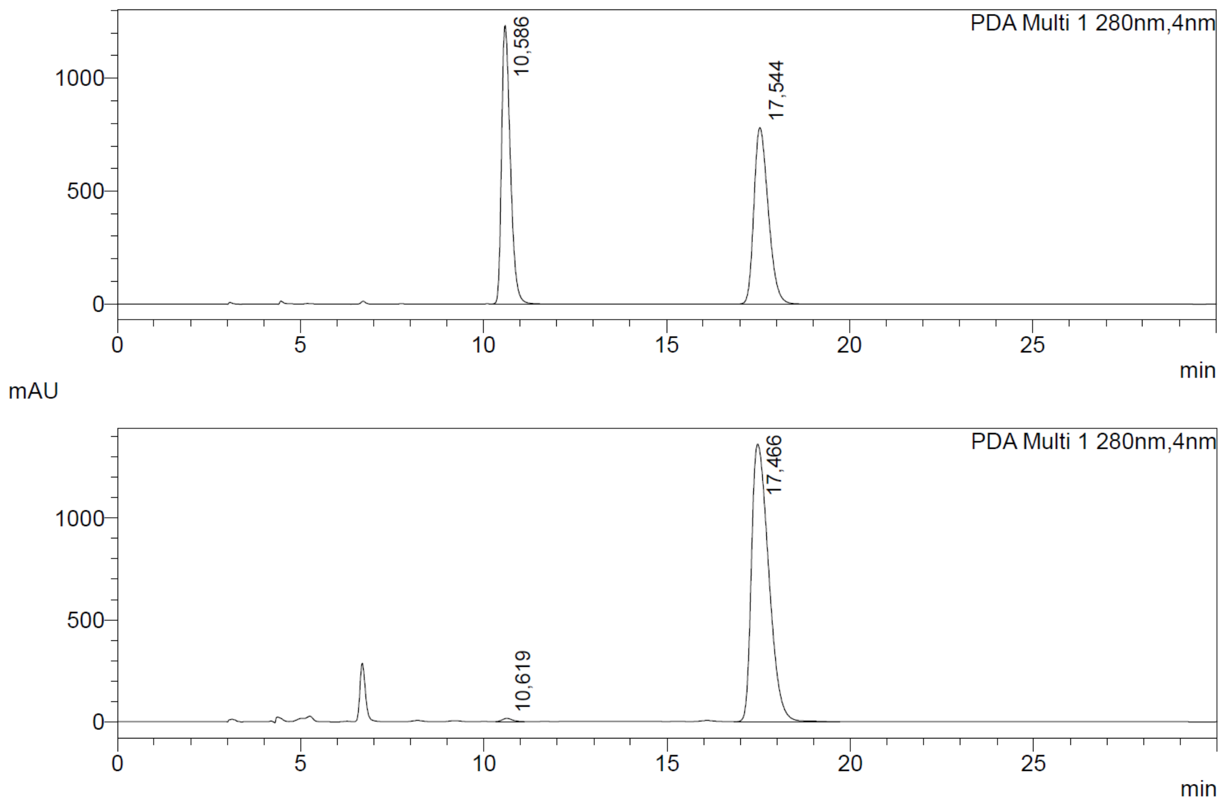

Figure S36. Chromatographic separation of the enantiomers of tetrahydro-β-carboline **3i** using HPLC method HPLC-C1. Racemic standard (upper), biotransformation using *Rs*STR V176L/V208A (lower).

Determination of absolute configurations:

The absolute configurations of tetrahydro-β-carbolines (*R*)-**3a**, (*R*)-**3b**, (*R*)-**3h** and (*R*)-**3i** were determined by one or more of the following methods: (*i*) Comparison of HPLC elution order of enantiomers with the elution order reported in the literature under identical chromatographic conditions (Chiralcel OD-H column, method HPLC-C4), (*ii*) comparison of the optical rotation of the compound isolated from a preparative-scale biotransformation using *Rs*STR V176L/V208A with values reported in the literature, (*iii*) comparison of the circular dichroism (CD) spectrum with the spectra of close structural analogues whose absolute configurations were assigned based on the other methods. Table S15 summarises the methods used for each tetrahydro-β-carboline and lists the literature references used, where applicable.

Table S15. Summary of methods used for the determination of absolute configurations, including literature references.

Compound	Elution order	Optical rotation	Circular dichroism
(*R*)-**3a**	[16]	[15]	X
(*R*)-**3b**	[16]	[16]	X
(*R*)-**3h**			X
(*R*)-**3i**		[17]	X

The CD spectra (wavelength range: 200–320 nm) of the tetrahydro-β-carbolines isolated from preparative-scale biotransformations (**3a**, **3b**, **3h** and **3i**) were recorded at 0.05 mM concentration in MeOH solution using a *Jasco* J-1500 CD spectrometer and a quartz cuvette with 10 mm path length. The raw spectra were baseline-corrected by subtracting the spectrum of the solvent (MeOH) and smoothened by the means-movement method (convolution width = 15) using the *Jasco* Spectra Manager Version 2 software. The processed spectra are shown in Figure S37 (overleaf). All four spectra show similar characteristics, including a positive Cotton effect at around 210 nm, a negative Cotton effect between 250 and 260 nm, and a small positive bump at slightly above 290 nm. The matching spectral features indicate that the four compounds have the same absolute configuration. **3a** and **3b** were shown to be (*R*)-configured by two independent methods (elution order and optical rotation), and **3i** by one (optical rotation, albeit with a very low numerical value). Therefore, the absolute configuration of **3h**, for which no literature reference data were available, was also assigned as (*R*).

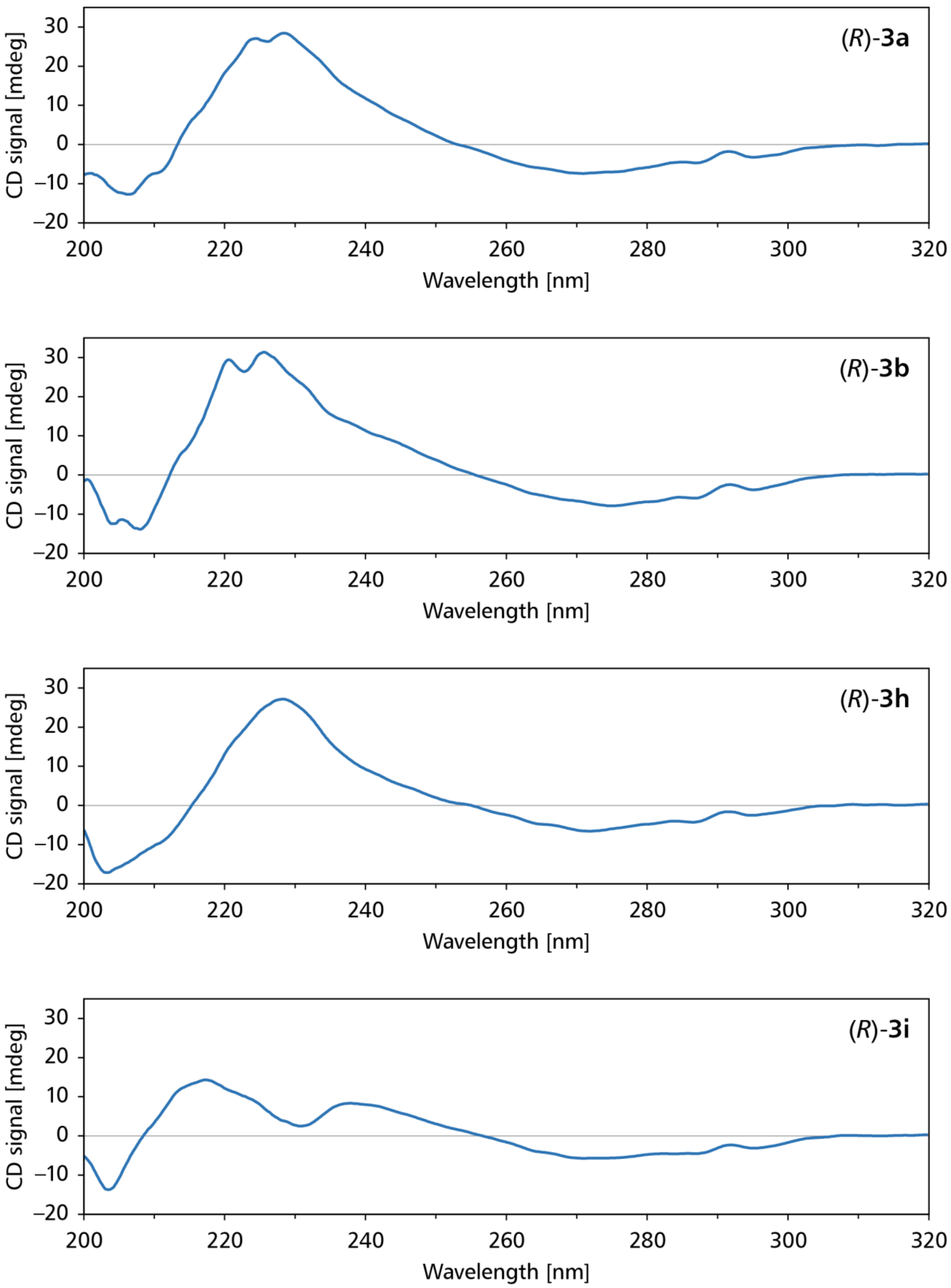

Figure S37. Circular dichroism (CD) spectra of tetrahydro-β-carbolines isolated from preparative-scale biotransformations. The matching spectral features support the conclusion that the four compounds have the same absolute configuration.

NMR and MS Spectra

rac-1-(*sec*-Butyl)-2,3,4,9-tetrahydro-1*H*-pyrido[3,4-*b*]indole hydrochloride (5a·HCl)

- ^{1}H-NMR spectrum (Figure S38)
- ^{13}C-NMR spectrum (Figure S39)
- COSY-NMR spectrum (Figure S40)
- HSQC-NMR spectrum (Figure S41)
- MS spectrum (Figure S42)
- HRMS spectrum (Figure S43)

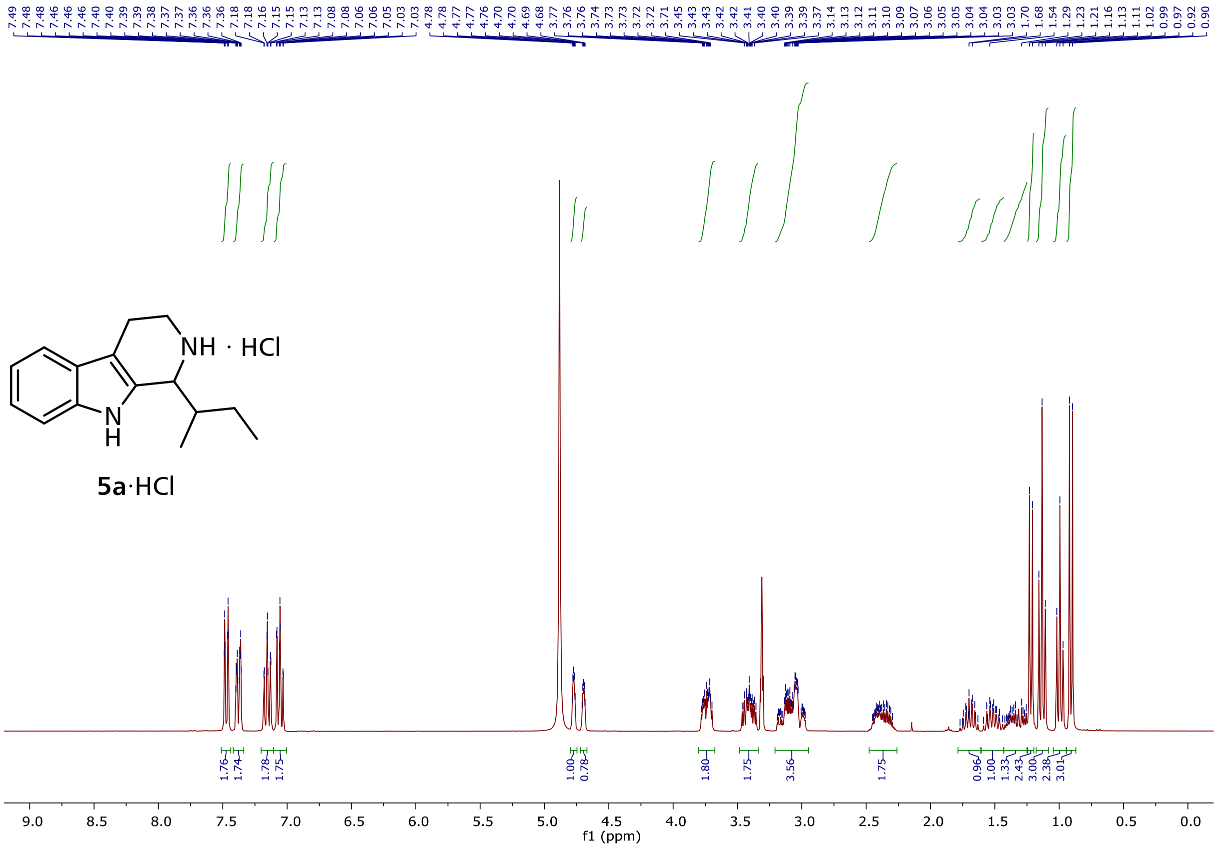

Figure S38. ^{1}H-NMR spectrum of *rac*-**5a**·HCl.

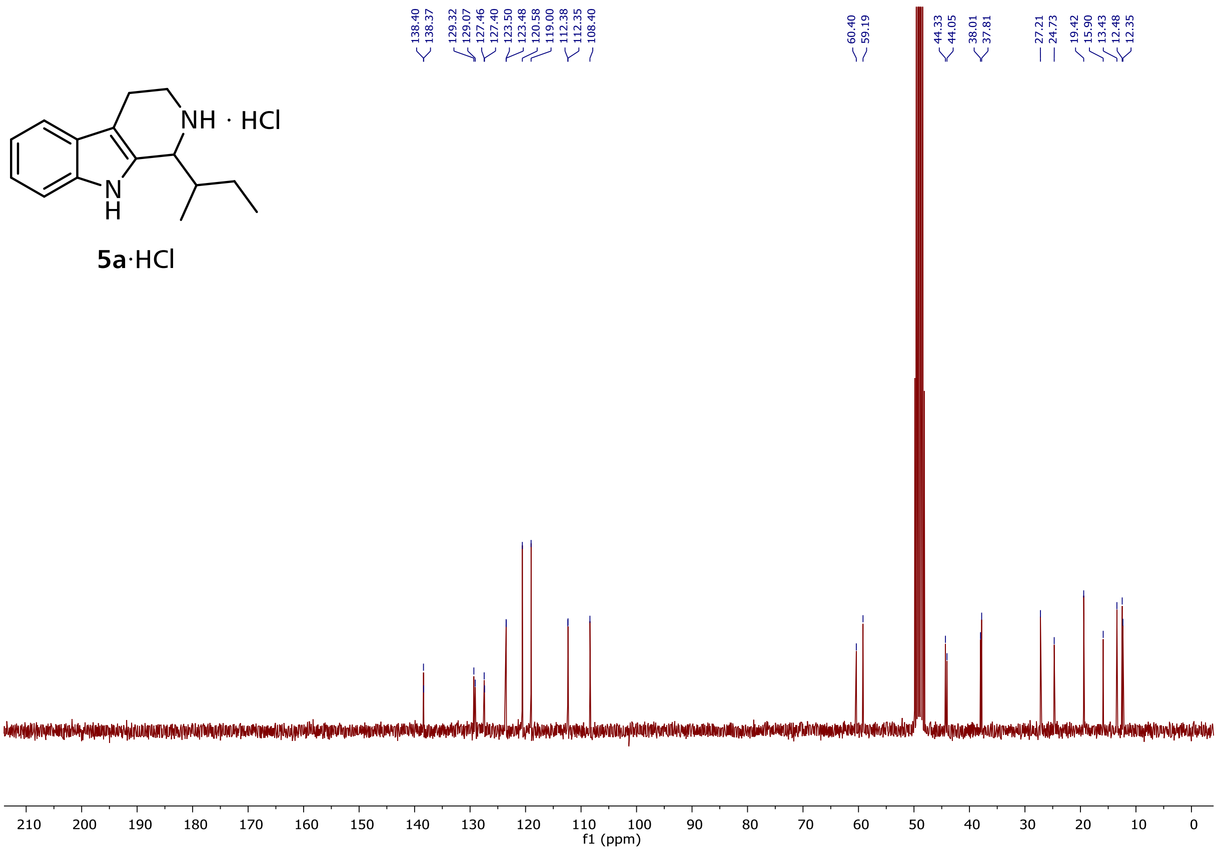

Figure S39. ^{13}C-NMR spectrum of *rac*-**5a**·HCl.

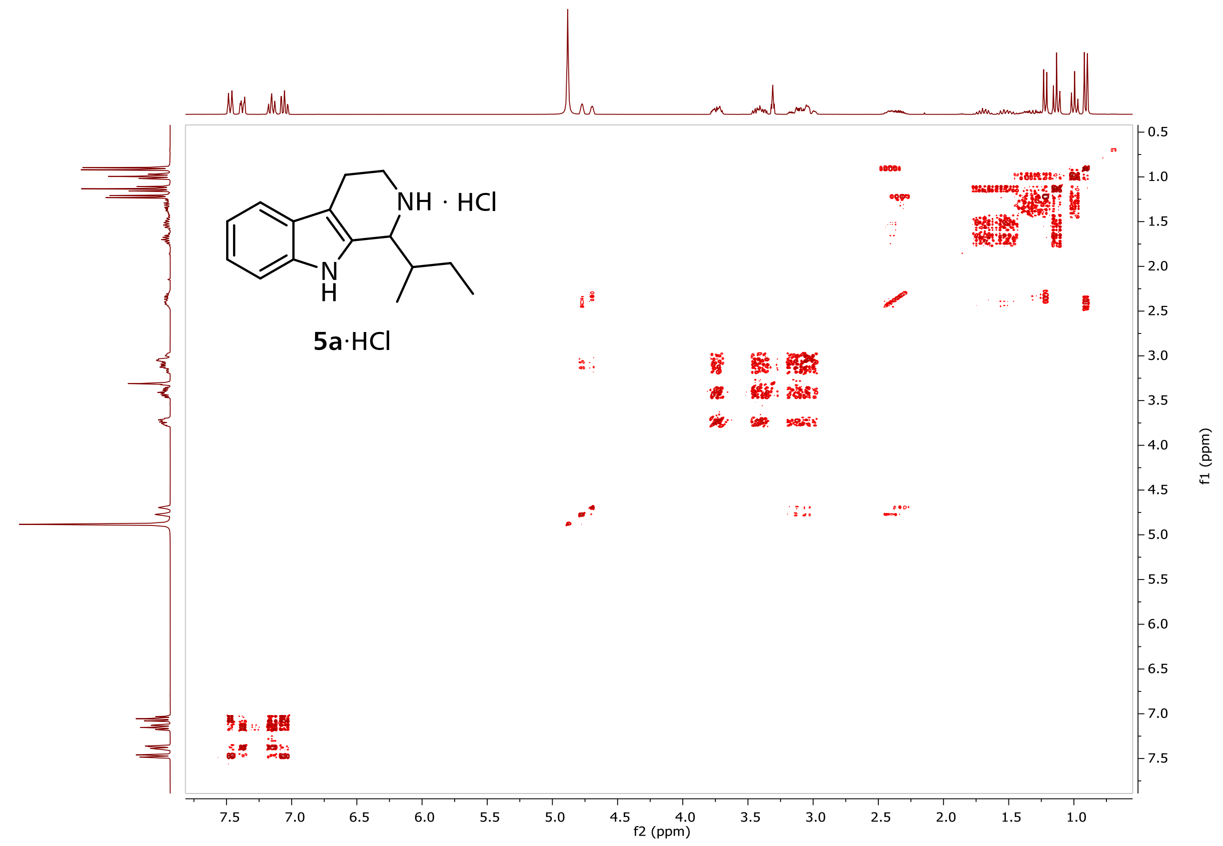

Figure S40. COSY-NMR spectrum of *rac*-**5a**·HCl.

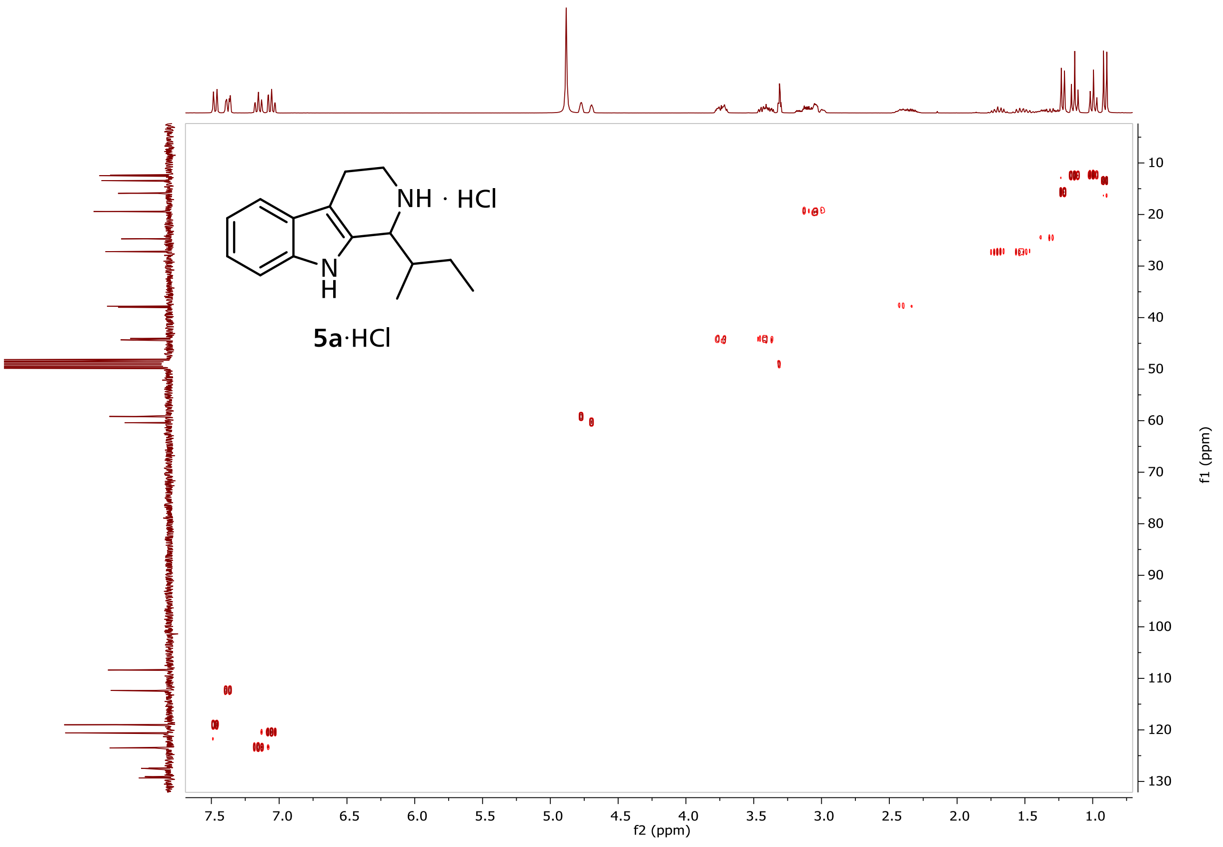

Figure S41. HSQC-NMR spectrum of *rac*-**5a**·HCl.

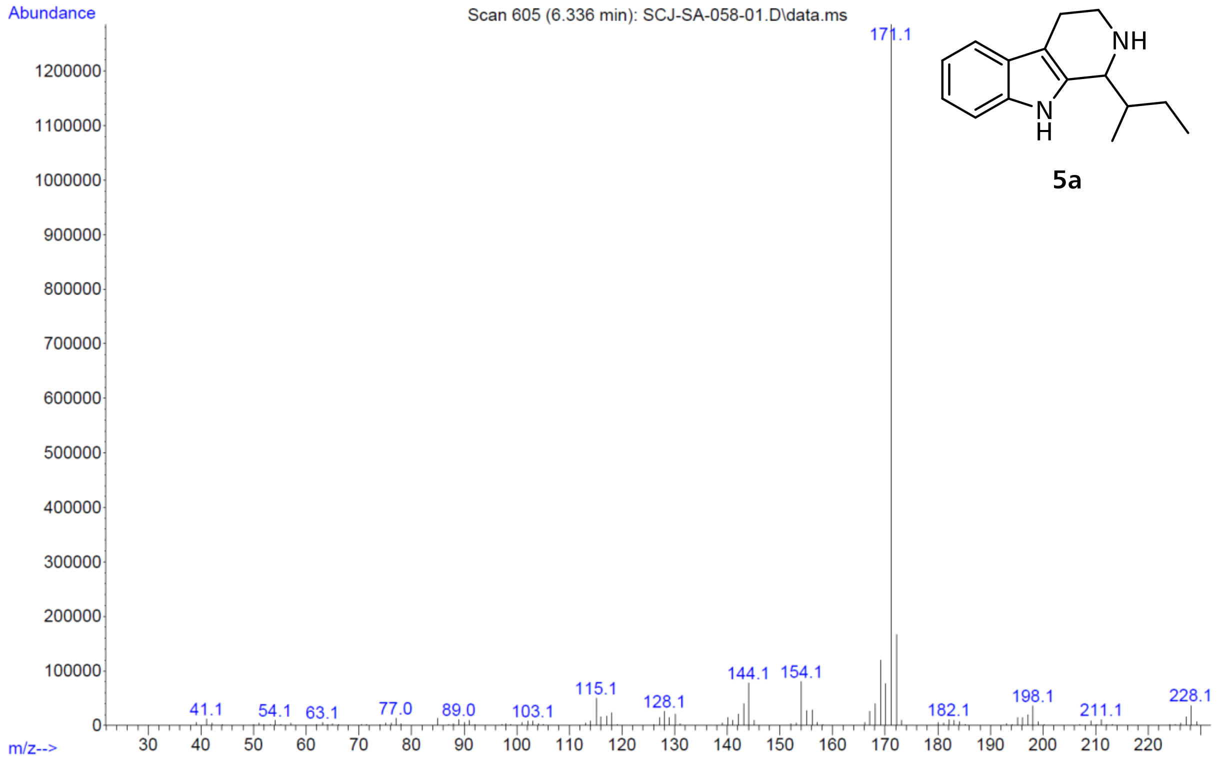

Figure S42. MS spectrum of *rac*-**5a**.

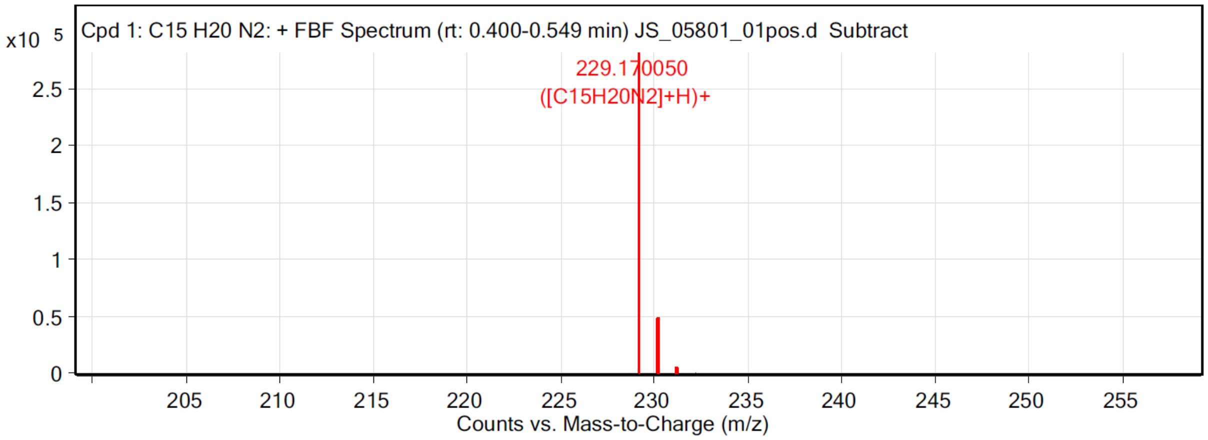

Figure S43. HRMS spectrum of *rac*-**5a**.

rac-1-Phenyl-2,3,4,9-tetrahydro-1*H*-pyrido[3,4-*b*]indole hydrochloride (3a·HCl)

- ▶ ^{1}H-NMR spectrum (Figure S44)
- ▶ ^{13}C-NMR spectrum (Figure S45)
- ▶ MS spectrum (Figure S46)
- ▶ HRMS spectrum (Figure S47)

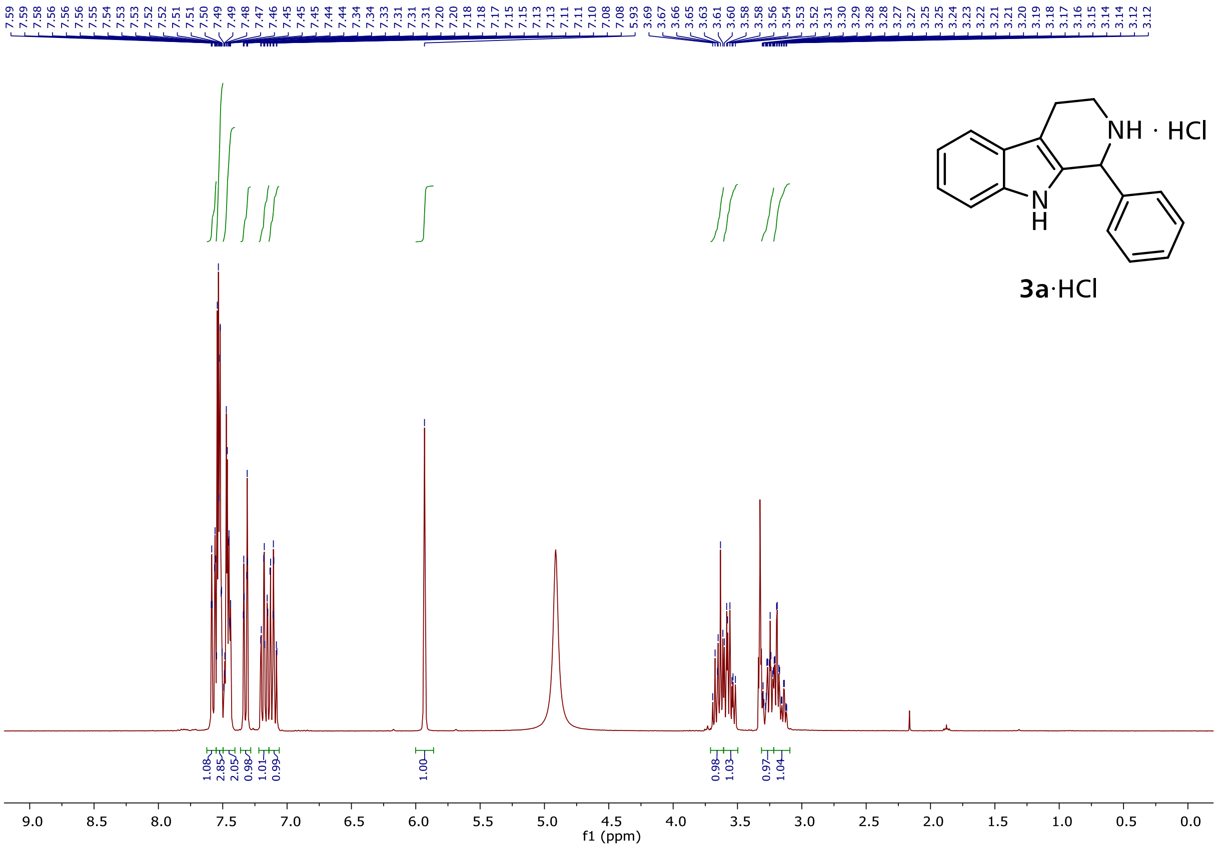

Figure S44. ^{1}H-NMR spectrum of *rac*-**3a**·HCl.

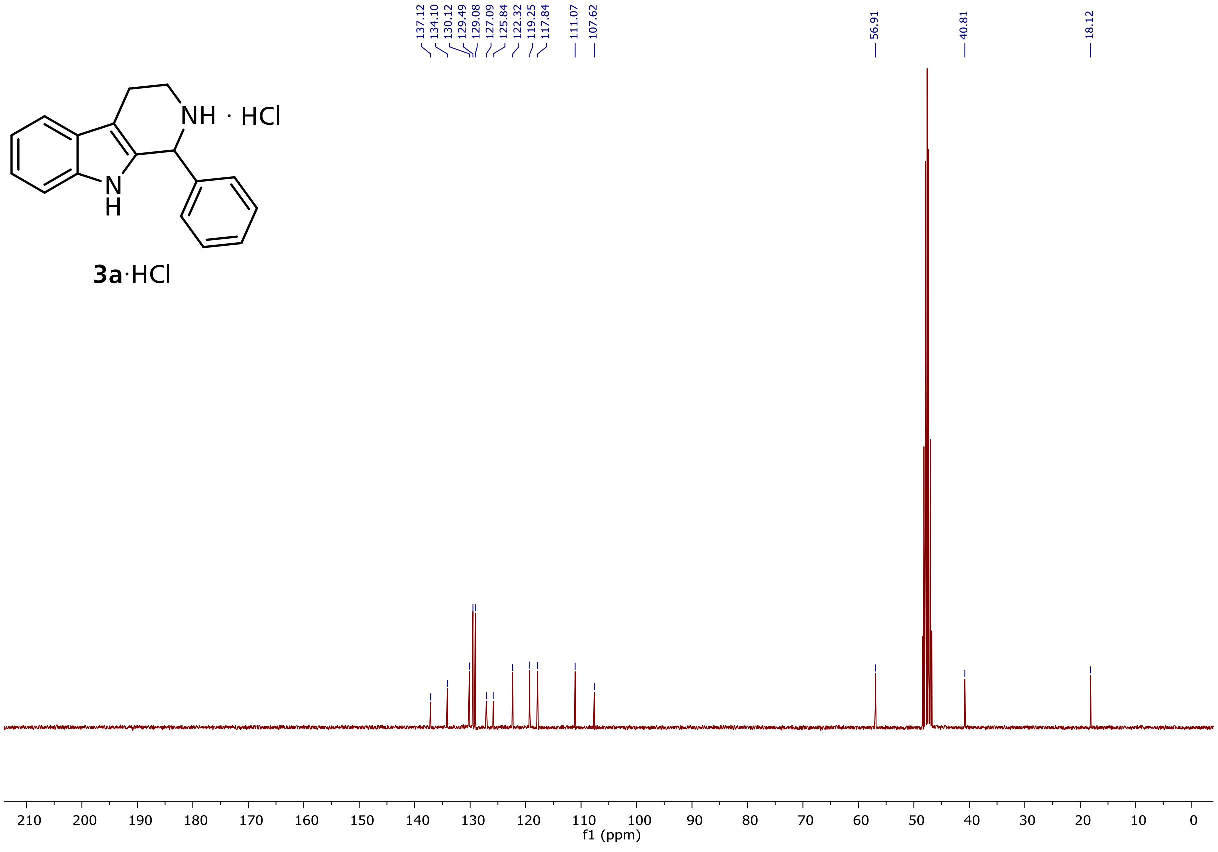

Figure S45. ^{13}C-NMR spectrum of *rac*-**3a**·HCl.

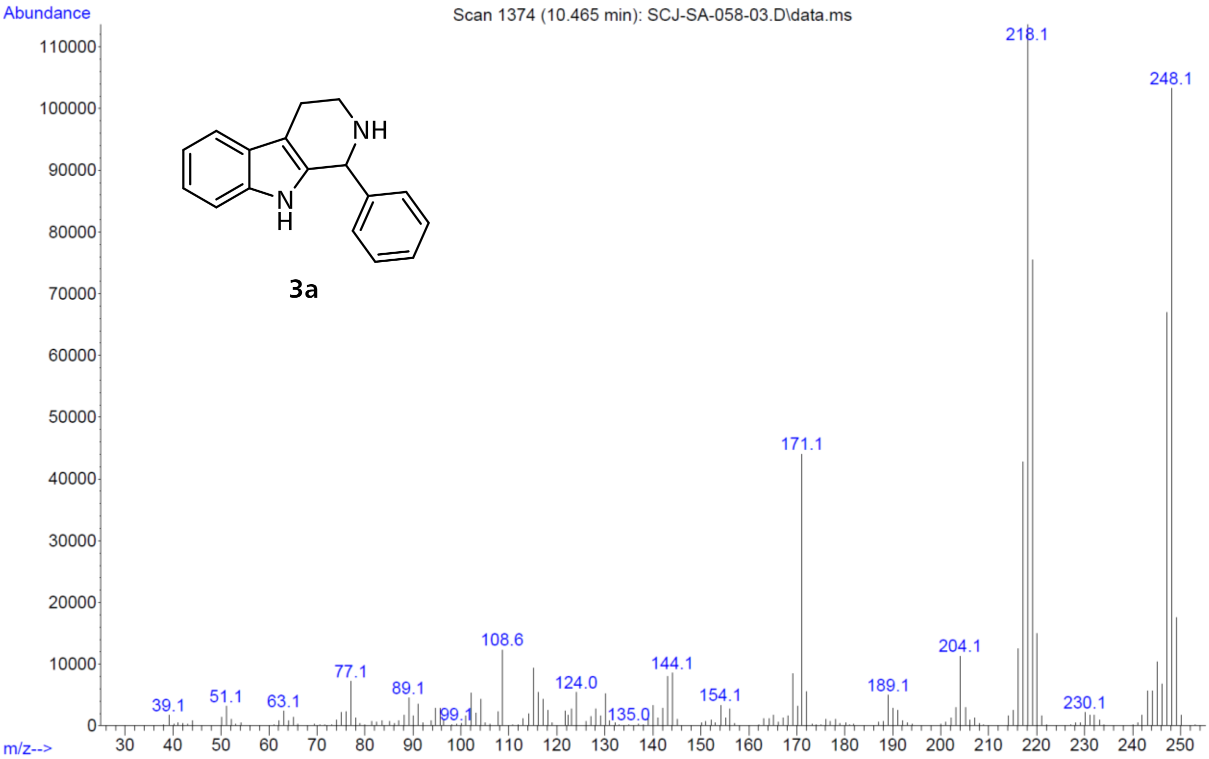

Figure S46. MS spectrum of *rac*-**3a**.

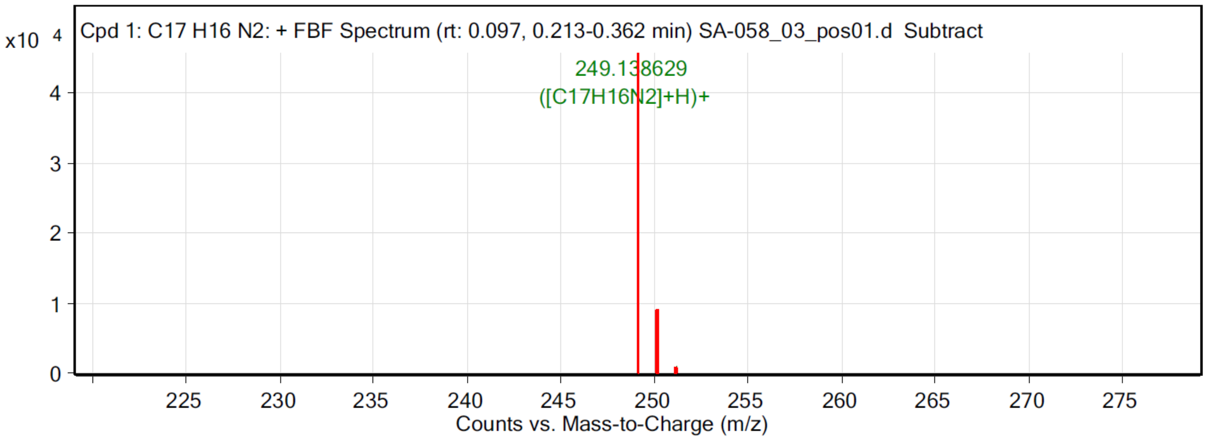

Figure S47. HRMS spectrum of *rac*-**3a**.

rac-1-(4-Fluorophenyl)-2,3,4,9-tetrahydro-1*H*-pyrido[3,4-*b*]indole hydrochloride (3b·HCl)

- ^{1}H-NMR spectrum (Figure S48)
- ^{13}C-NMR spectrum (Figure S49)
- MS spectrum (Figure S50)
- HRMS spectrum (Figure S51)

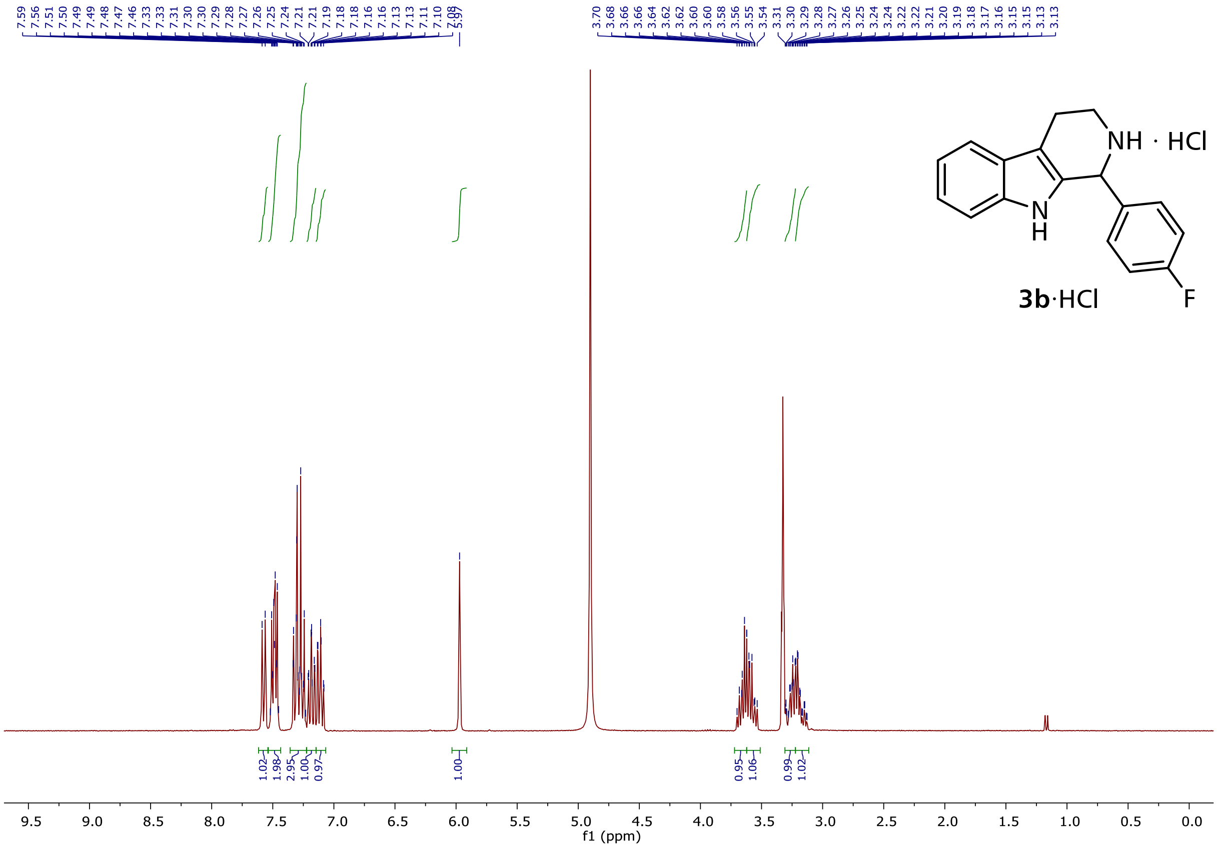

Figure S48. ^{1}H-NMR spectrum of *rac*-**3b**·HCl.

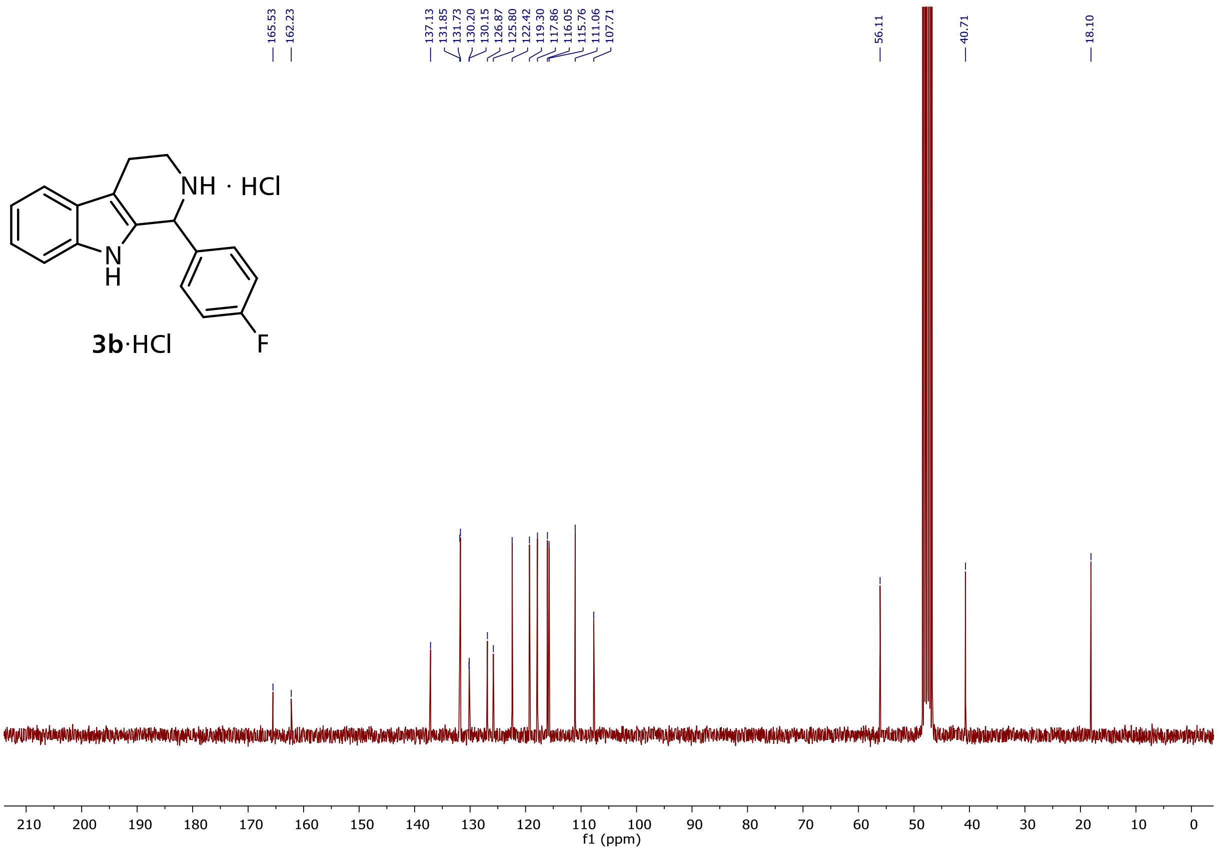

Figure S49. ^{13}C-NMR spectrum of *rac*-**3b**·HCl.

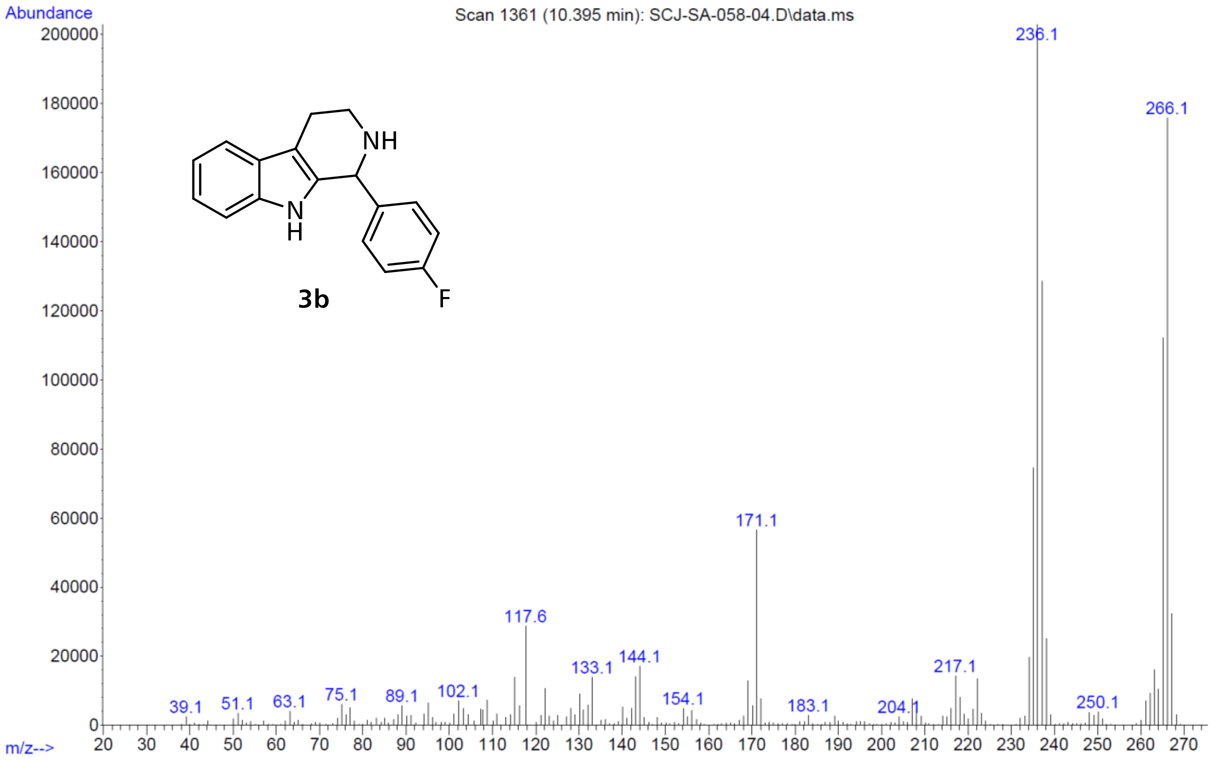

Figure S50. MS spectrum of *rac*-**3b**.

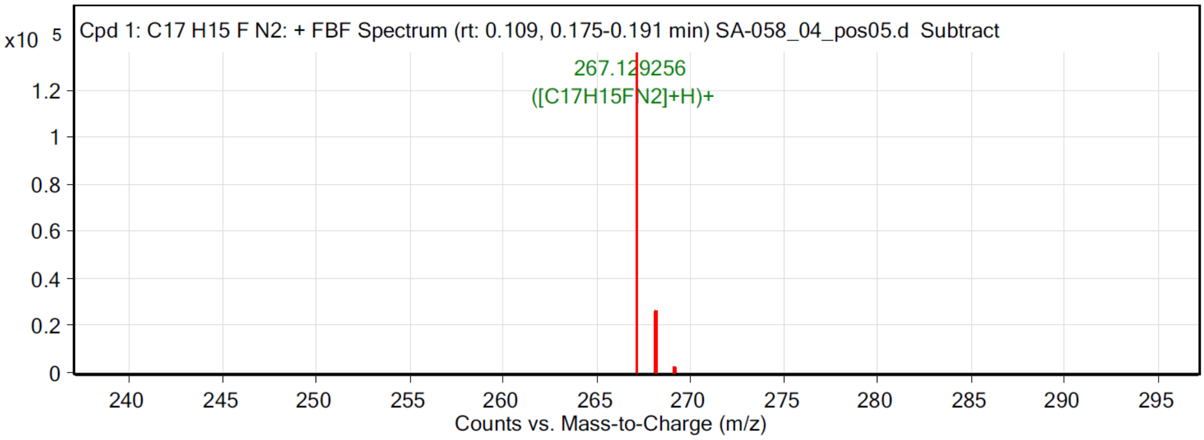

Figure S51. HRMS spectrum of *rac*-**3b**.

rac-1-(3-Fluorophenyl)-2,3,4,9-tetrahydro-1*H*-pyrido[3,4-*b*]indole hydrochloride (3c·HCl)

- ▶ ^{1}H-NMR spectrum (Figure S52)
- ▶ ^{13}C-NMR spectrum (Figure S53)
- ▶ MS spectrum (Figure S54)
- ▶ HRMS spectrum (Figure S55)

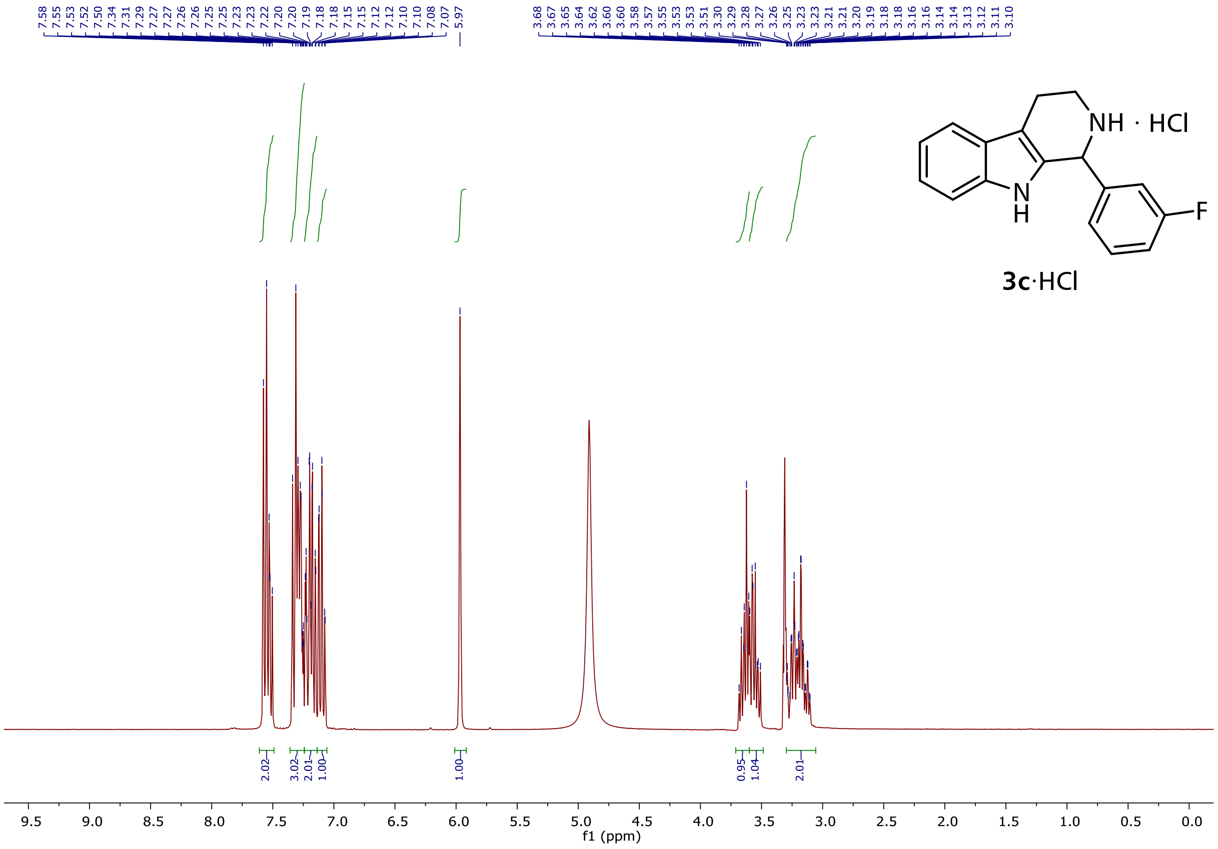

Figure S52. ^{1}H-NMR spectrum of *rac*-**3c**·HCl.

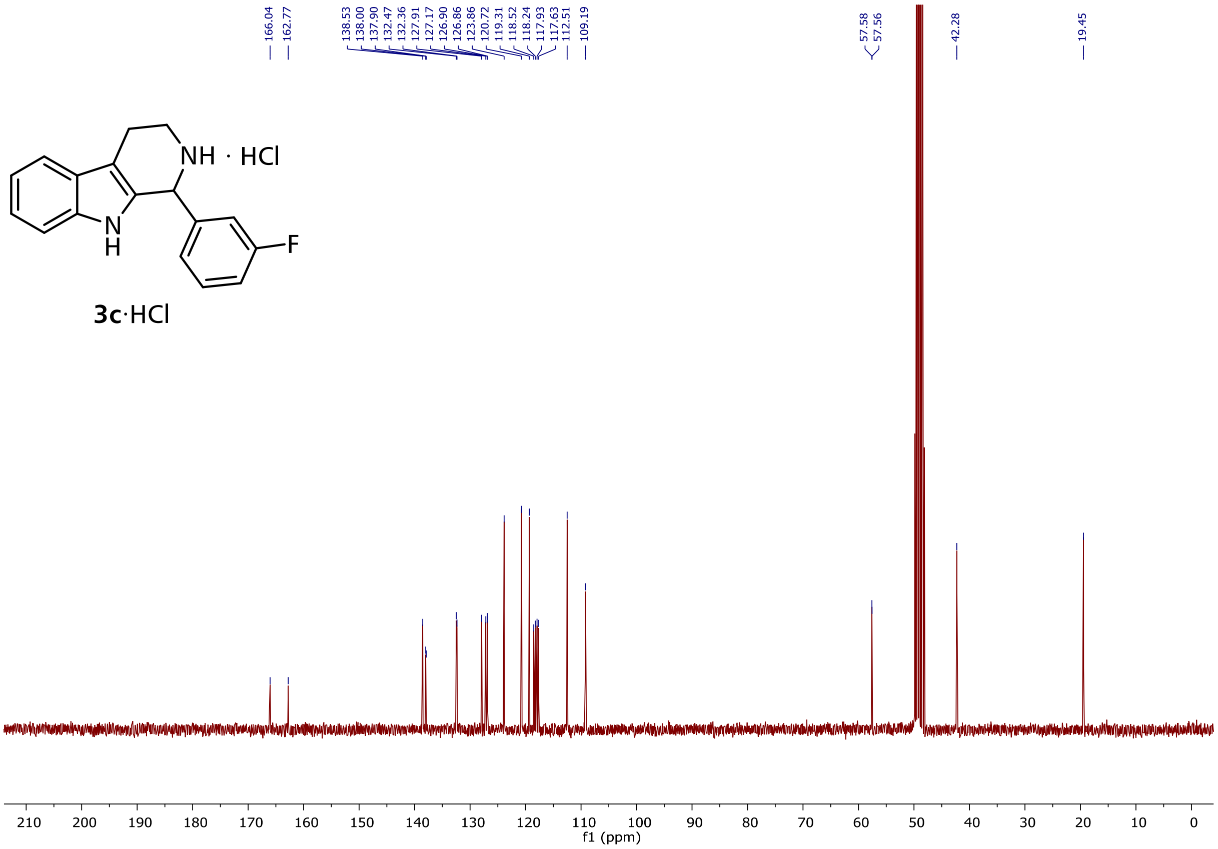

Figure S53. ^{13}C-NMR spectrum of *rac*-**3c**·HCl.

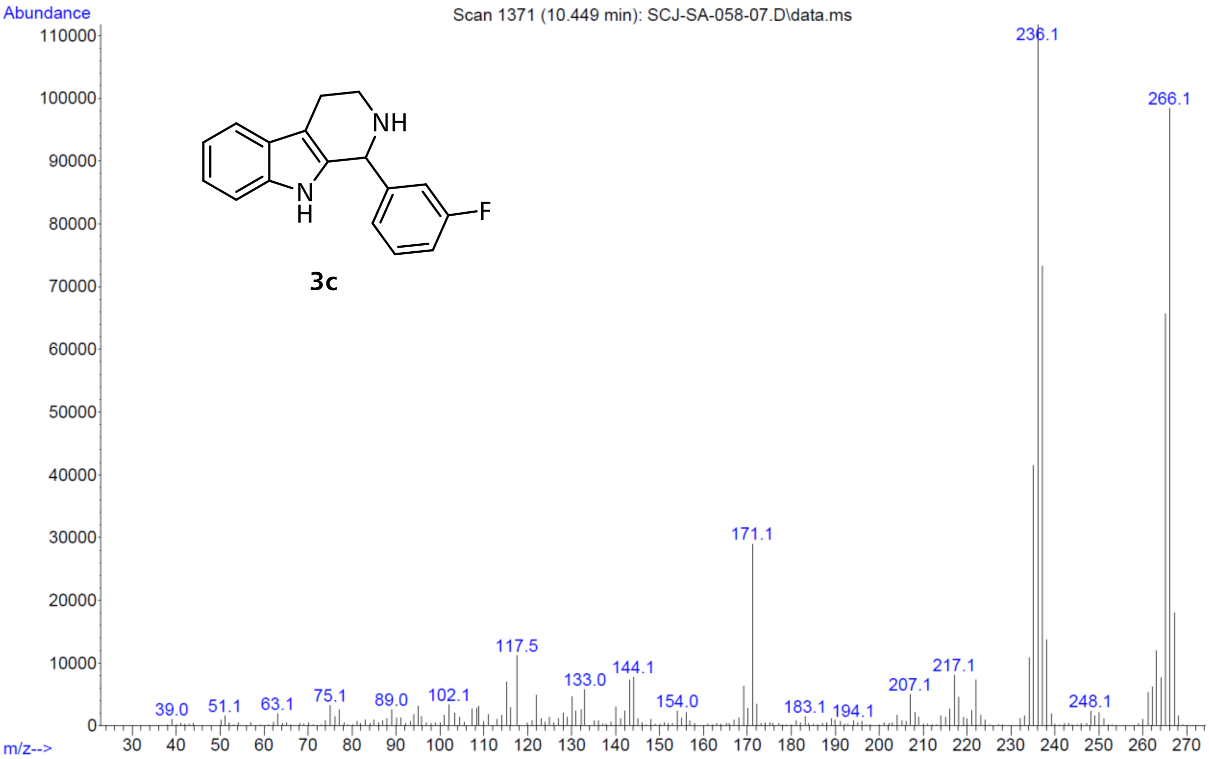

Figure S54. MS spectrum of *rac*-**3c**.

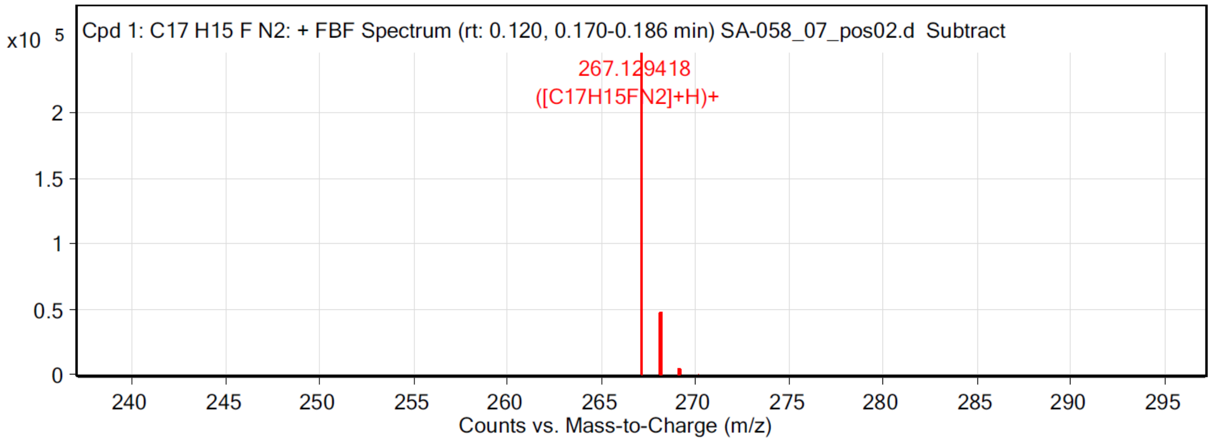

Figure S55. HRMS spectrum of *rac*-**3c**.

rac-1-(3-Chlorophenyl)-2,3,4,9-tetrahydro-1*H*-pyrido[3,4-*b*]indole hydrochloride (3d·HCl)

- ^{1}H-NMR spectrum (Figure S56)
- ^{13}C-NMR spectrum (Figure S57)
- MS spectrum (Figure S58)
- HRMS spectrum (Figure S59)

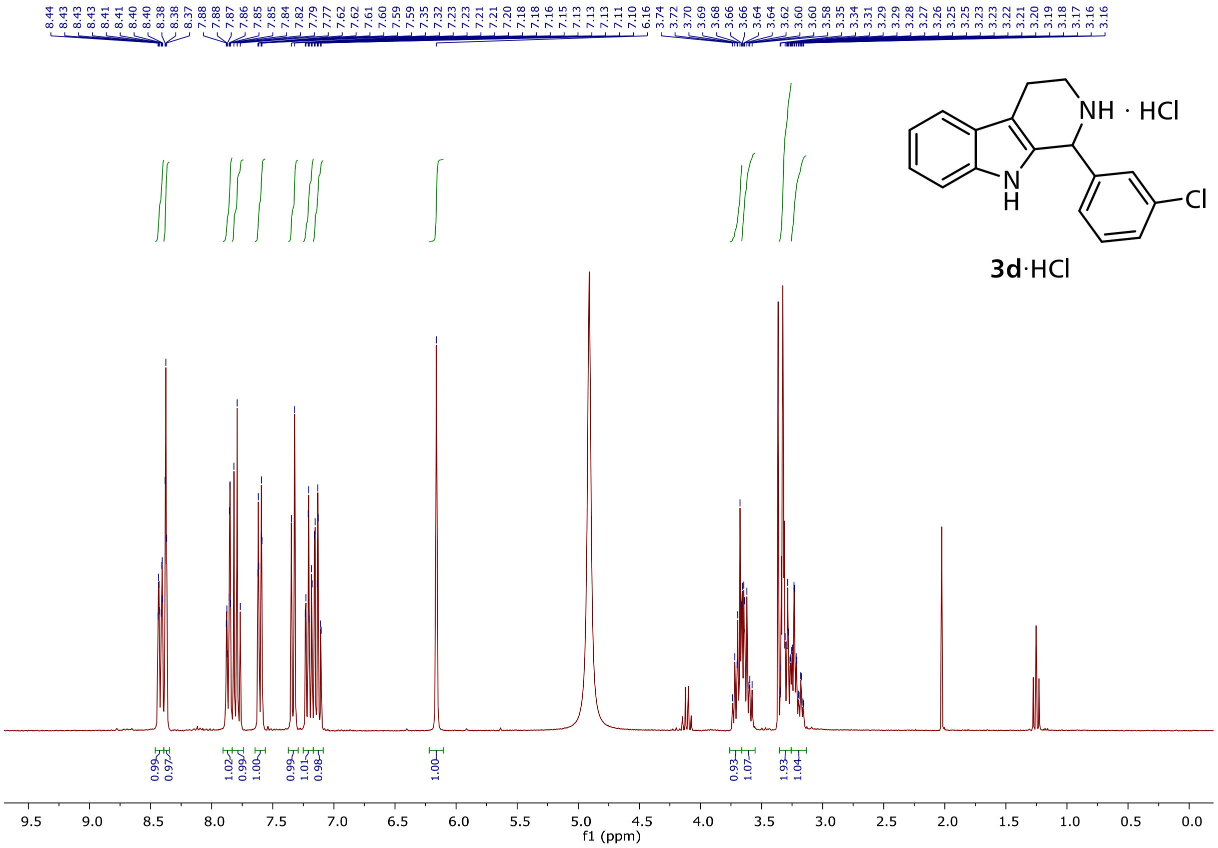

Figure S56. ^{1}H-NMR spectrum of *rac*-**3d**·HCl.

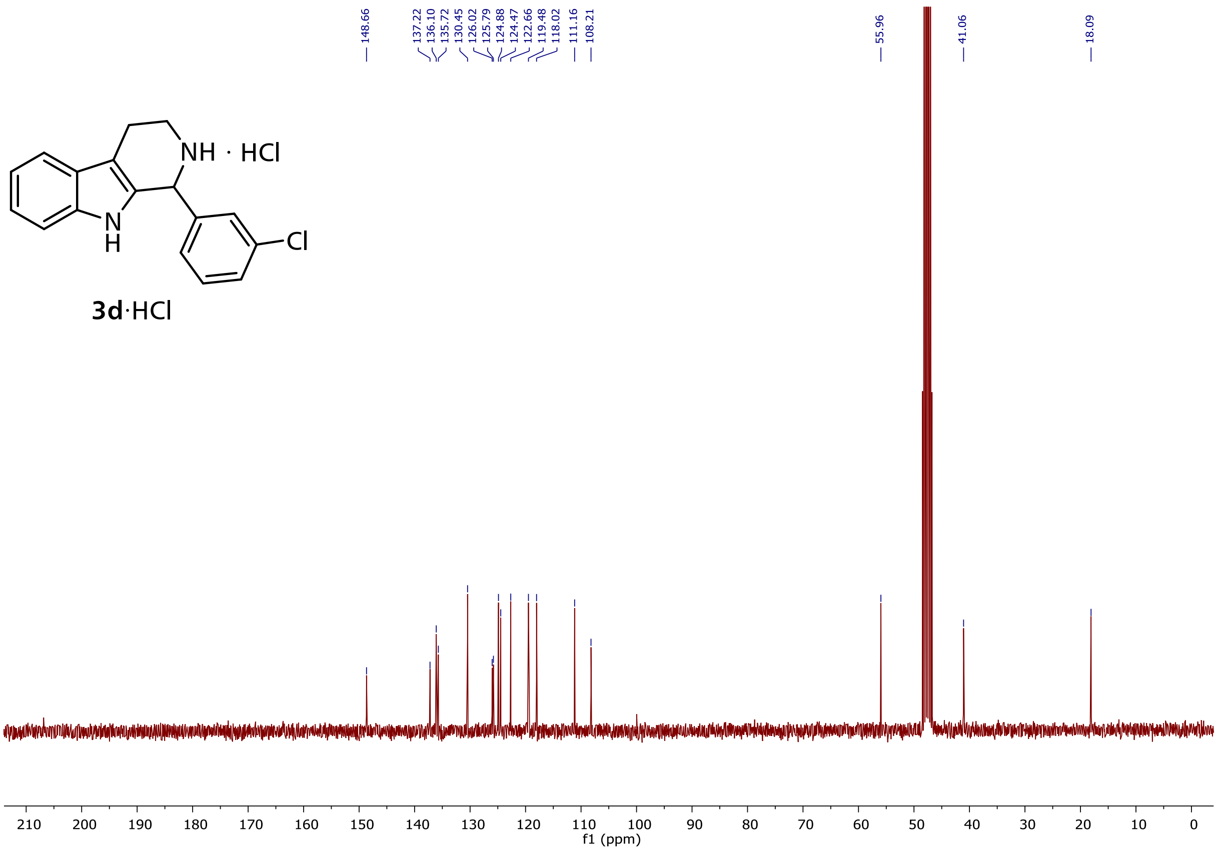

Figure S57. ^{13}C-NMR spectrum of *rac*-**3d**·HCl.

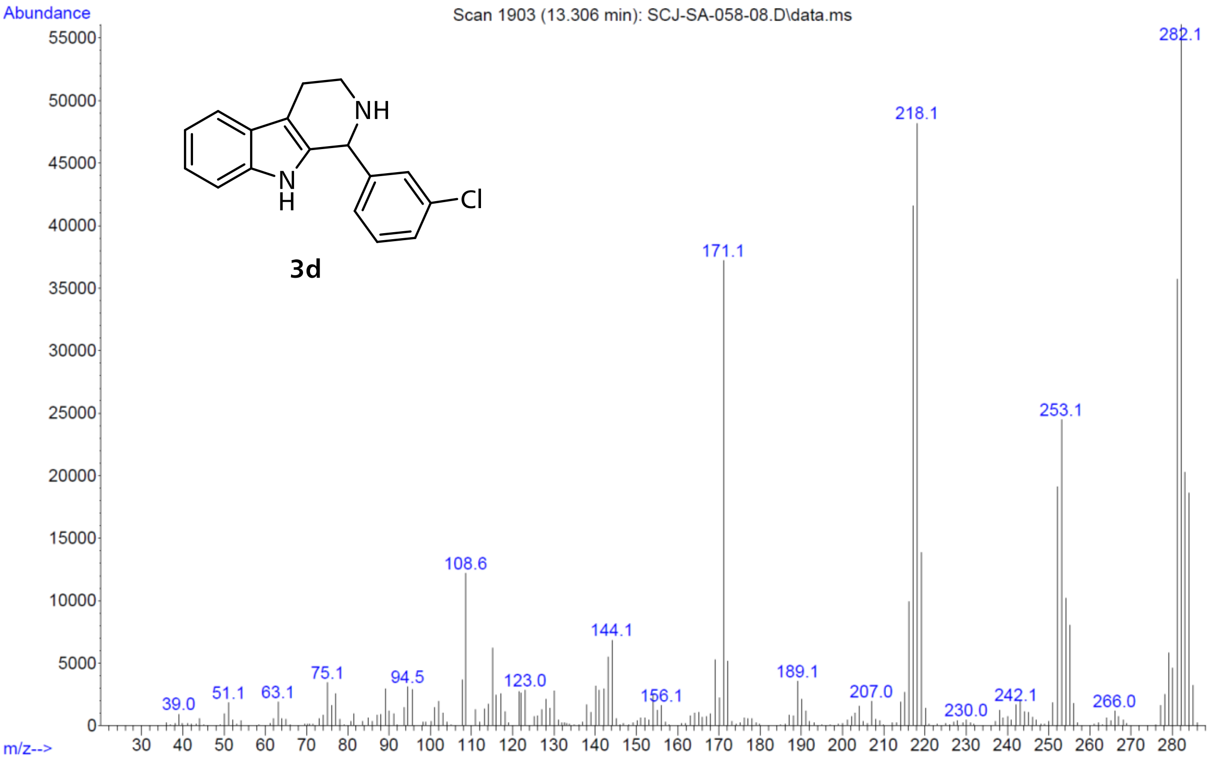

Figure S58. MS spectrum of *rac*-**3d**.

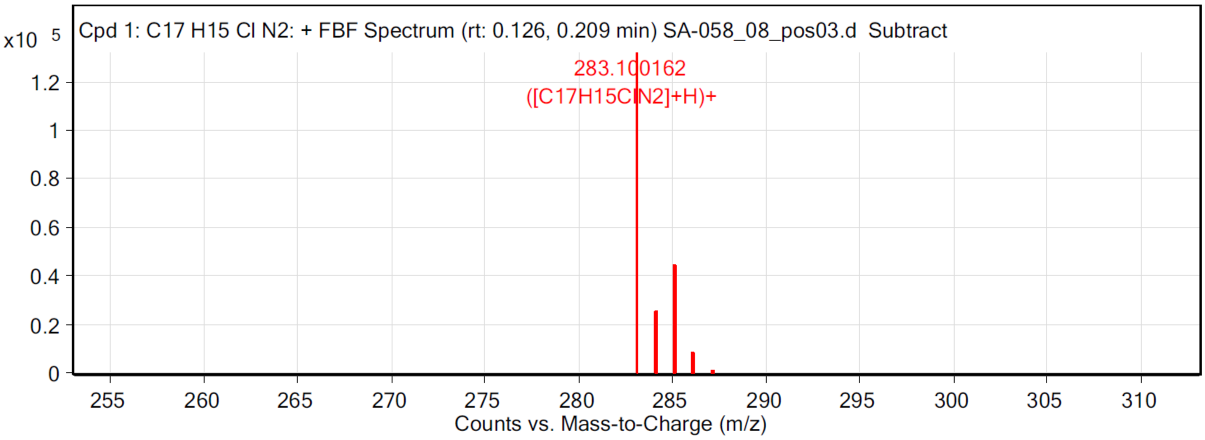

Figure S59. HRMS spectrum of *rac*-**3d**.

rac-1-(3-Bromophenyl)-2,3,4,9-tetrahydro-1*H*-pyrido[3,4-*b*]indole hydrochloride (3e·HCl)

- ^{1}H-NMR spectrum (Figure S60)
- ^{13}C-NMR spectrum (Figure S61)
- MS spectrum (Figure S62)
- HRMS spectrum (Figure S63)

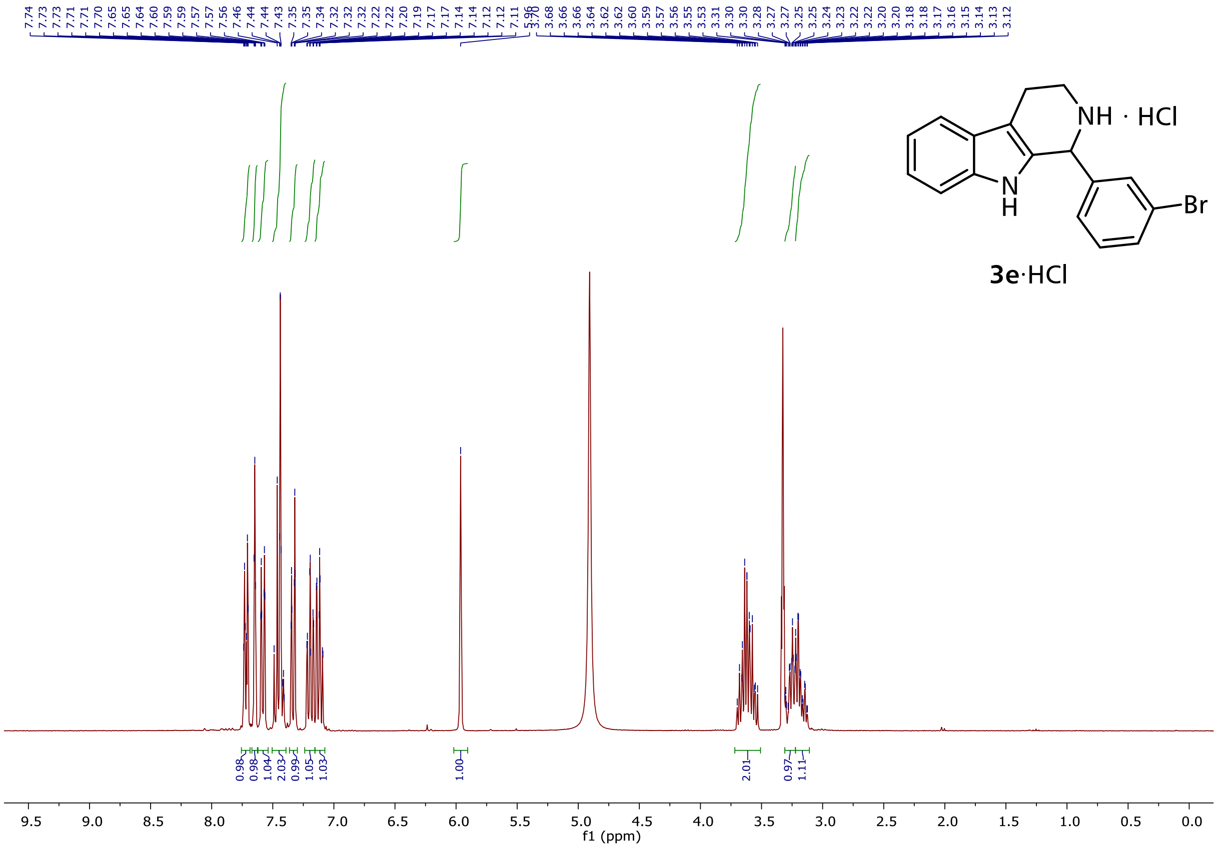

Figure S60. ^{1}H-NMR spectrum of *rac*-**3e**·HCl.

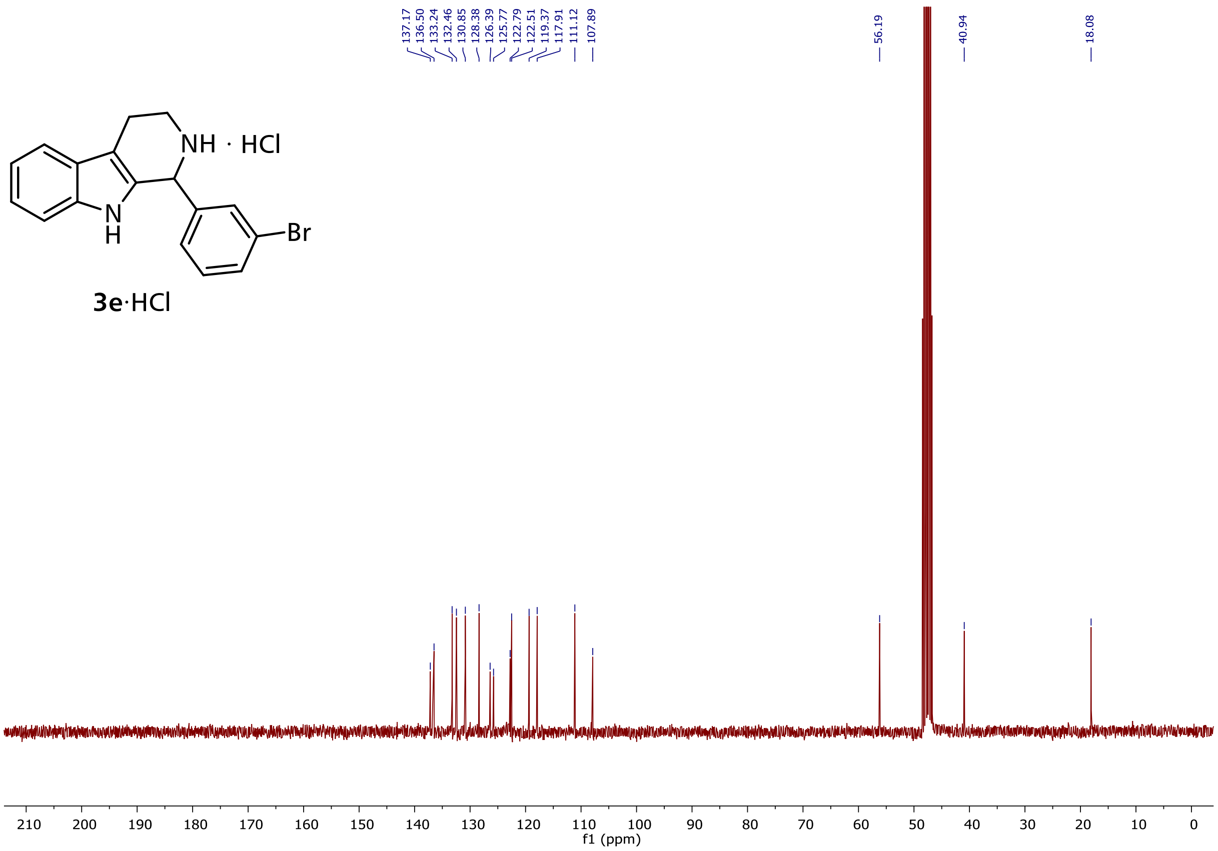

Figure S61. ^{13}C-NMR spectrum of *rac*-**3e**·HCl.

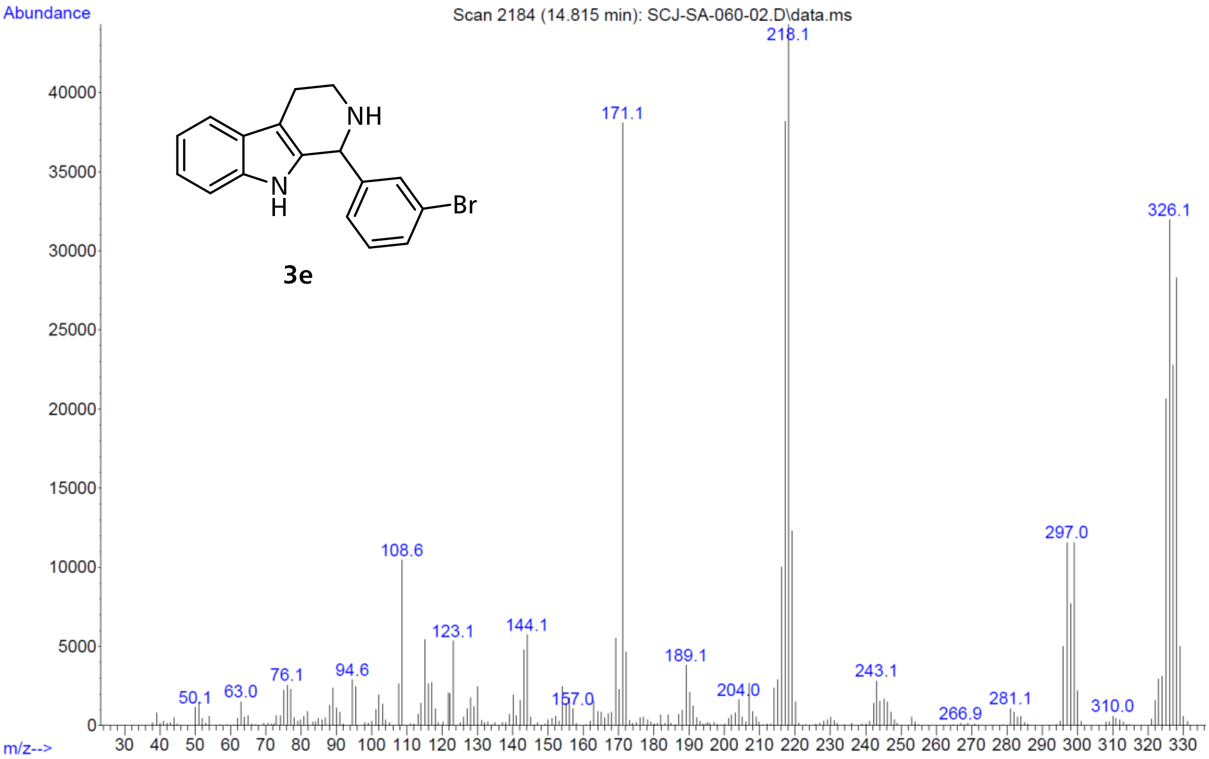

Figure S62. MS spectrum of *rac*-**3e**.

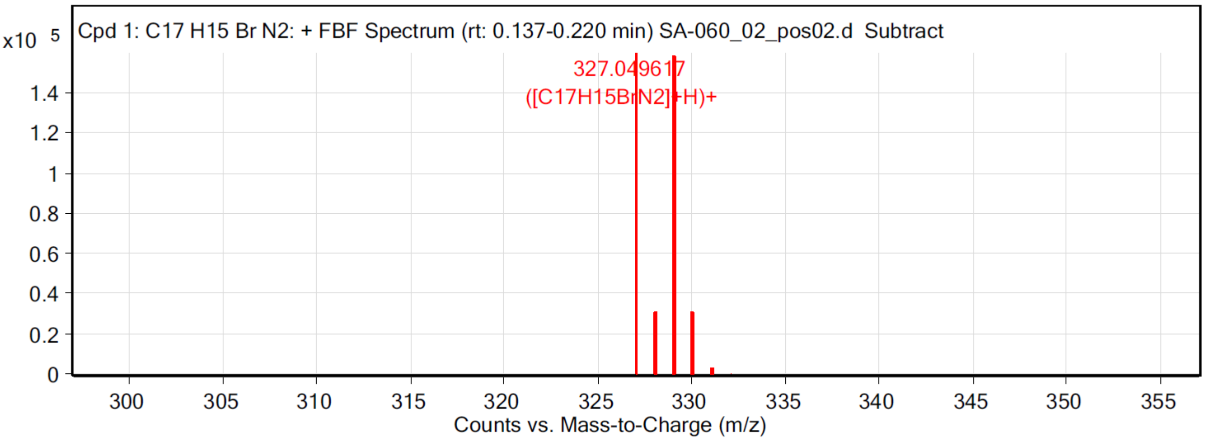

Figure S63. HRMS spectrum of *rac*-**3e**.

rac-1-(3-Methoxyphenyl)-2,3,4,9-tetrahydro-1*H*-pyrido[3,4-*b*]indole hydrochloride (3f·HCl)

- ▶ ^{1}H-NMR spectrum (Figure S64)
- ▶ ^{13}C-NMR spectrum (Figure S65)
- ▶ MS spectrum (Figure S66)
- ▶ HRMS spectrum (Figure S67)

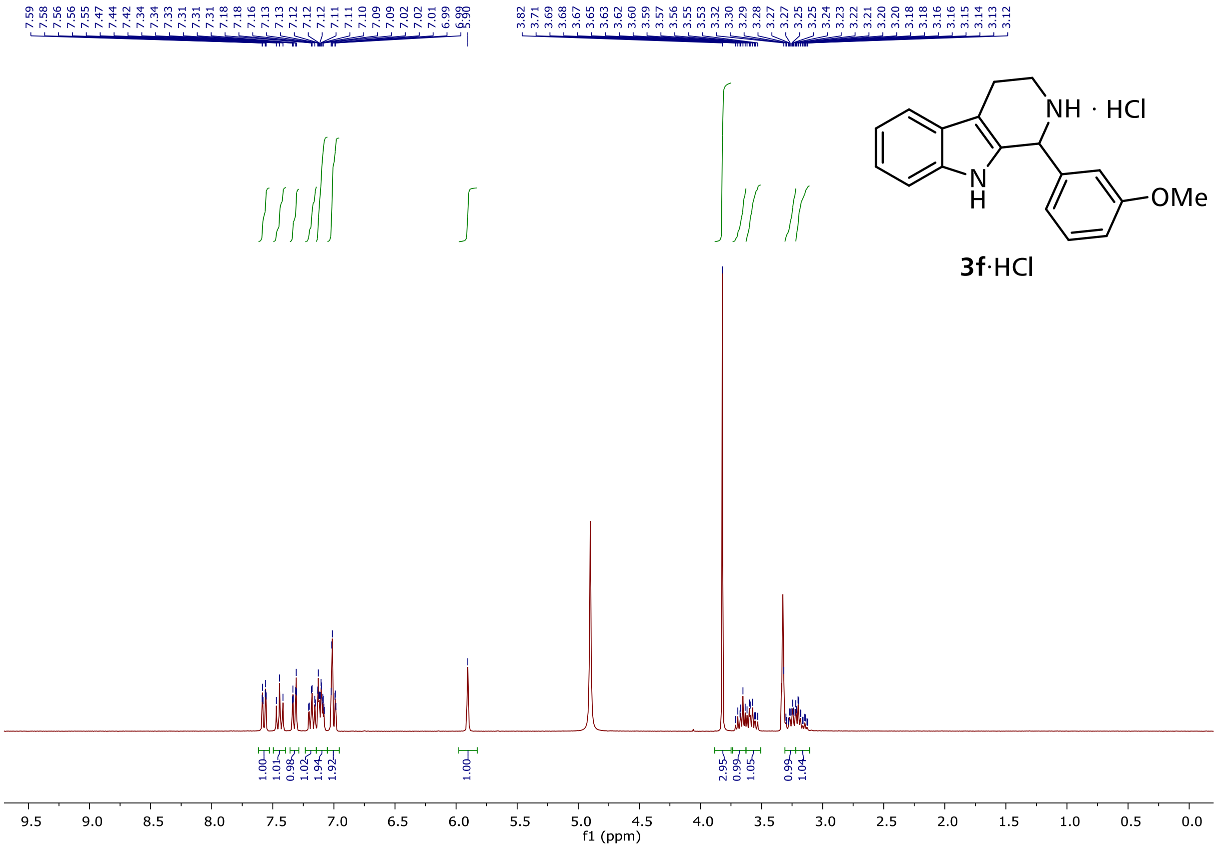

Figure S64. ^{1}H-NMR spectrum of *rac*-**3f**·HCl.

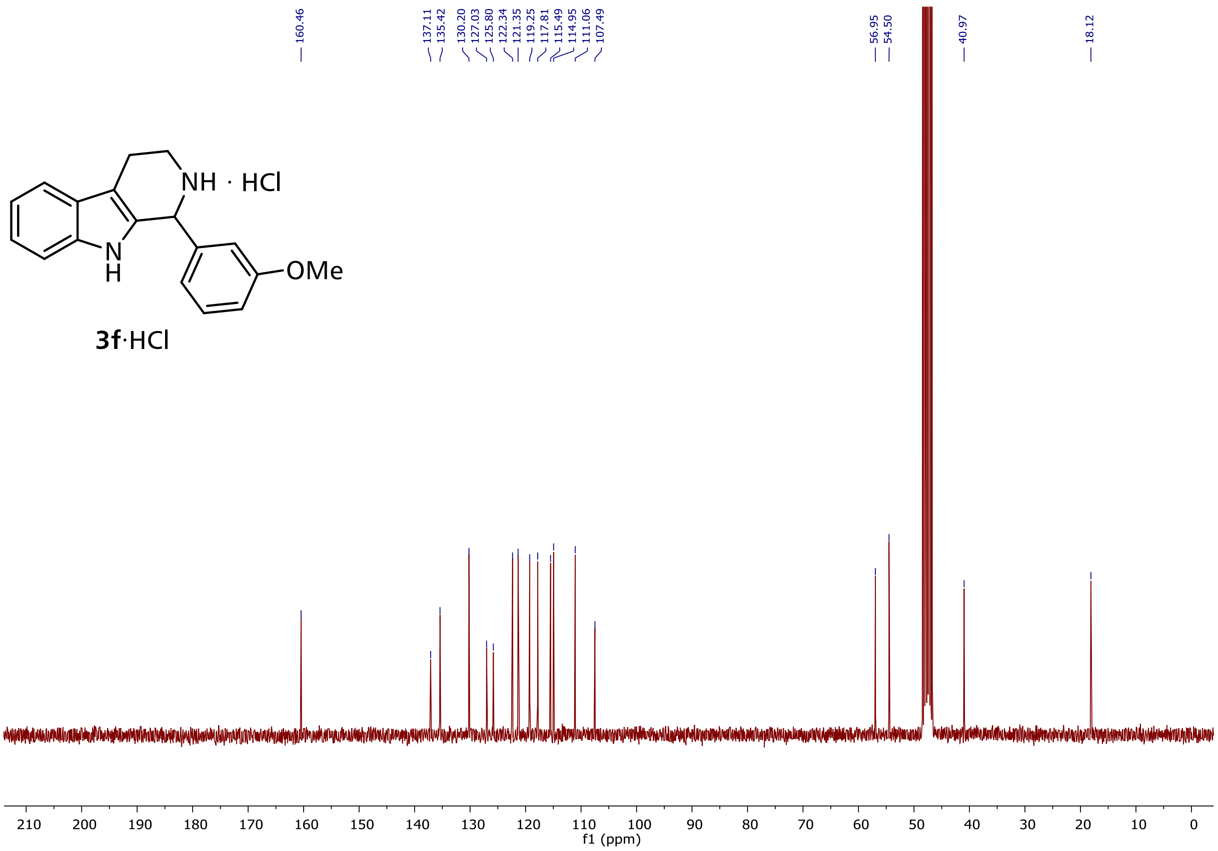

Figure S65. ^{13}C-NMR spectrum of *rac*-**3f**·HCl.

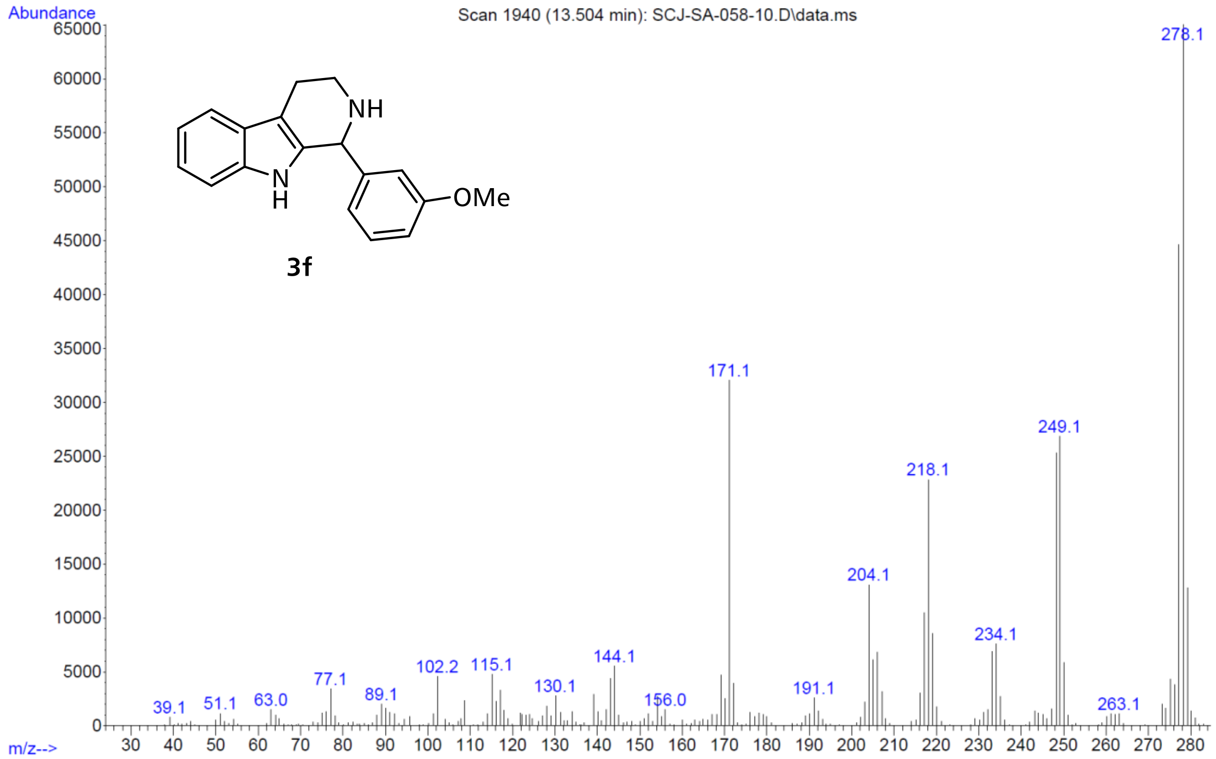

Figure S66. MS spectrum of *rac*-**3f**.

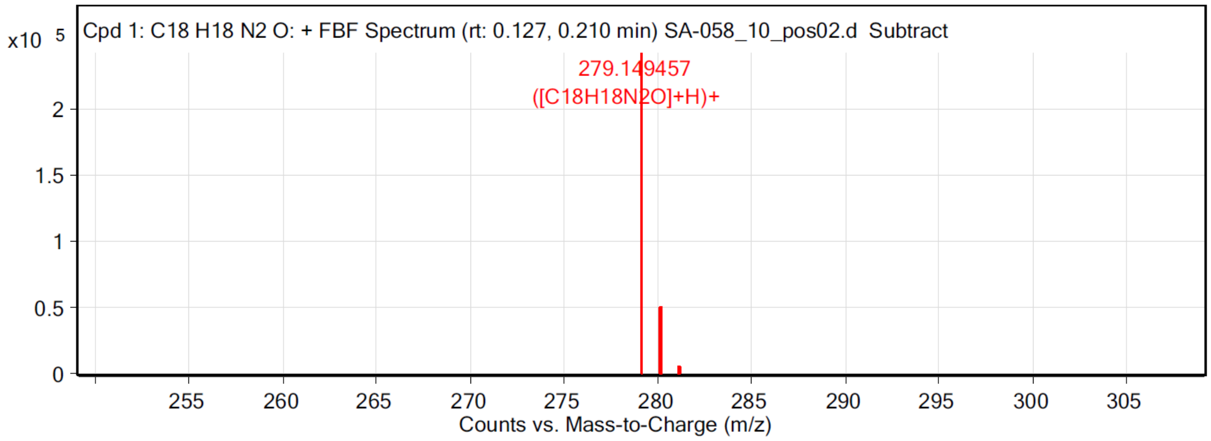

Figure S67. HRMS spectrum of *rac*-**3f**.

rac-1-(3-Nitrophenyl)-2,3,4,9-tetrahydro-1*H*-pyrido[3,4-*b*]indole hydrochloride (3g·HCl)

- ^{1}H-NMR spectrum (Figure S68)
- ^{13}C-NMR spectrum (Figure S69)
- MS spectrum (Figure S70)
- HRMS spectrum (Figure S71)

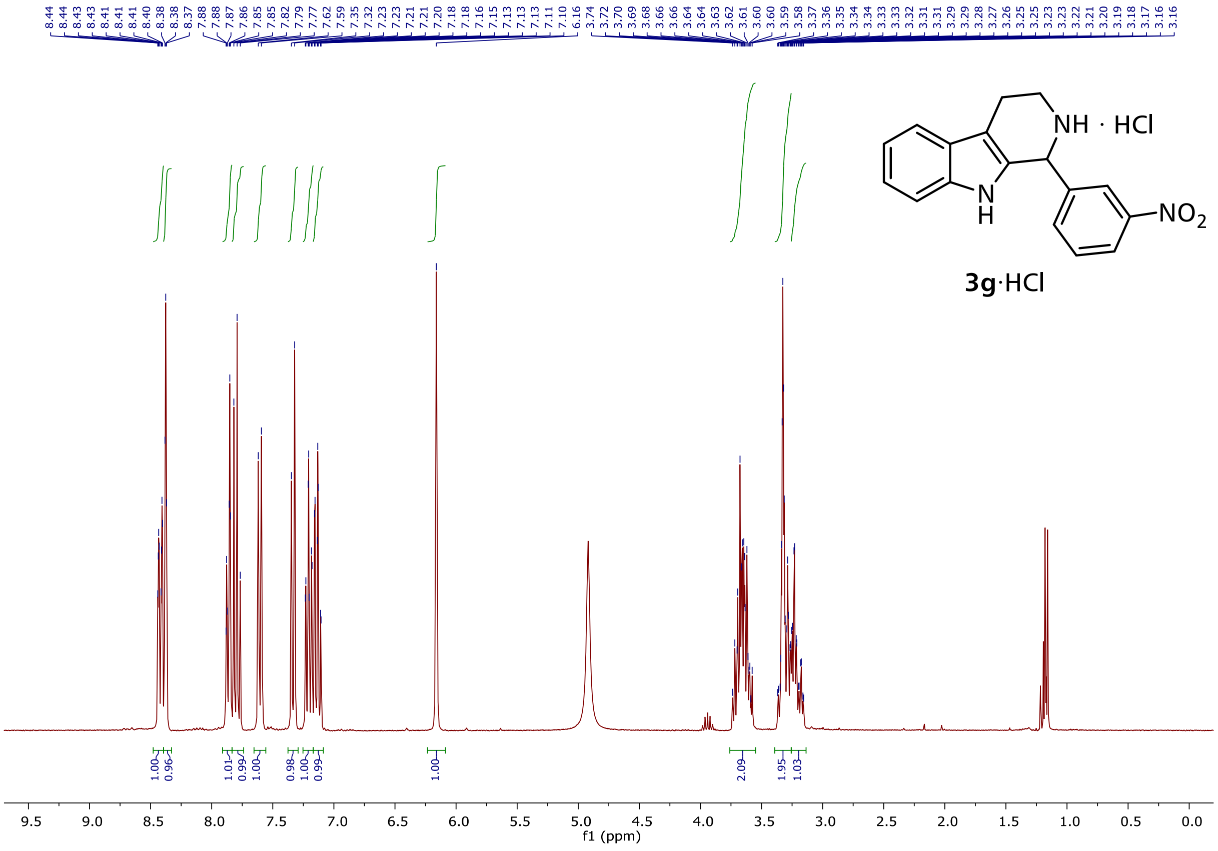

Figure S68. ^{1}H-NMR spectrum of *rac*-**3g**·HCl.

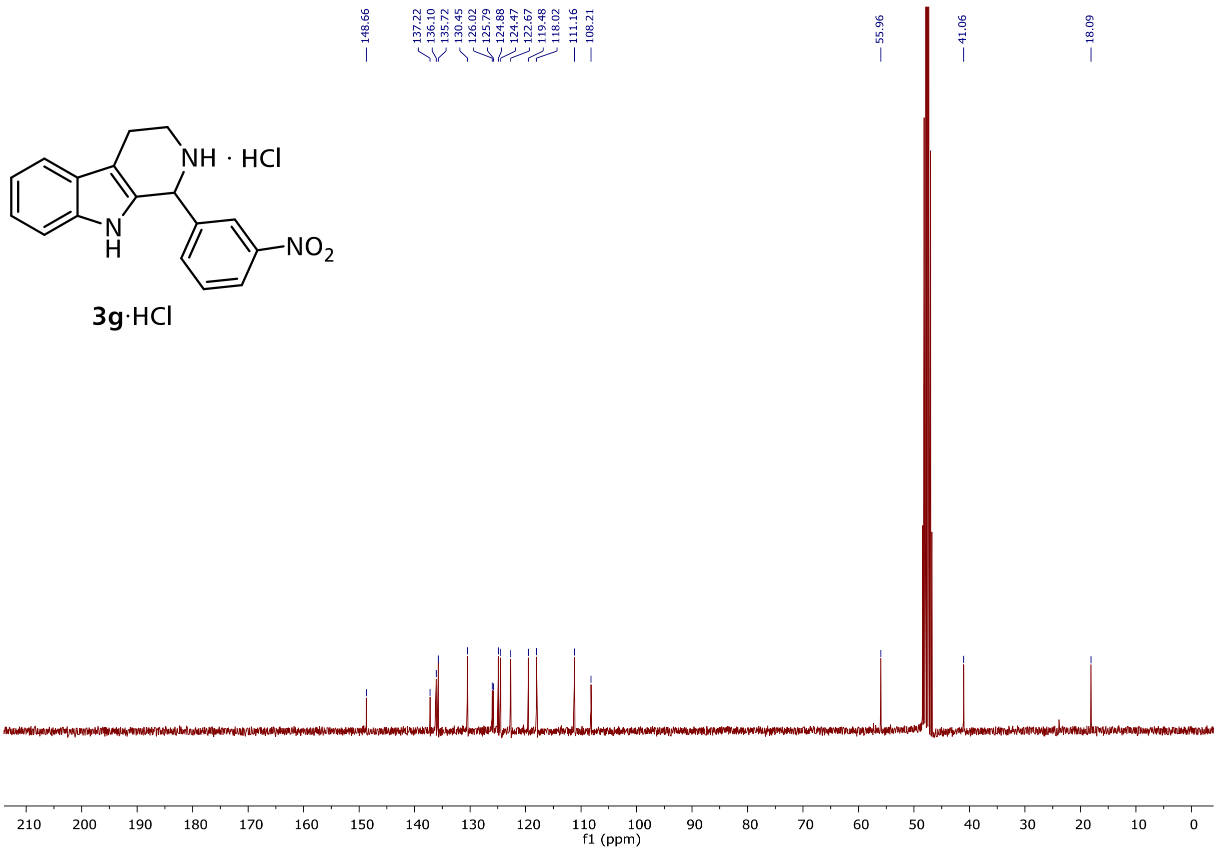

Figure S69. ^{13}C-NMR spectrum of *rac*-**3g**·HCl.

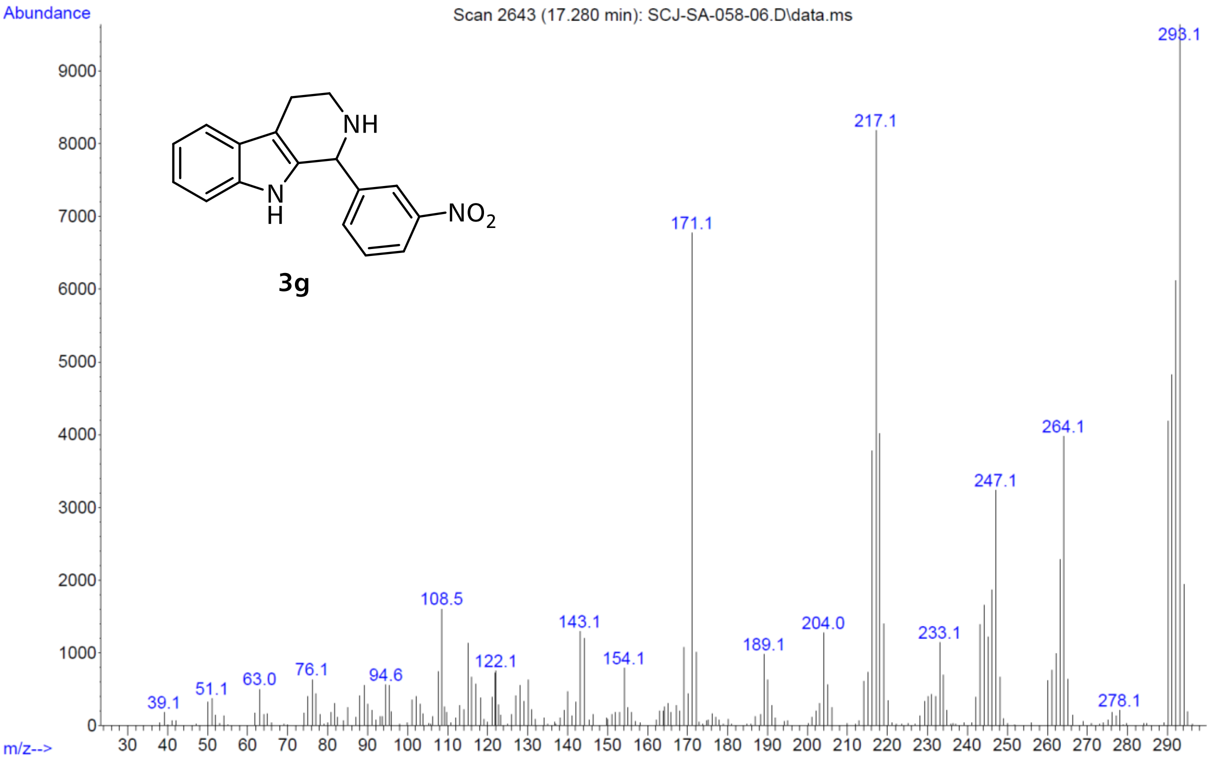

Figure S70. MS spectrum of *rac*-**3g**.

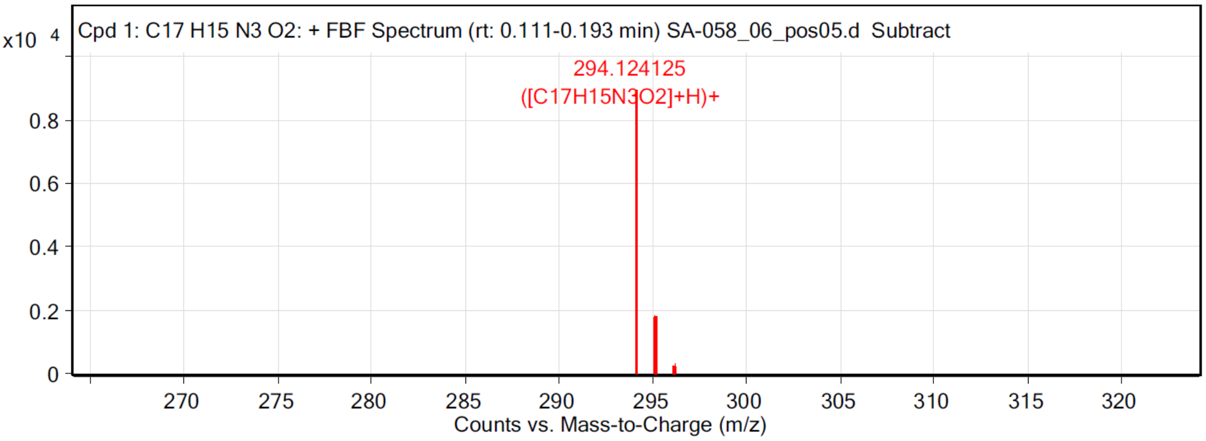

Figure S71. HRMS spectrum of *rac*-**3g**.

rac-1-(2-Fluorophenyl)-2,3,4,9-tetrahydro-1*H*-pyrido[3,4-*b*]indole hydrochloride (3h·HCl)

- ^{1}H-NMR spectrum (Figure S72)
- ^{13}C-NMR spectrum (Figure S73)
- MS spectrum (Figure S74)
- HRMS spectrum (Figure S75)

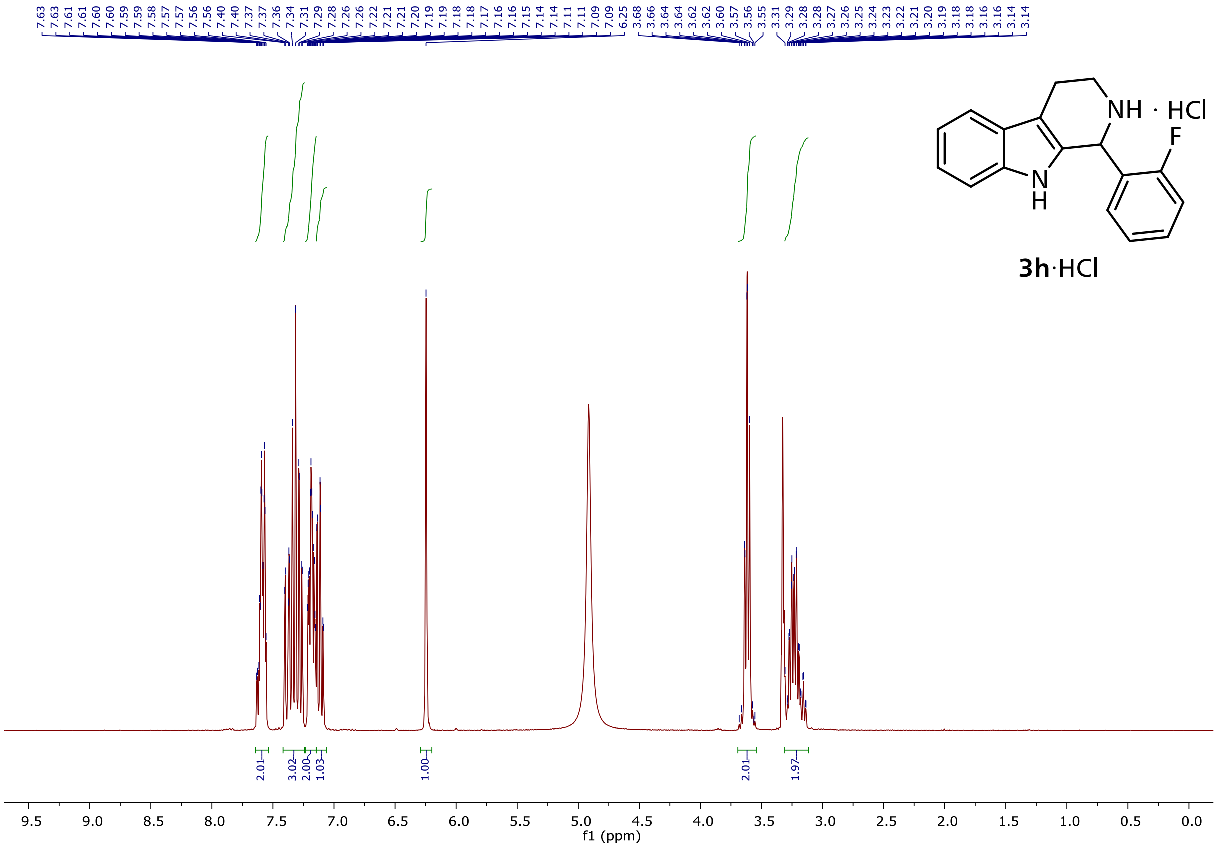

Figure S72. ^{1}H-NMR spectrum of *rac*-**3h**·HCl.

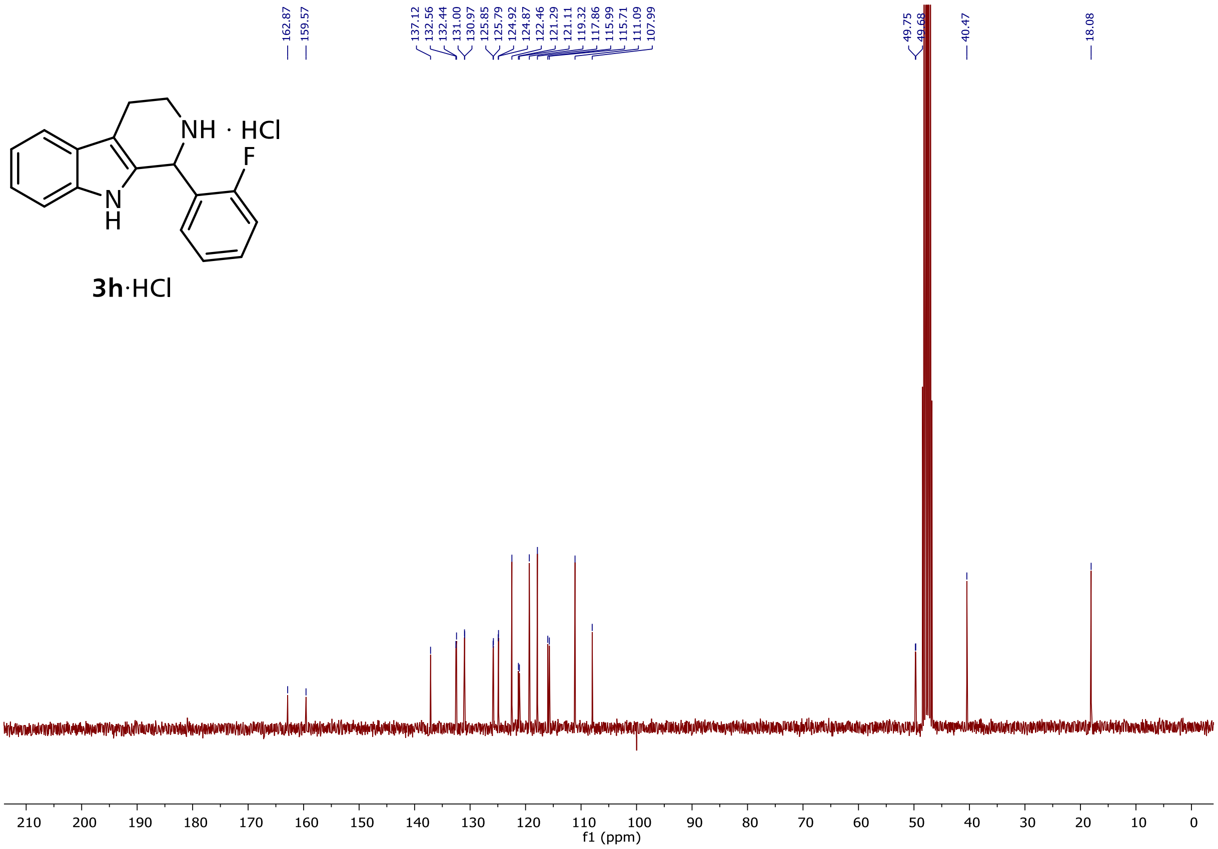

Figure S73. ^{13}C-NMR spectrum of *rac*-**3h**·HCl.

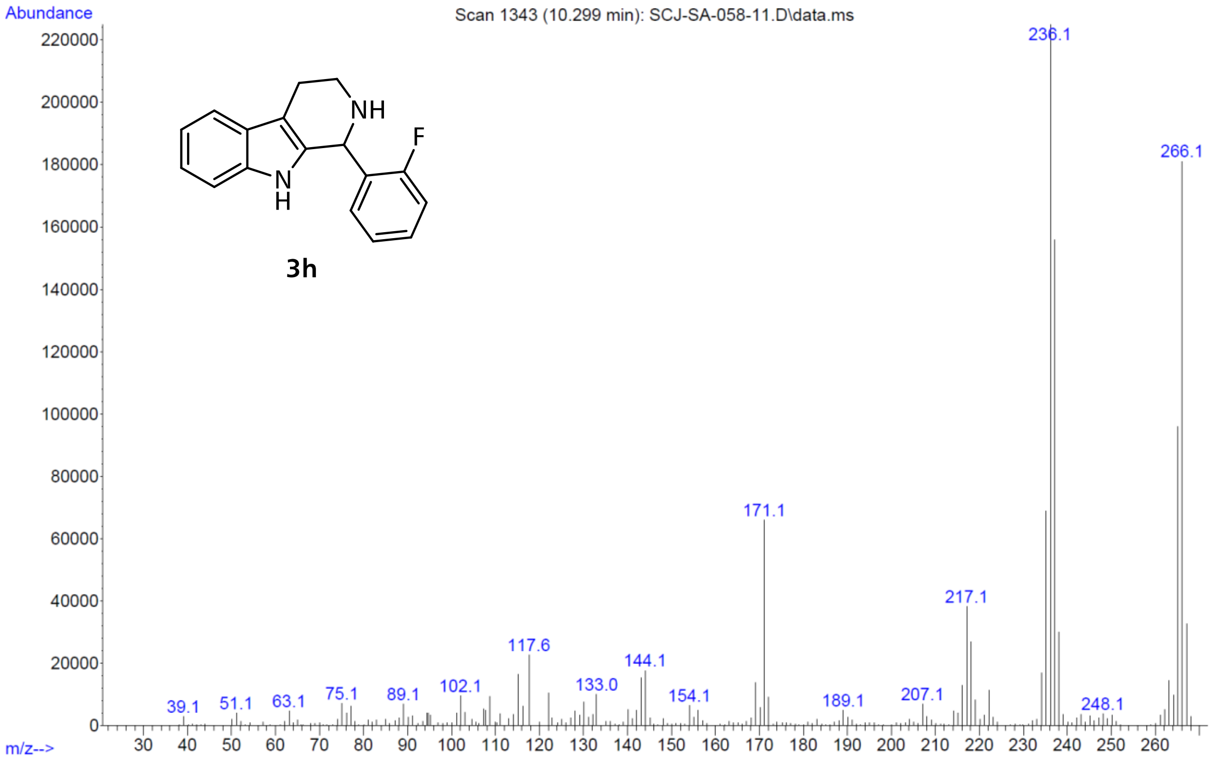

Figure S74. MS spectrum of *rac*-**3h**.

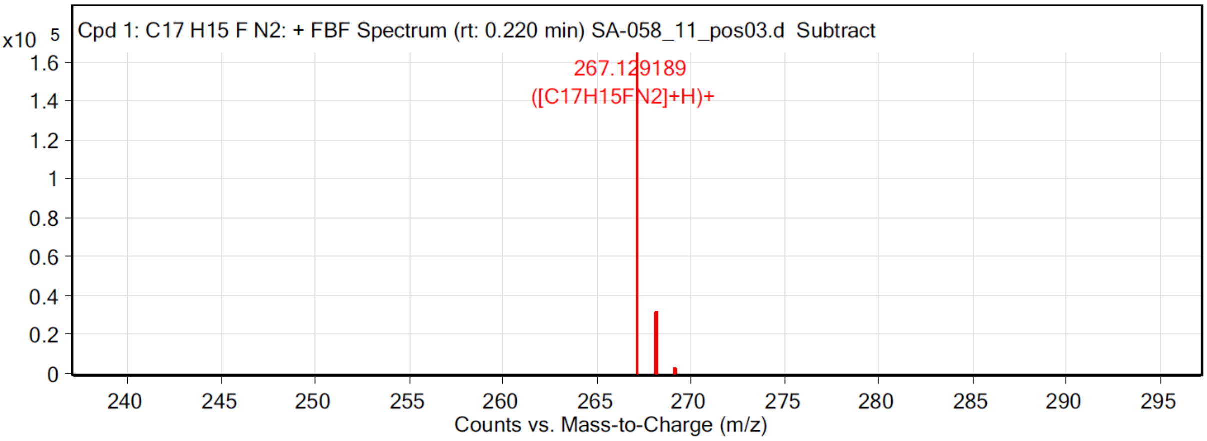

Figure S75. HRMS spectrum of *rac*-**3h**.

rac-1-(2-Bromophenyl)-2,3,4,9-tetrahydro-1*H*-pyrido[3,4-*b*]indole hydrochloride (3i·HCl)

- ^{1}H-NMR spectrum (Figure S76)
- ^{13}C-NMR spectrum (Figure S77)
- MS spectrum (Figure S78)
- HRMS spectrum (Figure S79)

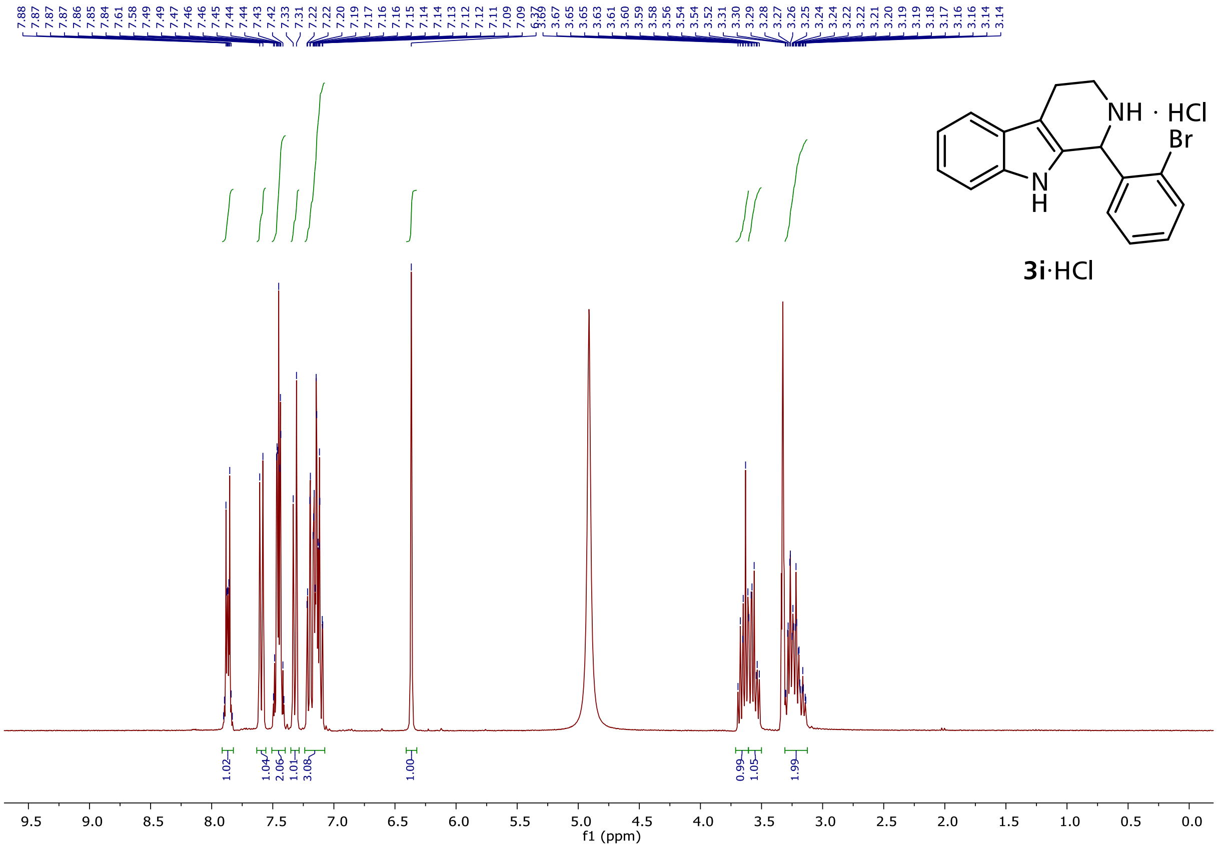

Figure S76. ^{1}H-NMR spectrum of *rac*-**3i**·HCl.

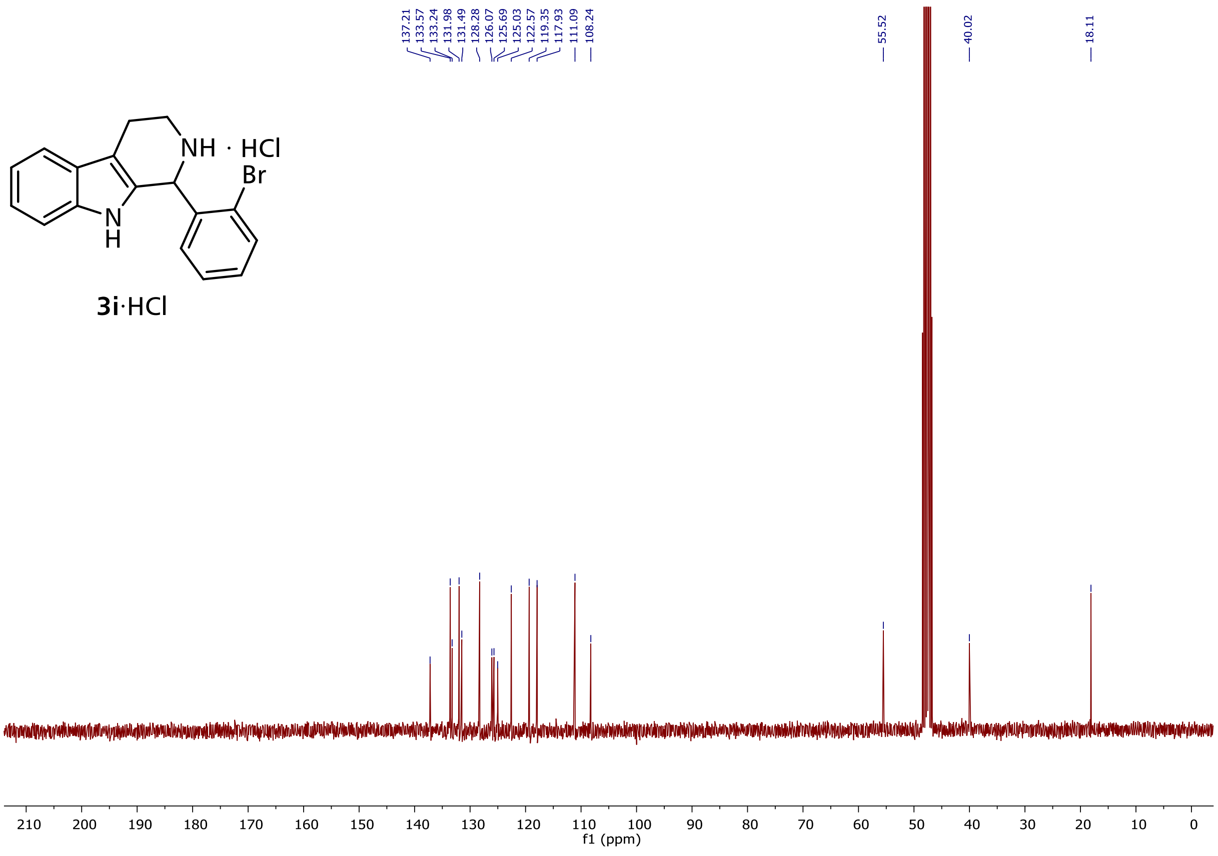

Figure S77. ^{13}C-NMR spectrum of *rac*-**3i**·HCl.

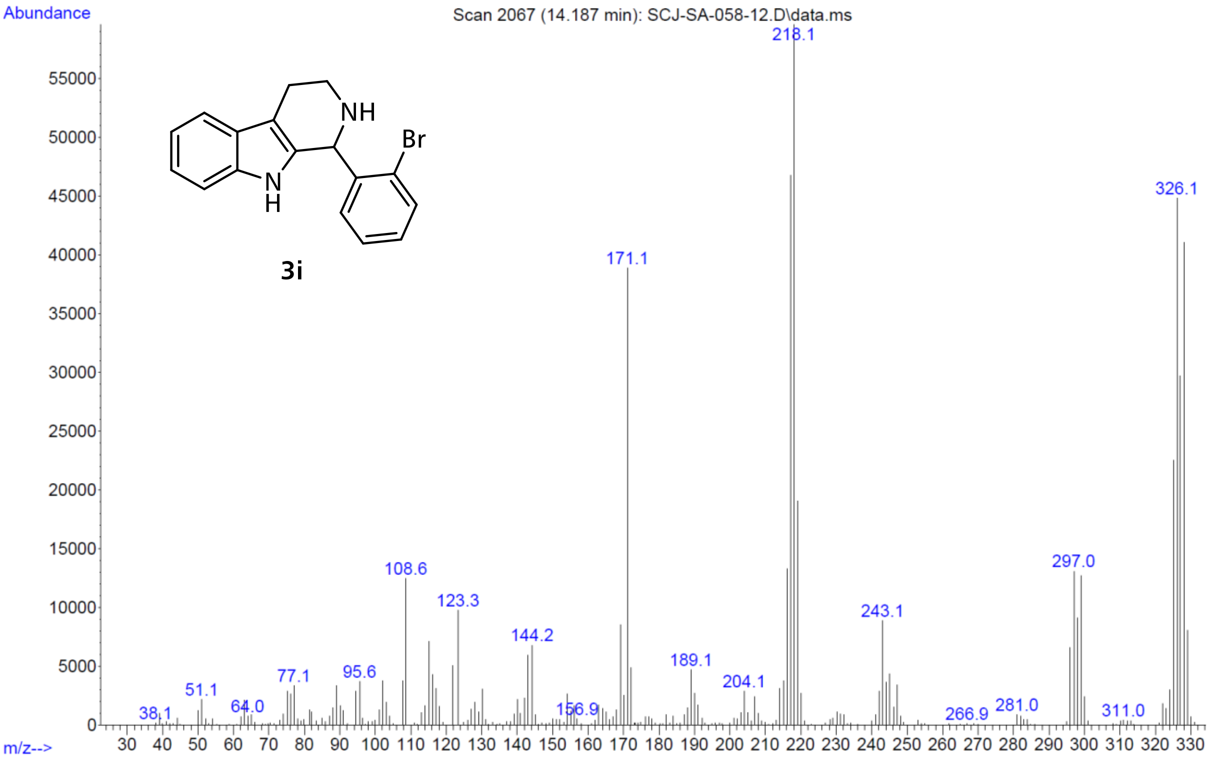

Figure S78. MS spectrum of *rac*-**3i**.

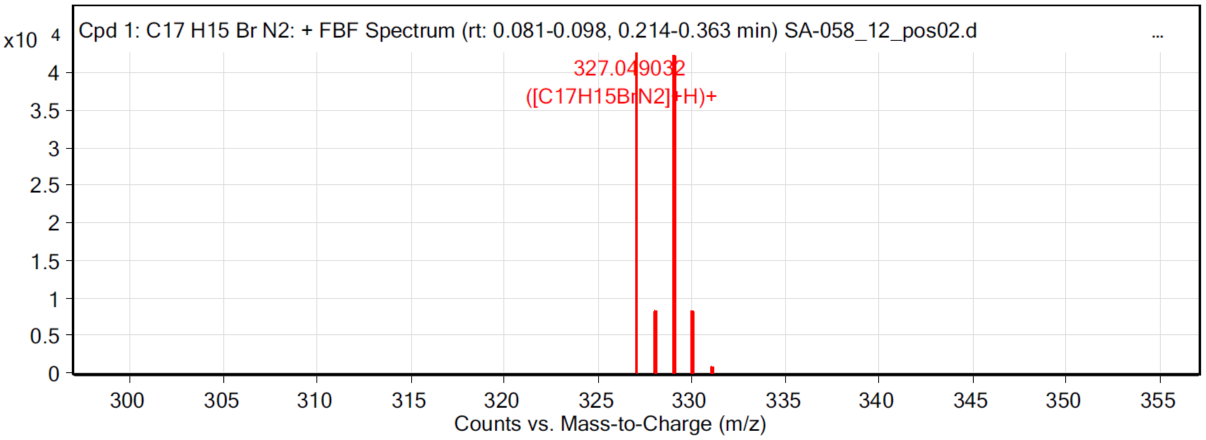

Figure S79. HRMS spectrum of *rac*-**3i**.

rac-1-(4-Chlorophenyl)-2,3,4,9-tetrahydro-1*H*-pyrido[3,4-*b*]indole hydrochloride (3j·HCl)

- ^{1}H-NMR spectrum (Figure S80)
- ^{13}C-NMR spectrum (Figure S81)
- MS spectrum (Figure S82)
- HRMS spectrum (Figure S83)

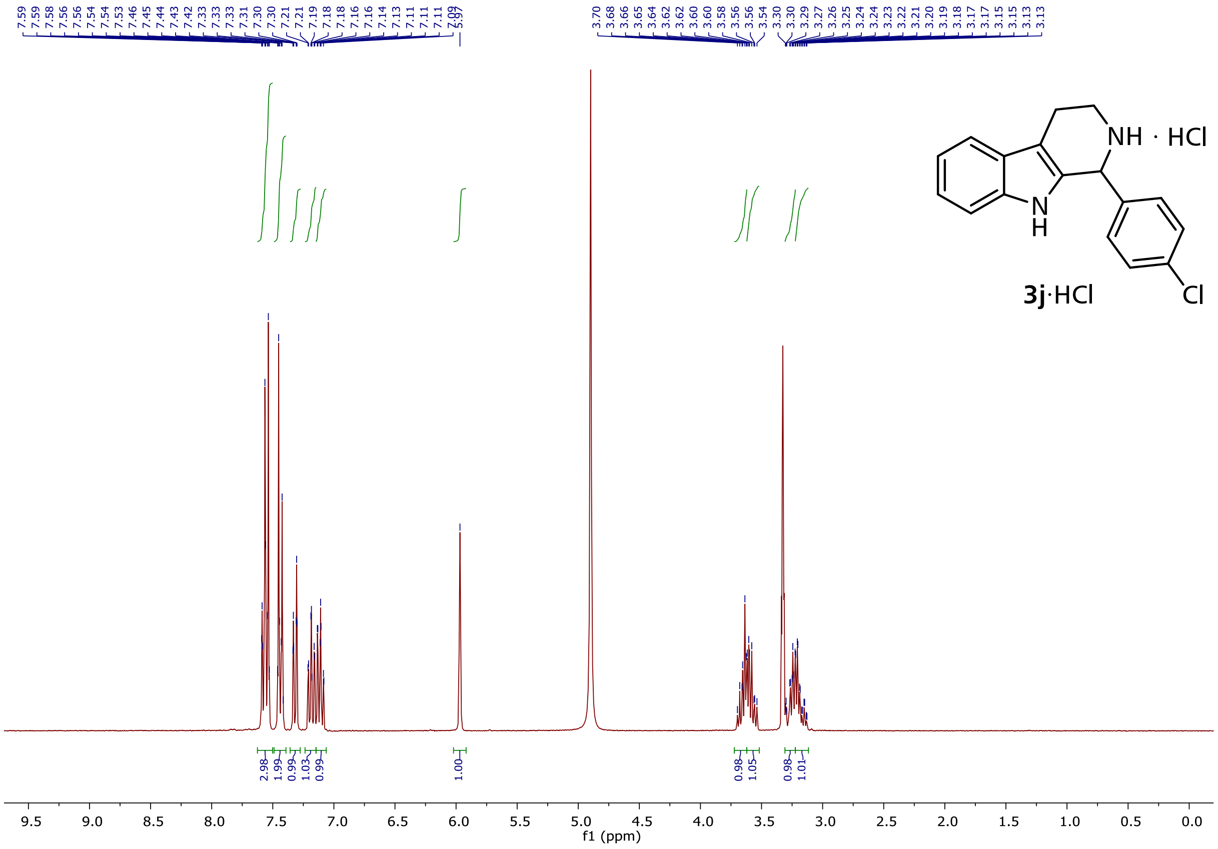

Figure S80. ^{1}H-NMR spectrum of *rac*-**3j**·HCl.

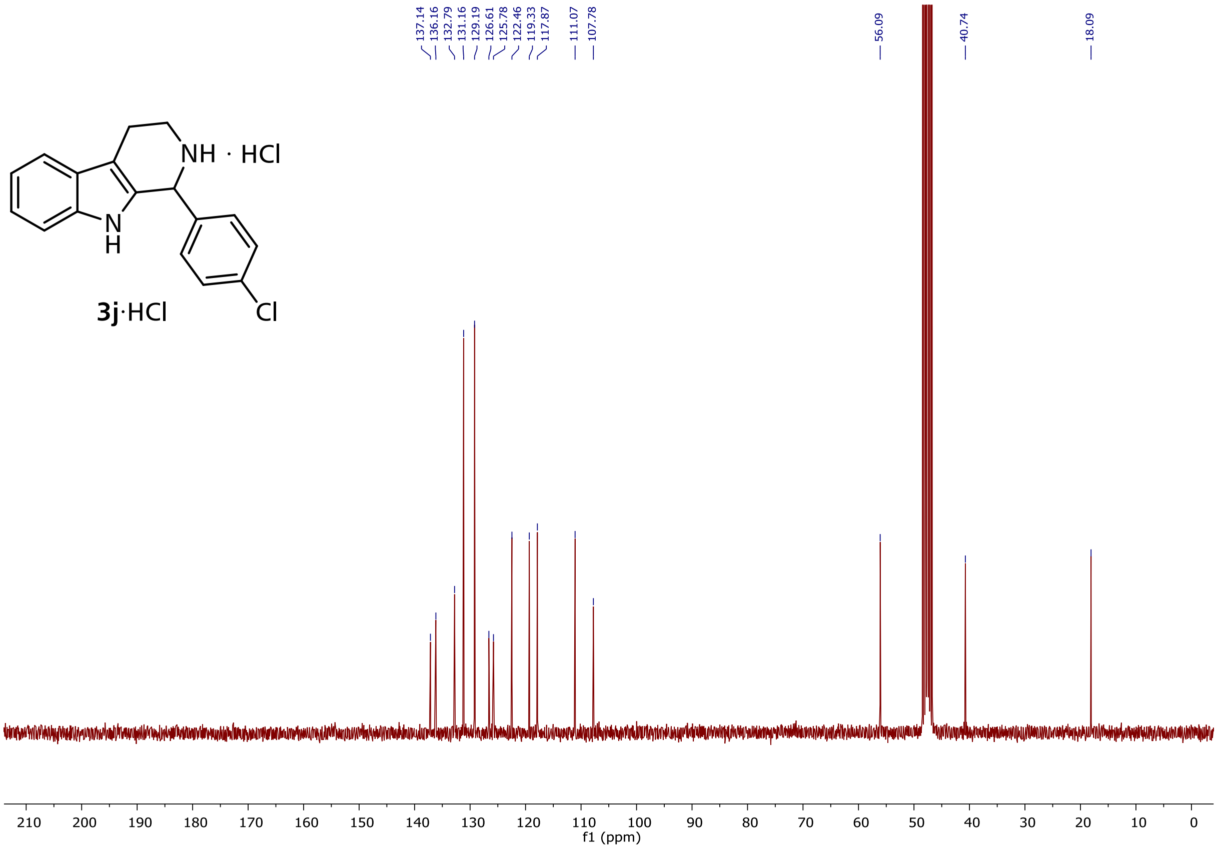

Figure S81. ^{13}C-NMR spectrum of *rac*-**3j**·HCl.

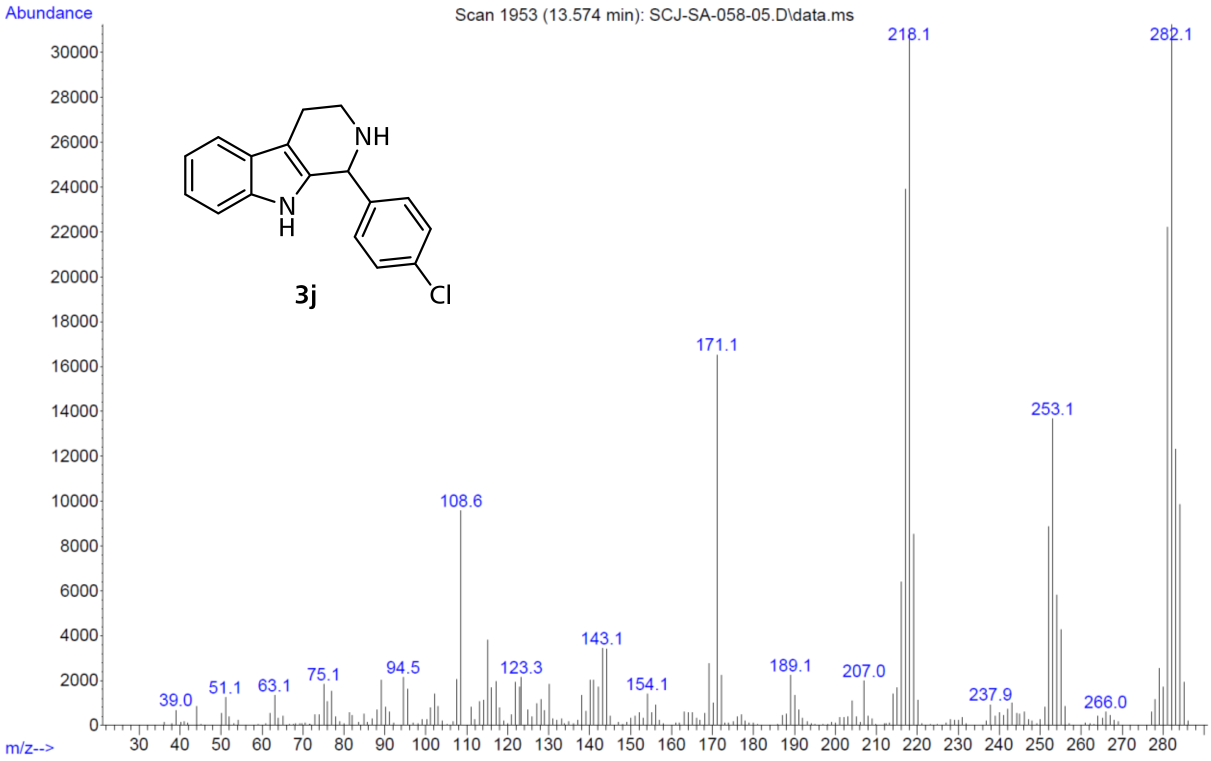

Figure S82. MS spectrum of *rac*-**3j**.

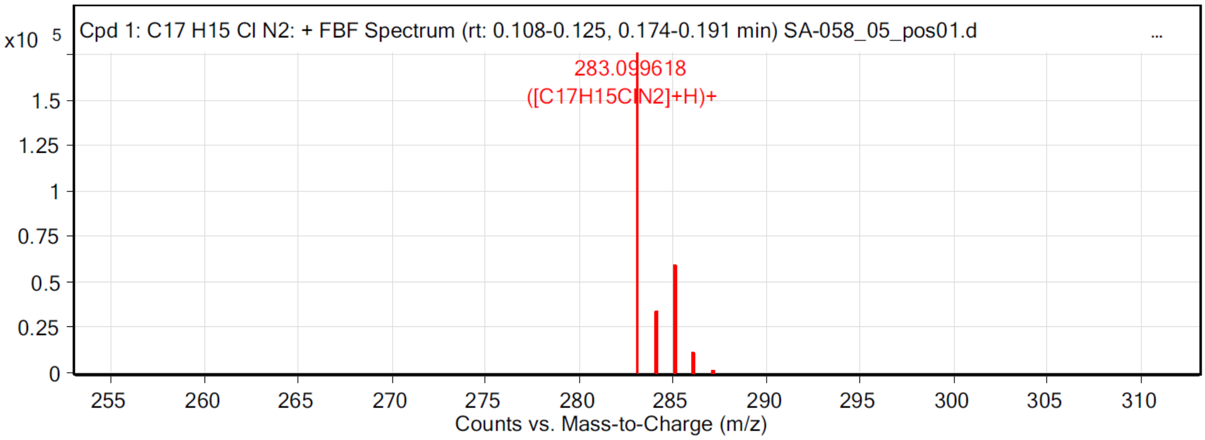

Figure S83. HRMS spectrum of *rac*-**3j**.

rac-1-(4-Methoxyphenyl)-2,3,4,9-tetrahydro-1*H*-pyrido[3,4-*b*]indole hydrochloride (3k·HCl)

- ^{1}H-NMR spectrum (Figure S84)
- ^{13}C-NMR spectrum (Figure S85)
- MS spectrum (Figure S86)
- HRMS spectrum (Figure S87)

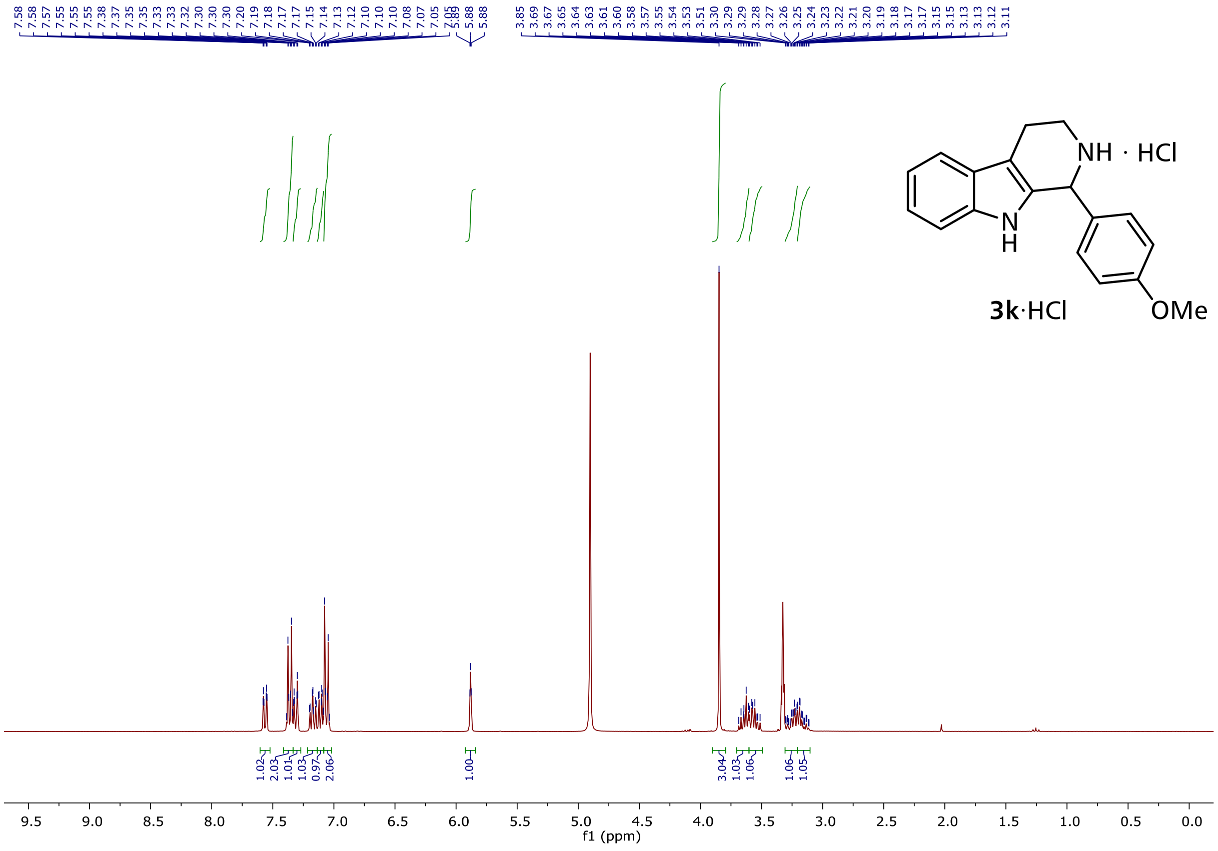

Figure S84. ^{1}H-NMR spectrum of *rac*-**3k**·HCl.

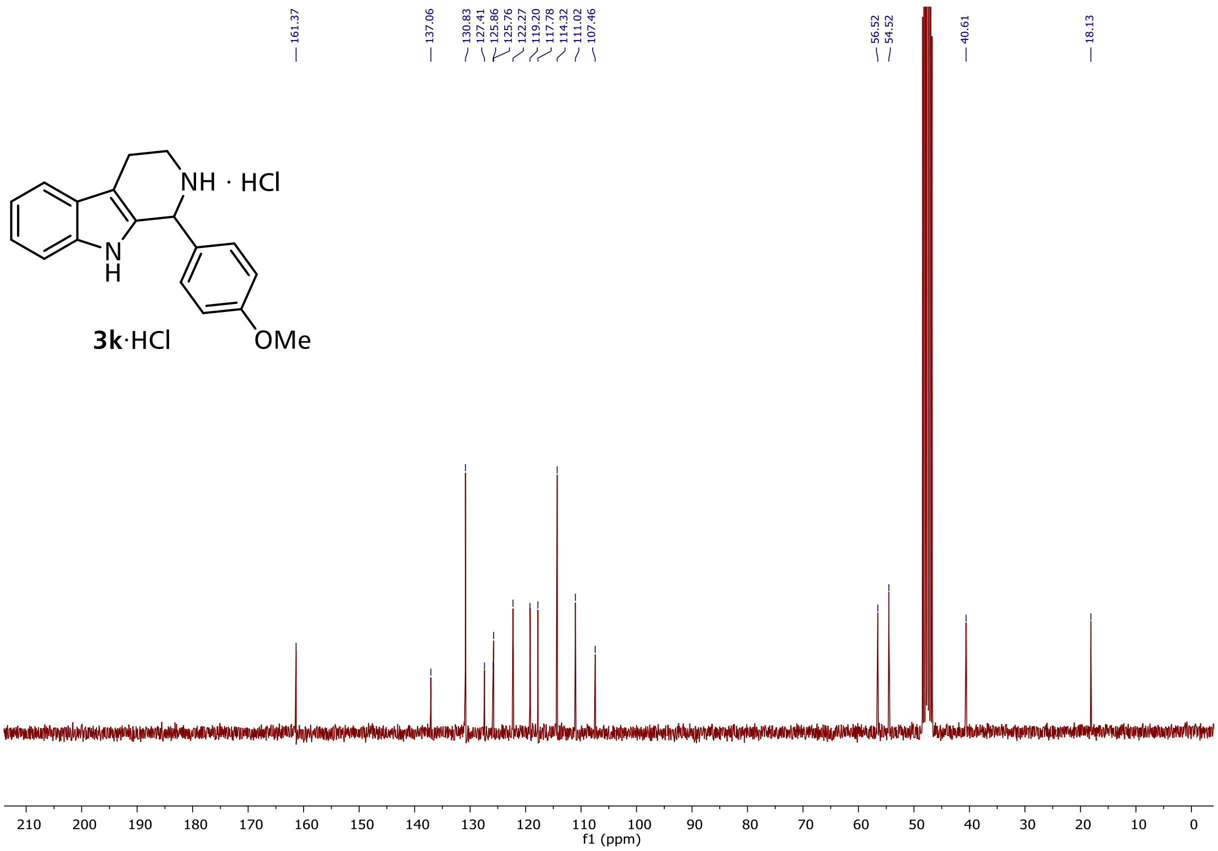

Figure S85. ^{13}C-NMR spectrum of *rac*-**3k**·HCl.

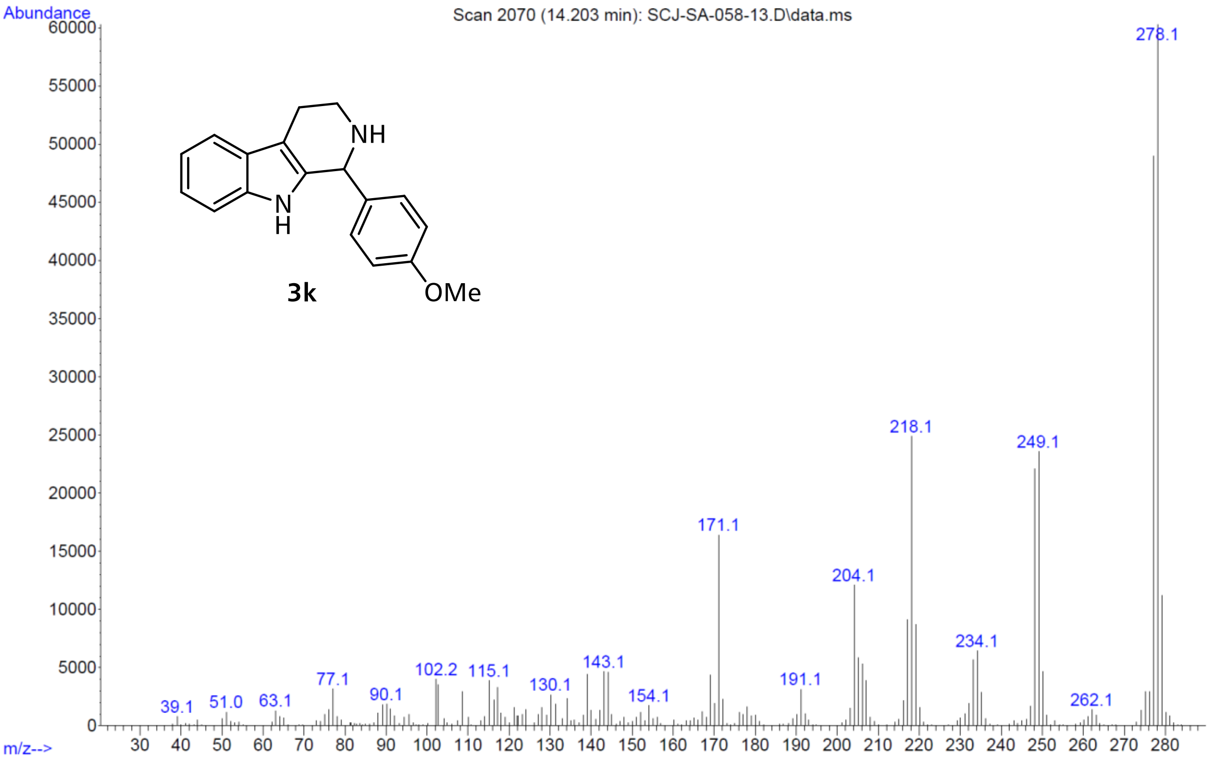

Figure S86. MS spectrum of *rac*-**3k**.

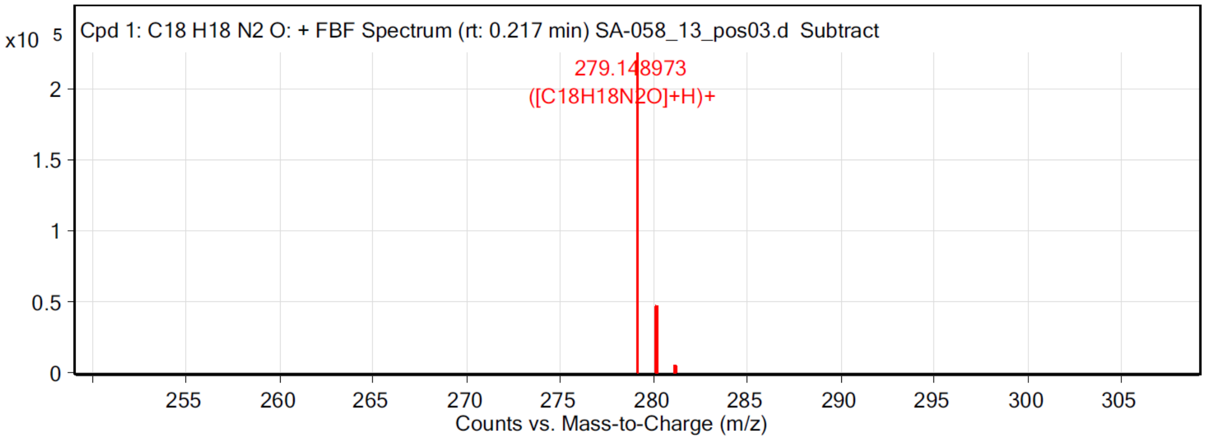

Figure S87. HRMS spectrum of *rac*-**3k**.

rac-1-(4-Nitrophenyl)-2,3,4,9-tetrahydro-1*H*-pyrido[3,4-*b*]indole hydrochloride (3l·HCl)

- ▶ ^{1}H-NMR spectrum (Figure S88)
- ▶ ^{13}C-NMR spectrum (Figure S89)
- ▶ MS spectrum (Figure S90)
- ▶ HRMS spectrum (Figure S91)

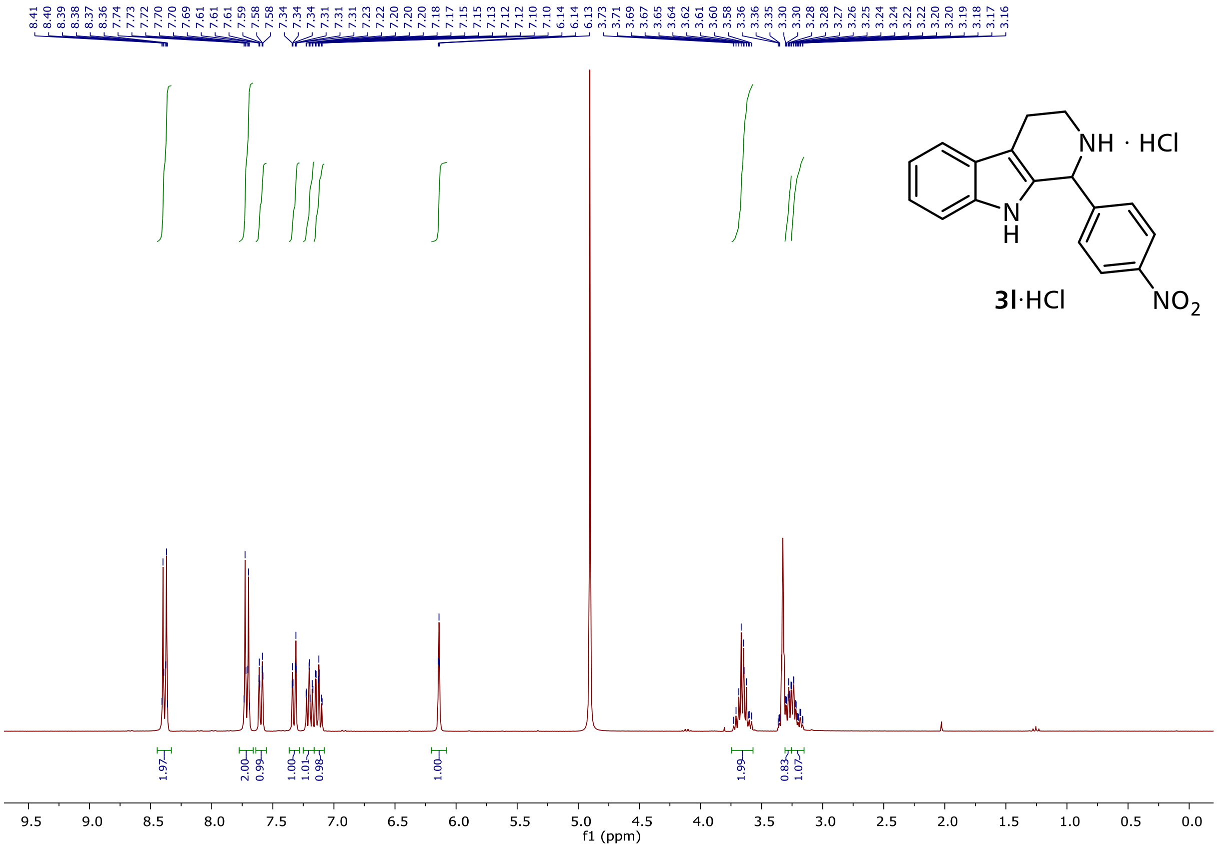

Figure S88. ^{1}H-NMR spectrum of *rac*-**3l**·HCl.

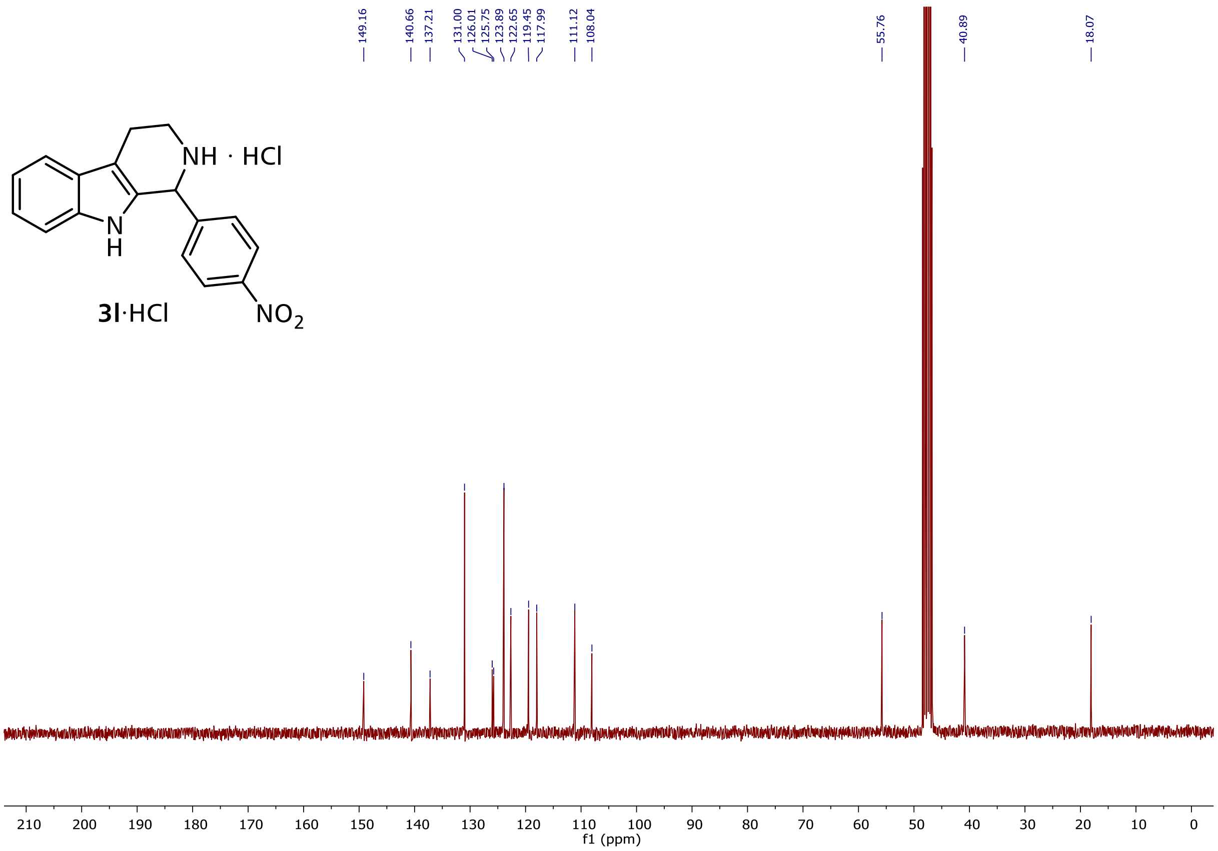

Figure S89. ^{13}C-NMR spectrum of *rac*-**3l**·HCl.

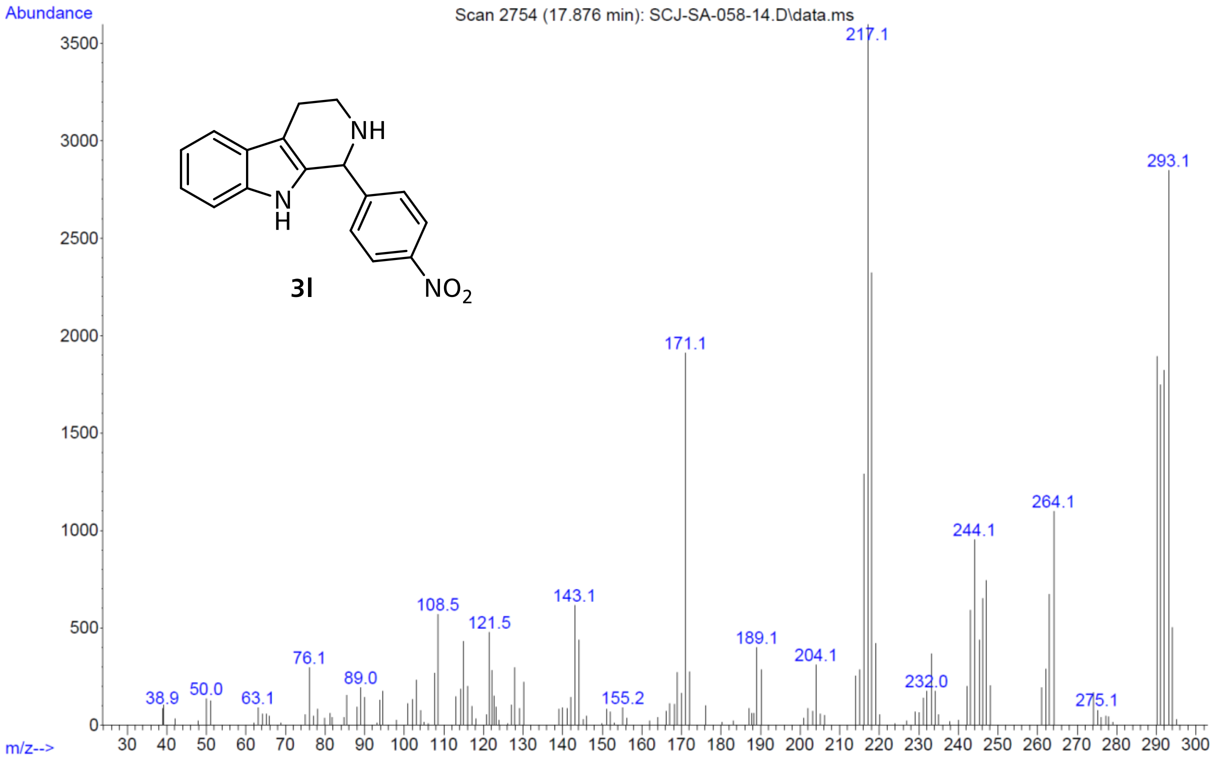

Figure S90. MS spectrum of *rac*-**3l**.

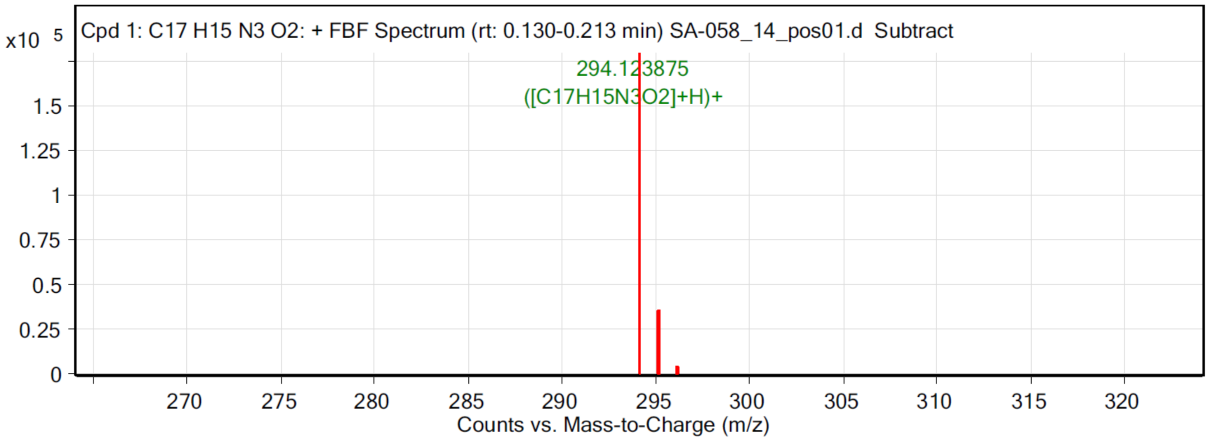

Figure S91. HRMS spectrum of *rac*-**3l**.

(*R*)-1-Phenyl-2,3,4,9-tetrahydro-1*H*-pyrido[3,4-*b*]indole (3a)

- ▶ ^{1}H-NMR spectrum (Figure S92)
- ▶ ^{13}C-NMR spectrum (Figure S93)
- ▶ DEPT135-NMR spectrum (Figure S94)
- ▶ DEPT90-NMR spectrum (Figure S95)
- ▶ COSY-NMR spectrum (Figure S96)
- ▶ HSQC-NMR spectrum (Figure S97)
- ▶ MS spectrum (Figure S98)
- ▶ HRMS spectrum (Figure S99)

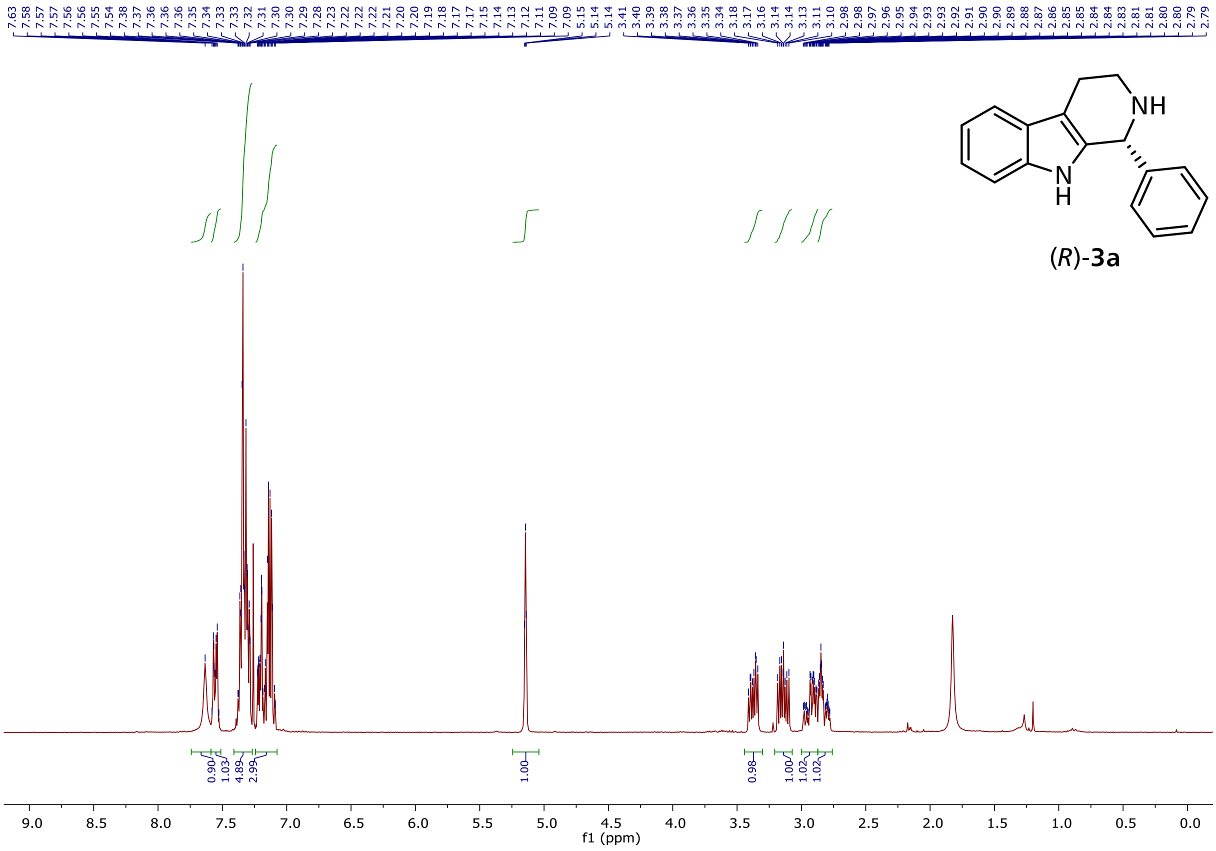

Figure S92. ^{1}H-NMR spectrum of (*R*)-**3a**.

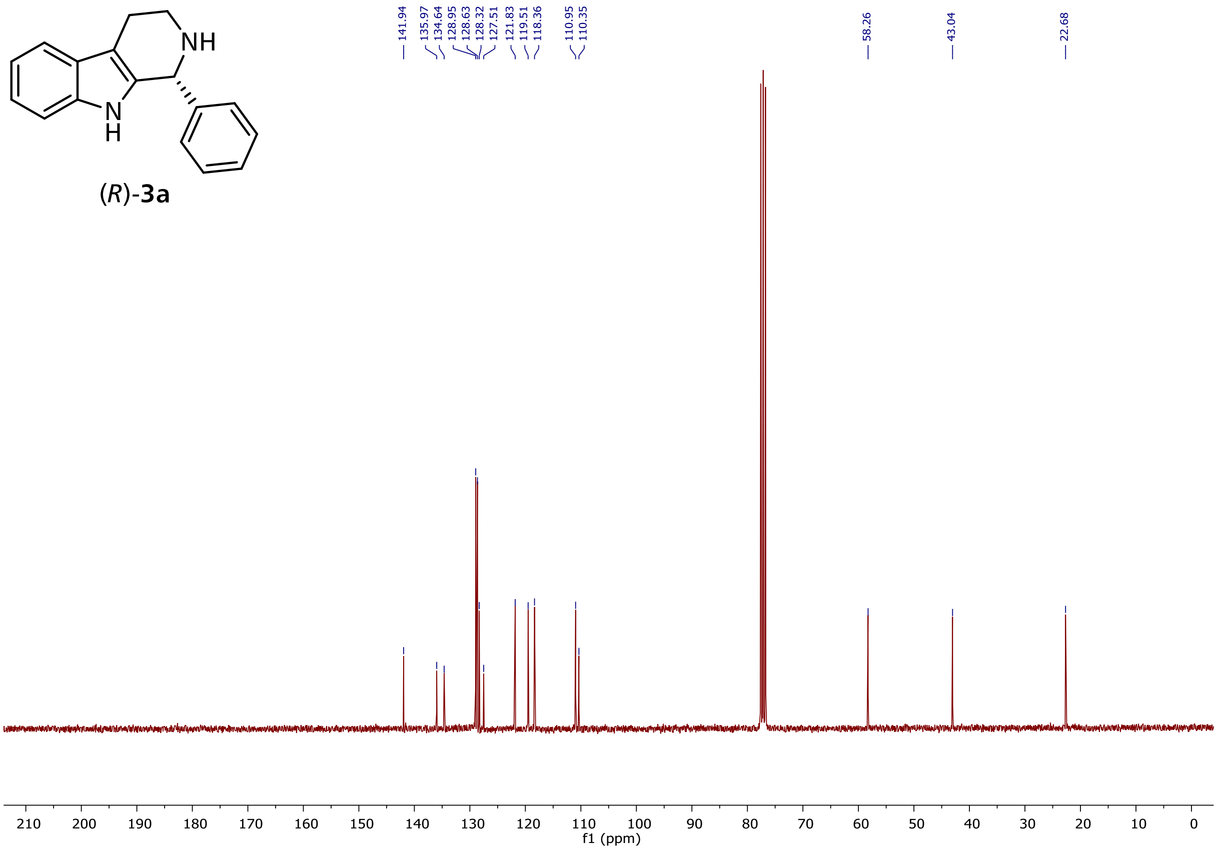

Figure S93. ^{13}C-NMR spectrum of (*R*)-**3a**.

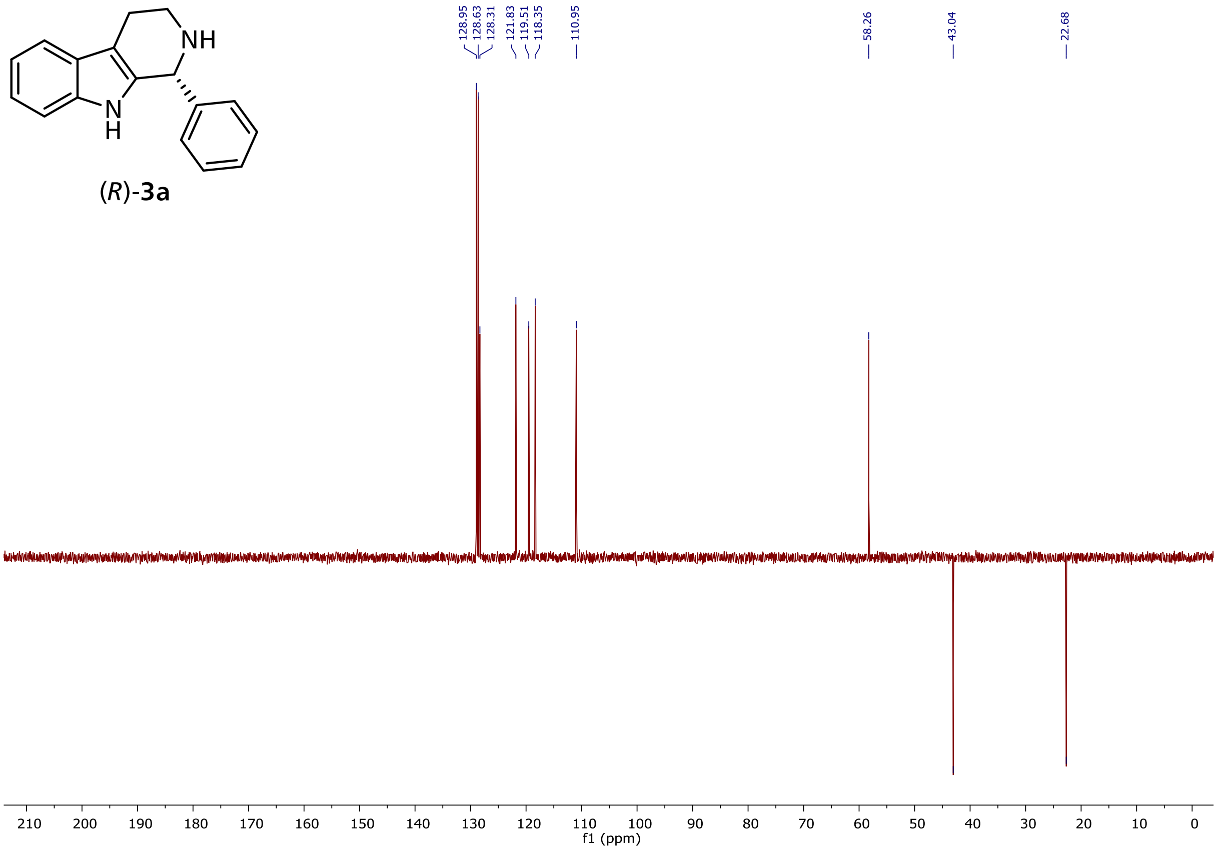

Figure S94. DEPT135-NMR spectrum of (*R*)-**3a**.

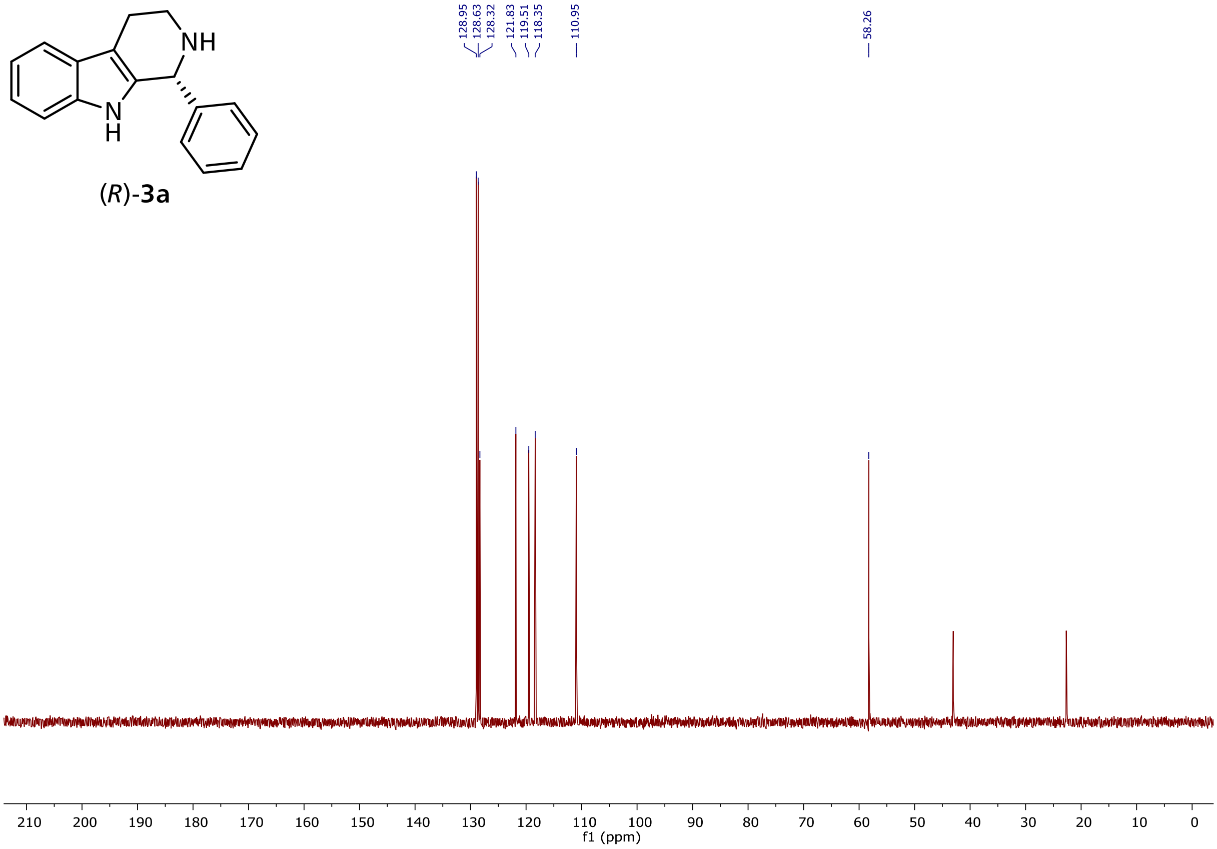

Figure S95. DEPT90-NMR spectrum of (*R*)-**3a**.

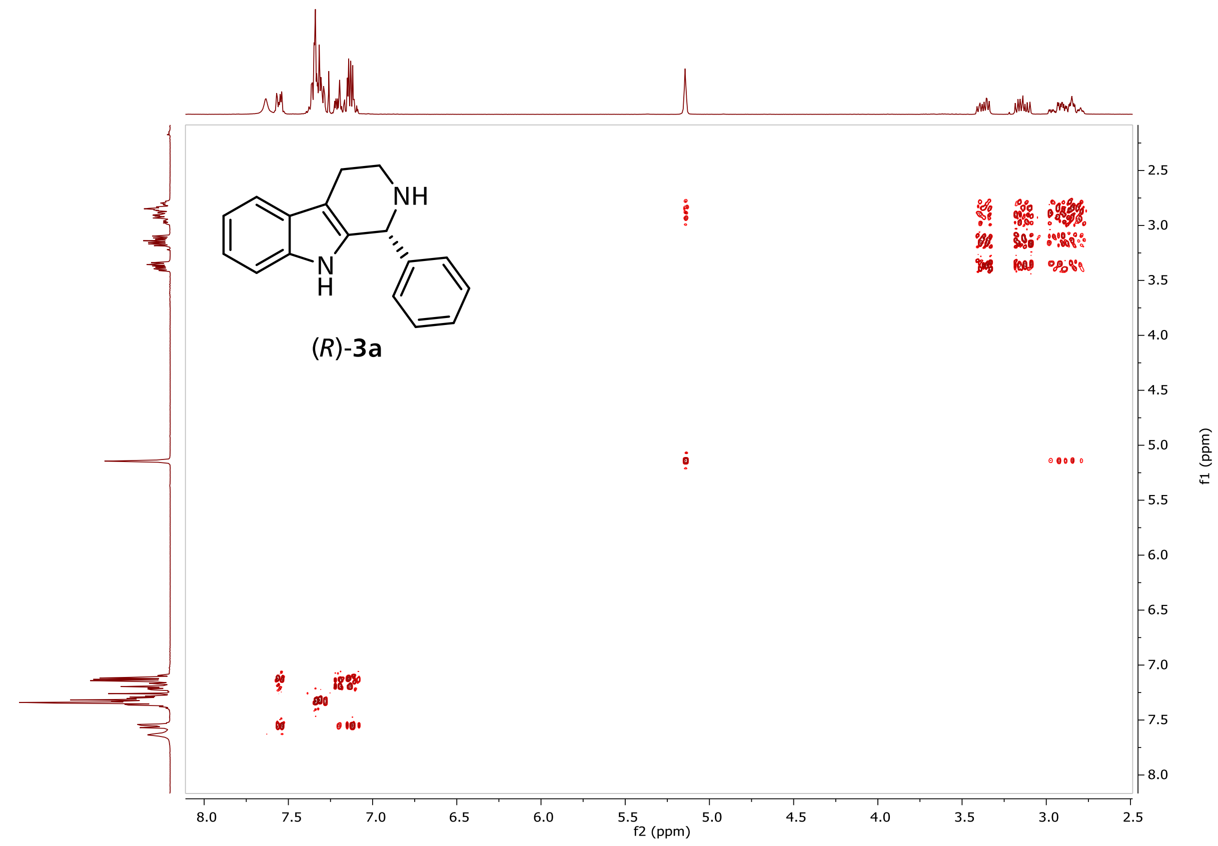

Figure S96. COSY-NMR spectrum of (*R*)-**3a**.

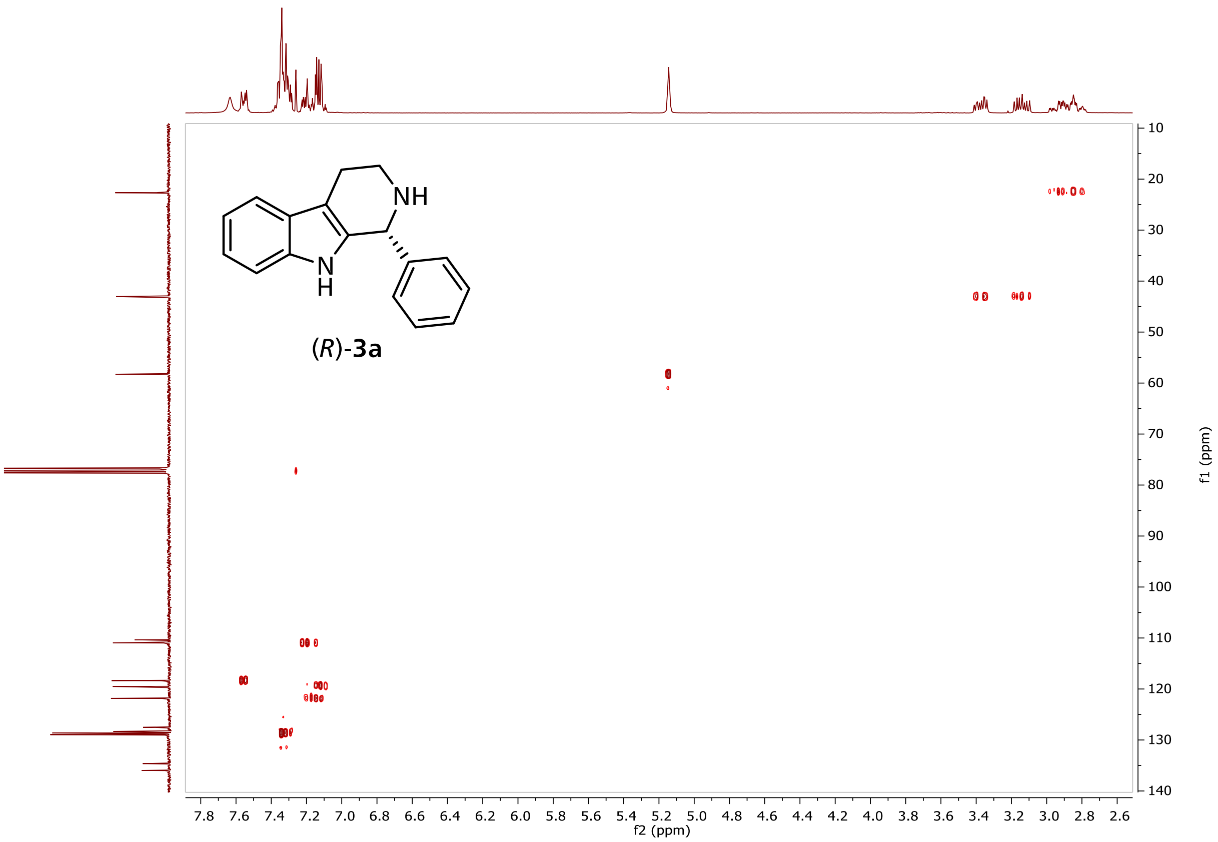

Figure S97. HSQC-NMR spectrum of (*R*)-**3a**.

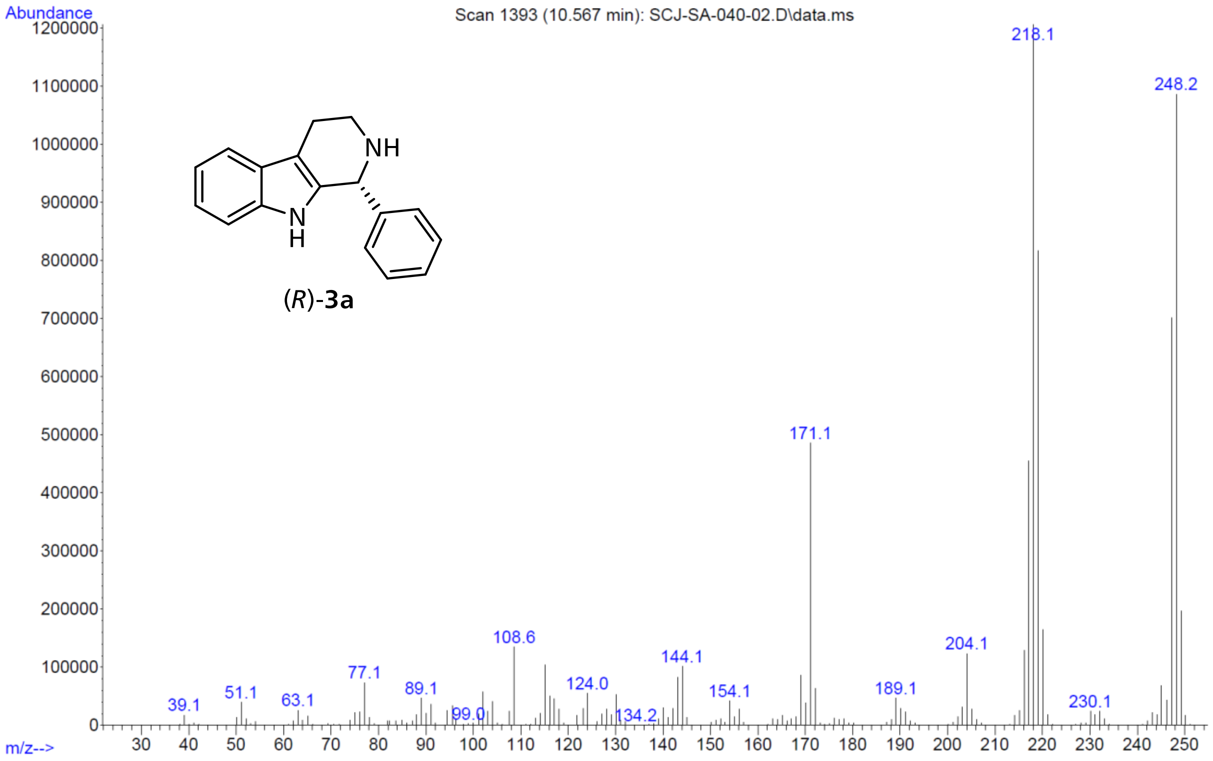

Figure S98. MS spectrum of (*R*)-**3a**.

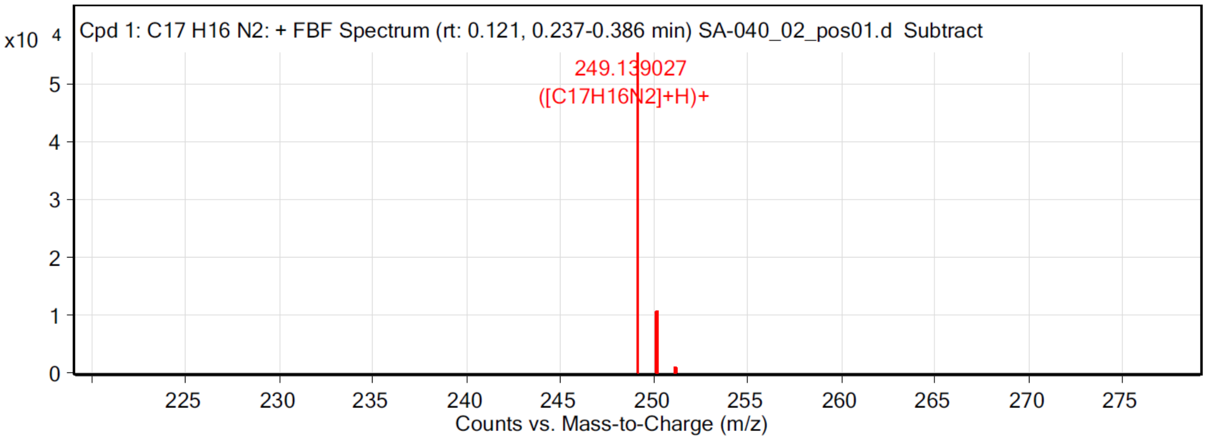

Figure S99. HRMS spectrum of (*R*)-**3a**.

(*R*)-1-(4-Fluorophenyl)-2,3,4,9-tetrahydro-1*H*-pyrido[3,4-*b*]indole (3b)

- ▶ ^{1}H-NMR spectrum (Figure S100)
- ▶ ^{13}C-NMR spectrum (Figure S101)
- ▶ DEPT135-NMR spectrum (Figure S102)
- ▶ DEPT90-NMR spectrum (Figure S103)
- ▶ COSY-NMR spectrum (Figure S104)
- ▶ HSQC-NMR spectrum (Figure S105)
- ▶ MS spectrum (Figure S106)
- ▶ HRMS spectrum (Figure S107)

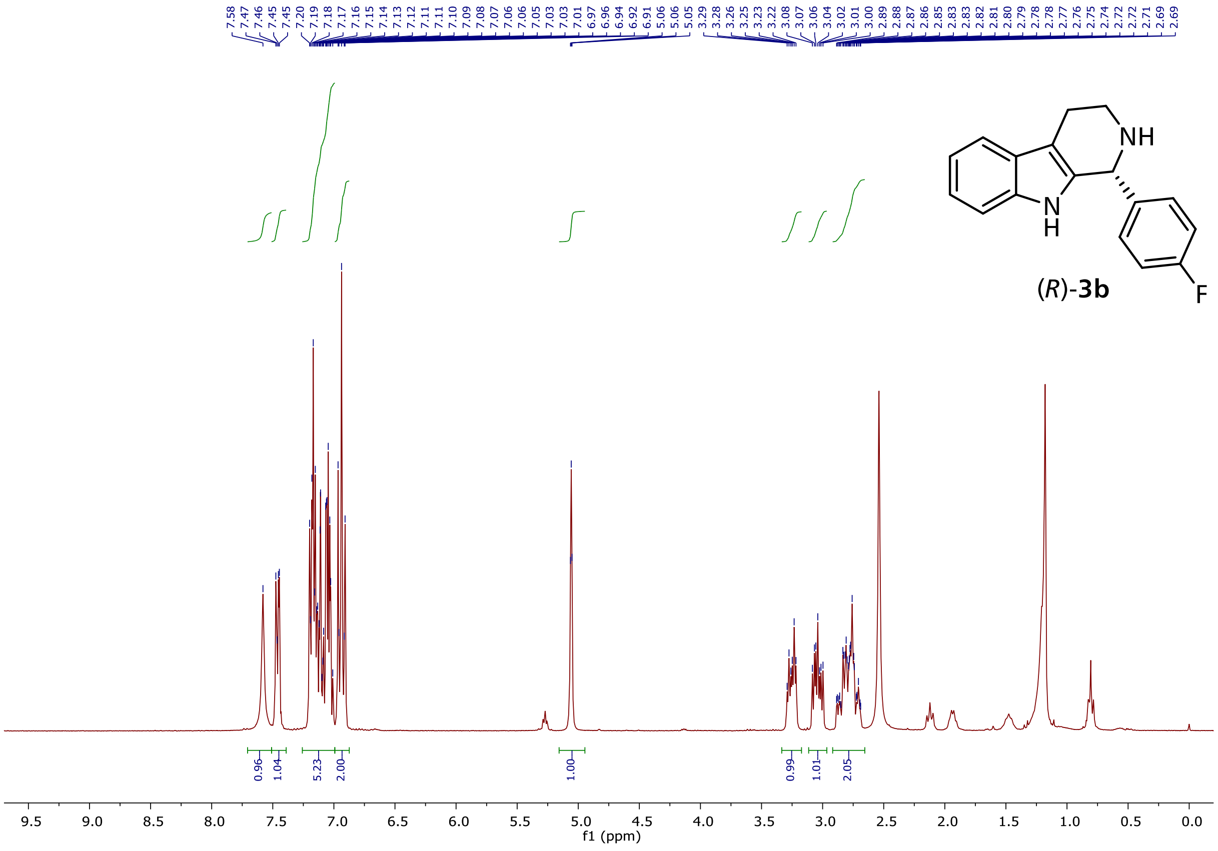

Figure S100. ^{1}H-NMR spectrum of (*R*)-**3b**.

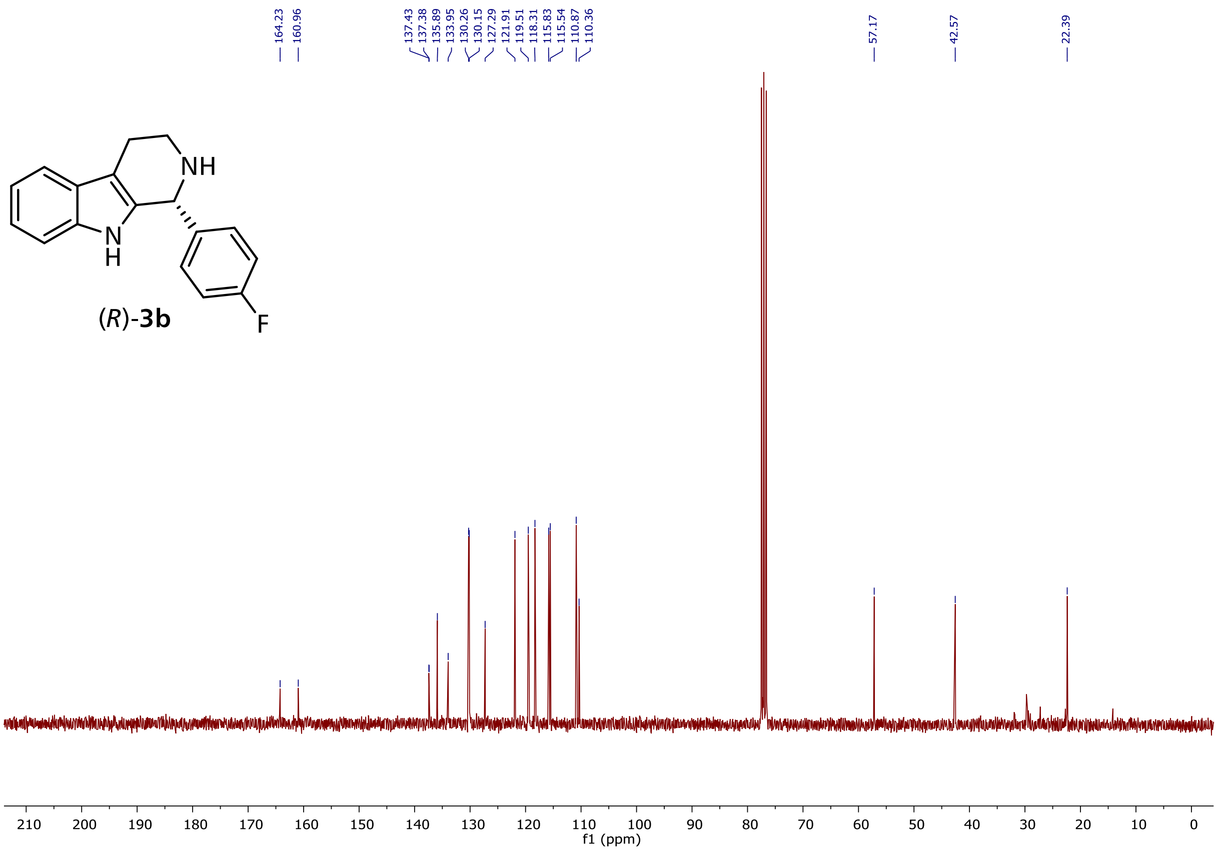

Figure S101. ^{13}C-NMR spectrum of (*R*)-**3b**.

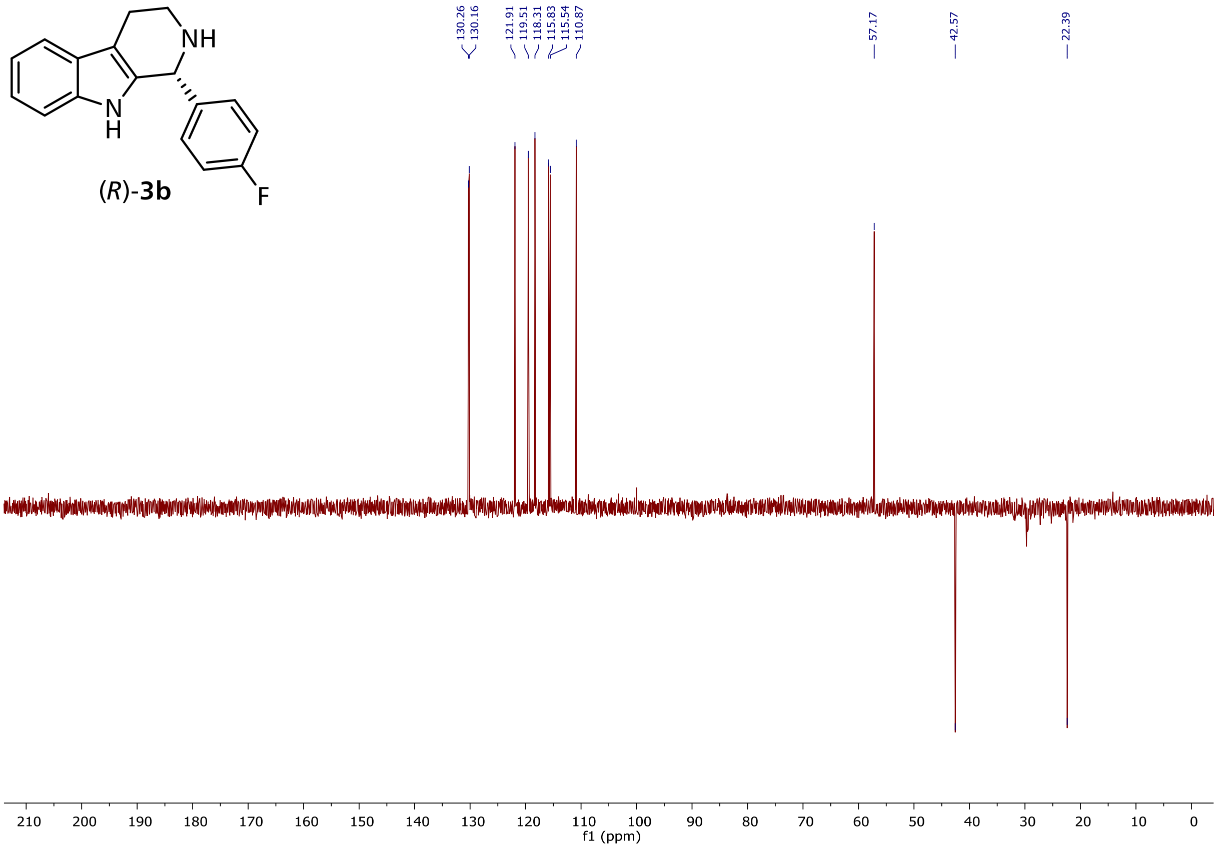

Figure S102. DEPT135-NMR spectrum of (*R*)-**3b**.

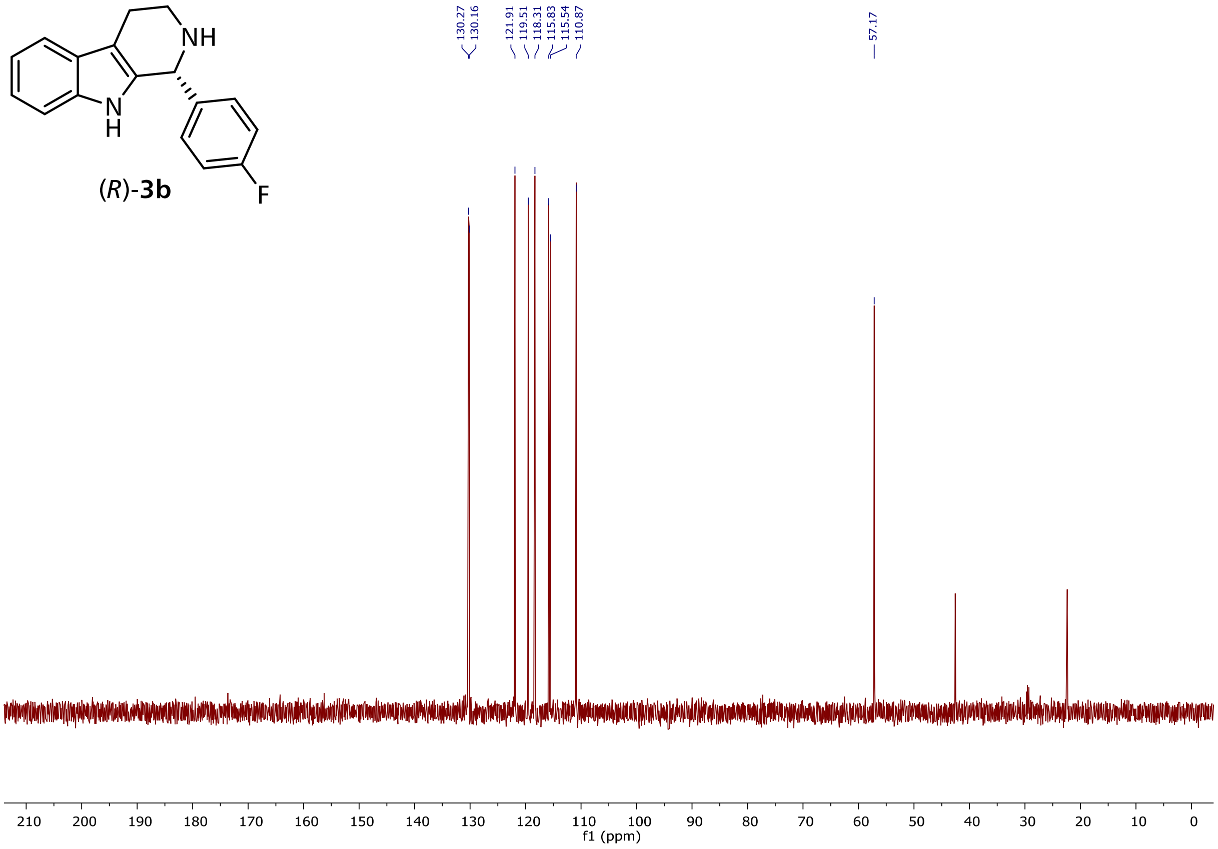

Figure S103. DEPT90-NMR spectrum of (*R*)-**3b**.

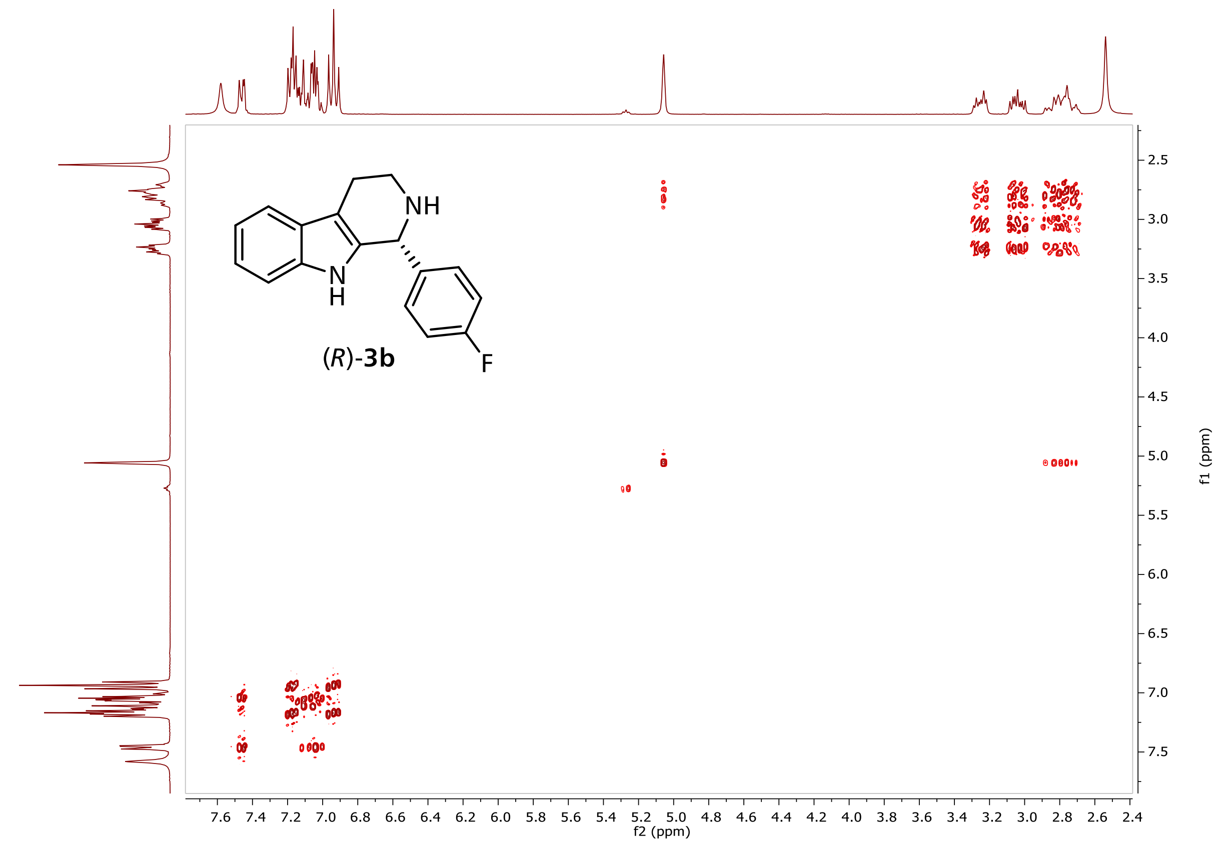

Figure S104. COSY-NMR spectrum of (*R*)-**3b**.

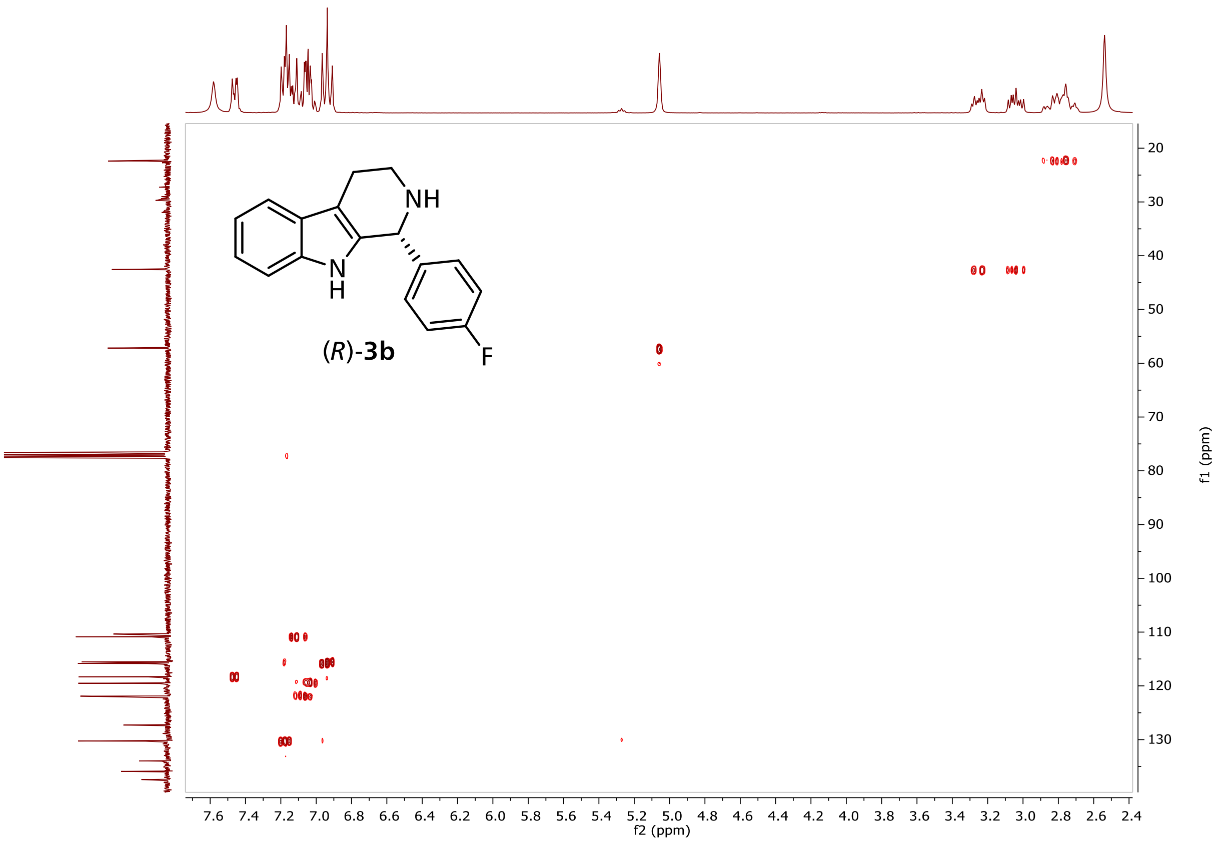

Figure S105. HSQC-NMR spectrum of (*R*)-**3b**.

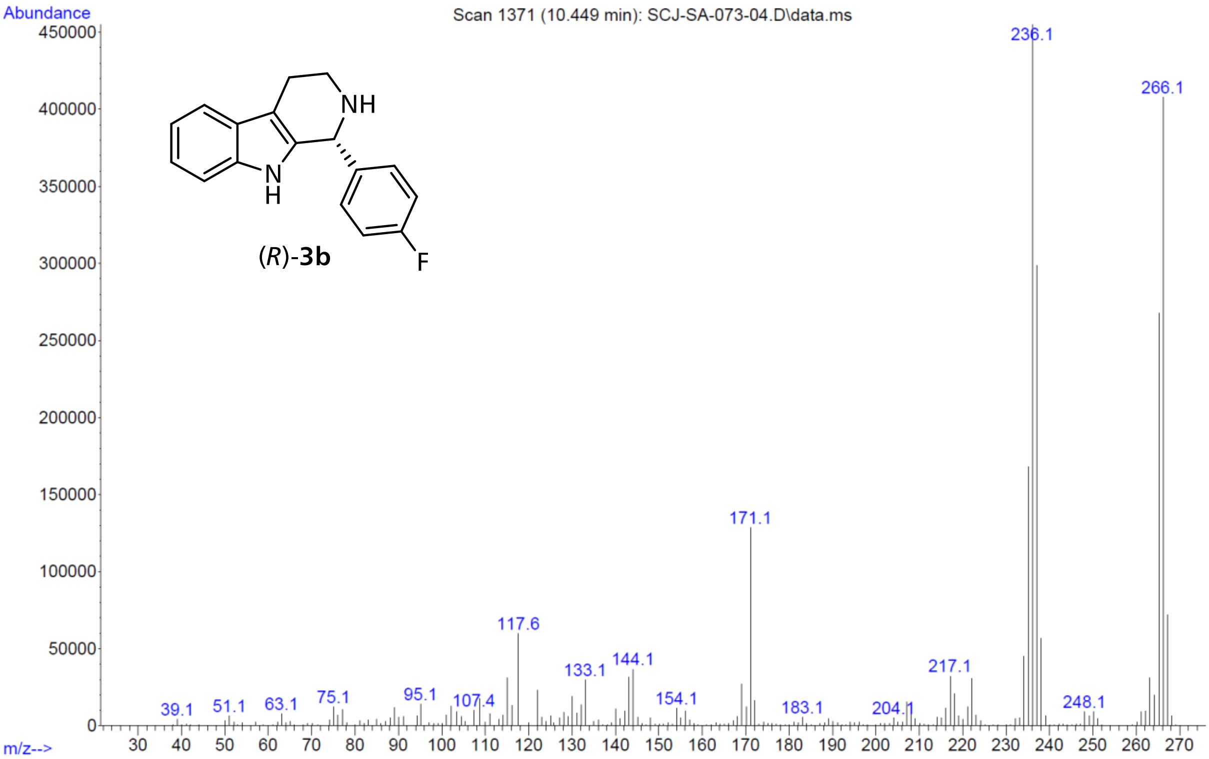

Figure S106. MS spectrum of (*R*)-**3b**.

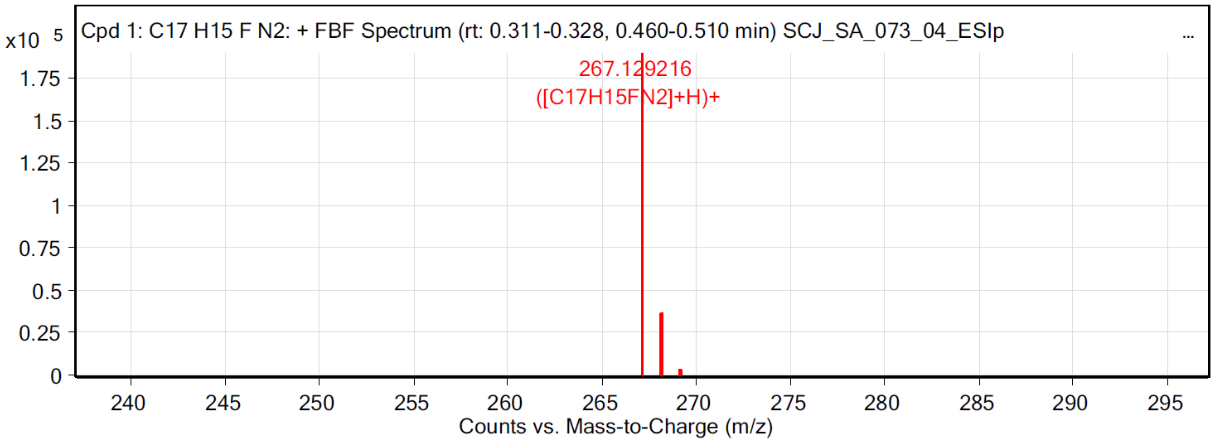

Figure S107. HRMS spectrum of (*R*)-**3b**.

(*R*)-1-(2-Fluorophenyl)-2,3,4,9-tetrahydro-1*H*-pyrido[3,4-*b*]indole (3h)

- ^{1}H-NMR spectrum (Figure S108)
- ^{13}C-NMR spectrum (Figure S109)
- DEPT135-NMR spectrum (Figure S110)
- DEPT90-NMR spectrum (Figure S111)
- COSY-NMR spectrum (Figure S112)
- HSQC-NMR spectrum (Figure S113)
- MS spectrum (Figure S114)
- HRMS spectrum (Figure S115)

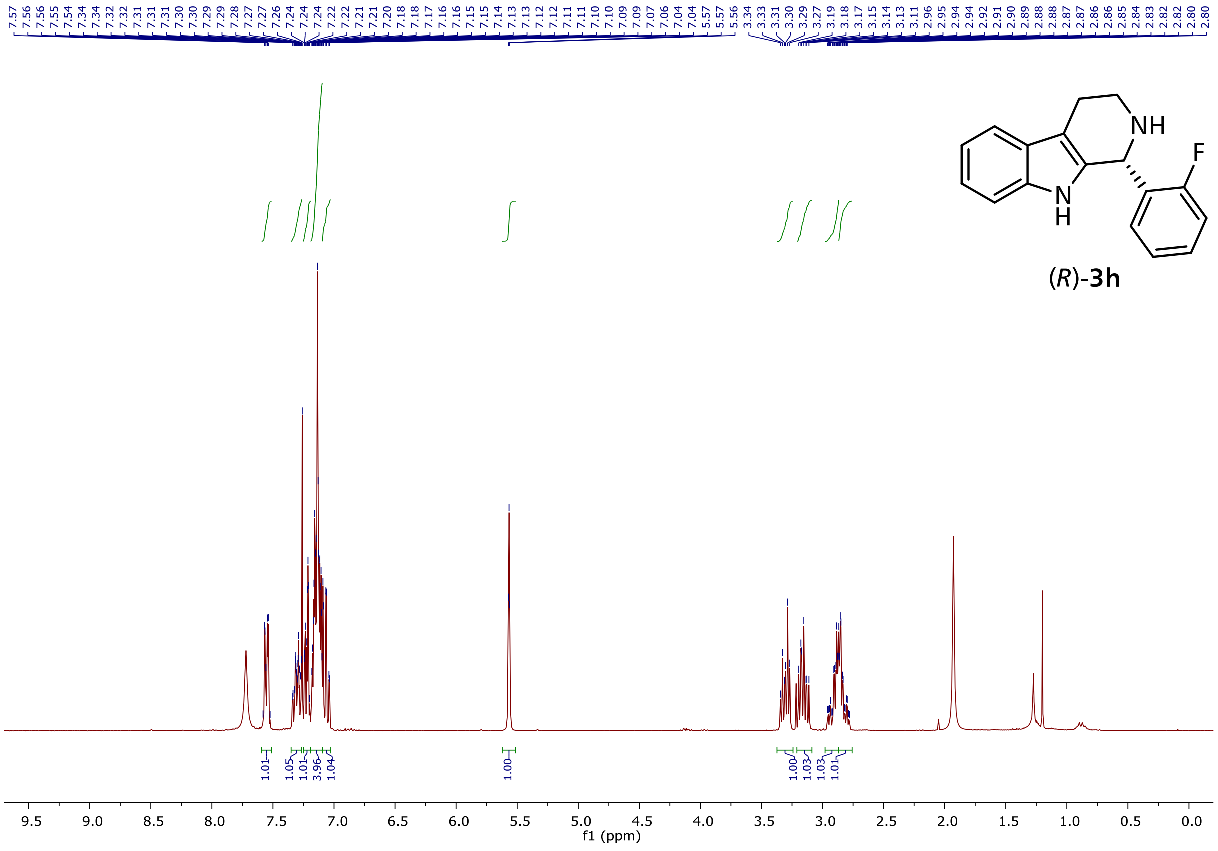

Figure S108. ^{1}H-NMR spectrum of (*R*)-**3h**.

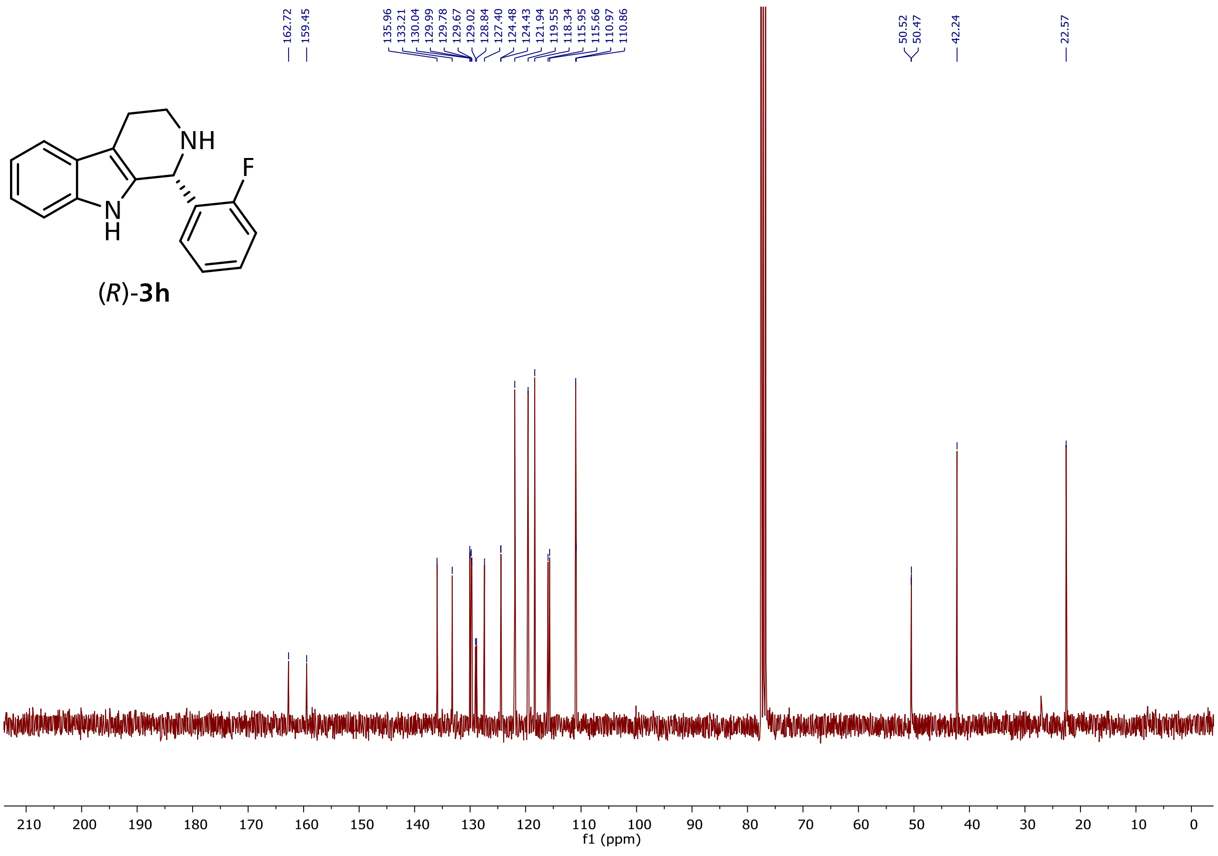

Figure S109. ^{13}C-NMR spectrum of (*R*)-**3h**.

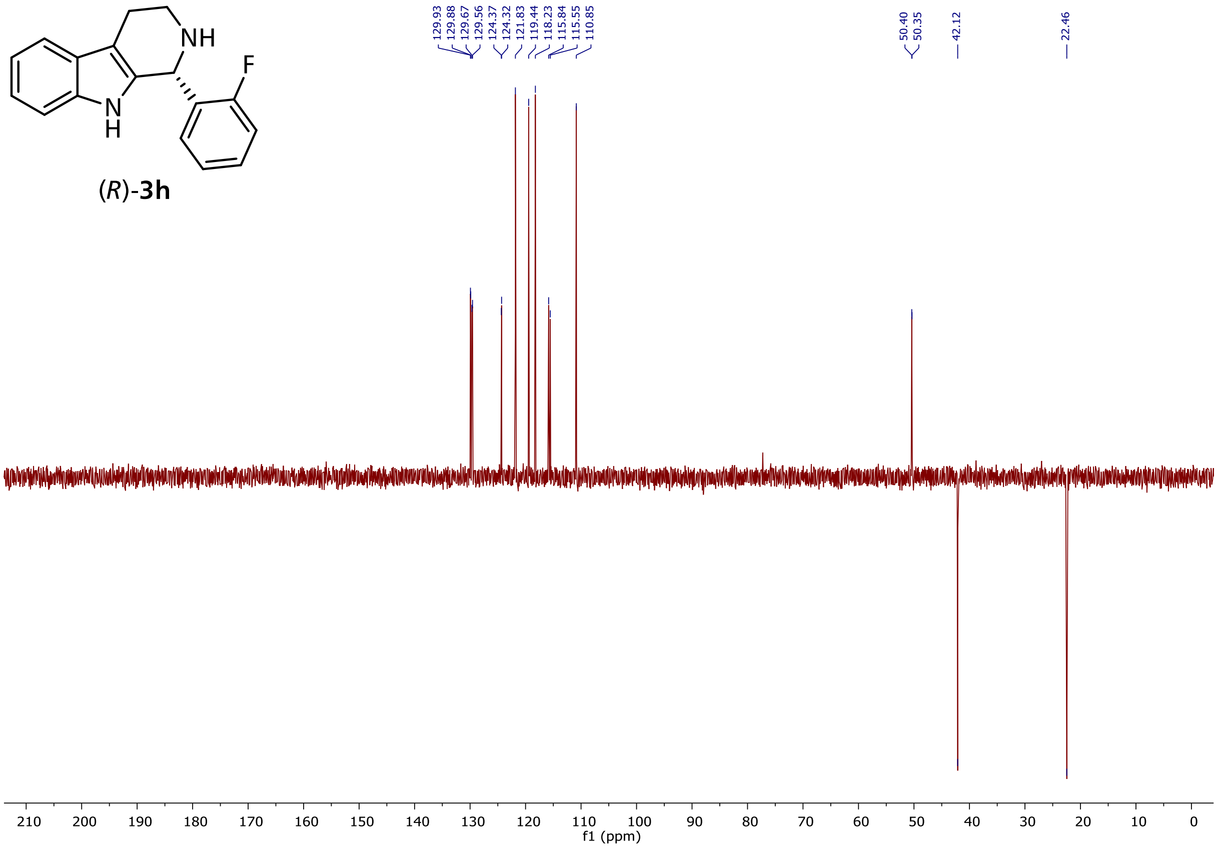

Figure S110. DEPT135-NMR spectrum of (*R*)-**3h**.

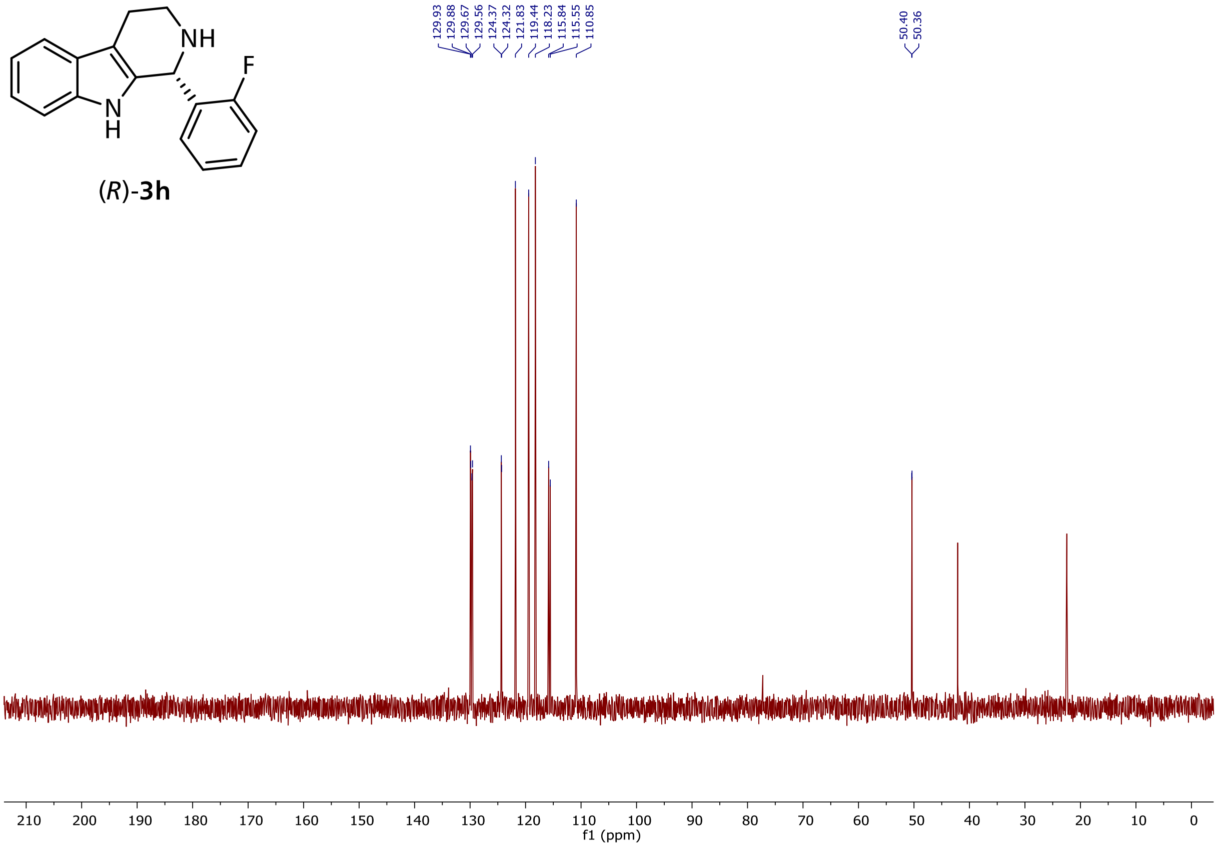

Figure S111. DEPT90-NMR spectrum of (*R*)-**3h**.

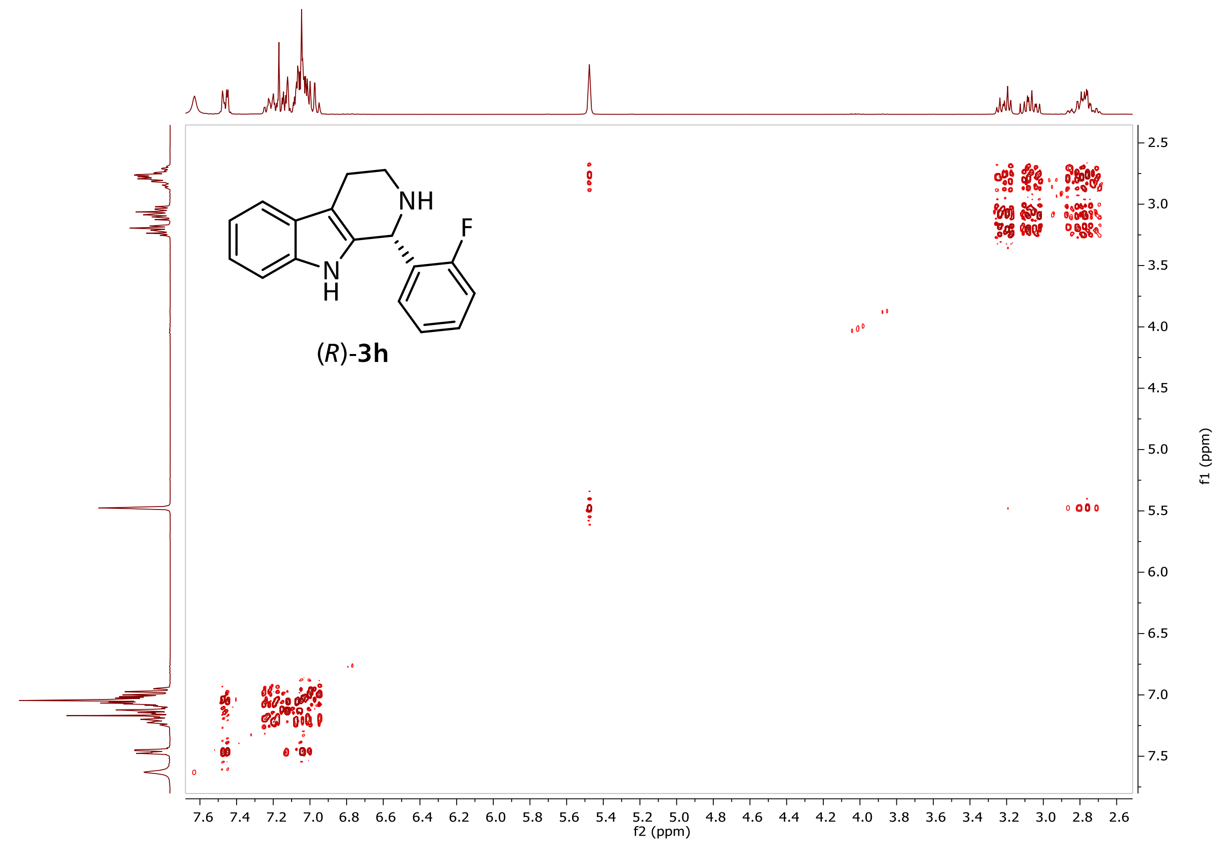

Figure S112. COSY-NMR spectrum of (*R*)-**3h**.

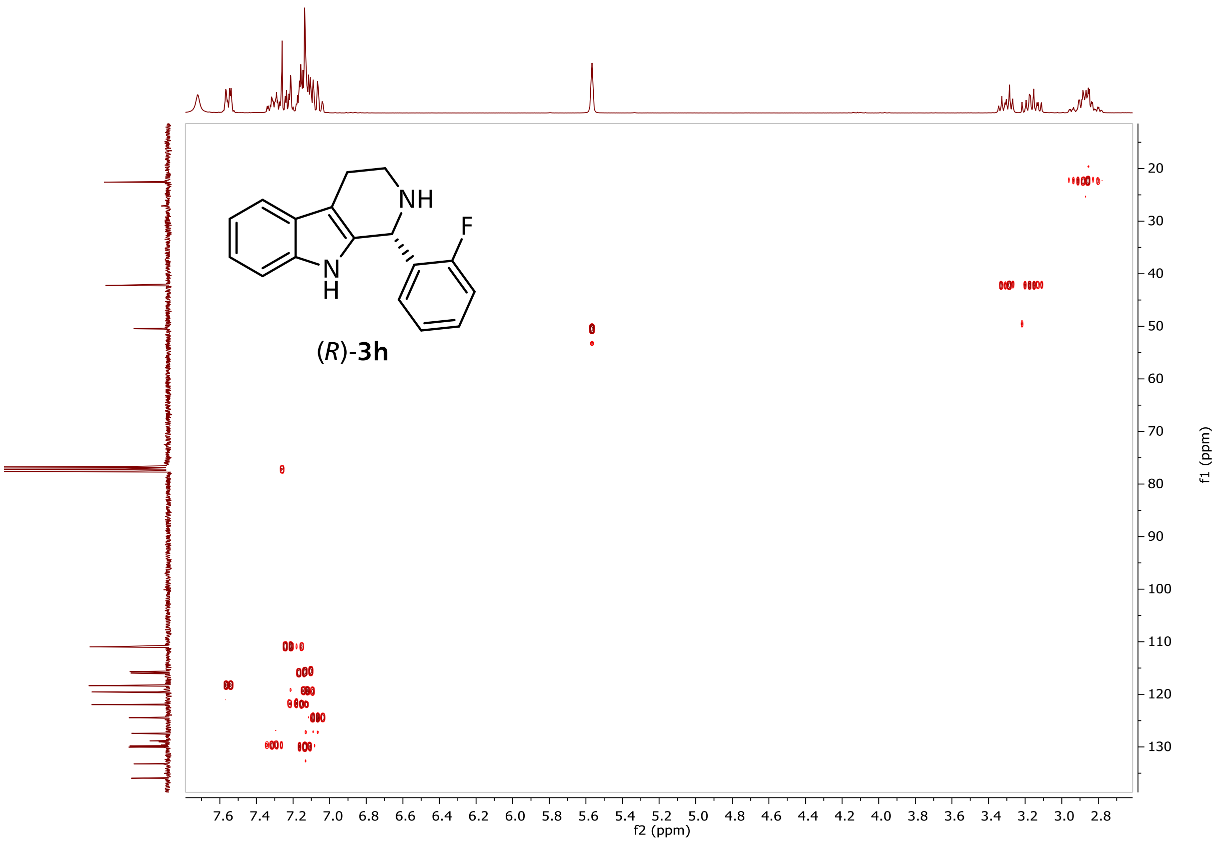

Figure S113. HSQC-NMR spectrum of (*R*)-**3h**.

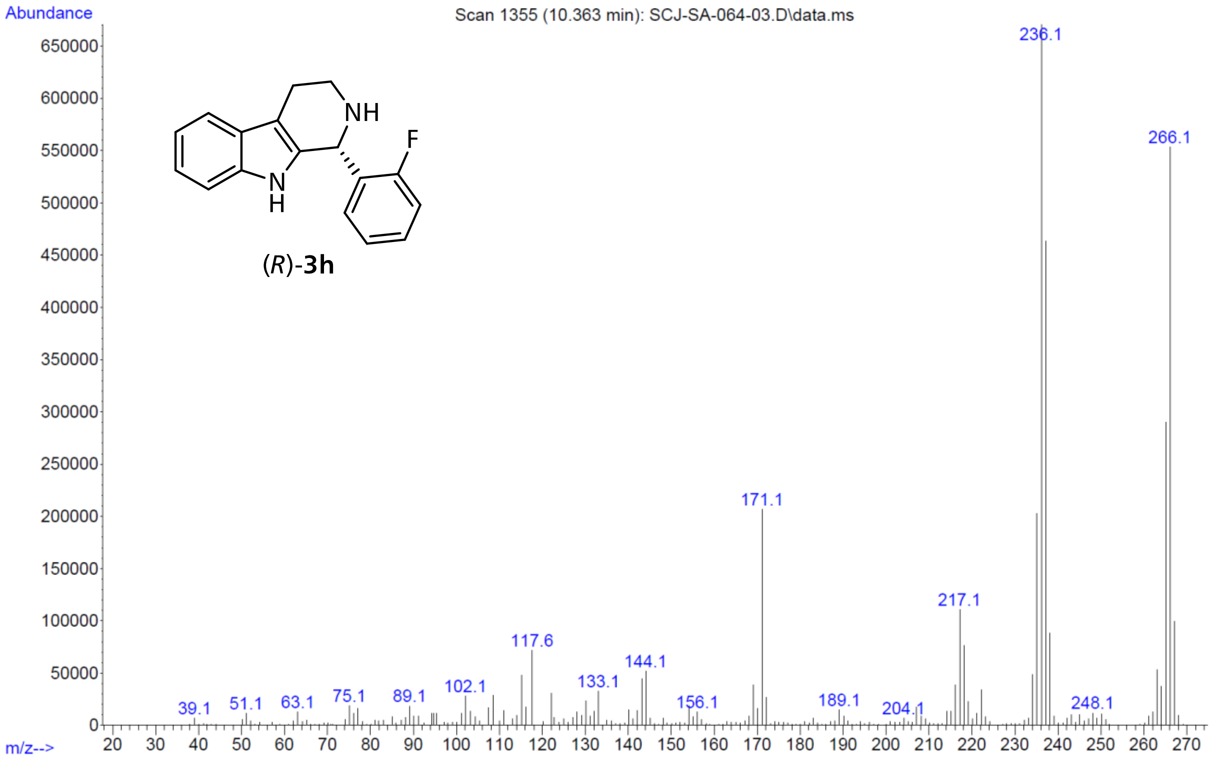

Figure S114. MS spectrum of (*R*)-**3h**.

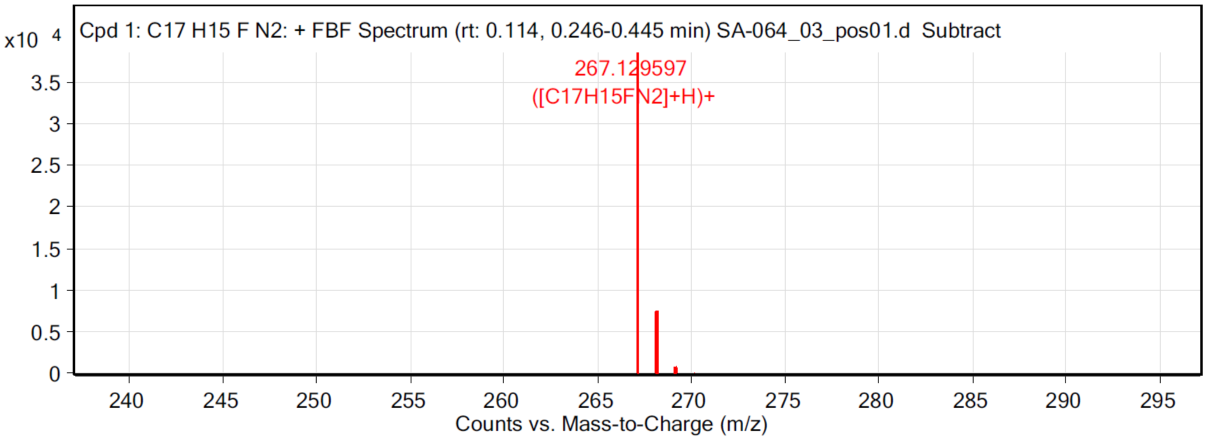

Figure S115. HRMS spectrum of (*R*)-**3h**.

(*R*)-1-(2-Bromophenyl)-2,3,4,9-tetrahydro-1*H*-pyrido[3,4-*b*]indole (3i)

- ▶ ^{1}H-NMR spectrum (Figure S116)
- ▶ ^{13}C-NMR spectrum (Figure S117)
- ▶ DEPT135-NMR spectrum (Figure S118)
- ▶ DEPT90-NMR spectrum (Figure S119)
- ▶ COSY-NMR spectrum (Figure S120)
- ▶ HSQC-NMR spectrum (Figure S121)
- ▶ MS spectrum (Figure S122)
- ▶ HRMS spectrum (Figure S123)

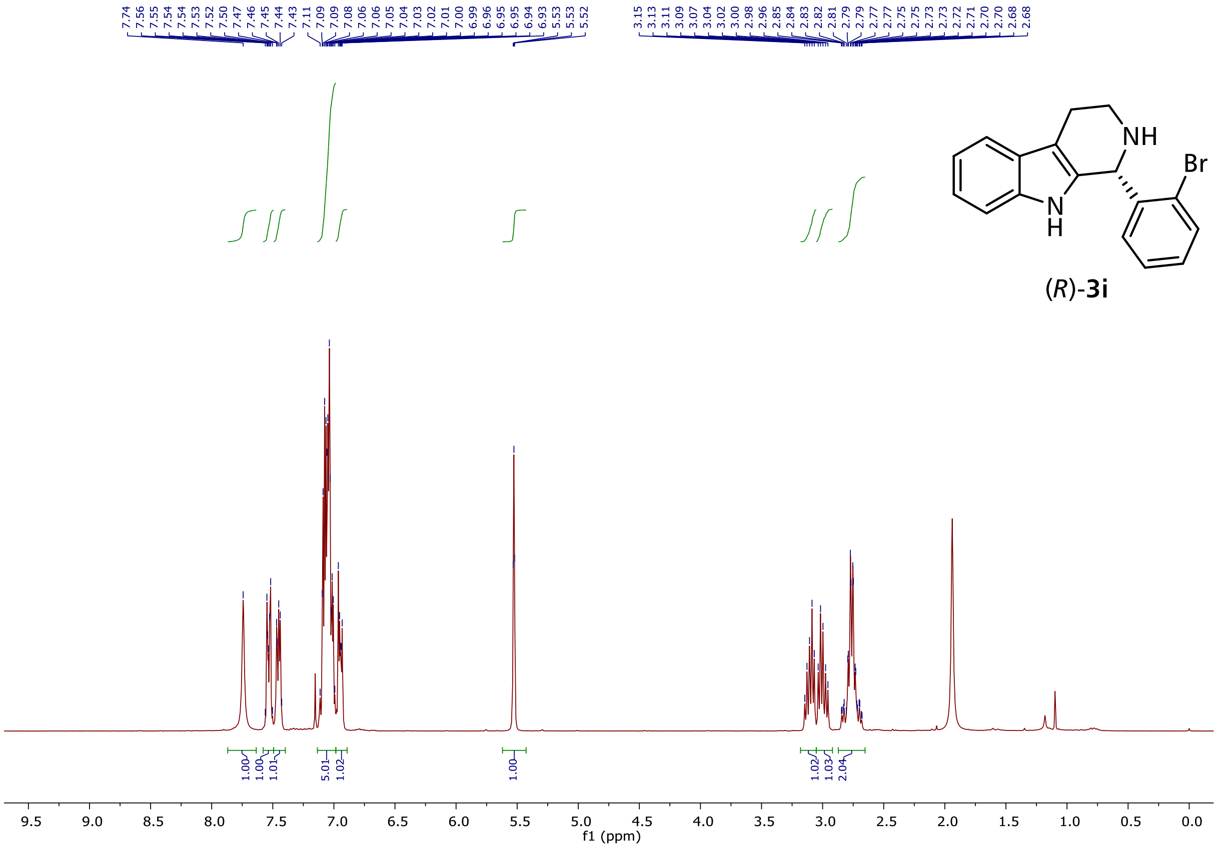

Figure S116. ^{1}H-NMR spectrum of (*R*)-**3i**.

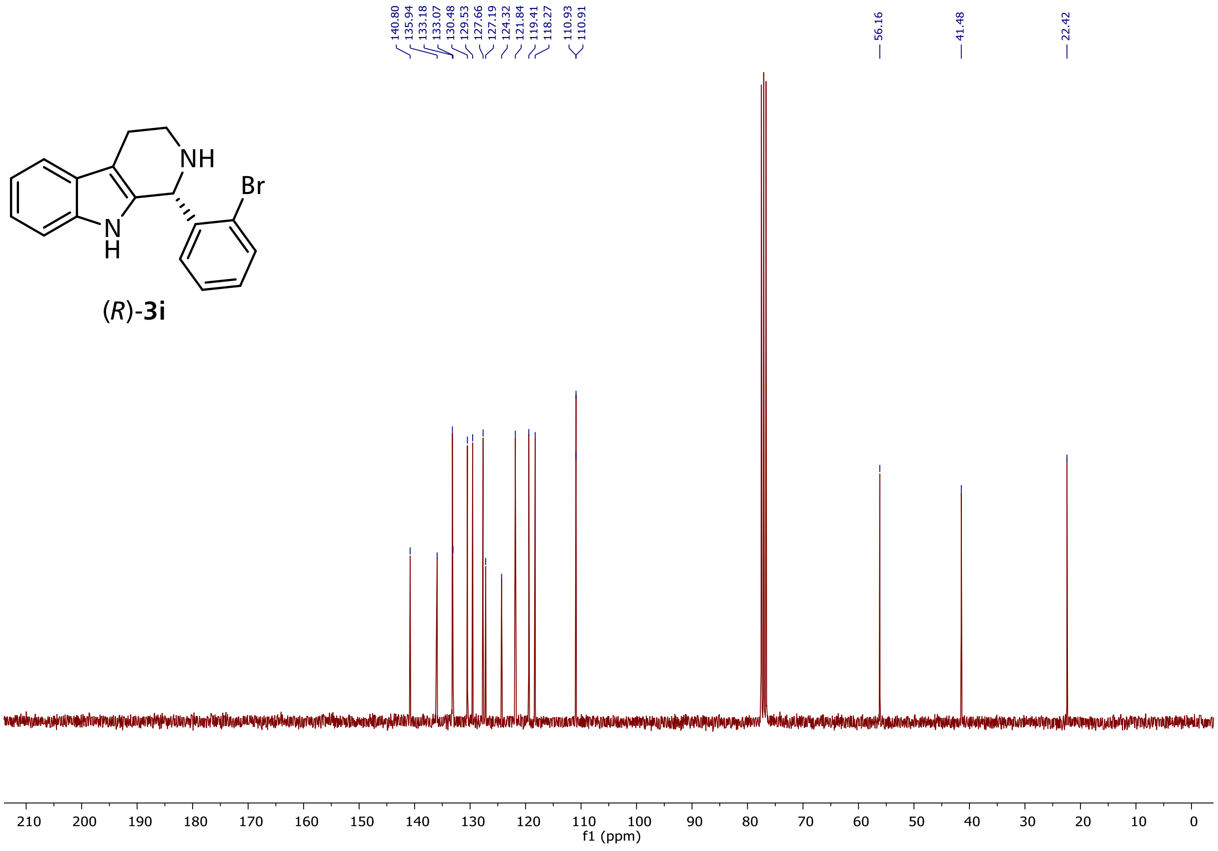

Figure S117. ^{13}C-NMR spectrum of (*R*)-**3i**.

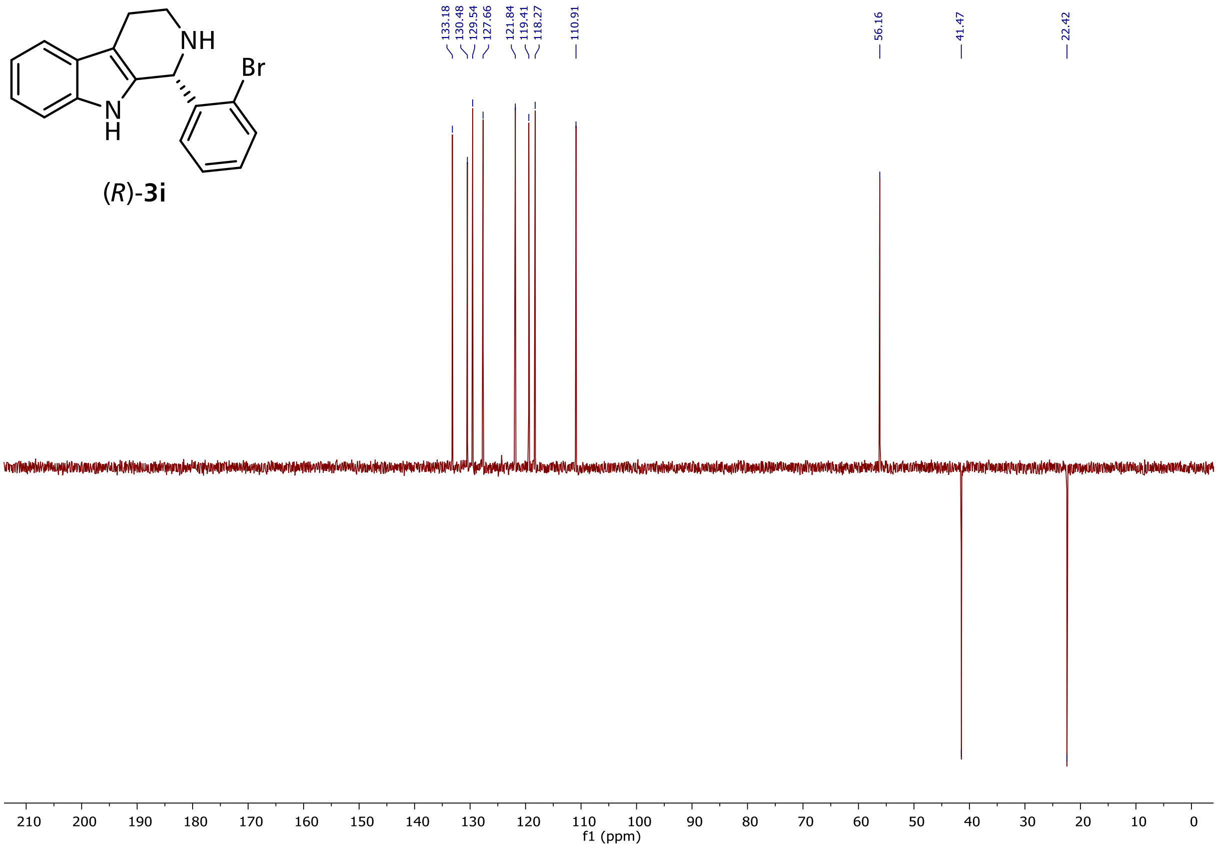

Figure S118. DEPT135-NMR spectrum of (*R*)-**3i**.

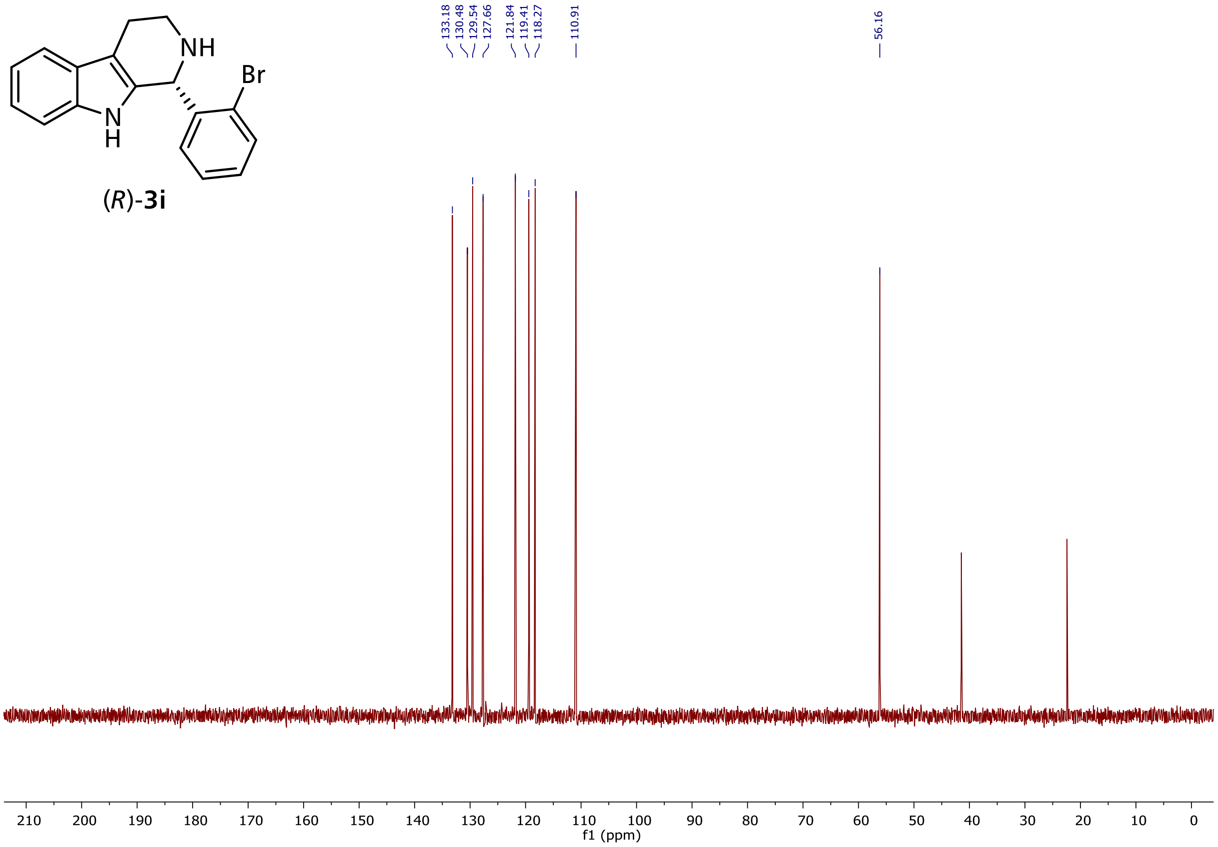

Figure S119. DEPT90-NMR spectrum of (*R*)-**3i**.

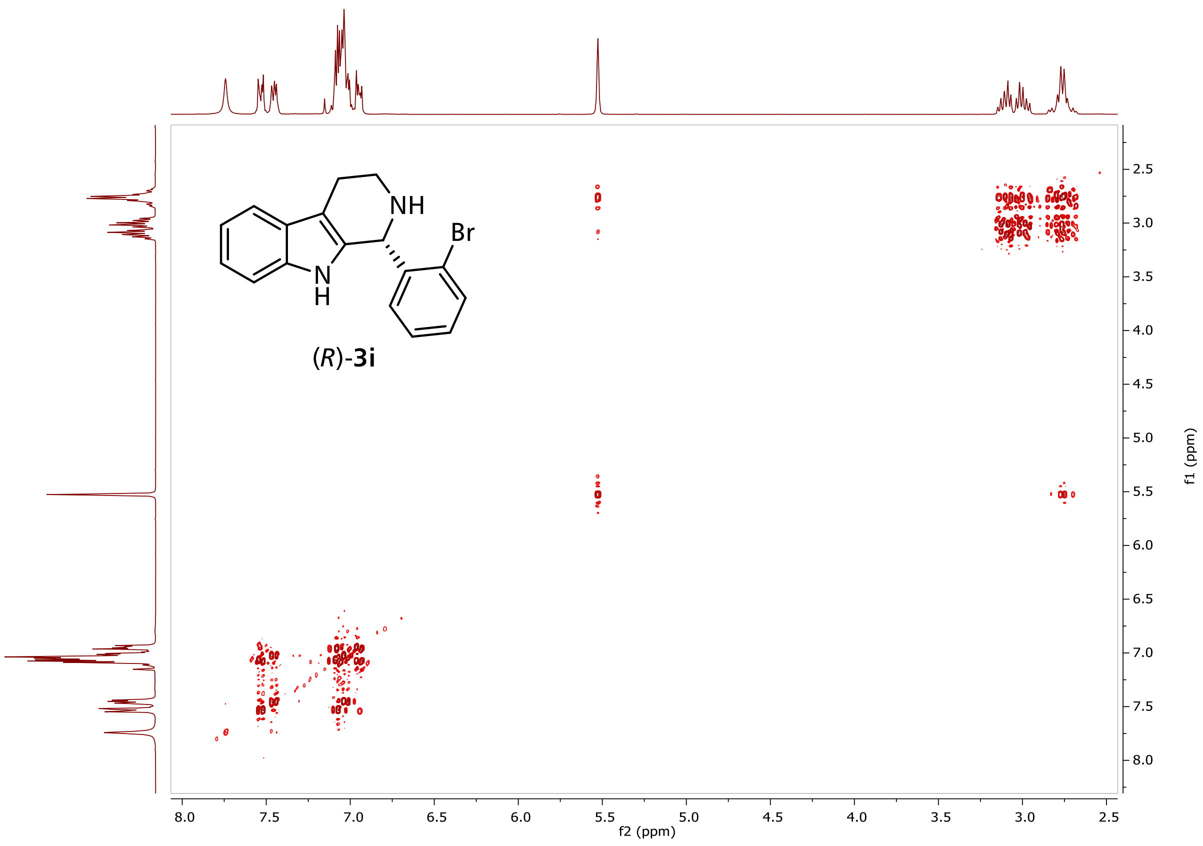

Figure S120. COSY-NMR spectrum of (*R*)-**3i**.

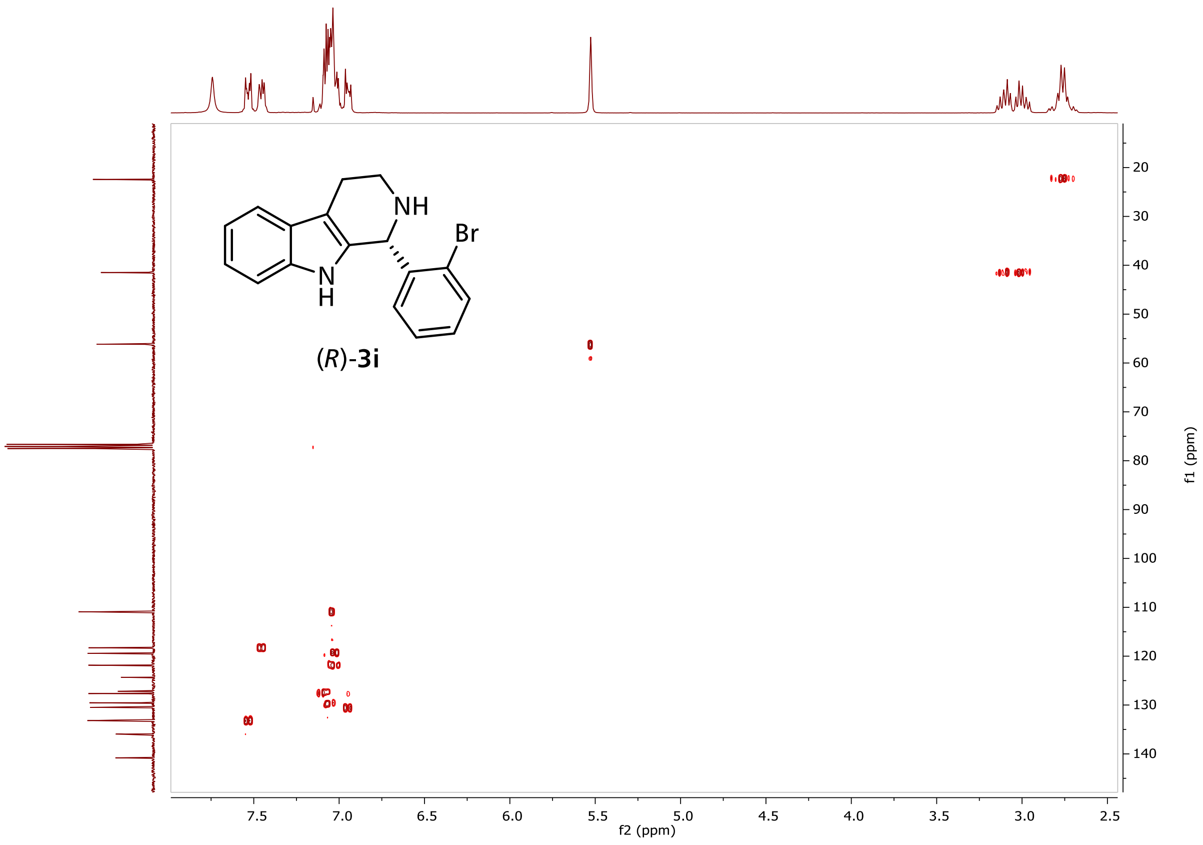

Figure S121. HSQC-NMR spectrum of (*R*)-**3i**.

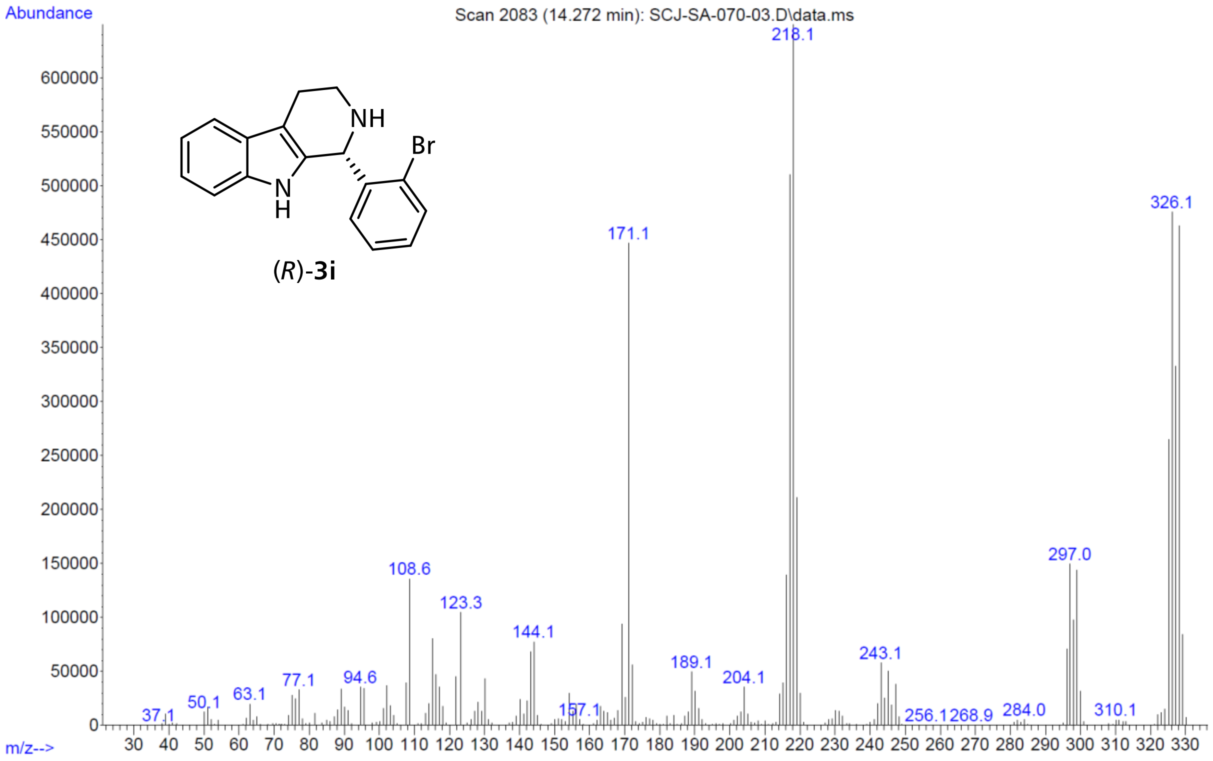

Figure S122. MS spectrum of (*R*)-**3i**.

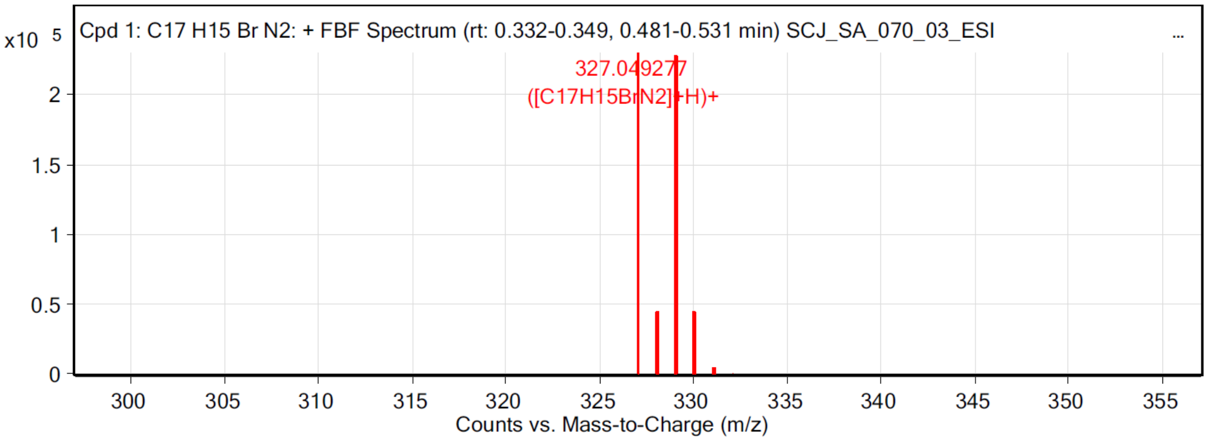

Figure S123. HRMS spectrum of (*R*)-**3i**.

References

[1] Swiss Institute of Bioinformatics, ExPASy Bioinformatics Resource Portal – ProtParam; available online at: https://web.expasy.org/protparam/ (accessed: 02.10.2019).

[2] D. Pressnitz, E.-M. Fischereder, J. Pletz, C. Kofler, L. Hammerer, K. Hiebler, H. Lechner, N. Richter, E. Eger, W. Kroutil, *Angew. Chem. Int. Ed.* **2018**, *57*, 10683–10687.

[3] R. S. Heath, M. Pontini, B. Bechi, N. J. Turner, *ChemCatChem* **2014**, *6*, 996–1002.

[4] S. Eagon, M. O. Anderson, *Eur. J. Org. Chem.* **2014**, *2014*, 1653–1665.

[5] D. Prajapati, M. Gohain, *Synth. Commun.* **2008**, *38*, 4426–4433.

[6] F. Liu, Q.-D. You, *Synth. Commun.* **2007**, *37*, 3933–3938.

[7] N. E. Agafonov, A. V. Dudin, A. A. Preobrazhenskii, V. M. Zhulin, *Russ. Chem. Bull.* **2001**, *50*, 560–562.

[8] Y.-C. Shen, C.-Y. Chen, P.-W. Hsieh, C.-Y. Duh, Y.-M. Lin, C.-L. Ko, *Chem. Pharm. Bull.* **2005**, *53*, 32–36.

[9] E. A. Loris, S. Panjikar, M. Ruppert, L. Barleben, M. Unger, H. Schübel, J. Stöckigt, *Chem. Biol.* **2007**, *14*, 979–985.

[10] E. Eger, A. Simon, M. Sharma, S. Yang, W. B. Breukelaar, G. Grogan, K. N. Houk, W. Kroutil, *J. Am. Chem. Soc.* **2020**, *142*, 792–800.

[11] a) M. T. Reetz, M. Bocola, J. D. Carballeira, D. Zha, A. Vogel, *Angew. Chem. Int. Ed.* **2005**, *44*, 4192–4196; b) M. T. Reetz, L.-W. Wang, M. Bocola, *Angew. Chem. Int. Ed.* **2006**, *45*, 1236–1241; c) J. Xu, Y. Cen, W. Singh, J. Fan, L. Wu, X. Lin, J. Zhou, M. Huang, M. T. Reetz, Q. Wu, *J. Am. Chem. Soc.* **2019**, *141*, 7934–7945; d) M. T. Reetz, J. D. Carballeira, *Nat. Protoc.* **2007**, *2*, 891–903.

[12] A. Simon, S. Yang, K. N. Houk, personal communication.

[13] a) Integrated DNA Technologies, OligoAnalyzer Tool; available online at: https://eu.idtdna.com/pages/tools (accessed: 08.08.2019); b) R. Owczarzy, A. V. Tataurov, Y. Wu, J. A. Manthey, K. A. McQuisten, H. G. Almabrazi, K. F. Pedersen, Y. Lin, J. Garretson, N. O. McEntaggart, C. A. Sailor, R. B. Dawson, A. S. Peek, *Nucleic Acids Res.* **2008**, *36*, W163–W169.

[14] H. Waldmann, G. Schmidt, M. Jansen, J. Geb, *Tetrahedron* **1994**, *50*, 11865–11884.

[15] T. Kawate, M. Yamanaka, M. Nakagawa, *Heterocycles* **1999**, *50*, 1033–1039.

[16] I. Kreituss, K.-Y. Chen, S. H. Eitel, J.-M. Adam, G. Wuitschik, A. Fettes, J. W. Bode, *Angew. Chem. Int. Ed.* **2016**, *55*, 1553–1556.

[17] R. S. Klausen, E. N. Jacobsen, *Org. Lett.* **2009**, *11*, 887–890.

[18] *Molecular Operating Environment (MOE)*, v. 2018.01, Chemical Computing Group ULC, Montreal, Canada, **2018**.

[19] M. M. Hamed, R. H. Abu-Eittah, Z. Mobark, M. M. Abdou, *Int. J. Quantum Chem.* **1992**, *44*, 379–392.

[20] P. Bernhardt, E. McCoy, S. E. O'Connor, *Chem. Biol.* **2007**, *14*, 888–897.
